# Synergy-Promoted Specific Alkyltriphenylphosphonium Binding to CB[8]

**DOI:** 10.1021/acs.joc.4c02546

**Published:** 2025-02-10

**Authors:** Mauro Díaz-Abellás, Iago Neira, Arturo Blanco-Gómez, Carlos Peinador, Marcos D. García

**Affiliations:** Departamento de Química and Centro Interdisciplinar de Química y Biología (CICA). Facultad de Ciencias, 16737 Universidade da Coruña, A Coruña 15071, Spain

## Abstract

Biological substrate specificity ensures that organisms interact accurately with biomolecular receptors, crucial for key functions such as signaling and immunity. Nevertheless, this phenomenon is still poorly understood, with host–guest chemistry offering a suitable platform for studying simplified models. Herein, we report an in-depth study of the host–guest chemistry of alkyltriphenylphosphonium cations with cucurbit[8]­uril (CB[8]), initiated by the serendipitous discovery of salt forming a tightly bound pseudoheteroternary 1:1 complex with CB[8]. A first generation of model substrates was designed to explore an unusual binding mode characterized by the simultaneous introduction of two distinct guest fragments within the host cavity. Structural features of the complexes were elucidated using ESI-MS and NMR 1D/2D techniques; thermodynamic properties were assessed by isothermal titration calorimetry, and kinetic parameters were derived from selective inversion–recovery NMR. Experimental results aligned well with electronic structure calculations, revealing a reproducible binding motif with submicromolar affinities. This peculiar complexation mode involves a synergistic effect caused by steric crowding around the P^+^ atom, facilitating the insertion of two aromatic units into CB[8] while hindering association with CB[7]. Based on these findings, a second generation of minimalistic substrates was developed, preserving the synergistic interaction mode and exhibiting specific binding to CB[8].

## Graphical abstract

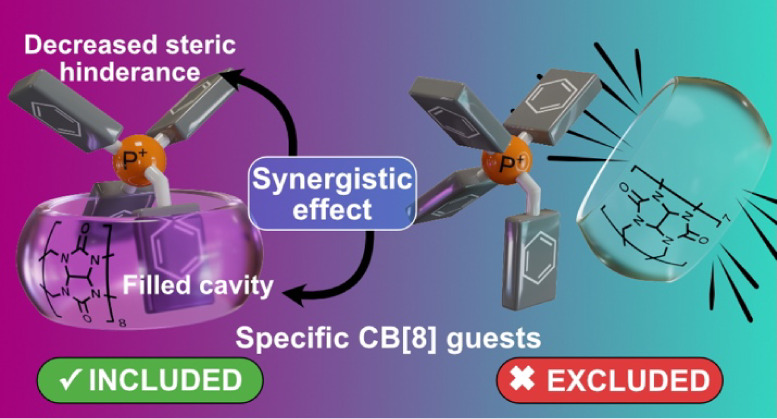

## 1 Introduction

The macrocycle cucurbit[8]­uril (CB[8])^1^ has significantly transformed supramolecular chemistry^2,3^ due to its rare ability to form either binary or ternary inclusion complexes and, crucially, because of the apparent predictability in the design of those in terms of stoichiometry and association constants. In essence, like the smaller members of the CB­[n] family, CB[8] forms binary complexes with positively charged substrates of the likes of viologens (**V** ^2+^), with hydrophobic effects being the main guiding force of the process, accompanied to a lesser extent by other attractive interactions such as dispersion, cation-dipole, or hydrogen bonding.^2^ Considering these lax requisites, homo/heteroternary complexes can also be easily projected for CB[8], taking advantage as well in this case of cavity-enhanced interactions such as the pimerization of radical cations (e.g., (**V** ^+·^)_2_⊂CB­[8])^4^ or electron donor–acceptor charge transfer (e.g., **V** ^2+^·N⊂CB­[8]) (Figure 1a).^5^

**Figure 1:**
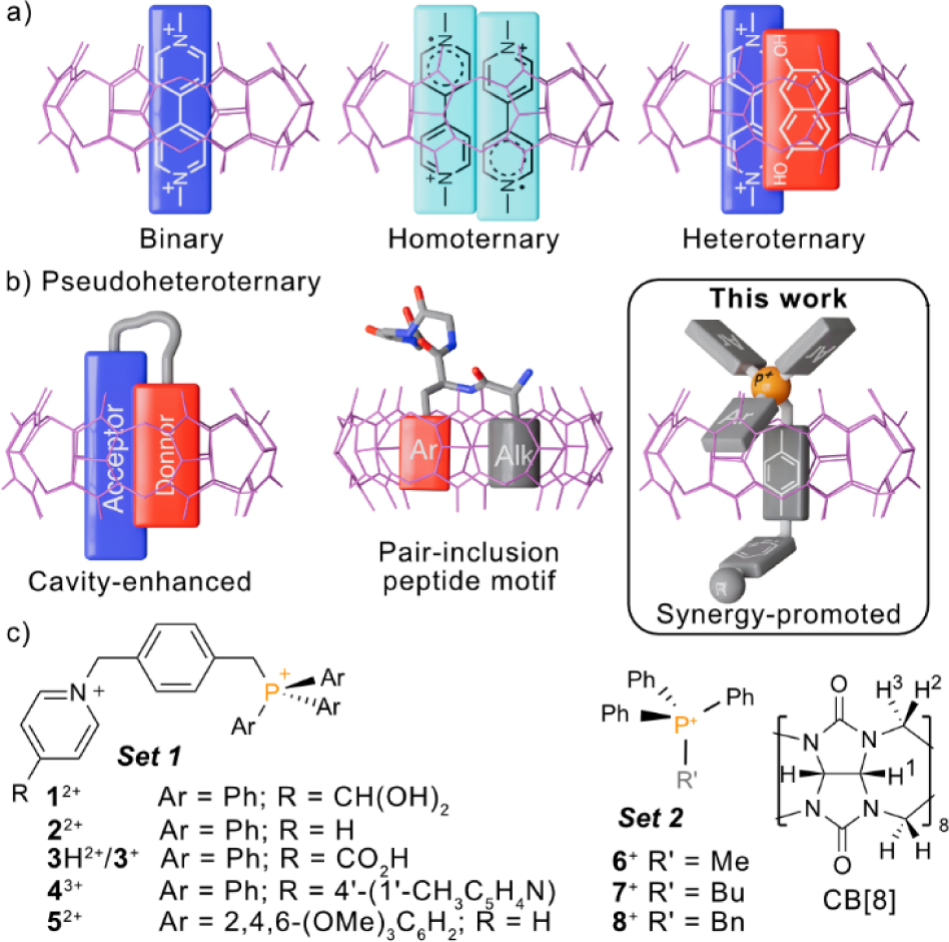
(a) Classical examples of binary and ternary CB[8]-based inclusion complexes. (b) Reported types of pseudoheteroternary complexes. (c) Chemical structures of model compounds in **sets 1** and **2** discussed in this work.

A notable exception to this robust predictability is associated with a more infrequent type of 1:1 complexes, termed in this work pseudoternary, in which two distinct structural fragments of a given guest are included within the cavity of CB[8] (Figure 1b).^6^ Despite some trivial examples that can be easily envisioned by covalently joining two appropriate moieties, as, for instance, in the pseudohomo/heteroternary versions of the above-mentioned pimerized or donor–acceptor guest pairs, other cases of this type of complexation are much less predictable. This unpredictability arises not from cavity-enhanced stabilizing effects but from the promiscuity of the receptor to fill up its hydrophobic cavity. For instance, this tendency of CB[8] has been extensively explored by Scherman and Urbach,^7^ developing “pair-inclusion dipeptide motifs” that illustrate both the potential of pseudoheteroternary complexation for the development of functional CB[8] high-affinity binders and the difficulties associated with their rational design and predictability.

Following our interest in CB­[n]-based chemistry,^8,9^ we have recently reported on the use of triphenylphosphonium groups (RP^+^Ph_3_) as potential stoppers for the creation of CB­[n]-based rotaxanes.^10,11^ Although we found this moiety appropriate for the task with CB[7], in the case of CB[8], it resulted in unpredicted pseudoheteroternary complexation.^11^ In particular, salt **1** ^2+^ (Figure 1c) was found, as expected, to dispose of its two charged atoms centered on each of the portals of the host but, surprisingly, as well to accommodate within the macrocycle both the xylyl moiety and one of the phenyl rings attached to the phosphorus atom, resulting in a tightly bound complex (*K*_*a*_ = (3.6 ± 0.7)·10^10^ M^–1^). Furthermore, we also found that CB[7] was able to complex **1** ^2+^ with a completely different binding mode, implying the sole introduction of the pyridinium group within its cavity.

Based on these observations and the high synthetic accessibility of RP^+^Ph_3_ derivatives, we envisioned an in-depth study of the complexation of this type of organic salt with CB[8]. Hence, a first group of analogues of **1** ^2+^ (**set 1**, Figure 1) was designed to reproduce the pseudoheteroternary motif, in an effort to assert the structural, thermodynamic, and kinetic features of the complexation. As we will see, the obtained results served us to evolve a second set of simplified guests (**set 2**, Figure 1), which delimits the features of the alkyltriphenylphosphonium group as a specific pseudoheteroternary CB[8] motif. Consequently, the designed substrates **7** ^+^ and **8** ^+^ were found capable of producing 1:1 complexes with the macrocycle and, crucially, not with smaller congeners of the cucurbit­[n]­uril family (i.e., CB[7] and CB[6]).

While the interest in the development of new high-affinity guests for the CB[8] host is clear, due to their practical applicability,^2,3^ the study of minimalistic substrates capable of specifically binding one among similar receptors has deeper implications. As such, the corresponding host–guest systems would serve as valuable toy models for a better understanding of subtler aspects of biomolecular recognition associated with substrate specificity,^12^ critical bottlenecks, for instance, in the development of antibody-inspired chemical sensing^13^ or the pharmacodynamic optimization of drug candidates.^14^

## 2 Results and Discussion

### 2.1 Reproducibility of the Pseudoheteroternary Binding Motif: Structural Characterization of Host–Guest Complexes

Salts **2**–**3** ^2+^/**4** ^3+^/**5** ^2+^ included in **set 1** were designed to keep all the structural features of **1** ^2+^ prone to replicate the peculiar pseudoheteroternary complexation mode (i.e., a hydrophobic xylyl core flanked by the positively charged pyridinium/phosphonium groups). As with all the other salts reported in this work, those from **set 1** were synthesized with moderate-to-good yields, reaching as high as 92% in some cases, by bimolecular nucleophilic substitutions, using commercially available pyridines or phosphines as nucleophiles and alkyl or benzylic halides as electrophiles.^15^ The obtained compounds were fully characterized by means of ESI-MS and 1D/2D NMR techniques (COSY, HSQC, and HMBC experiments), paying special attention to the full assignment of the ^1^H and ^13^C nuclei in D_2_O, in order to prospectively determine the complexation-induced shifts (CISs) of the subsequent CB[8]-based complexes.^16^

Consequently, we proceeded to study the association between salts in **set 1** and CB[8] by recording the ^1^H NMR of 2 mM solutions of the corresponding guest in D_2_O containing either 0.5 or 1 equiv of host. In all cases but for the seemingly noninteracting salt **5** ^2+^, these experiments allowed corroboration of not only the formation of the expected complexes but also their 1:1 stoichiometry. Due to the slow exchange observed for the processes on the NMR time scale, separate signals for free and bound guests appear in each case in the spectra with 0.5 equiv of CB[8] (e.g., in Figure 2 for **2** ^2+^⊂CB­[8]), allowing the direct determination of the 1:1 host–guest ratio by integration.^17^ Furthermore, the full assignment of the ^1^H nuclei on the complexes could be conveniently attained with the aid of 2D NMR experiments, allowing for the estimation of the CISs for the interacting species upon complex formation and the extraction of valuable structural information on the binding mode of the phosphonium substrates within the receptor.

**Figure 2:**
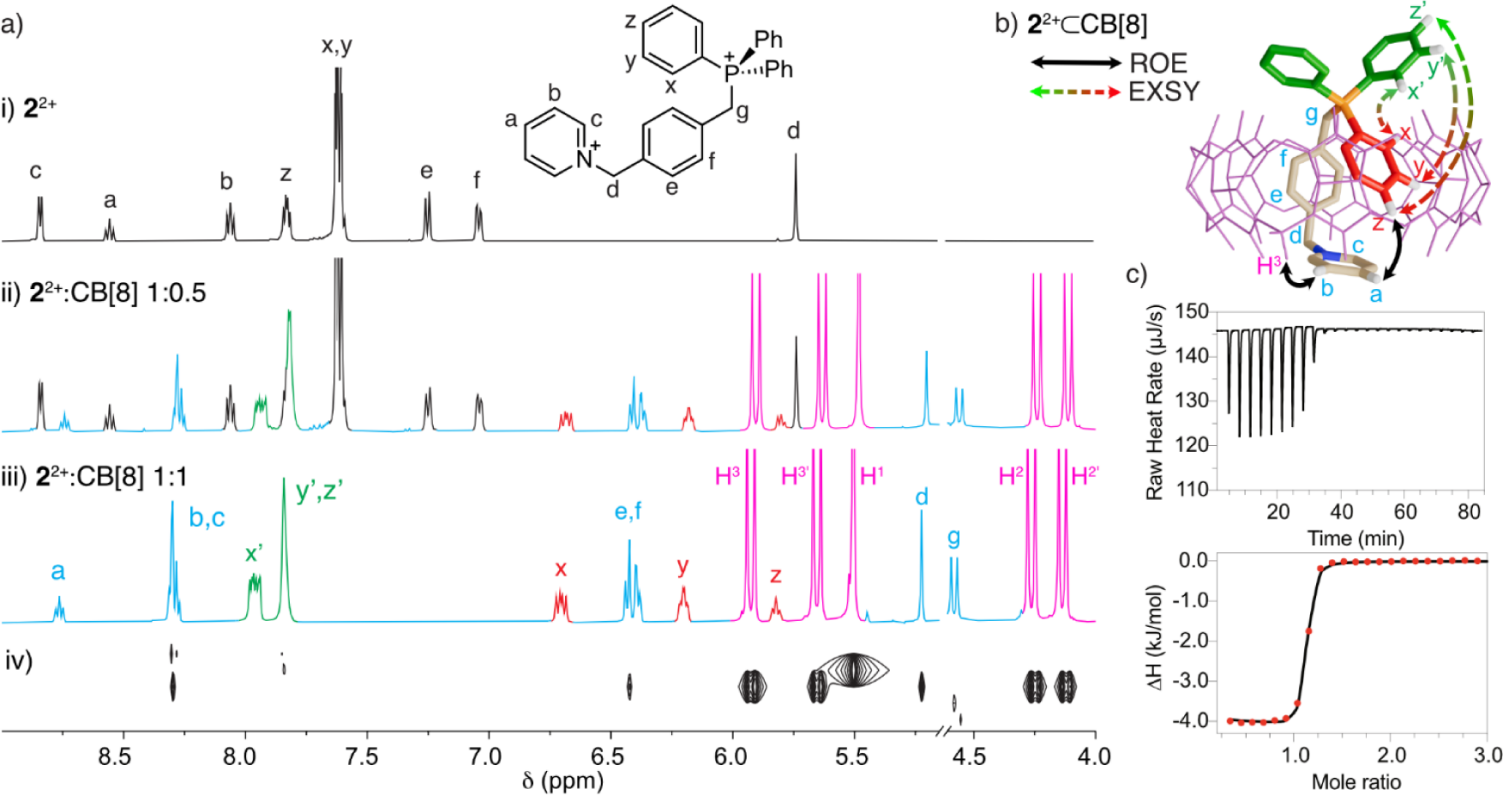
Pseudoheteroternary complexation between **2** ^2+^ and CB[8]. (a) Partial ^1^H NMR (500 MHz, D_2_O) spectrum for: (i) 2 mM solution of **2** ^2+^, (ii) 2 mM of **2** ^2+^ + 0.5 equiv of CB[8], (iii) 2 mM of **2** ^2+^ + 1 equiv of CB[8], and (iv) DOSY experiment for sample (iii). (b) Dashed arrow depiction of the key ROE and EXSY correlations on the representative minima at the r^2^SCAN-3c/CPCM­(water) level for **2** ^2+^⊂CB­[8]. (c) ITC titration data and fitting for **2** ^2+^ + CB[8] ⇌ **2** ^2+^⊂CB­[8].

First, regarding the signals for the host on the ^1^H NMR of **2**–**3** ^2+^/**4** ^3+^⊂CB­[8], a clear loss of symmetry is observed in the spectra compared with that of free CB[8], producing the splitting of the resonances of protons H_2,3_ into two sets of doublets, as a result of the different chemical environments on the otherwise identical carbonyl-laced portals. Regarding the CISs for the guests (as exemplified in Figure 2 for **2** ^2+^⊂CB­[8]), those show in all cases considerable shielding of the nuclei assigned to the xylyl group (H_d‑g_), pointing out the positioning of this moiety deep within the hydrophobic cavity of the receptor. Conversely, protons assigned to the pyridinium-containing moiety (H_a–c_), are more erratically affected by the complexation, with shifts in agreement with a placement of the heterocyclic ring closer to the deshielding region on the CB[8] portals. Finally, the same effect is observed on the ^31^P CISs, with the resonances for these nuclei in the complexes appearing downfield shifted due to the presumed location of the P^+^ atoms above the carbonyl groups (Figure S74). Furthermore, the anticipated pseudoheteroternary binding mode could be clearly assigned for **2**–**3** ^2+^/**4** ^3+^⊂CB­[8] on the basis of the NMR data, due to the unusual pattern of resonances observed for the RP^+^Ph_3_ group, and imposed by the idealized *C*_*s*_ symmetry of the complexed guests. In essence, as shown in Figure 2 for **2** ^2+^⊂CB­[8], two different sets of signals appear in all cases for the three phenyl rings linked to the P^+^ atom, with a group of resonances for ten protons being slightly affected by the complexation and a second set for five protons being strongly shielded, undoubtedly as a result of their inclusion within the cavity of the macrocycle. Importantly, the splitting and shielding of those signals in the RP^+^Ph_3_ moiety is consistent with a situation of slow exchange of those groups on the NMR time scale, as corroborated for **2** ^2+^⊂CB­[8] by the acquisition of DOSY and ROESY experiments. As shown in Figure 2a, the DOSY NMR for the species exhibits a sole diffusion coefficient for the signals of interacting host and guest, while the ROESY clearly correlates by EXSY cross peaks the exchange of the two observed sets of aromatic protons directly attached to the P^+^ atom (Figure S71). As a final point, accounting for the lack of conformational freedom of the guests on the pseudoheteroternary complexes, the ROESY experiment also shows clear ROE correlations for **2** ^2+^⊂CB­[8], being especially relevant to that connecting H_*z*_ and H_a_ of the guest, reflecting a preferred folded *syn*-conformation of the inserted Ph and pyridinium rings regarding the xylyl moiety (Figure 2b).

Following the very same protocol explained for CB[8], the complexation between salts in **set 1** and CB[7] was studied by NMR.^15^ In this case, including **5** ^2+^, we found all the cations interacting with the macrocycle under rapid exchange on the time scale of the technique, with, as previously reported,^11^ the corresponding guest partially inserting the pyridyl-based moiety into the cavity of the host to form the corresponding binary complex.

Finally, the structural characterization of **2**–**3** ^2+^/**4** ^3+^⊂CB­[8] was completed by means of mass spectrometry, showing in all cases HR-ESI-MS peaks corresponding to the complexes.

### 2.2 Thermodynamic Features of Pseudoheteroternary Complexation

The thermodynamics of the complexation reactions between guests in **set 1** and CB[8]/CB[7] were investigated by calorimetric titrations under isothermal conditions at 25 °C, using buffered solutions at pH = 7.^18^

For CB[8], all guests but **5** ^2+^ showed ITC data in good agreement with exergonic processes that could be appropriately fitted to 1:1 models (e.g., in Figure 2c, for **2** ^2+^⊂CB­[8]), certifying the formation of the pseudoheteroternary complexes and their stoichiometry. As shown in Table 1, the obtained values for the free energy of association Δ*G*°_exp_, and consequently for *K*_*a*_, are considerably high for **2**–**3** ^2+^/**4** ^3+^⊂CB­[8] (in the order of 10^7^ M^–1^) but, intriguingly, 3 orders of magnitude lower than the value previously reported for **1** ^2+^⊂CB­[8].^11^ In line with the results obtained by NMR spectroscopy, salt **5** ^2+^ was found not to bind CB[8] to a measurable extent under the experimental conditions, as a likely consequence of the larger steric hindrance over the P^+^ atom.

**Table 1:** Experimental and Calculated Thermodynamic Quantities at 298.15 K for the Complexes Discussed in This Work

Complex	K_a_ (exp)^a^/M^–^^1^	*ΔG*°_exp_ ^b^/kcal·mol^–1^	ΔG°_DFT_ ^c^/kcal·mol^–1^
**1**^2+^⊂CB[8][Bibr ref11]	(3.6 ± 0.7) ×10^10^	–14.5	–18.6
**2**^2+^⊂CB[8]	(1.2 ± 0.7) ×10^7^	–9.6 ± 0.4	–16.2
**3**H^2+^⊂CB[8]	(2.4 ± 0.3) ×10^7^	–10.1 ± 0.1	–14.9
**3**^**+**^⊂CB[8]			–13.8
**4**^3+^⊂CB[8]	(6.5 ± 1.0) ×10^6^	–9.3 ± 0.1	–18.1
**5**^2+^·CB[8]	--^d^		
**6**^**+**^·CB[8]	--^d^		
**7**^**+**^⊂CB[8]	(2.2 ± 0.4) ×10^5^	–7.3 ± 0.1	–6.4
**8**^**+**^⊂CB[8]	(7.4 ± 2.3) × 10^4^	–6.6 ± 0.2	–9.4

a *K*_*a*_ is the average value calculated from triplicate measurements, and the error the standard deviation.

b Δ*G*°_exp_ = −*RT*Ln*K*_a_, calculated in kcal mol^–1^.

c Δ*G*°_DFT_ computed using the multilevel protocol discussed in the text.

d No binding observed.

In the case of CB[7], we were successful in obtaining association constants for two representative cations in **set 1**, namely **2** ^2+^ (owning a pyridinium interaction site, *K*_*a*_ = (3.3 ± 1.4) × 10^4^ M^–1^) and **4** ^3+^ (having a bipyridinium secondary binding moiety, *K*_*a*_ = (2.7 ± 2.4) × 10^6^ M^–1^). The obtained values for the corresponding 1:1 binary complexes are not only in good agreement with previously reported data for the interaction of this type of binding motif with CB[7] ^11,19^ but also support our finding of no specificity of models in **set 1** for CB[7]/CB[8] based on the previously discussed NMR results.

With the aim of explaining the differences observed in binding affinity, we modeled the potential 1:1 complexation equilibria between guests in **set 1** and CB[8], using state-of-the-art methods for the generation of representative lowest-lying structures for the species via force field and semiempirical methods.^20–24^ Strikingly, in all cases but for **5** ^2+^, the recently developed aISS docking workflow^24^ produced, as preferred poses for **2**-**3** ^2+^/**4** ^3+^·CB­[8] at the GFN2-xTB^21^/ALPB^22^(water) level, structures in good agreement with the experimentally observed pseudoheteroternary *syn*-folded inclusion complexes **2**-**3** ^2+^/**4** ^3+^⊂CB­[8] (e.g., **2** ^2+^⊂CB­[8] in Figure 2b). Subsequent reoptimization of the representative geometries was tackled by the highly efficient and computationally affordable, r^2^SCAN-3c composite DFT-D method,^25^ including solvation effects in water by means of the CPCM scheme.^26^ As shown in Table 1, the corresponding free energies of association Δ*G*°_DFT_ for the equilibria at the r^2^SCAN-3c/CPCM­(water) level^27^ produced values that not only qualitatively reflect the observed experimental trend of highly favored processes but also show a large overestimation of the binding.^28,29^

In the particular case of **1** ^2+^⊂CB­[8], inspection of its representative minimum partly explains the anomalously high value of the calculated and measured Δ*G*° as that can be ascribed to the presence of various stabilizing hydrogen bonding interactions between the hydrated aldehyde on the guest and the carbonyl oxygen nuclei on the host (Figure S156). For the noncomplexing guest **5** ^2+^, a positive value of Δ*G*°_DFT_ was obtained for its complexation by CB[8] (Table 1), resulting from the best pose obtained through the docking protocol, which shows the sole partial inclusion of the pyridinium subunit within the cavity of the host. The DFT-D calculations also qualitatively explain the intuitively anomalous value of the association constant measured for isonicotinic acid derivative **3** ^+^/**3**H^2+^. Although the complete deprotonation of the salt can be safely assumed on the ITC experiments at pH = 7,^30,31^ NMR experiments recorded for the complex at pD = 3 and 12 do not differ significantly from those in D_2_O (Figures S81 and S82), suggesting a negligible influence of the potential carboxylate-carbonyl repulsions on the binding. Conversely, the ionization of the carboxylic acid crucially affects the affinity of the salt for CB[7], as demonstrated by the ^1^H NMR spectra of an equimolar mixture of substrate and receptor at pD = 12, which exclusively shows the resonances of unreacted components (Figure S126). These experimental observations are supported by DFT-D calculations, which showed almost no differences in the geometries and Δ*G*°_DFT_ obtained for the pseudoheteroternary **3** ^+^/**3**H^2+^⊂CB­[8] complexes, but huge dissimilarities in the behavior with CB[7], which is only able to produce a slightly exergonic inclusion complex with the protonated form **3**H^2+^ (Figure 3).

**Figure 3:**
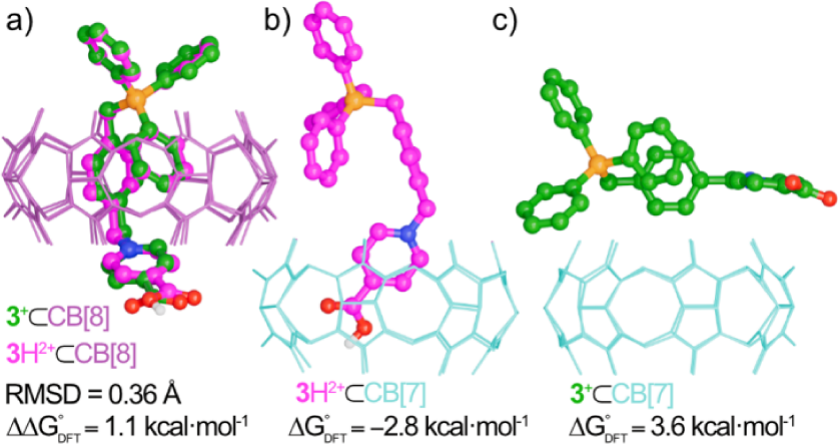
Structures of the representative minima at the r^2^SCAN-3c/CPCM­(water) level for complexes between **3** ^+^/**3**H^2+^ and CB­[7/8]. (a) Overlay of **3** ^+^/**3**H^2+^⊂CB­[8] (RMSD: root-mean-square deviation of atomic positions, computed with the software CREST).^22^ (b) **3**H^2+^⊂CB­[7]. (c) **3** ^+^⊂CB­[7].

### 2.3 Dynamic Features of Pseudoheteroternary Complexation

Aiming to extract more information about the intricate dynamics of the complexation processes, we decided to take advantage of the slow regime observed by NMR for the exchange equilibria associated with the simpler species in **set 1**: **2** ^2+^⊂CB­[8]. First, VT-NMR experiments were conducted in the 298.15–368.15 K window for a sample containing equimolar amounts of host and guest (Figure S78). This experiment clearly shows how the exchange between the phenyl groups on RP^+^Ph_3_ becomes increasingly affected as the temperature rises, reaching a situation of almost complete coalescence at 368.15 K. However, no significant changes were observed in the signals for the pyridinium and xylyl moieties when the temperature increases, with chemical shifts coinciding with those for the bound guest. This fact qualitatively certifies that the slow regime persists for the overall self-assembly process even at the highest temperature recorded, being consequently slower if compared to the rate for the phenyl exchange.

To clarify and quantify the kinetics of the two different equilibria inferred from the NMR data of **2** ^2+^⊂CB­[8], we obtained the exchange rate constants (*k*_ex_) at room temperature using the selective inversion recovery (SIR) method. This method was previously described for similar host–guest systems, where different concurrent slow exchange processes were observed.^32^ Consequently, spectra for a mixture of host and guest with a 100% excess of **2** ^2+^ were acquired, selecting the characteristic signals of each observed process for the SIR experiments (Figure 4). Hence, for the guest exchange, we chose to selectively invert one of the two doublets corresponding to the xylyl moiety on the noncomplexed cation (H_f,free_, δ = 6.94 ppm), observing the effect of the magnetization transfer at different delay times for the equivalent nuclei on the bound substrate (H_f,bound_, δ = 6.26 ppm). In this case, fitting of the obtained data to an appropriate kinetic model resulted in *k*_ex,guest_ = 0.32 ± 0.09 s^–1^. Conversely, the same protocol applied to the equivalent protons on the outside (H_*x*_,δ = 7.85 ppm) and inside (H_x’_,δ = 6.60 ppm) of the exchanging phenyl units in **2** ^2+^⊂CB­[8] allowed us to derive *k*_ex,Ph_ = 6.78 ± 0.44 s^–1^, confirming the experimental observation of this process being substantially faster than the self-assembly. Furthermore, by considering both equilibria consisting of two unimolecular elementary reactions and using the corresponding eqs S1–S5, the kinetic parameters (*k*_*in*_ and *k*_*out*_) of the guest exchange and phenyl exchange were determined (Table S33).

**Figure 4:**
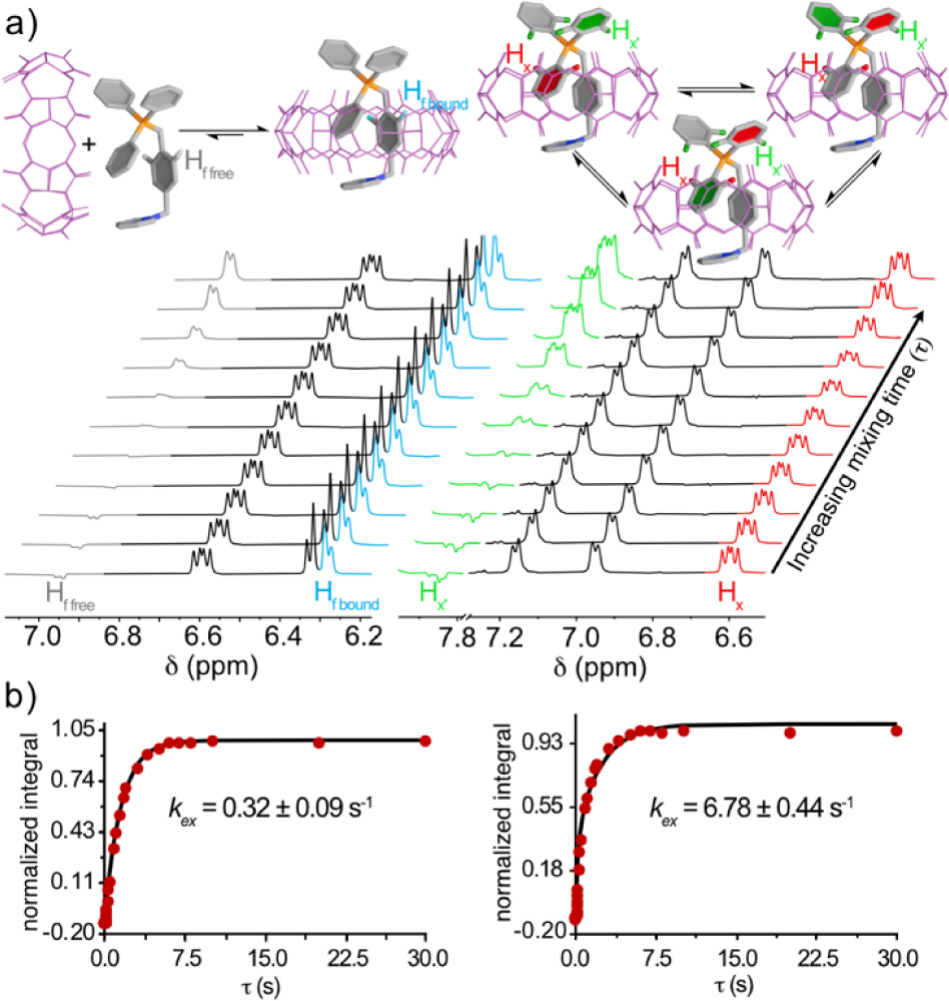
(a) Partial ^1^H NMR (400 MHz, D_2_O, 298.15 K) at different mixing times (τ), obtained from the SIR experiments for **2** ^2+^⊂CB­[8] involving exchanging nuclei: H_f_ on the xylyl group (left), H_*x*_ on the phenyl moiety (right). (b) Experimental normalized integration vs time data (red dots) and least-squared minimization of the predicted values according to the McClung’s formulation (solid line), used for the extraction of the exchange rate constants *k*_ex,guest_ (left) and *k*_ex,Ph_ (right) *via* the CIFIT software.^33^

Intrigued by the observed exchange dynamics, we proceeded to explore it *in silico* at the computationally affordable GFN2-xTB/ALPB­(H_2_O) level of theory.^21,22,34^ First, in order to tackle a potential binary dissociative mechanism for the ingression/egression of the guest, we considered as a starting point the previously optimized representative minima for **2** ^2+^⊂CB­[8],^20^ and the two possible options for the complete attachment/detachment of the guest: arbitrarily to the right or left relative to the center of mass of the complex and along the C_8_ rotational axis of the host.^15^ In this situation, we explored the potential energy surface connecting the noninteracting host and guest with the minima, by using the well-known nudged elastic band (NEB) methodology.^35^ This approach led us to two different mechanistic proposals, which have in common rate-determining steps associated with the disruption of the favorable cation-dipole interactions and the subsequent displacement of either the charged N^+^ or P^+^-containing moieties of the guest through the hydrophobic cavity of the receptor. As intuitively expected, the path involving the translation of the smaller pyridinium moiety was favored by *ΔΔ*G^‡^ = 5.7 kcal/mol (path A, Figure 5), compared to that comprising the RP^+^Ph_3_ group (path B, Figure S158). As shown, the proposed mechanism through path A implies the barrierless formation of a stable preassociation intermediate (**I**_**1**_), in which the pyridinium and one of the phenyl rings attached to the P^+^ are partially inserted into the cavity of CB[8]. From that point, both the xylyl and phenyl rings can be pushed to the observed pseudoheteroternary binding mode following a highly exergonic step.

**Figure 5:**
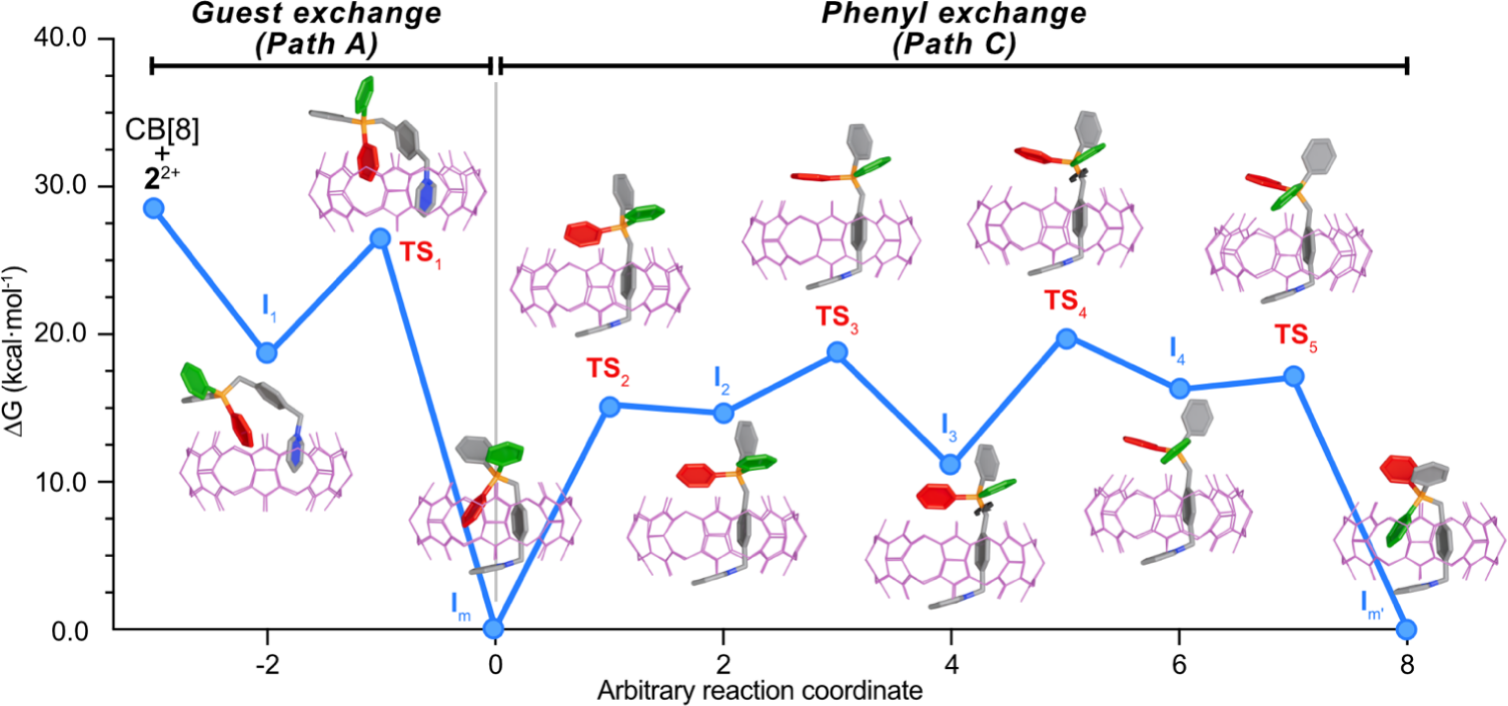
Normalized free energy surface at the GFN2-xTB/alpb­(H_2_O) level, showing the structures of intermediates and transition states in the tentative mechanism proposed for the guest (path A) and Ph exchange (path C) in **2** ^+^ + CB[8] ⇌ **2** ^+^⊂CB­[8].

Regarding the mechanism for the exchange between Ph/Ph’ on RP^+^Ph_2_Ph’, and to justify the experimentally observed different rate of the process compared to the guest exchange, we explored an alternative to that of paths A/B by envisioning an idealized 120° rotation along the RCH_2_–P^+^Ph_3_ bond without dissociation of the guest. This approach led us to the multistep mechanism shown in Figure 5 (path C), consisting of the initial highly endergonic expulsion of the inserted P^+^Ph group from the cavity of CB[8] through the transition state **TS**_**2**_, inversion of the planar chirality over the P^+^ atom (**TS**_**3**_), rotation along the RCH_2_–P^+^Ph_3_ bond (**TS**_**4**_), and a final exergonic reinsertion of the nonequivalent P-Ph’ group within the macrocycle (TS_5_). Despite being energetically quite costly, this alternative is faster than the guest exchange through path A by *ΔΔ*G^‡^(**TS**_4_-**TS**_1_) = 11.4 kcal/mol at this level of theory, being qualitatively in good agreement with the observed experimental data.

### 2.4 Deciphering the Structural Requisites for the Pseudoheteroternary Specific Binding to CB[8]

The results obtained thus far indicate that the substitution on the P^+^ atom controls the ability of the studied salts to be complexed by CB[8], primarily through a remarkable synergistic effect (Figure 6). Despite all guests in **set 1** having three available fragments that contribute to the overall stability of the pseudoheteroternary complexes (i.e., the phenyl, xylyl, and pyridinium-containing moieties), the relative energies of the reaction intermediates in the complexation mechanism proposed for **2** ^2+^(Figure 5), and the results discussed for the noncomplexing salt **5** ^2+^, emphasize how none of them are individually capable of producing significant binding with CB[8]. Hence, while the inclusion of any of the individual aromatic binding units within the host is precluded by steric repulsions, the pseudoheteroternary binding mode allows both to diminish this hindrance and to increase the occupied volume within the host. In turn, this conformation allows the pyridinium-like moiety to contribute to the overall binding by an appropriate positioning on the second portal of the macrocycle. Conversely, being not able to form the pseudoheteroternary motif, CB[7] is capable of complexing only the sterically more accessible pyridinium moiety. Consequently, we argued that the removal of the heterocyclic substituents in **set 1** would not only have little impact on the binding mode but would deprive the designed guests of the ability to be complexed by smaller cucurbituril analogues. In order to explore this hypothesis, we intended to study the monocationic salts **6** ^+^-**8** ^**+**^ in **set 2** with the CB­[6/7/8] macrocycles.

**Figure 6:**
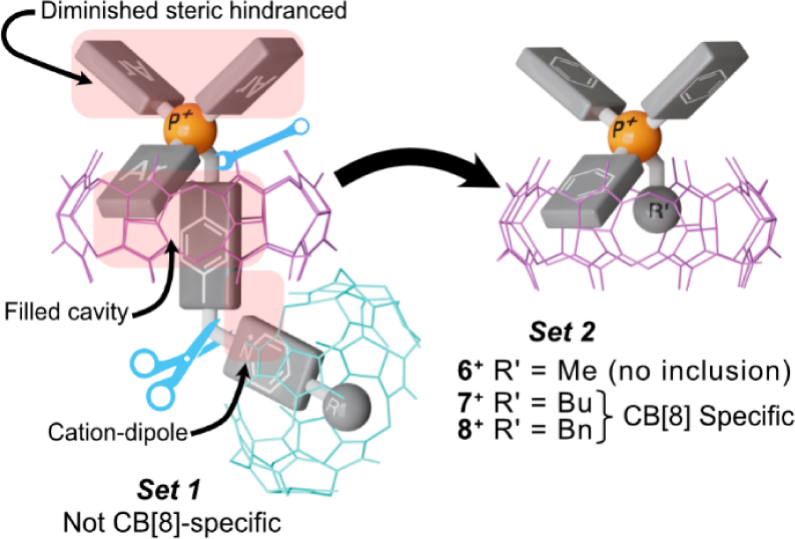
**Set 2** model compounds: improving the alkyltriphenylphosphonium specificity to CB[8] by simplification.

To further corroborate our assumption of the requirement of a synergistic effect by two appropriate substituents over the P^+^ atom to achieve complexation by CB[8], we proceeded to record the ^1^H NMR in D_2_O at room temperature for **6** ^+^ with equimolar concentrations of the macrocycle (Figure S104). The results demonstrated the absence of CISs for the mixed species, in good agreement with the faint value of Δ*G*°_DFT_ estimated for the process (Table 1). Pointing in this very direction, the same ^1^H NMR experiments for cation **7** ^**+**^ showed indisputable signs of complexation by CB[8], in this case under a rapid but near-coalescence exchange regime on the NMR time scale at room temperature, as certified by the appearance of the same averaged signals on the corresponding experiment with a defect of host (Figure 7). VT ^1^H NMR experiments produced in this case sharper resonances upon increasing the temperature, allowing us to unambiguously assign the resonances of the interacting species, with the help of the ^31^P-induced multiplicities in the case of the butyl group. Although more subtly than for salts in **set 1**, the CISs for the interacting species point out the formation of the **7** ^+^⊂CB­[8] pseudoheteroternary complex (Figure 7). In essence, the positioning of the butyl group deep within the cavity of the receptor can be easily inferred, based on the shielding of the resonances of the alkyl group upon complexation. Regarding the expected inclusion of one of the phenyl moieties within CB[8], although harder to assess in this case due to the rapid exchange on the NMR time scale of the three equivalent groups, it can be implied by the slim but noticeable shielding of their resonances.

**Figure 7:**
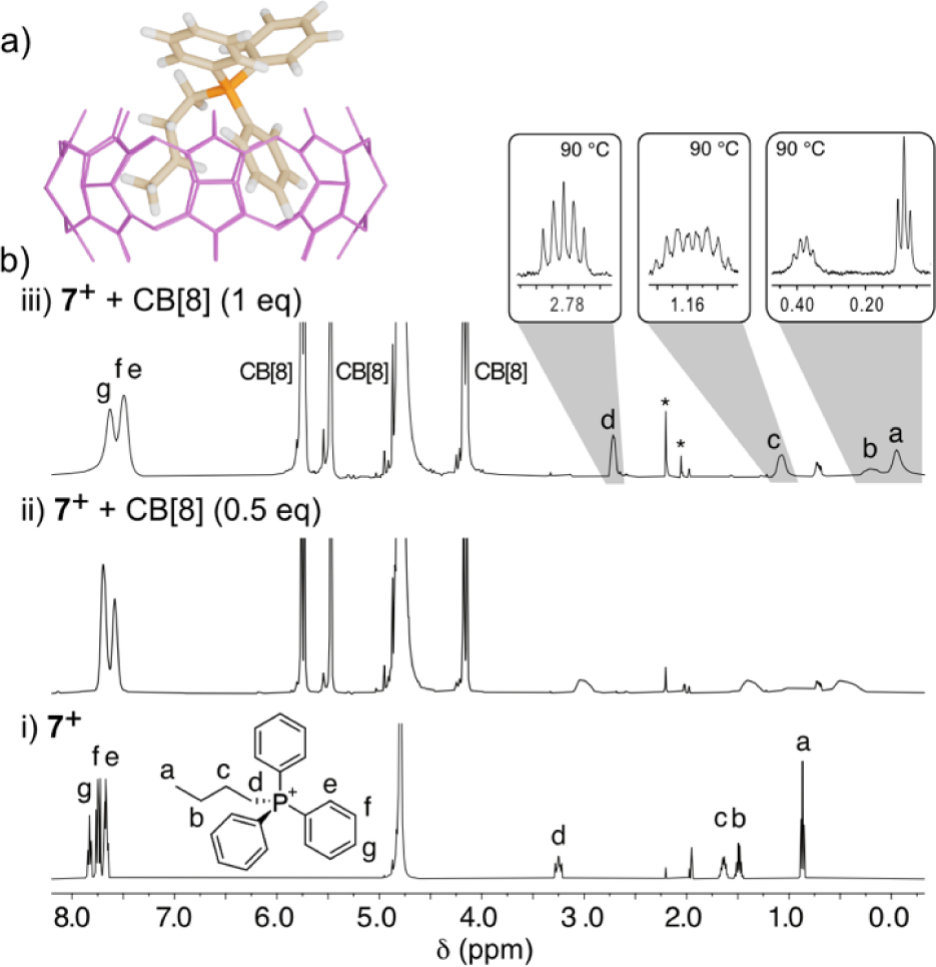
(a) Representative minima at the r^2^SCAN-3c/CPCM (water) level for **7** ^+^⊂CB­[8]. (b) Partial ^1^H NMR (500 MHz, D_2_O, 298 K) spectra for 1 mM solutions of (i) **7** ^+^, (ii) **7** ^+^ + 0.5 equiv of CB[8], and (iii) **7** ^+^ + 1 equiv of CB[8] (with insets showing the resolved multiplicities for the sample at 363 K). Impurities are marked with *: CH_3_CN (1.98 ppm, s) and acetone (2.22 ppm, s).

A more familiar situation was found for **8** ^+^, which showed a near coalescence situation at room temperature on its interaction with the macrocycle (Figure S111), this time being closer to the slow-exchange situation discussed for salts in **set 1**. In particular, on the equimolar mixture of the salt and CB[8], the signals observed at room temperature appear significantly broadened, a fact that hinders the assignment of the CISs, and that was not significantly improved by increasing the temperature in VT ^1^H NMR experiments (Figure S113). Fortunately, those could be recorded as well in the 298.15–278.15 K range by using a 1:2 molar mixture of host and guest in the presence of 1 M NaCl. As shown in Figure 8a, the ^1^H NMR at 278 K in those conditions clearly shows a slow-exchange regime with separate well-resolved signals for the exchanging Ph groups and guest, which was corroborated as well in the corresponding ^1^P NMR experiment even at room temperature (Figure S114).

**Figure 8:**
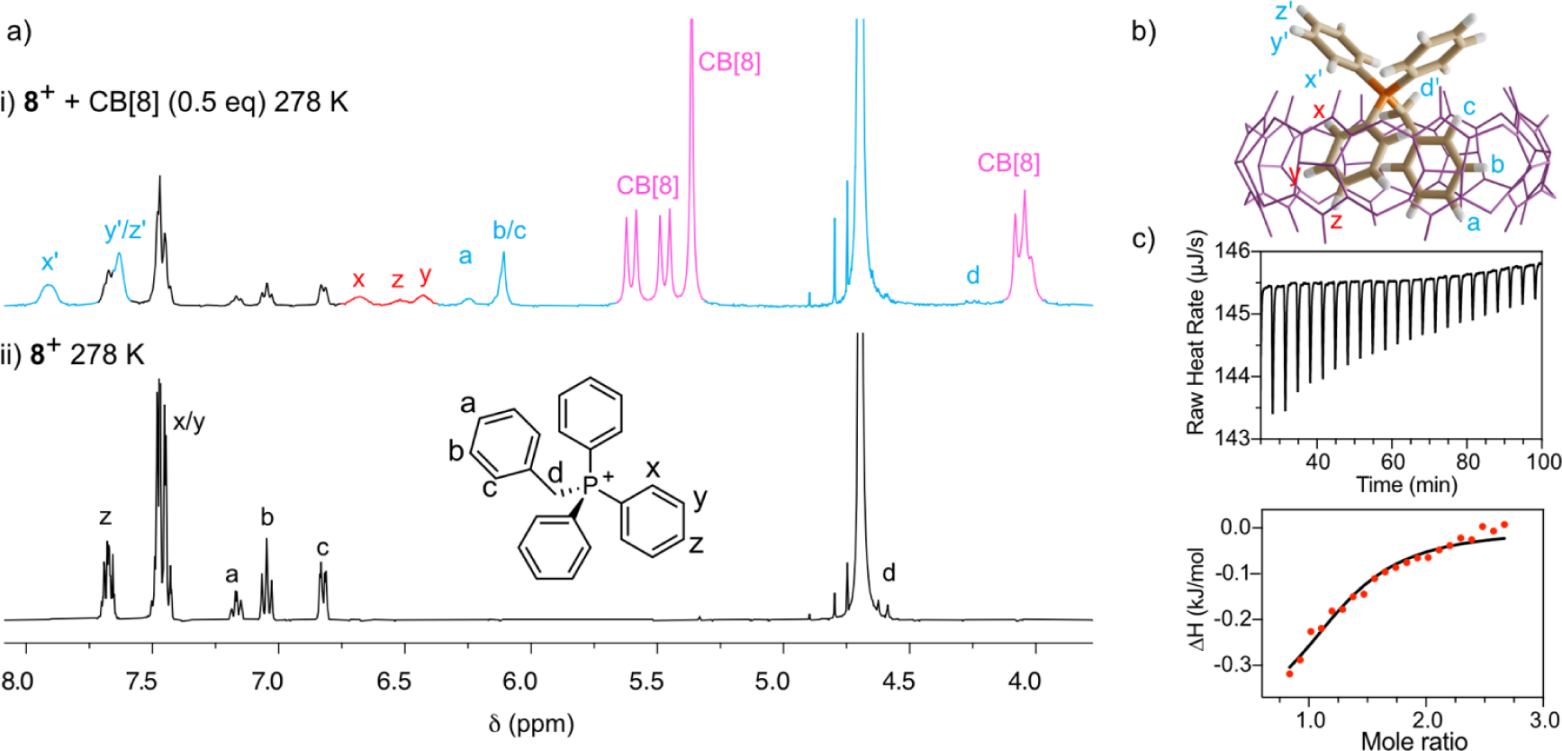
Pseudoheteroternary complexation between **8** ^+^ and CB[8]. (a) Partial ^1^H NMR (500 MHz, D_2_O) spectrum (containing 1 M NaCl), for 2 mM solutions of (i) **8** ^+^ + 0.5 equiv of CB[8] at 278 K and (ii) **8** ^+^ at 278 K. (b) Representative minima at the r^2^SCAN-3c/CPCM­(water) level for **8** ^+^⊂CB­[8]. (c) ITC titration data and fitting for **8** ^+^ + CB[8] ⇌ **8** ^+^⊂CB­[8].

As for salts in **set 1**, ITC experiments were recorded for the complexation processes with CB[8], yielding values for the association constants in the 10^5^ M^–1^ range for **7** ^+^ **/8** ^+^, which although lower than for salts in **set 1** due to the lack of a second charged moiety, implicitly corroborate the formation of pseudoheteroternary 1:1 complexes (Table 1 and Figure 8 for **8** ^+^⊂CB­[8]). Furthermore, computation of the free energies of association Δ*G*°_DFT_ for the pseudoheteroternary inclusion complexes with salts in **set 2** resulted in values in good agreement with the observed experimental data at the r^2^SCAN-3c/CPCM­(water) level of theory. Additionally, application of the same computational protocol discussed for the study of the dynamics of **2** ^2+^⊂CB­[8] to complex **7** ^+^⊂CB­[8], produced as well, results in good qualitative agreement with the swapping of the observed NMR exchange to a faster regime. In this case, due to the absence of the second charged group, the energy barrier leading to the pseudoheteroternary pose in **7** ^+^⊂CB­[8] is significantly lowered compared with that in **2** ^2+^ as the former would not involve, in this case, the unfavorable translation of a positively charged fragment across the host cavity (Figure S159).

Finally, regarding the potential complexation of salts in **set 2** with smaller analogues of CB[8] (i.p. CB[6] and CB[7]), those showed no signs of interaction with these macrocycles by NMR (Figures S134–S139), pointing out a remarkable specificity of **7** ^+^/**8** ^+^ for CB[8] despite the availability of appropriate hydrophobic groups attached to the overcrowded positively charged atom.

## 3 Conclusions

We have reported herein an in-depth study on the complexation capabilities of two series of the easily accessible and highly modifiable alkyltriphenylphosphonium class of organic salts, capable of forming pseudoheteroternary 1:1 inclusion complexes with the popular CB[8] host. Different from other pair-inclusion motifs for this type of binding that also substantially complex CB[7] (see examples in Figure 1),^6,7^ our salts produce this type of association on the basis of a synergistic effect provoked by the substitution on the sterically hindered P^+^ atom. Hence, despite having three different appropriate aromatic binding sites available for their complexation by CB­[7/8] (i.e., phenyl, xylyl, and pyridinium groups), inclusion of the guests on **set 1** within CB[8] is only produced when the xylyl and one of the phenyl moieties can be simultaneously inserted, a fact that promotes both a better occupation of the host and a clear decrease in the steric repulsions between the overcrowded P^+^ atom and the carbonyl rims of the macrocycle. Conversely, the inability of the smaller analogue CB[7] to accommodate two guests within its cavity prevents the formation of the pseudoheteroternary motif, favoring only the complexation of the more accessible pyridinium-containing moiety. Consequently, by removing the CB[7]-recognition site on the phosphonium guest, we designed and studied a second set of substrates capable of reproducing the synergy-promoted pseudoheteroternary complexation and being at the same time specific binders of CB[8] over smaller analogues CB[6] and CB[7]. Overall, our results nicely exemplify how the conjunction of currently available experimental and electronic structure theoretical techniques can enable, not only the detailed study of fairly complex host–guest systems of highly current practical interest^2,3^ but also shed some light on the subtleties associated with long-standing issues related to substrate specificity in molecular recognition.^12–14^

## Supplementary Material





## Data Availability

The data underlying this study are available in the published article and its Supporting Information.

## Notes

Experimental details, synthetic procedures and characterization data for new compounds, characterization data for inclusion complexes, ITC titration data for the determination of the association constants (*K*_a (exp)_), SIR kinetics experiments, and computational details (PDF)
Cartesian coordinates in xyz format for the species (ZIP)
