# Supplementary material for: Synergy-Promoted Specific Alkyltriphenylphosphonium Binding to CB[8]
Source: J Org Chem. 2025 Feb 10;90(12):4149–57. doi: 10.1021/acs.joc.4c02546 (PMC12123668; doi:10.1021/acs.joc.4c02546)
Supplement: Supplementary file 1 [file jo4c02546_si_001.pdf]

Supporting Information

Synergy-promoted Specific Alkyltriphenylphosphonium Binding to CB[8]

Mauro Díaz-Abellás, Iago Neira, Arturo Blanco-Gómez, Carlos Peinador* and Marcos D. García*

Departamento de Química and Centro Interdisciplinar de Química y Biología (CICA),

Facultad de Ciencias, Universidade da Coruña, 15071, A Coruña, Spain.

Email (corresponding authors): carlos.peinador@udc.es; marcos.garcia1@udc.es

Table of Contents:

1. GENERAL PROCEDURES S4
2. SYNTHETIC PROCEDURES S5
   2.1. Synthesis and characterization data of precursor P_3·Br S5
   2.2. Synthesis and characterization data of 2·2Br S8
   2.3. Synthesis and characterization data of 3·2TFA S15
   2.4. Synthesis and characterization data of 4·3Cl S21
   2.5. Synthesis and characterization data of 5·2Br S27
   2.6. Synthesis and characterization data of 6·I S33
   2.7. Synthesis and characterization data of 7·Br S38
   2.8. Synthesis and characterization data of 8·Br S44
3. HOST-GUEST CHEMISTRY S50
   3.1. NMR study of the interaction of 2·2Br and CB[8] S50
   3.2. NMR study of the interaction of 3·2TFA and CB[8] S59
   3.3. NMR study of the interaction of 4·3Cl and CB[8] S68
   3.4. NMR study of the interaction of 5·2Br and CB[8] S74
   3.5. NMR study of the interaction between 6·I and CB[8] S76
   3.6. NMR study of the interaction between 7·Br and CB[8] S78
   3.7. NMR study of the interaction between 8·Br and CB[8] S82
   3.8. NMR study of the interaction between 2·2Br and CB[7] S87
   3.9. NMR study of the interaction between 3H·2Br/3·Br and CB[7] S90
   3.10. NMR study of the interaction between 4·3Cl and CB[7] S94
   3.11. NMR study of the interaction between 5·2Br and CB[7] S97
   3.12. NMR study of the interaction between 6·I and CB[7] S99
   3.13. NMR study of the interaction between 7·Br and CB[7] S100
   3.14. NMR study of the interaction between 8·Br and CB[7] S101
   3.15. NMR study of the interaction between 6·I and CB[6] S102
   3.16. NMR study of the interaction between 7·Br and CB[6] S103
   3.17. NMR study of the interaction between 8·Br and CB[6] S104
4. ISOTHERMAL TITRATION CALORIMETRY EXPERIMENTS S105
   4.1. ITC data of complex 2^{2+}□CB[8] S106
   4.2. ITC data of complex 3^{2+}□CB[8] S110
   4.3. ITC data of complex 4^{3+}□CB[8] S114

4.4. ITC data of complex 6^{+}CB[8] S118
4.5. ITC data of complex 7^{+}CB[8] S122
4.6. ITC data of negative controls....................S127
4.7. ITC data of complex 2^{2+}CB[7] S128
5.1. ITC data of complex 4^{3+}CB[7] S133
6. SELECTIVE INVERSION RECOVERY KINETICS EXPERIMENTS S137
7. COMPUTATIONAL DETAILS:....................S148
7.1. Representative minimum structures.....................S148
7.2. Free energy calculations at r^2SCAN3c/CPCM(H_2O) level of theory. S153
7.3. Kinetic profiles for the complexation of guests 2^{2+} and 7^{+} at the GFN-xTB/alpb(H_2O) level of theory… S156

1. GENERAL PROCEDURES

Starting materials were purchased from commercial suppliers and used without further purification. Precursor **P1**·Br[1] and **P2**·PF_6[2] were prepared according to the literature. The purity of CB[7] and CB[8] was assessed as previously reported by Kaifer *et* al.[3] Milli-Q water was purified with a Millipore Gradient A10 apparatus. Merck 60 F254 foils were used for thin layer chromatography, and Merck 60 (230-400 mesh) silica gel was used for flash chromatography. NMR spectra were recorder on a Bruker Advance 400 or 500 MHz for ^{1}H, 125 MHz for ^{13}C equipped each other with a dual cryoprobe and 160 MHz for ^{31}P. The solvents used for NMR experiments were deuterated water (D_2O) and acetonitrile (CD_3CN). Chemical shifts are reported in ppm relative to the residual internal non deuterated solvent signals (D_2O: δ = 4.79 ppm, CD_3CN: δ = 1.94 ppm). Mass spectrometry experiments were carried out in a LCQ-q-TOF Applied Biosystems QSTAR Elite spectrometer for low and high resolution ESI. UV/Vis spectra were recorded on a Jasco V-650 spectrometer. Purifications were carried out in a preparative HPLC Agilent 1260 Infinity II, using an InfinityLab ZORBAX Eclipse Plus column (C18 stationary phase, 21.2 × 250 mm, ø = 5 µm). HPLC-MS analysis were performed using a Thermo Scientific UltiMate 3000 connected to a photo-diode array (PDA) detector using a Luna® Omega analytical column from Phenomenex (Polar-C18 stationary phase, 3 μm, 100 Å pore size, 150 × 2.1 mm). Titration experiments were carried out in 20 mM sodium phosphate buffer (pH = 7.00 or pH=3.00) at 25 ºC on a Nano-ITC calorimeter from TA instruments. Structural assignments were made with additional information from gCOSY, gHSQC, gHMBC, gROESY and gNOESY experiments.

---

[1] Neira, I.; Peinador, C.; García, M. D. CB[7] and CB[8]-based [2]-(pseudo)rotaxanes with triphenylphosphonium-capped threads: Serendipitous discovery of a new high-affinity binding motif. *Org. Lett.* **2022**, *24*, 4491-4495.
[2] Neira, I.; Domarco, O.; Barriada, J. L.; Franchi, P.; Lucarini, M.; Garcia, M. D.; Peinador, C. An electrochemically controlled supramolecular zip tie based on host-guest chemistry of CB[8] *Org. Biomol. Chem.* **2020**, *18*, 5228-5233.
[3] Yi, S.; Kaifer, A. E. Determination of the Purity of Cucurbit[n]Uril (n = 7, 8) Host Samples. *J. Org. Chem.* **2011**, *76* (24), 10275-10278.

2. SYNTHETIC PROCEDURES

2.1. Synthesis and characterization data of precursor P_3·Br

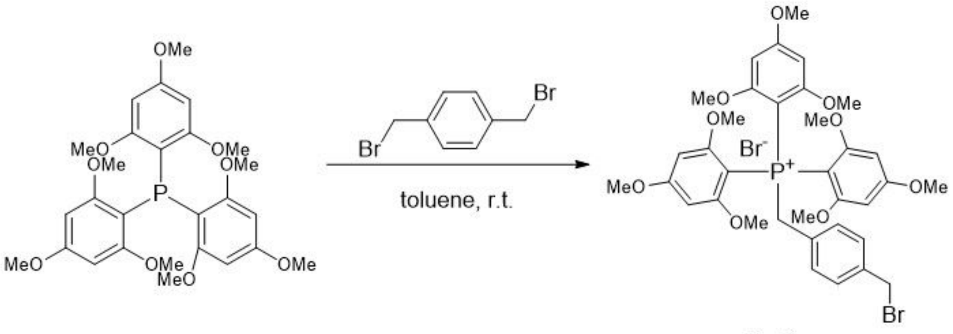

P₃·Br

A mixture of tris(2,4,6-trimethoxyphenyl)phosphine (334 mg, 0.628 mmol, 1 eq) and 1,4-bis(bromomethyl)benzene (500 mg, 1.88 mmol, 3 eq) in 20 mL of toluene was stirred at room temperature for 24 hours. The precipitate was filtered under vacuum, washed with Et_2O (3 × 15 mL) and dried in the vacuum line to leave **P_3**·Br as a white solid (417 mg, 83 %).

^{1}H NMR (400 MHz, D_2O) *δ (ppm)*: 7.09 – 7.00 (m, 4H), 6.18 (dd, *J* = 4.7, 1.9 Hz, 6H), 4.63 (d, *J* = 17.2 Hz, 2H), 4.40 (d, *J* = 1.6 Hz, 2H), 3.78 (s, 9H), 3.55 (s, 18H). **^{13}C{^{1}H} NMR** (126 MHz, D_2O) *δ (ppm)*: 165.20 (d, *J* = 1.8 Hz), 163.4 (d, *J* = 5.2 Hz), 138.6, 133.1 (d, *J* = 6.9 Hz), 129.8 (d, *J* = 8.4 Hz), 126.8 (d, *J* = 2.1 Hz), 92.8 (d, *J* = 105.2 Hz), 91.2 (d, *J* = 7.2 Hz), 63.3, 55.6 (d, *J* = 2.2 Hz), 55.5 (d, *J* = 5.9 Hz). **HRMS (ESI)** m/z: [**P_3**]$^+$ Calcd for $C_{35}H_{41}BrO_9P^+$ 715.1657; Found 715.1667.

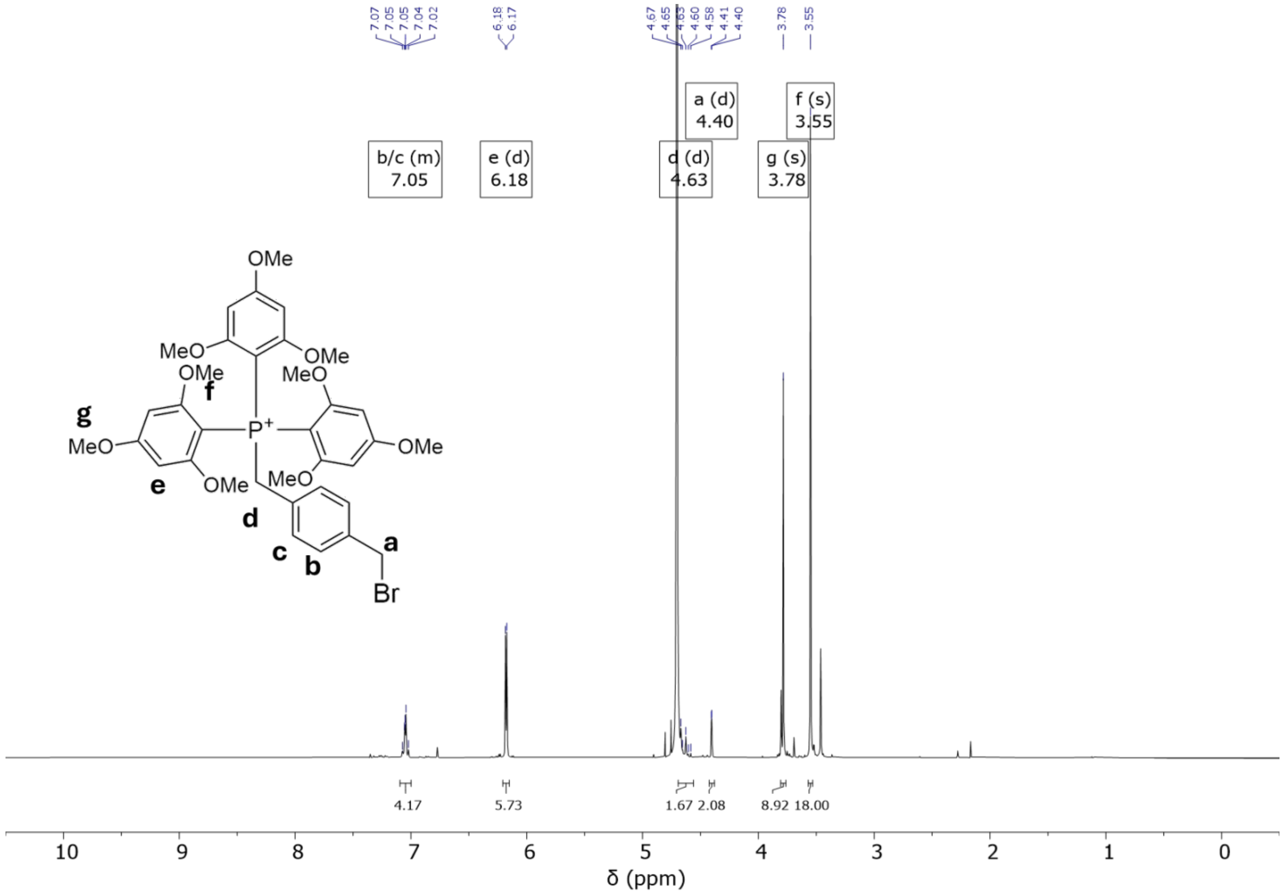

Figure S1: ^{1}H NMR (500 MHz, D_2O) spectrum of **P3**·Br.

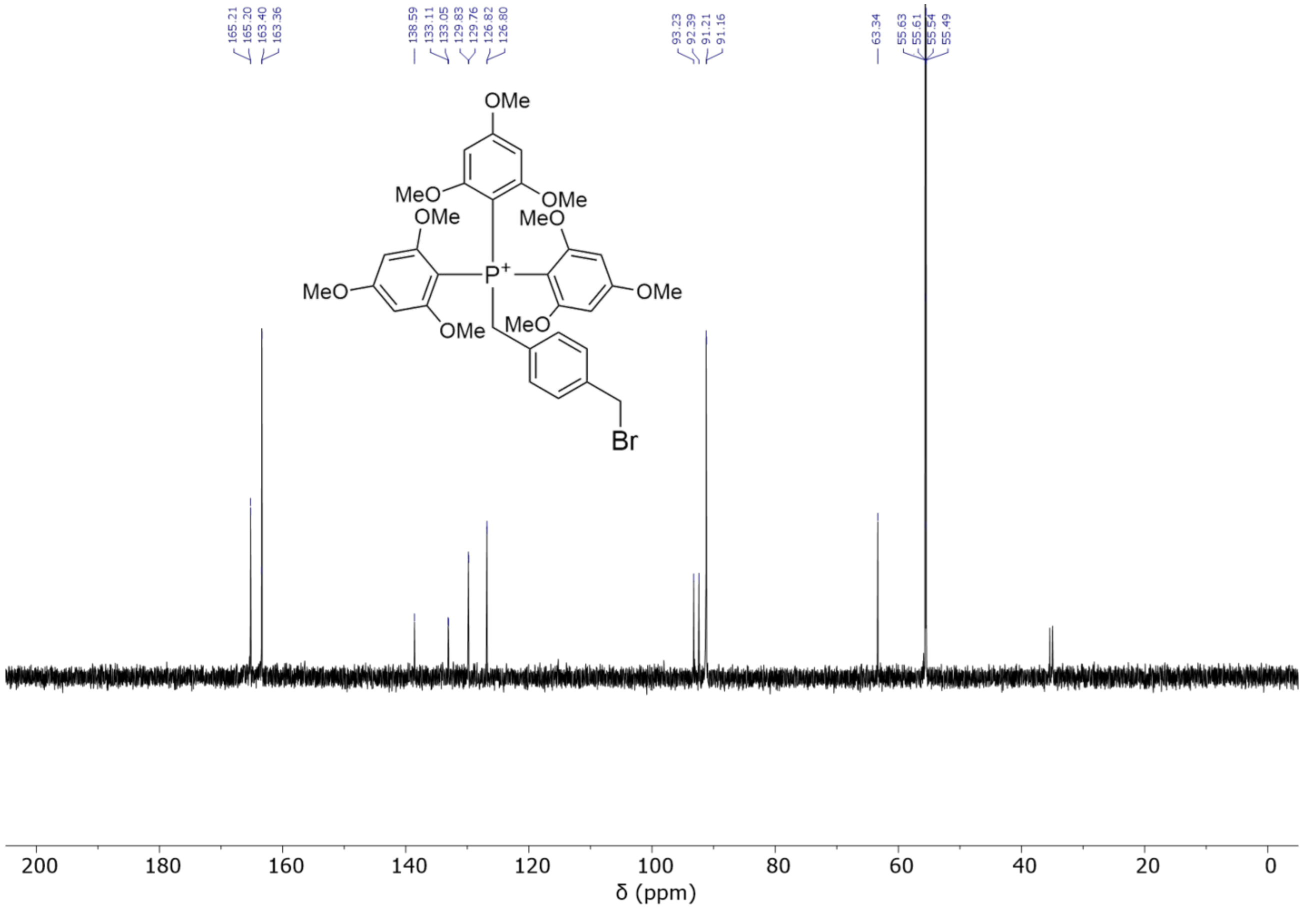

Figure S2: $^{13}C\{^1H\}$ NMR (126 MHz, D_2O) spectrum of **P3**·Br.

2.2. Synthesis and characterization data of 2·2Br

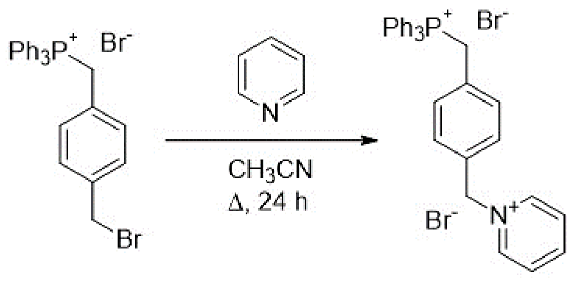

P₁·Br **2**·2Br

A mixture of $\mathbf{P_1}$·Br (435 mg, 0.826 mmol, 1 eq) and pyridine (200 µL, 196 mg, 2.5 mmol, 3 eq) in 25 mL of CH_3CN was stirred under reflux in a hot plate stirrer for 24 hours. The precipitate was filtered under vacuum, washed with CH_3CN (2 × 15 mL) and Et_2O (2 × 15 mL) and dried in the vacuum line to yield the product **2**·2Br as a white solid (260 mg, 52 %).

^{1}H NMR (500 MHz, D_2O) *δ (ppm)*: 8.84 (d, *J* = 6.1 Hz, 2H), 8.56 (t, *J* = 7.9 Hz, 1H), 8.06 (t, *J* = 7.1 Hz, 2H), 7.84 (m, 3H), 7.65 – 7.57 (m, 12H), 7.25 (d, *J* = 7.9 Hz, 2H), 7.04 (dd, *J* = 8.3, 2.5 Hz, 2H), 5.74 (s, 2H), 4.77 (d, 2H). **^{13}C NMR** (126 MHz, D_2O) *δ (ppm)*: 146.1, 144.2, 135.1 (d, *J* = 3.1 Hz), 134.0, 133.2 (d, *J* = 4.1 Hz), 131.8 (d, *J* = 5.2 Hz), 129.9, 129.3 (d, *J* = 3.3 Hz), 128.4, 117.29 (d, *J* = 2.5 Hz), 116.6 (d, *J* = 2.3 Hz), 63.9. **^{31}P{^{1}H} NMR** (400 MHz, D_2O) *δ (ppm)*: 22.57. **HRMS (ESI)** m/z: [$\mathbf{2}D_2$]$^{2+}$ Calcd for $C_{31}H_{26}D_2NP^{2+}$ 223.6037; Found 223.6034; Calcd for $C_{18}H_{15}P^{\bullet+}$ 262.0906; Found 262.0903; Calcd for $C_{26}H_{23}P^{\cdot+}$ 366.1520, Found 366.1515.

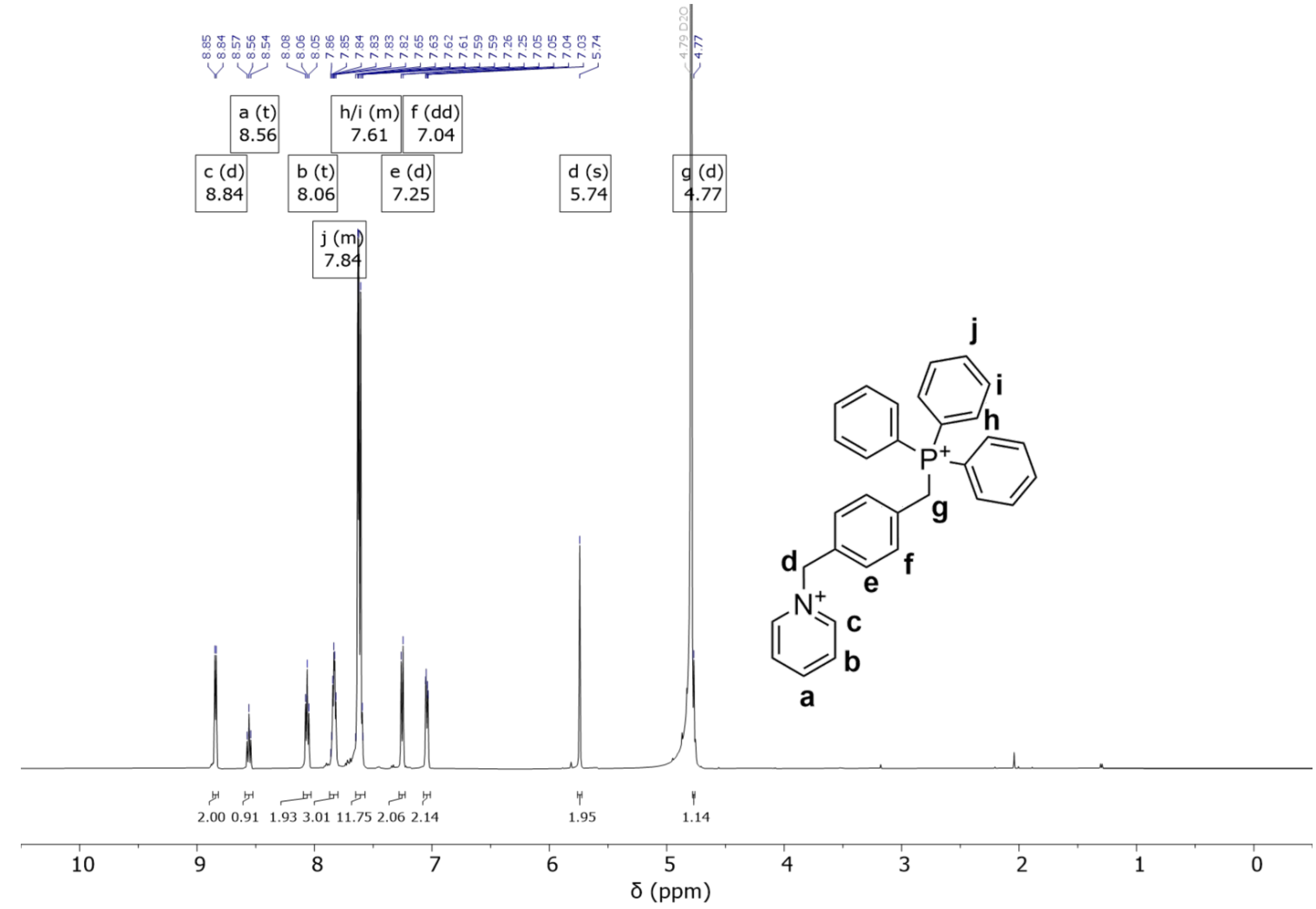

Figure S3: ^{1}H NMR (500 MHz, D_2O) spectrum of **2**·2Br.

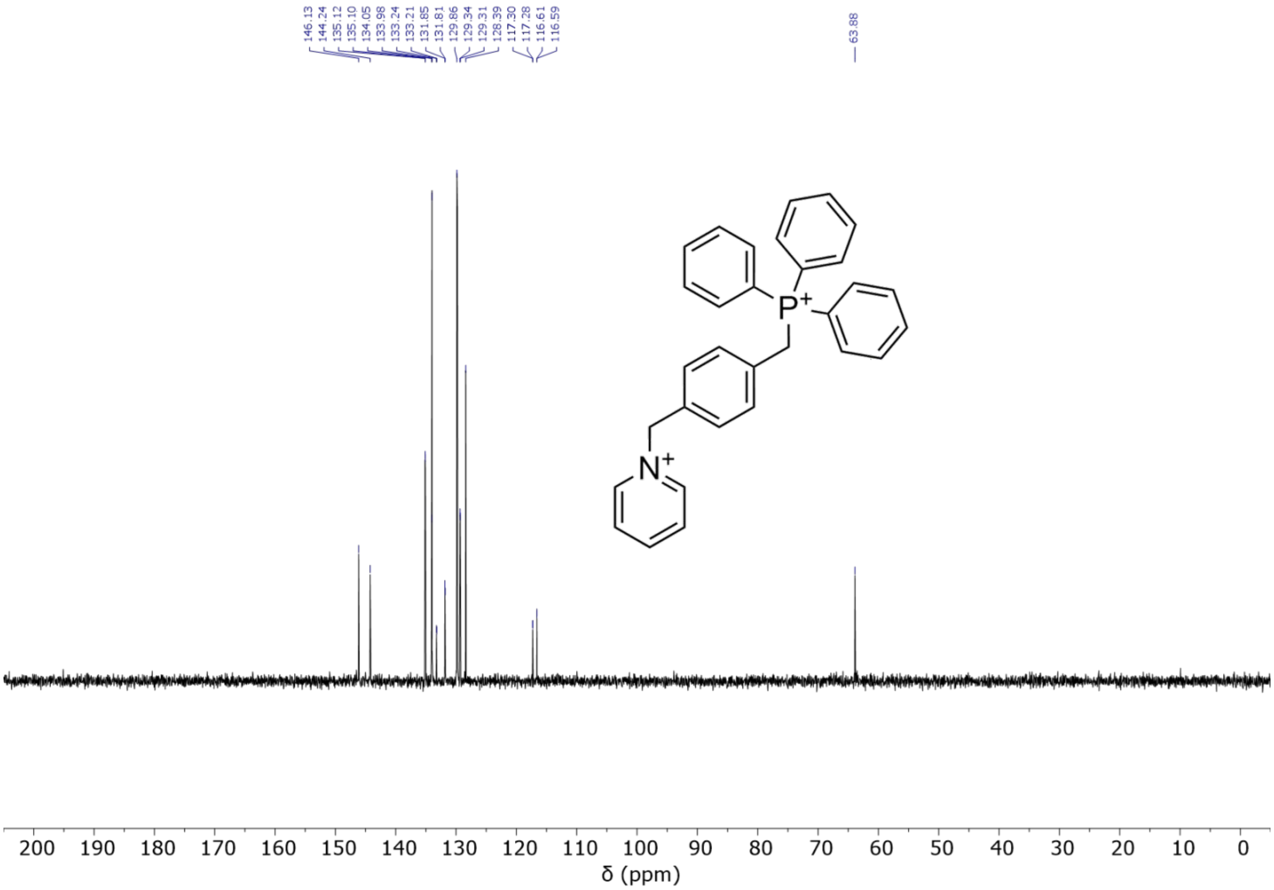

Figure S4: $^{13}C\{^{1}H\}$ NMR (126 MHz, D_2O) spectrum of **2**·2Br.

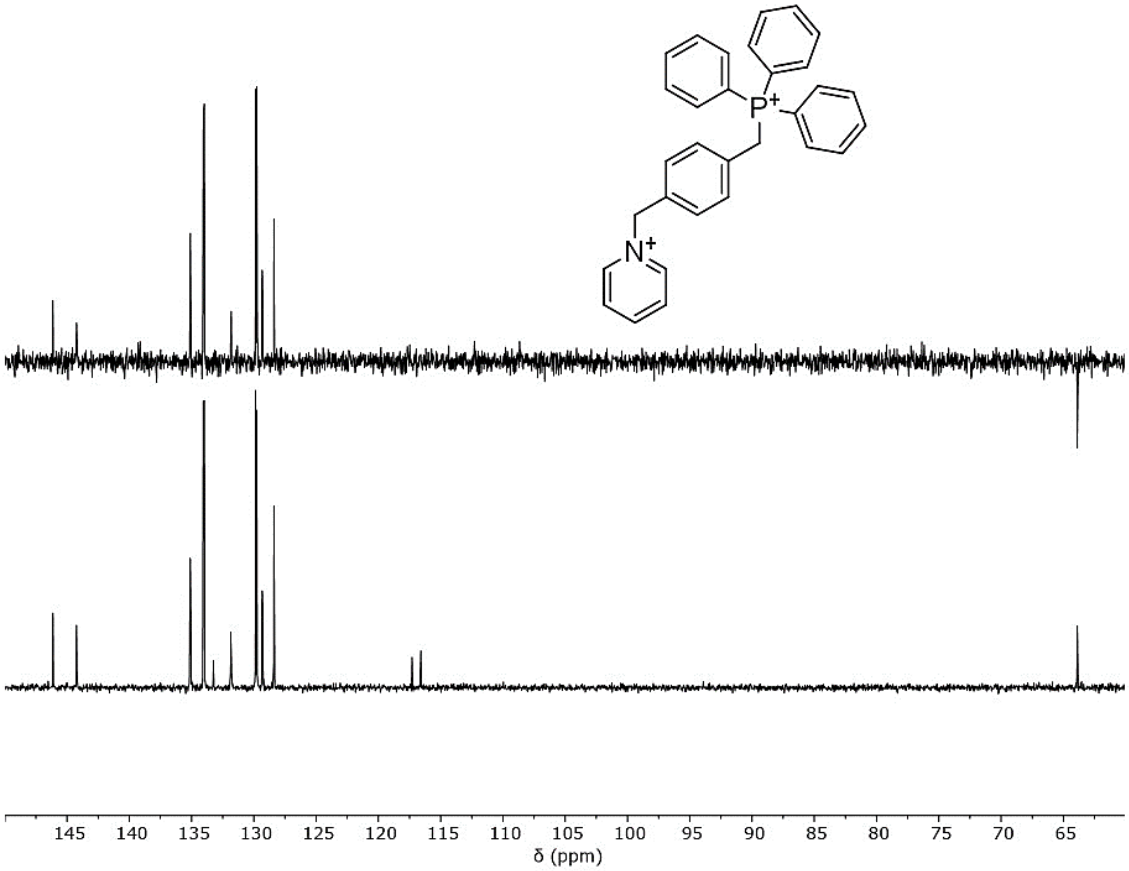

Figure S5: DEPT-135 NMR (126 MHz, D_2O) and $^{13}C\{^1H\}$ NMR (126 MHz, D_2O) spectrum of **2**·2Br.

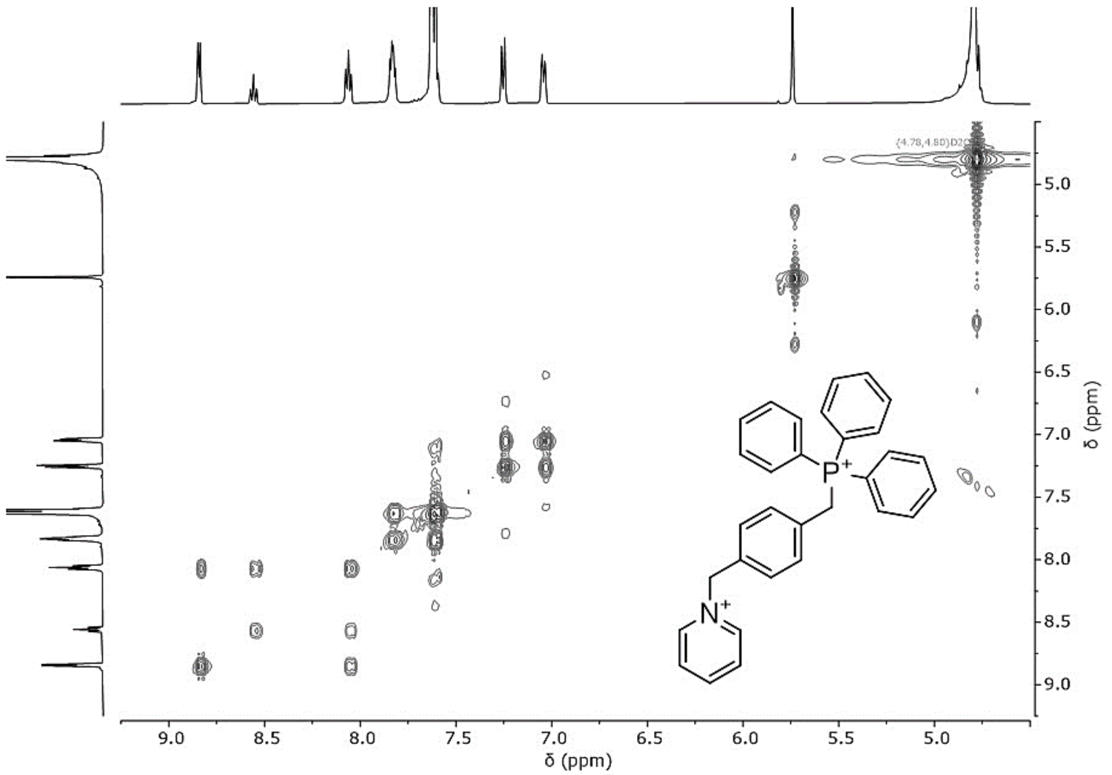

Figure S6: ^{1}H-^{1}H COSY (500 MHz, D_2O) spectrum of **2**·2Br.

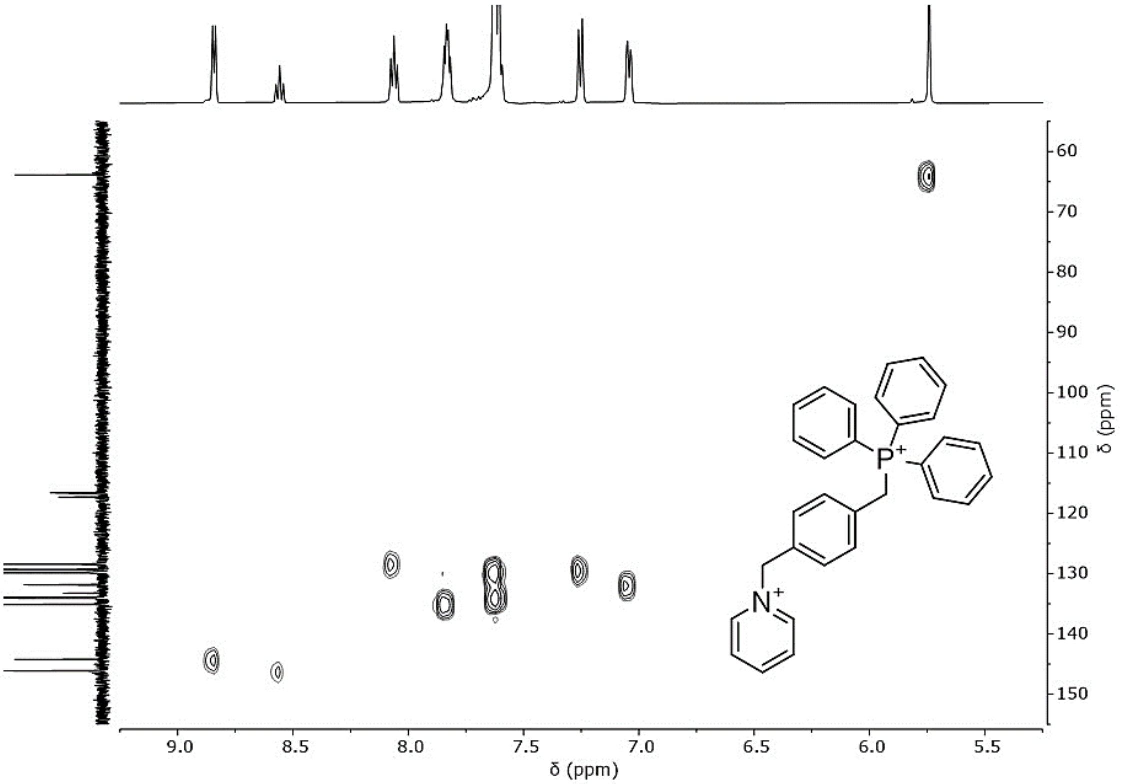

Figure S7: ^{1}H-^{13}C HSQC (500 MHz, D_2O) spectrum of **2**·2Br.

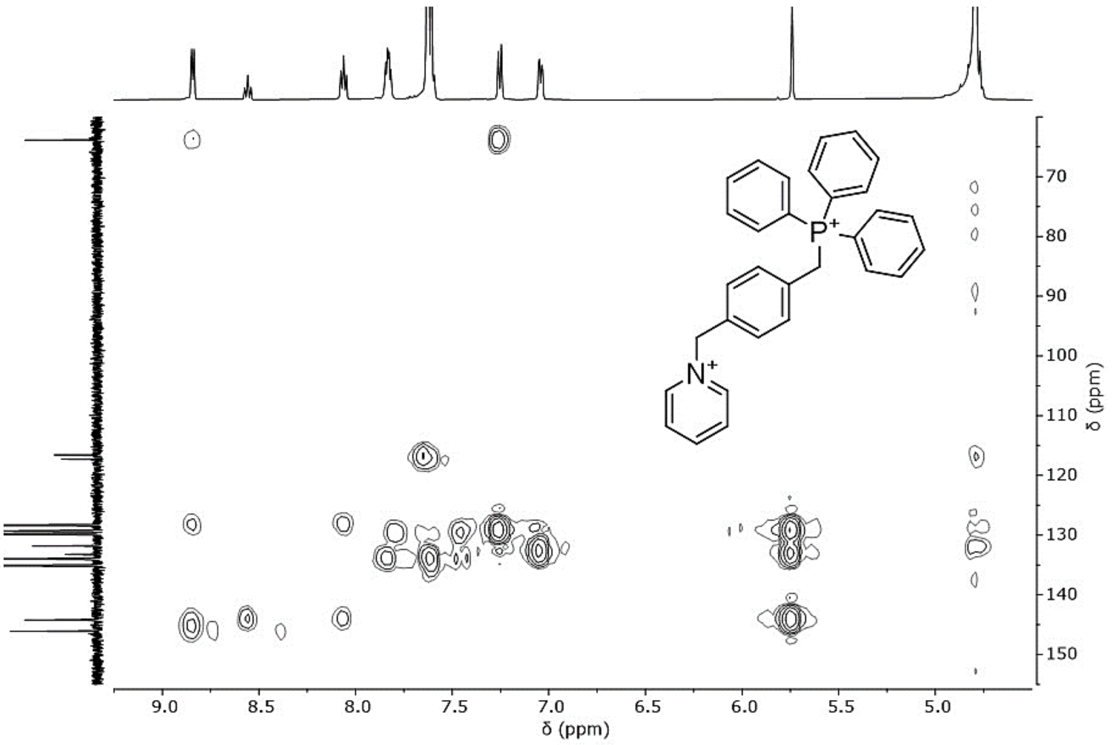

Figure S8: ^{1}H-^{13}C HMBC (500 MHz, D_2O) spectrum of **2**·2Br.

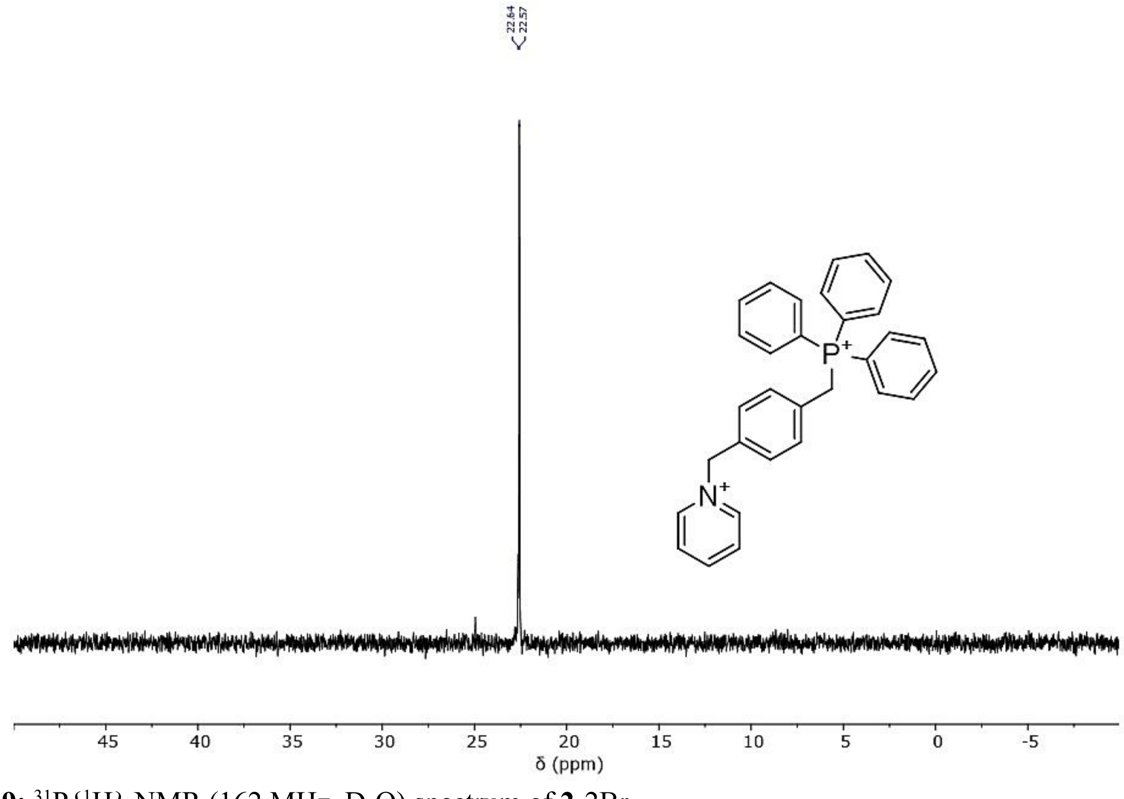

Figure S9: $^{31}P\{^{1}H\}$ NMR (162 MHz, D_2O) spectrum of **2**·2Br.

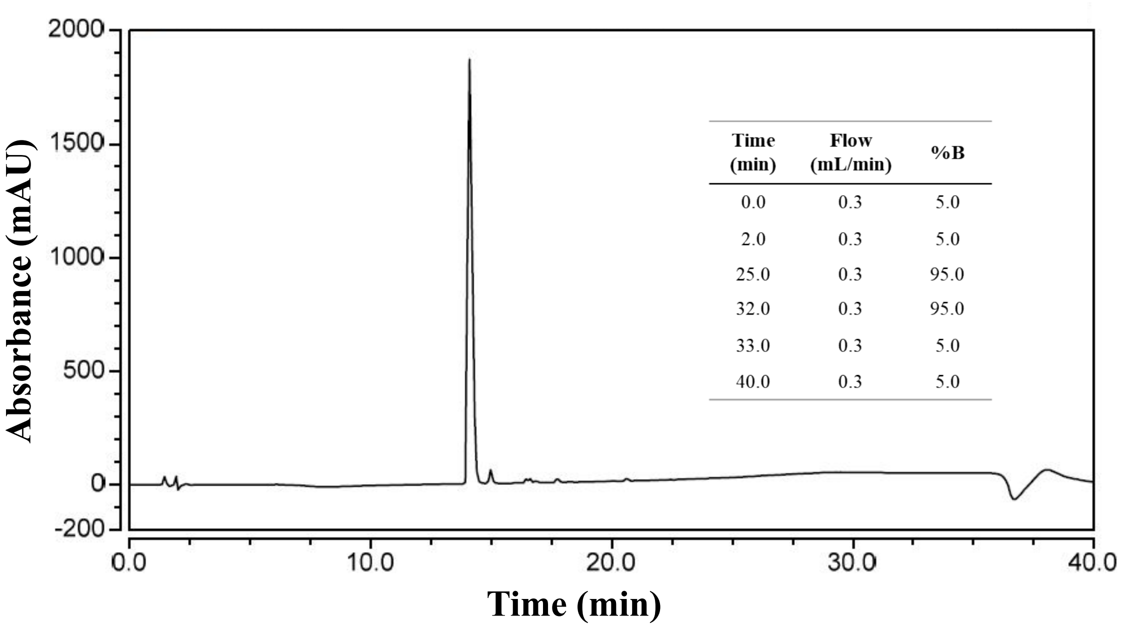

Figure S10: HPLC chromatogram (220 nm) of $\mathbf{2}^{2+}$ at t_R = 14.1 min (Inset: separation method; A = H_2O + 0.04 % TFA, B = CH_3CN + 0.04 % TFA).

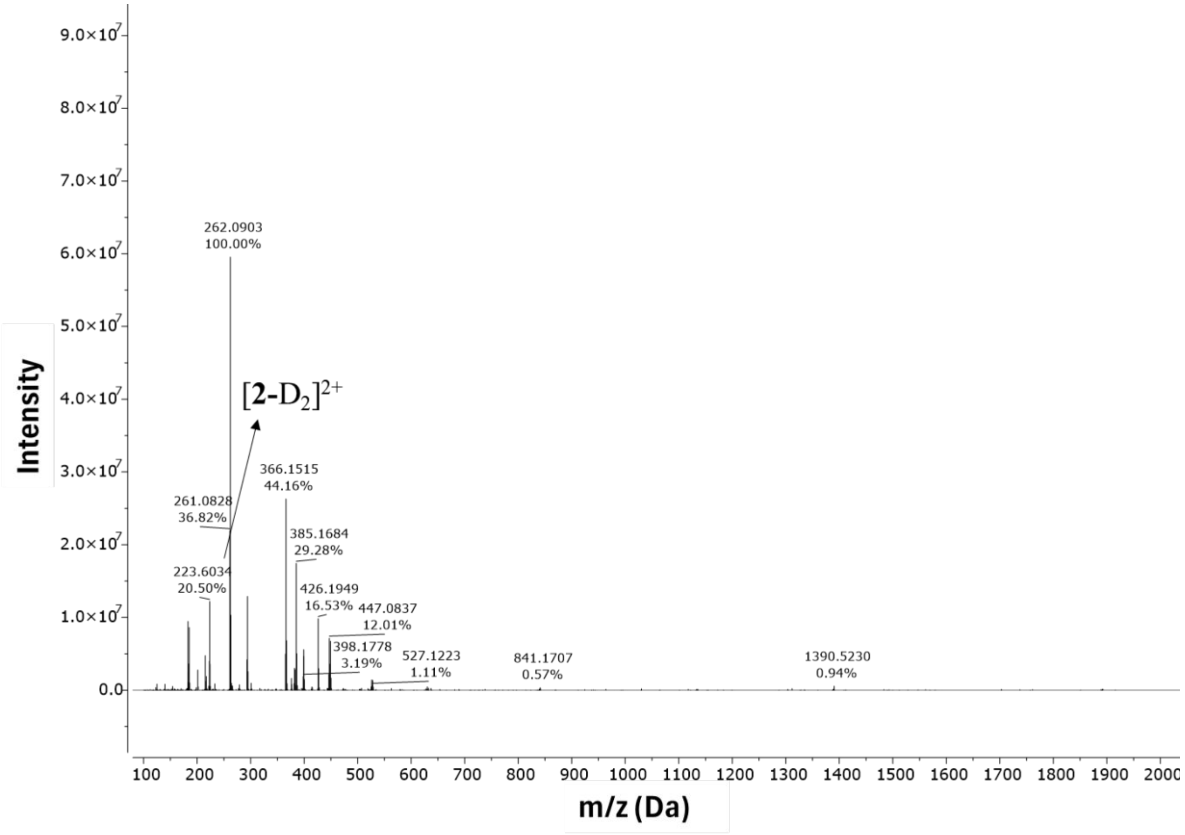

Figure S11: HRMS-ESI spectrum of **2**·Br.

2.3. Synthesis and characterization data of 3·2TFA

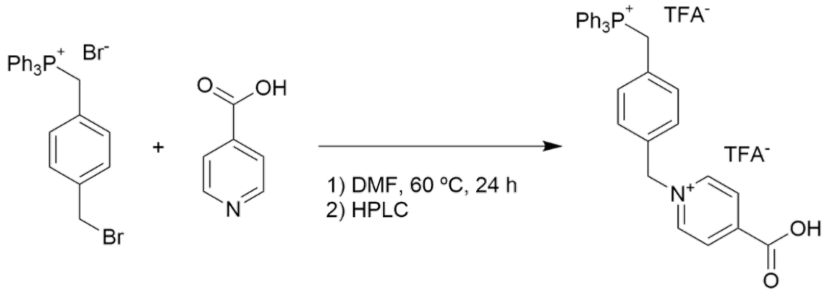

P1·Br 3·2TFA

To a solution of isonicotinic acid (106 mg, 0.855 mmol, 3 eq) in DMF (20 mL) was added dropwise a solution of **P1**·Br (150 mg, 0.285 mmol, 1 eq) in 5 mL of DMF. The mixture was stirred at 60 ºC in a hot plate stirrer for 24 hours. The solvent was removed, then CH_3CN (20 mL) was added, and the resulting suspension was filtered under vacuum. The organic phase was evaporated under reduced pressure to leave a residue (123 mg) which was purified through preparative HPLC with the method described in **Table S1**. The product containing fractions were lyophilized to yield **3**·2TFA as a white solid (67 mg, 36 %), which purity was assessed through analytical HPLC chromatography and HRMS-ESI.

^{1}H NMR (500 MHz, D_2O) *δ (ppm)*: 8.89 (d, *J* = 6.4 Hz, 2H), 8.32 (d, *J* = 6.3 Hz, 2H), 7.79 – 7.71 (m, 3H), 7.58 – 7.49 (m, 12H), 7.20 (d, *J* = 7.9 Hz, 2H), 6.97 (dd, *J* = 8.2, 2.5 Hz, 2H), 5.72 (s, 2H), 4.69 (s, 2H). ^{13}**C{^{1}H} NMR** (126 MHz, D_2O) *δ (ppm)*: 166.3, 148.9, 145.3, 135.1 (d, *J* = 3.1 Hz), 134.0 (d, *J* = 9.9 Hz), 132.9 (d, *J* = 4.1 Hz), 131.9 (d, *J* = 5.4 Hz), 129.9 (d, *J* = 12.8 Hz), 129.6 – 129.4 (m), 127.6, 117.0 (d, *J* = 86.8 Hz), 64.1, 29.3 (d, *J* = 48.3 Hz). **^{31}P{^{1}H} NMR** (162 MHz, D_2O) *δ (ppm)*: 22.66. **HRMS (ESI)** m/z: [**3**]$^{2+}$ Calcd for $C_{32}H_{28}NO_2P^{2+}$ 244.5924; Found 244.5923.

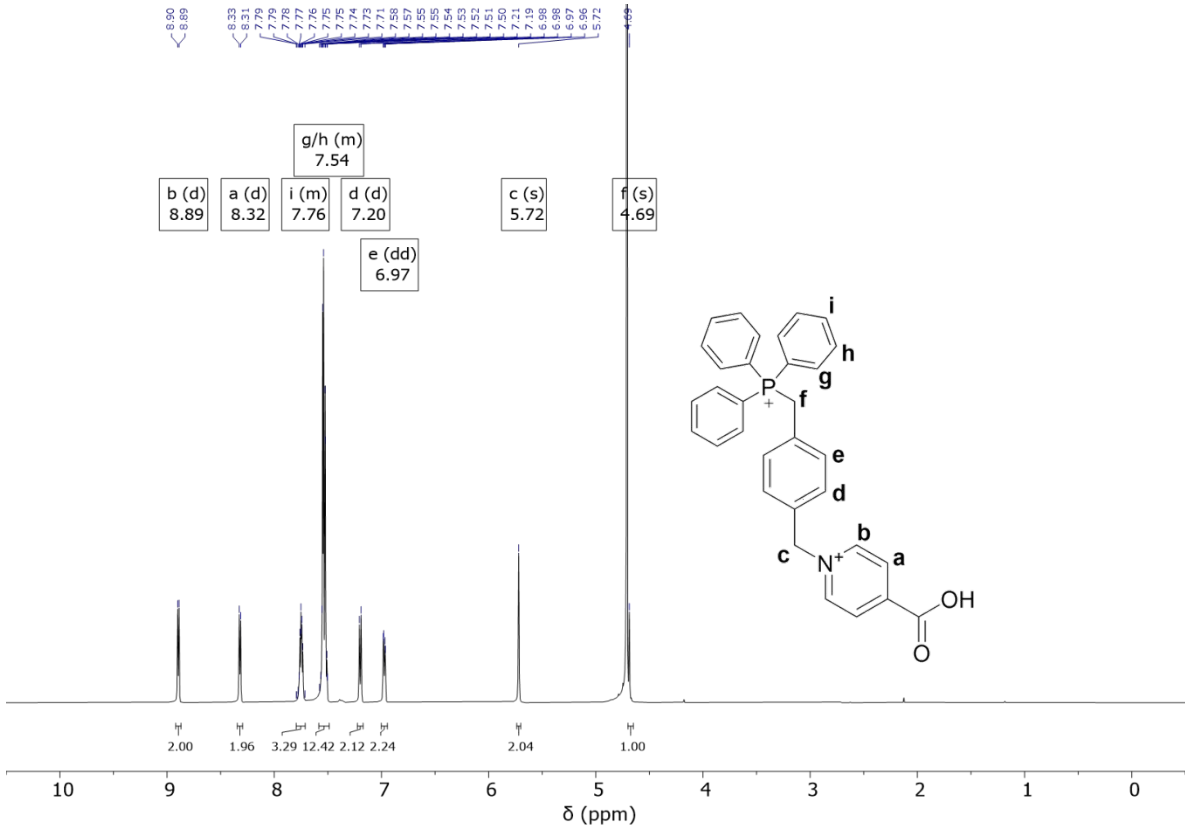

Figure S12: ^{1}H NMR (500 MHz, D_2O) spectrum of **3**·2TFA.

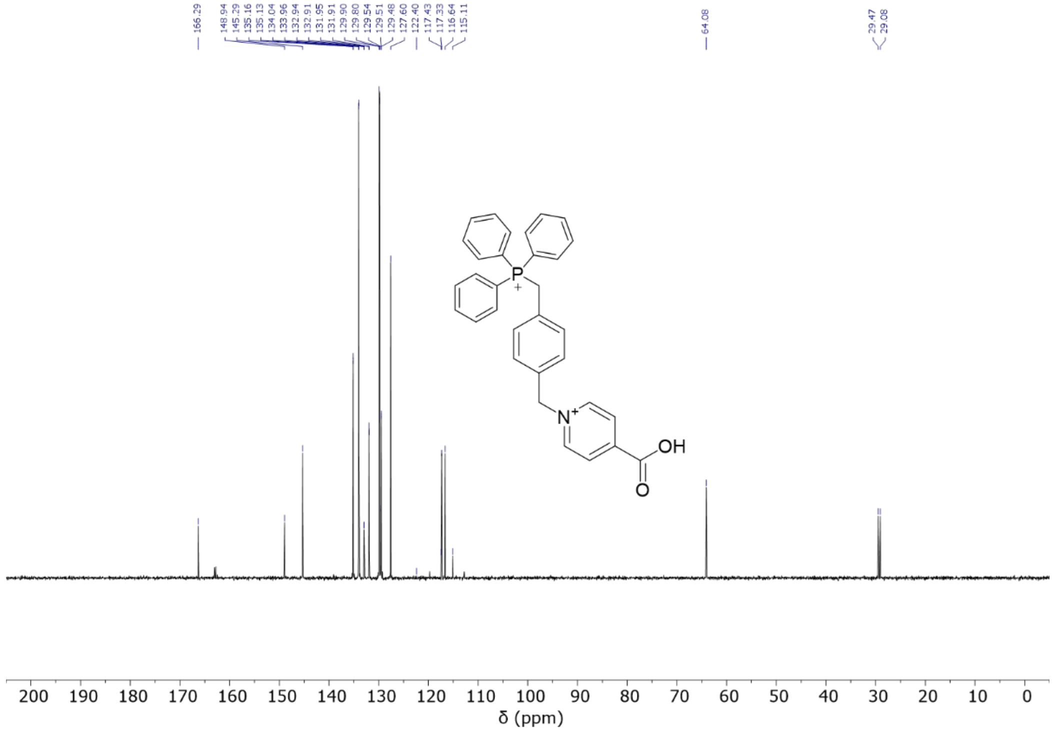

Figure S13: $^{13}C\{^{1}H\}$ NMR (126 MHz, D_2O) spectrum of **3**·2TFA.

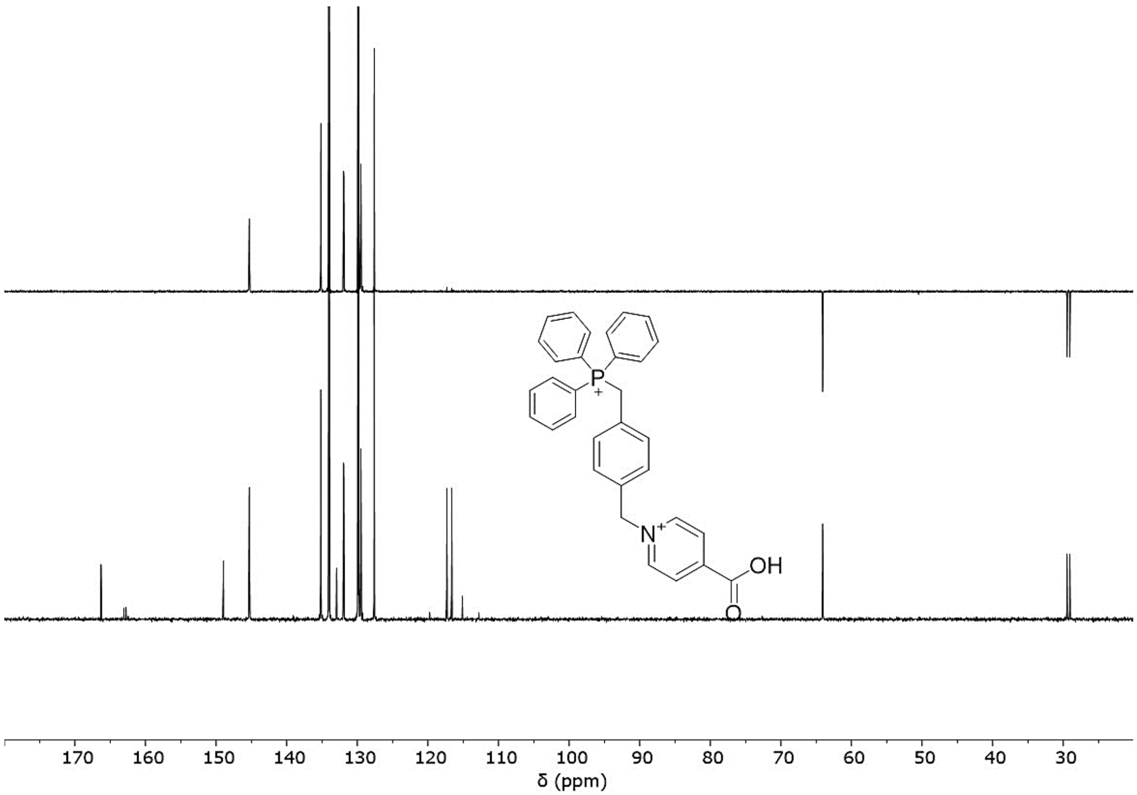

Figure S14: DEPT-135 (126 MHz, D_2O) spectrum (up) and $^{13}C\{^1H\}$ NMR (126 MHz, D_2O) spectrum (down) of **3**·2TFA.

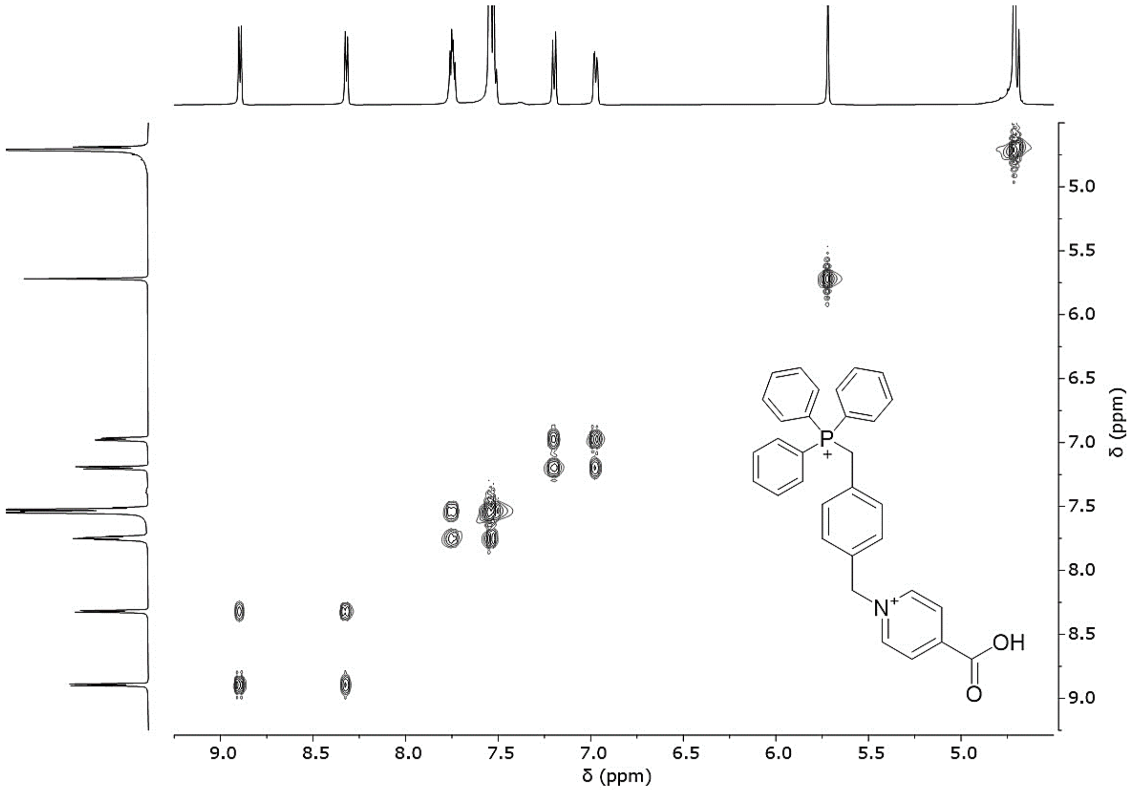

Figure S15: ^{1}H-^{1}H COSY (500 MHz, D_2O) spectrum of **3**·2TFA.

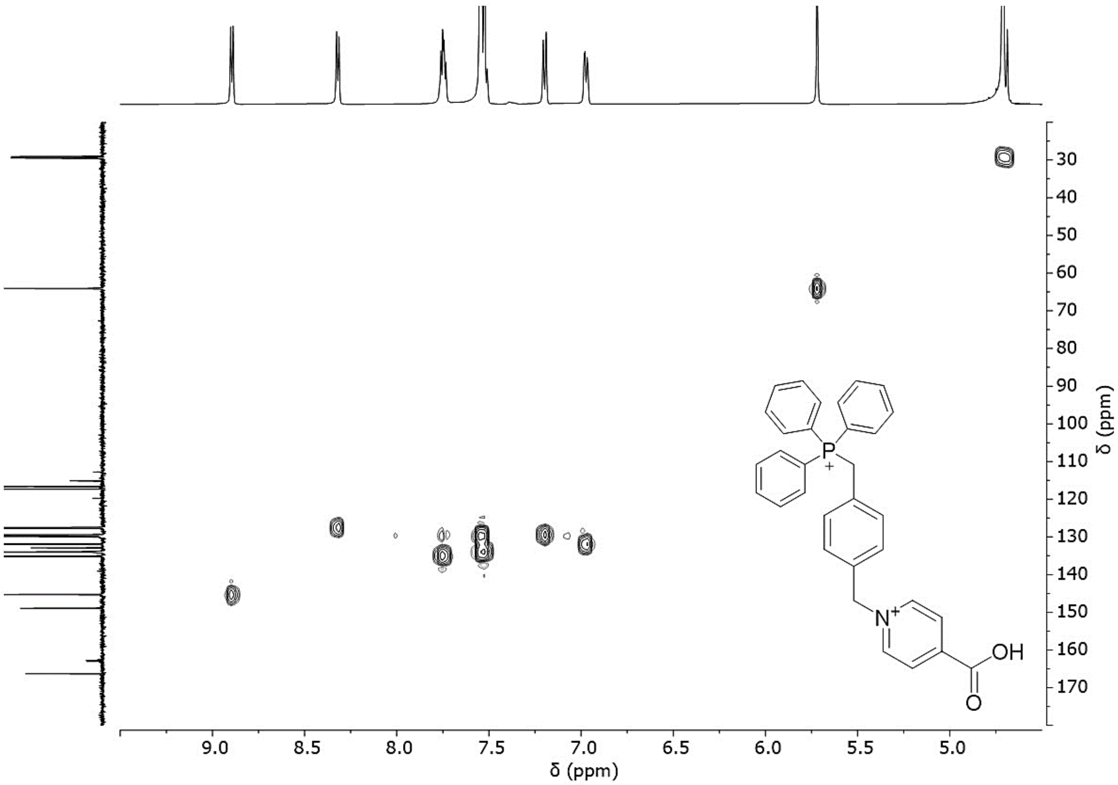

Figure S16: ^{1}H-^{13}C HSQC (500 MHz, D_2O) spectrum of **3**·2TFA.

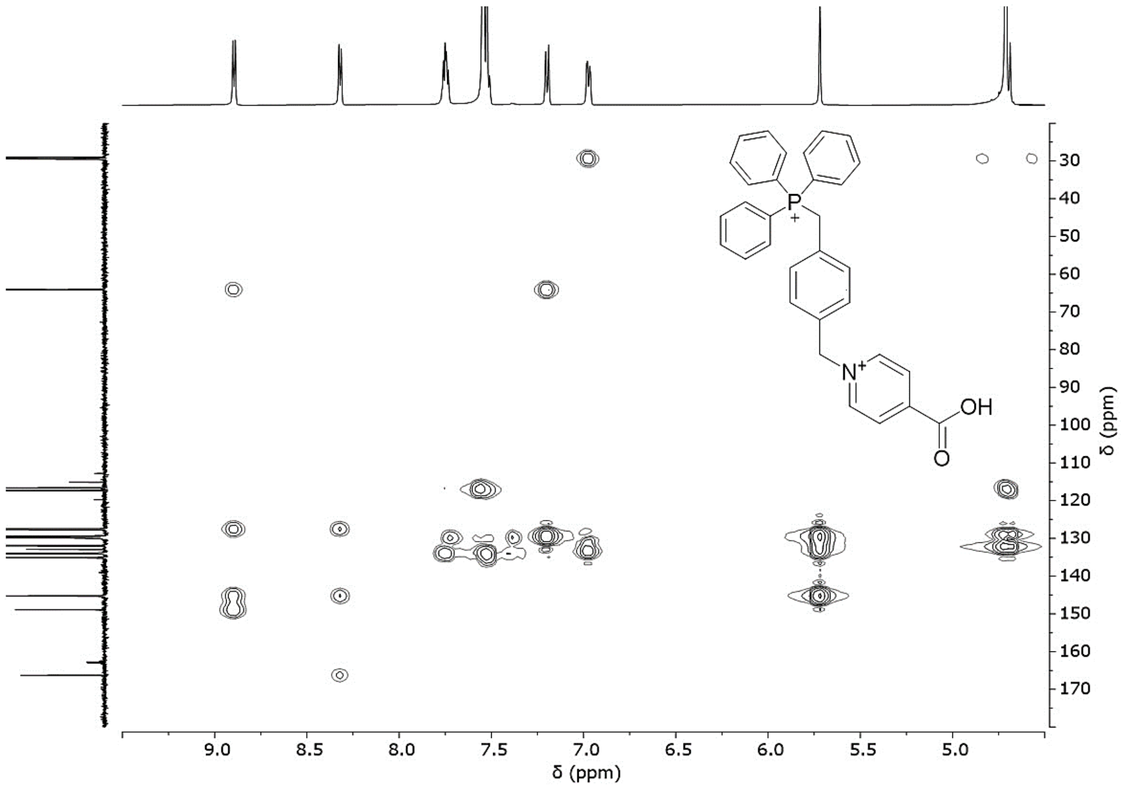

Figure S17: ^{1}H-^{13}C HMBC (500 MHz, D_2O) spectrum of **3**·2TFA.

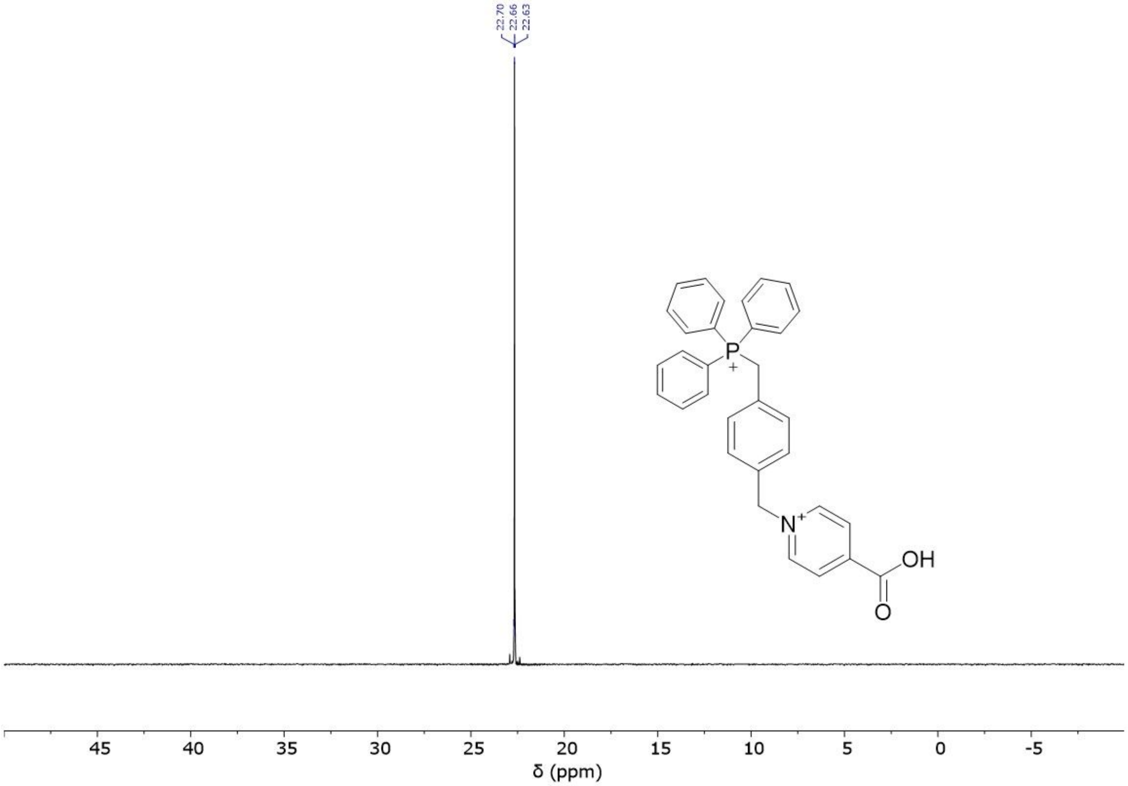

Figure S18: $^{31}P\{^{1}H\}$ NMR (162 MHz, D_2O) spectrum of **3**·2TFA.

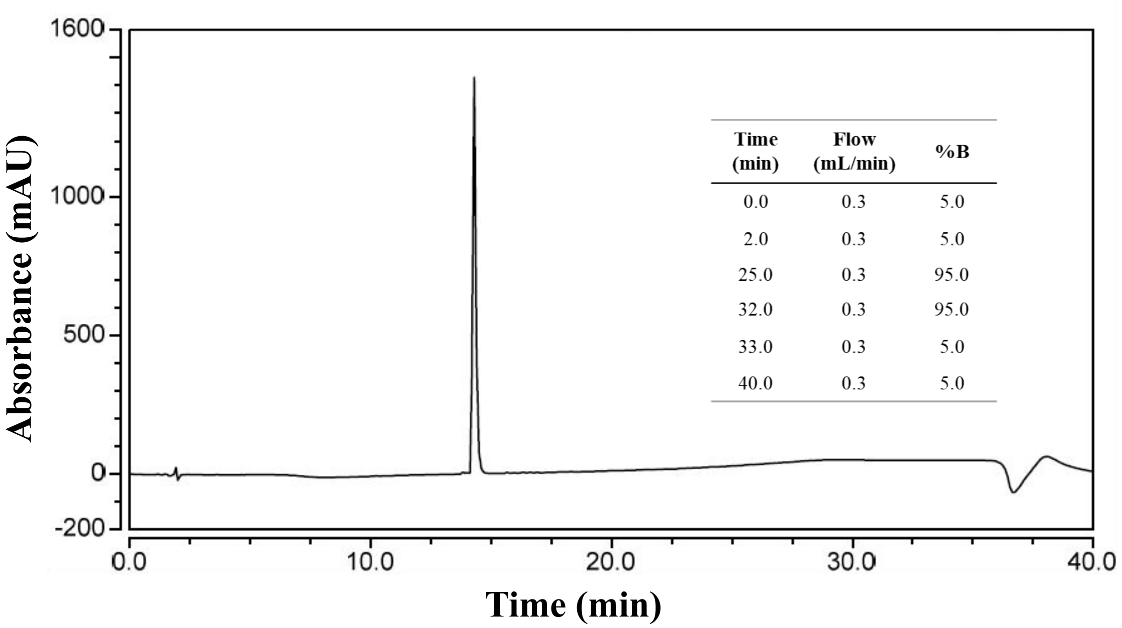

Figure S19: HPLC chromatogram (220 nm) of purified $\mathbf{3}^{2+}$ at t_R = 13.3 min (Inset: separation method; A = H_2O + 0.04 % TFA, B = CH_3CN + 0.04 % TFA).

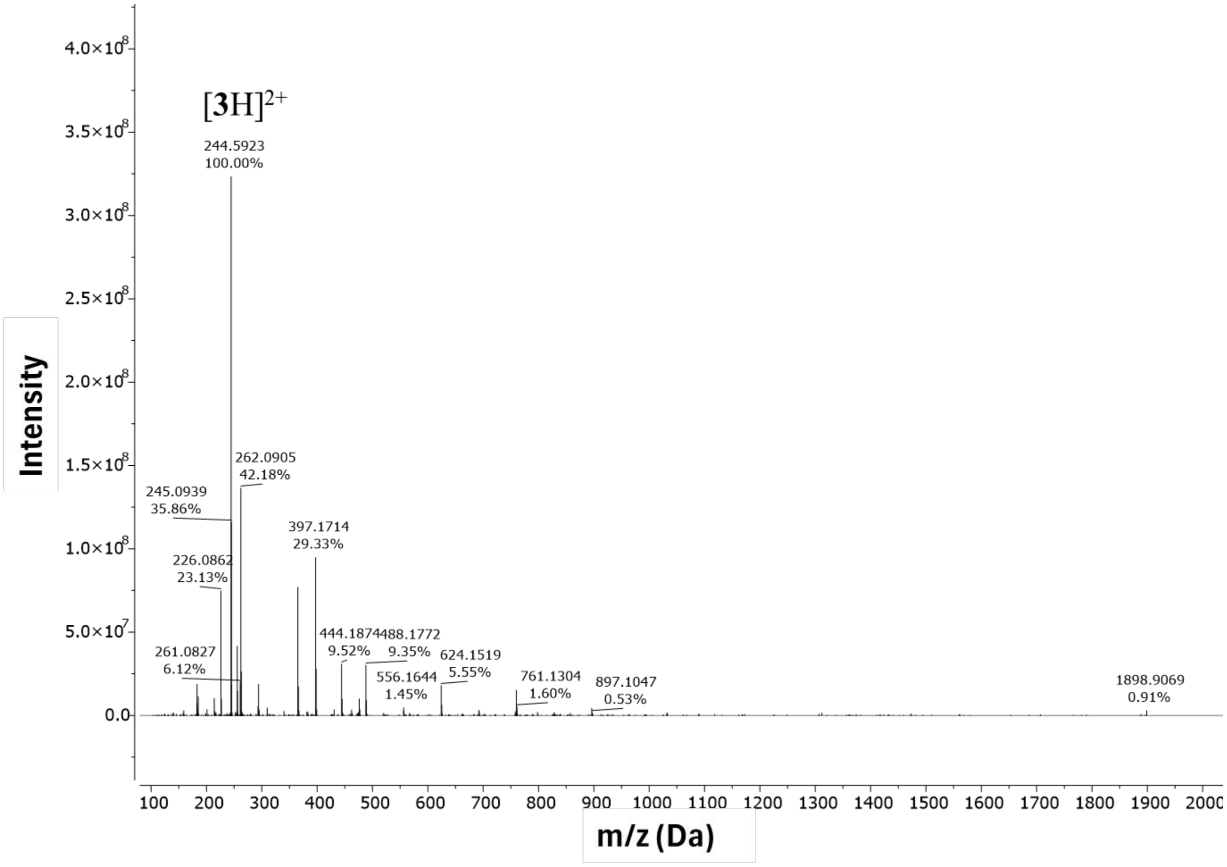

Figure S20: HRMS-ESI spectrum of **3**·2TFA.

2.4. Synthesis and characterization data of 4·3Cl

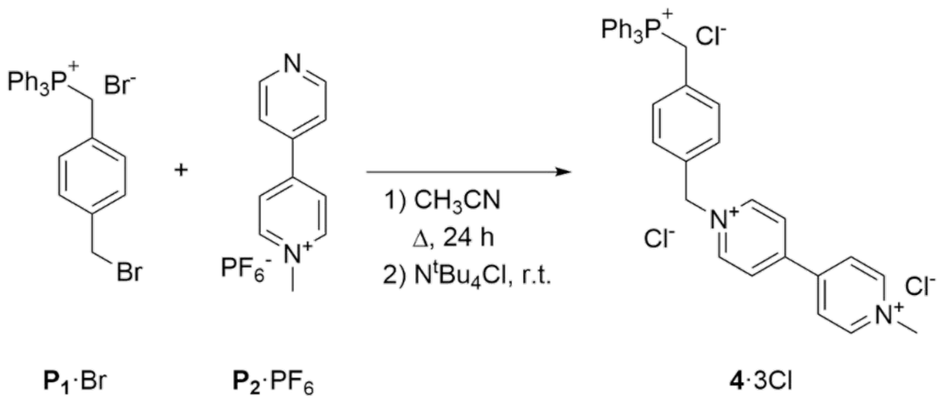

A mixture of $\mathbf{P_1}$·Br (162 mg, 0.307 mmol, 1 eq) and $\mathbf{P_2}$·PF_6 (292 mg, 0.921 mmol, 3 eq) in CH_3CN (15 mL) was stirred under reflux in a hot plate stirrer for 24 hours. The solvent was removed under vacuum and the crude was washed with H_2O (2 × 15 mL) and dried with Et_2O (3 × 20 mL) to leave a solid which was redissolved in CH_3CN (15 mL). Then, N^tBu_4Cl was added (500 mg, 1.5 mmol, 5 eq) and the mixture was stirred at room temperature for 24 hours. The precipitate was filtered under vacuum, washed with CH_3CN (2 × 15 mL) and dried with Et_2O (3 × 15 mL), to leave **4**·3Cl as a brown solid (135 mg, 52 %).

^{1}H NMR (500 MHz, D_2O) *δ (ppm)*: 9.09 (d, J = 6.9 Hz, 2H), 9.02 (d, J = 6.9 Hz, 2H), 8.52 (d, J = 6.9 Hz, 2H), 8.48 (d, J = 6.8 Hz, 2H), 7.83 (m, 3H), 7.62 (m, 12H), 7.30 (d, J = 7.9 Hz, 2H), 7.07 (dd, J = 8.3, 2.5 Hz, 2H), 5.85 (s, 2H), 4.77 (s, 2H), 4.47 (s, 3H). **^{13}C NMR** (126 MHz, D_2O) *δ (ppm)*: 150.6, 149.5, 146.3, 145.4, 135.1 (d, *J* = 3.1 Hz), 134.1, 132.7 (d, *J* = 4.0 Hz), 132.0 (d, *J* = 5.3 Hz), 129.9, 129.6 – 129.5 (m), 127.1, 126.6, 116.9 (d, *J* = 86.9 Hz), 64.1, 48.3, 29.3 (d, *J* = 49.0 Hz). **^{31}P{^{1}H} NMR** (400 MHz, D_2O) *δ (ppm)*: 22.60. **HRMS (ESI)** m/z: [**4**]$^{•2+}$·Calcd for $C_{37}H_{34}N_2P^{•2+}$ 268.6225; Found 268.6213.

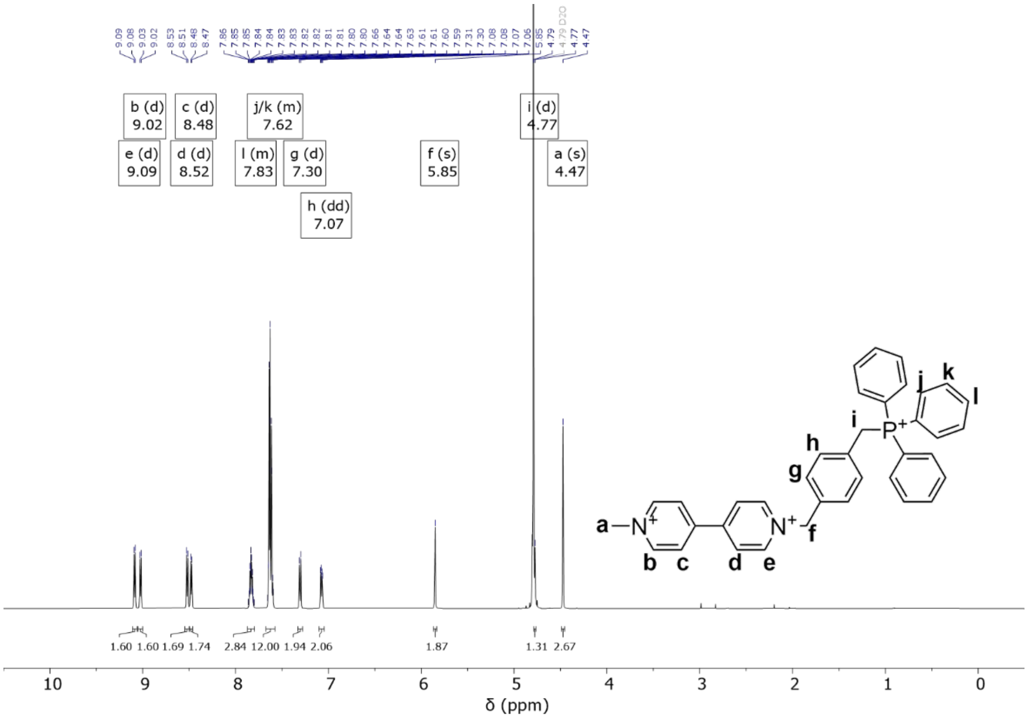

Figure S21: ^{1}H NMR (500 MHz, D_2O) spectrum of **4**·3Cl.

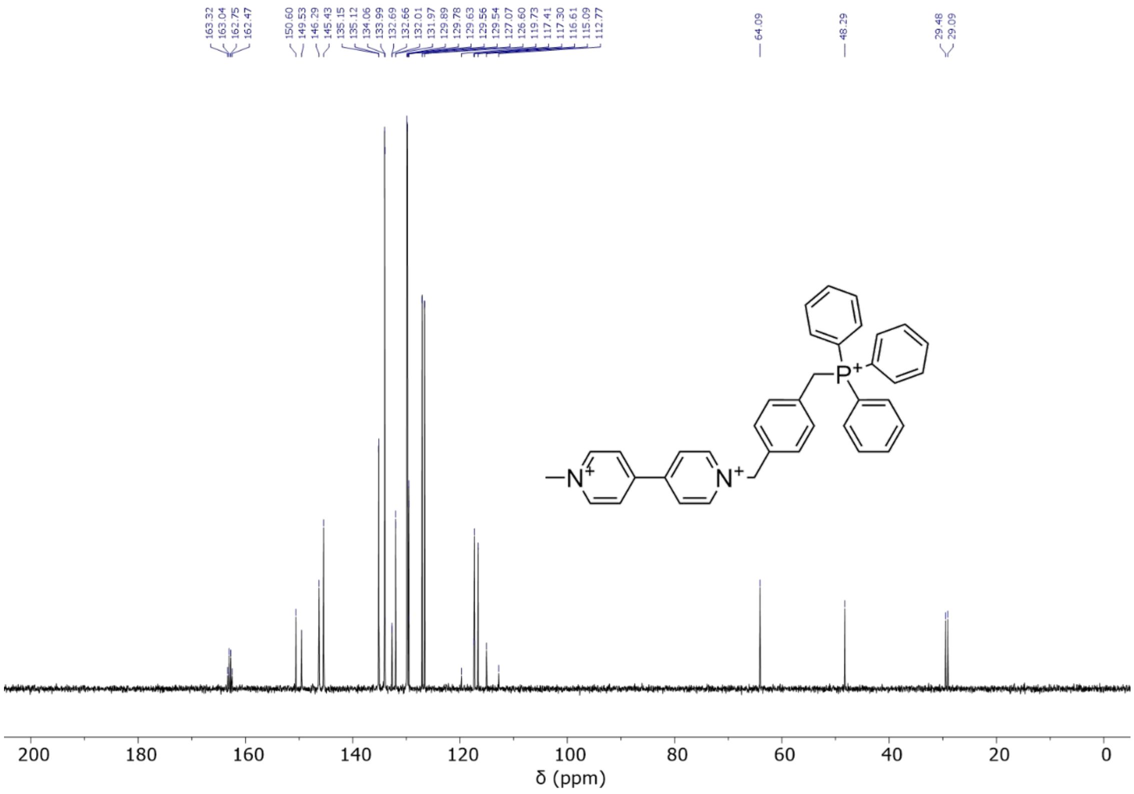

Figure S22: ^{13}C{^{1}H} NMR (126 MHz, D_2O) spectrum of **4**·3Cl.

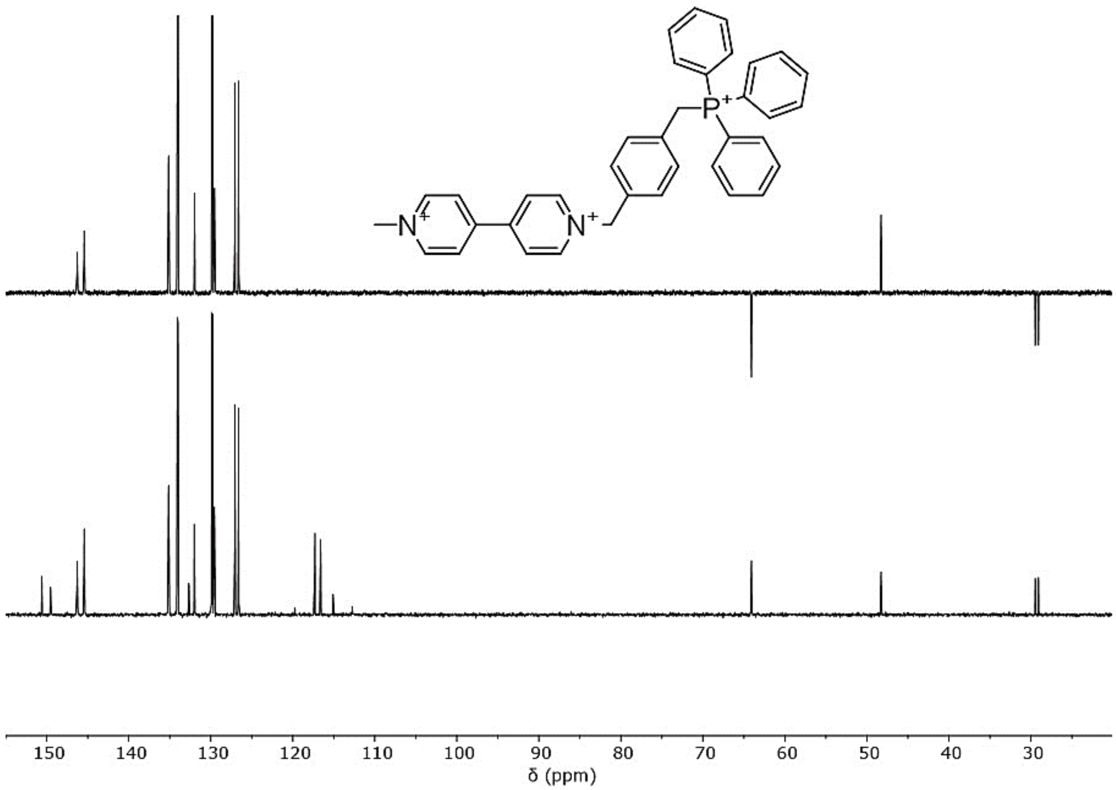

Figure S23: DEPT-135 NMR (126 MHz, D_2O) and $^{13}C\{^1H\}$ NMR (126 MHz, D_2O) spectrum of **4**·3Cl.

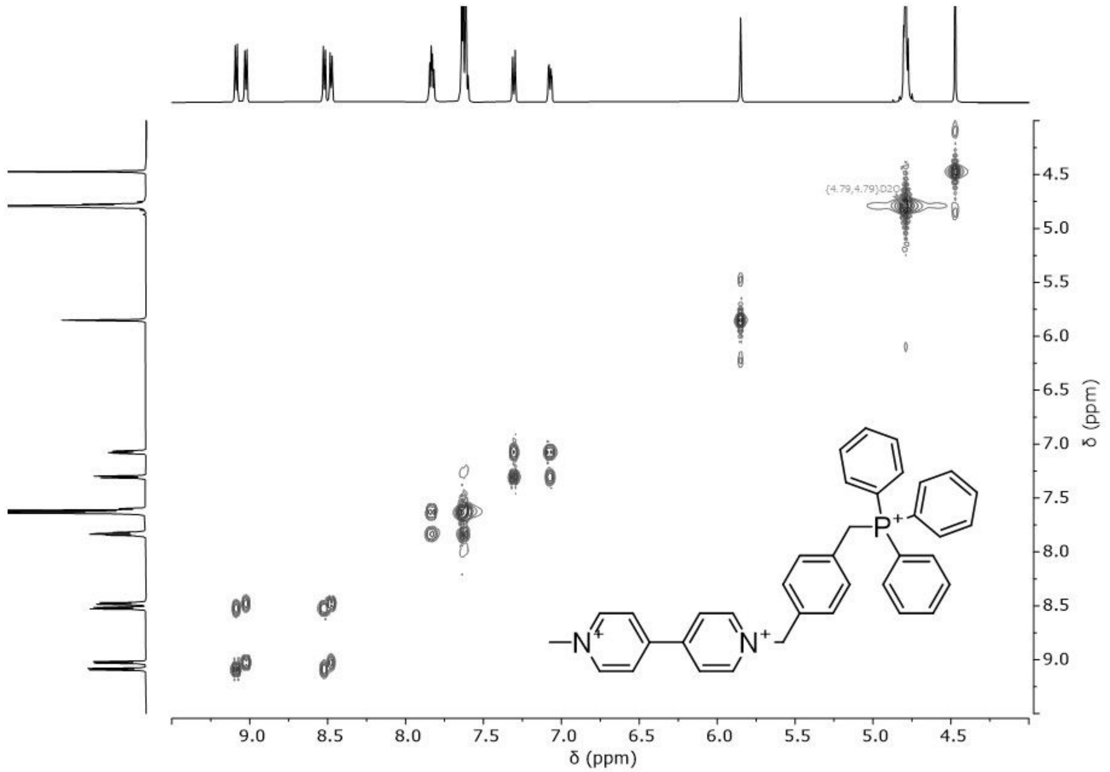

Figure S24: ^1H-^1H COSY (500 MHz, D_2O) spectrum of **4**·3Cl.

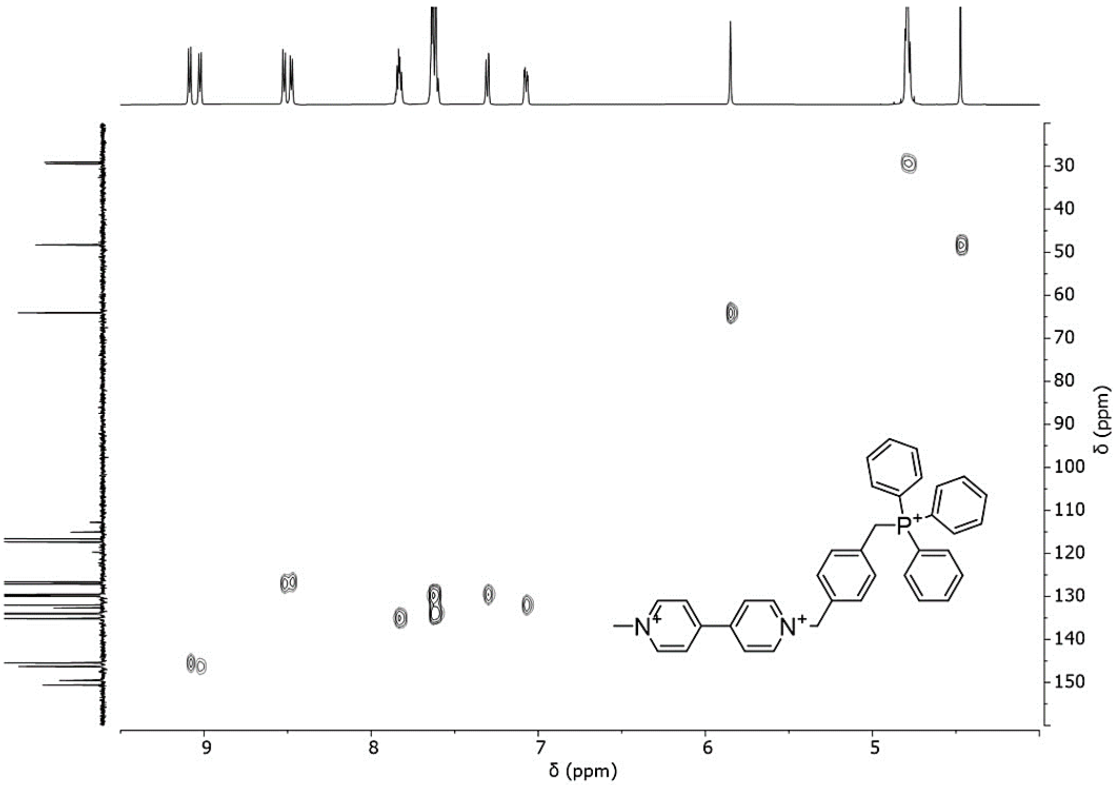

Figure S25: ^{1}H-^{13}C HSQC (500 MHz, D_2O) spectrum of **4**·3Cl.

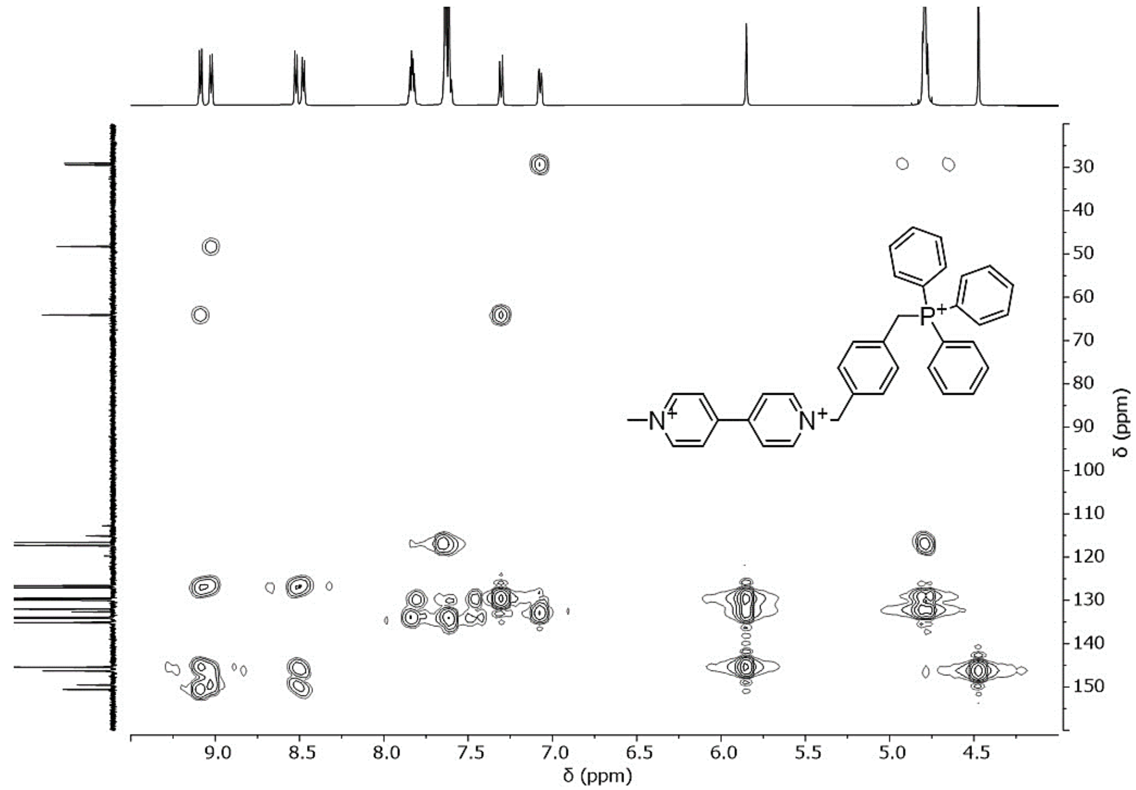

Figure S26: ^{1}H-^{13}C HMBC (500 MHz, D_2O) spectrum of **4**·3Cl.

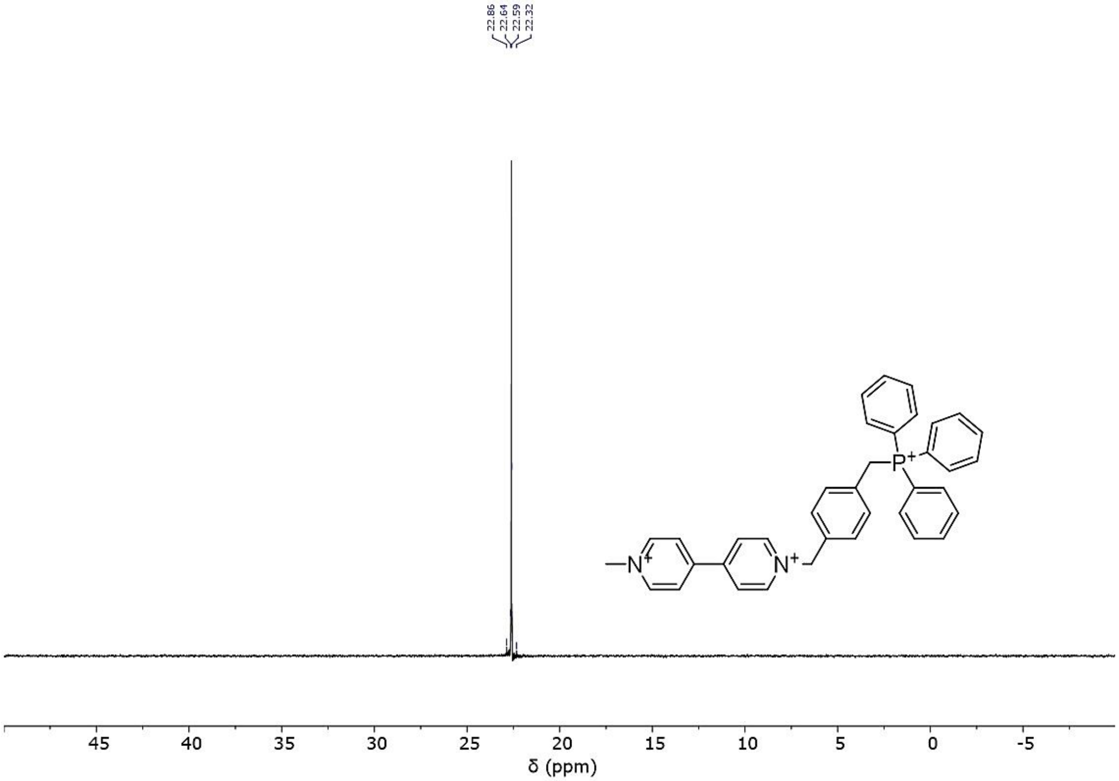

Figure S27: $^{31}P\{^{1}H\}$ NMR (162 MHz, D_2O) spectrum of **4**·3Cl.

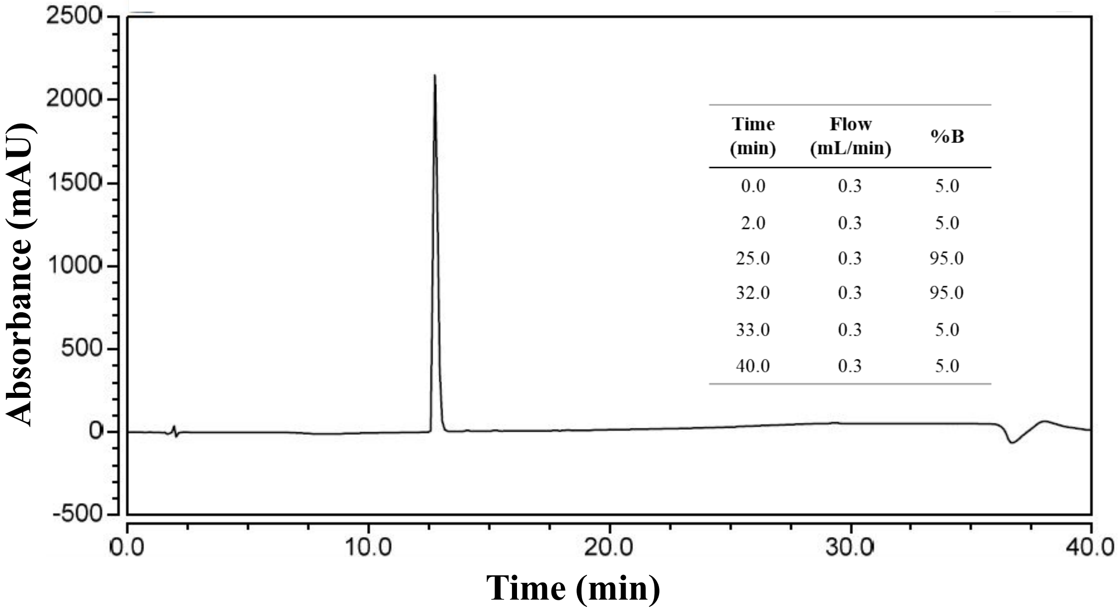

Figure S28: HPLC chromatogram (220 nm) of **4**$^{3+}$ at t_R = 12.8 min (Inset: separation method; A = H_2O + 0.04 % TFA, B = CH_3CN + 0.04 % TFA).

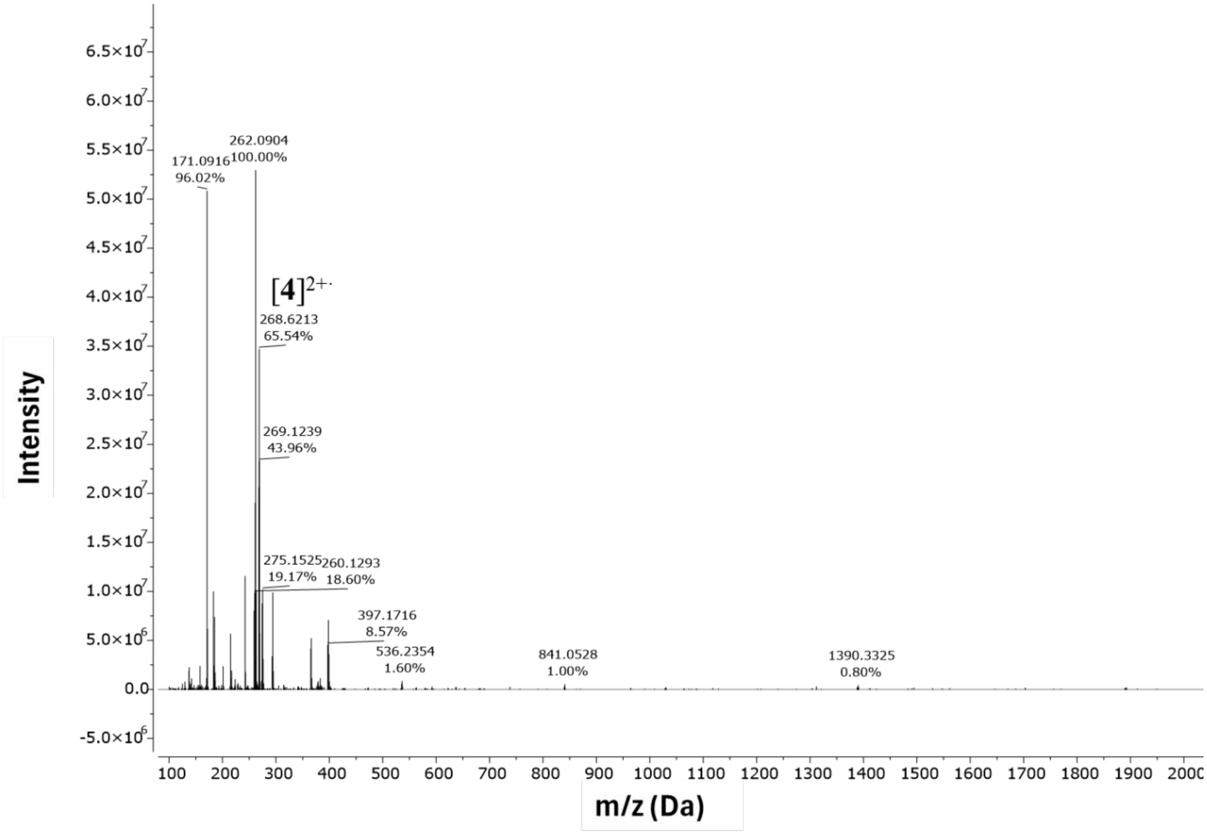

Figure S29: HRMS-ESI spectrum of **4**·3Cl.

2.5. Synthesis and characterization data of 5·2Br

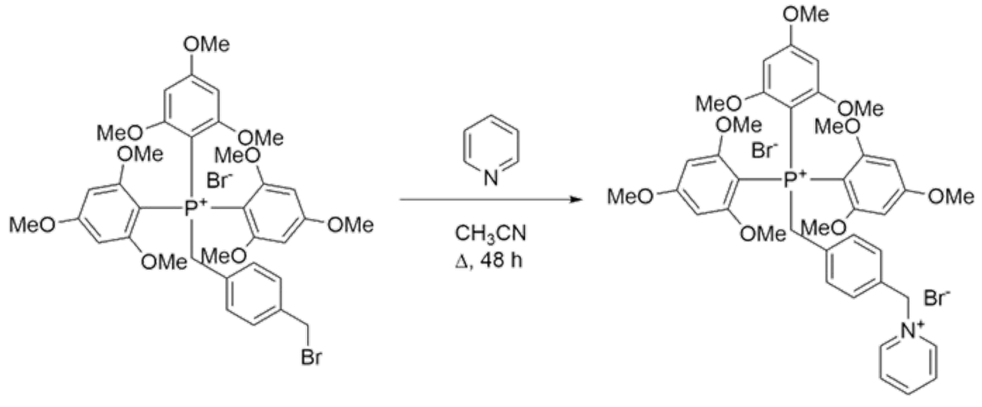

P3·Br **5**·2Br

To a solution of **P_3**·Br (200 mg, 0.251 mmol, 1 eq) in CH_3CN (20 mL) was added pyridine (61 µL, 60 mg, 0.753 mmol, 3 eq). The mixture was stirred under reflux in a hot plate stirrer for 48 hours. The solvent was removed under vacuum and the crude was washed with Et_2O (3 × 10 mL) to leave a solid witch was subjected to flash chromatography (SiO_2, solvent CH_3CN/NaCl (0.6 M)/MeOH: 4:1:1). The product containing fractions were combined and evaporated to yield **5**·2Br as a white solid (198 mg, 92 %).

^{1}H NMR (500 MHz, D_2O) *δ (ppm)*: 8.66 (d, 2H), 8.59 – 8.52 (m, 1H), 8.07 – 8.00 (m, 2H), 7.17 (d, *J* = 2.0 Hz, 4H), 6.14 – 6.09 (m, 6H), 5.60 (s, 2H), 4.72 (d, *J* = 17.2 Hz, 2H), 3.77 (s, 9H), 3.59 – 3.48 (m, 18H). **^{13}C{^{1}H} NMR** (126 MHz, D_2O) *δ (ppm)*: 165.2 (d, *J* = 1.8 Hz), 164.9, 163.3, 145.9, 143.7, 135.9 (d, *J* = 6.8 Hz), 131.3 (d, *J* = 3.6 Hz), 130.7 (d, *J* = 8.3 Hz), 128.5 (d, *J* = 2.4 Hz), 128.3, 92.7 (d, *J* = 105.7 Hz), 91.0 (d, *J* = 7.2 Hz), 64.2, 55.5, 35.2 (d, *J* = 56.6 Hz). **^{31}P{^{1}H} NMR** (162 MHz, D_2O) *δ (ppm)*: 5.78. **HRMS (ESI)** m/z: [**5**]$^{2+}$ Calcd for $C_{40}H_{46}NO_9P^{2+}$ 357.6450; Found 357.6449.

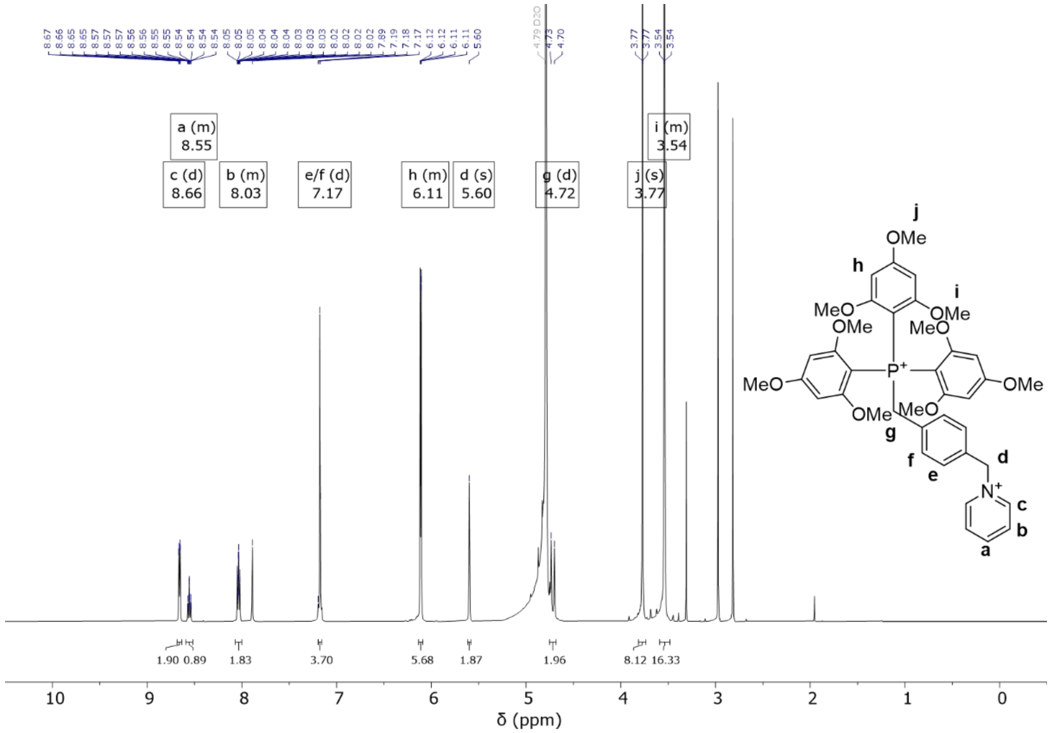

Figure S30: ^{1}H NMR (500 MHz, D_2O) spectrum of **5**·2Br.

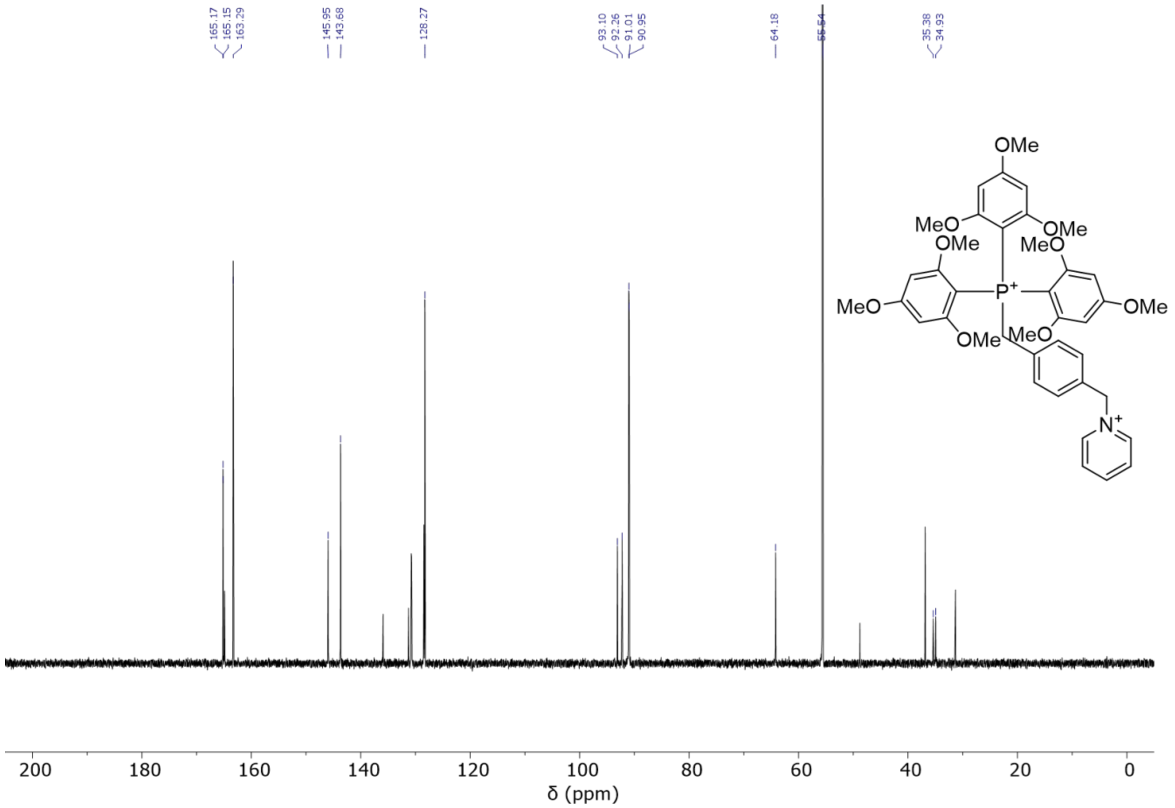

Figure S31: $^{13}C\{^{1}H\}$ NMR (126 MHz, D_2O) spectrum of **5**·2Br.

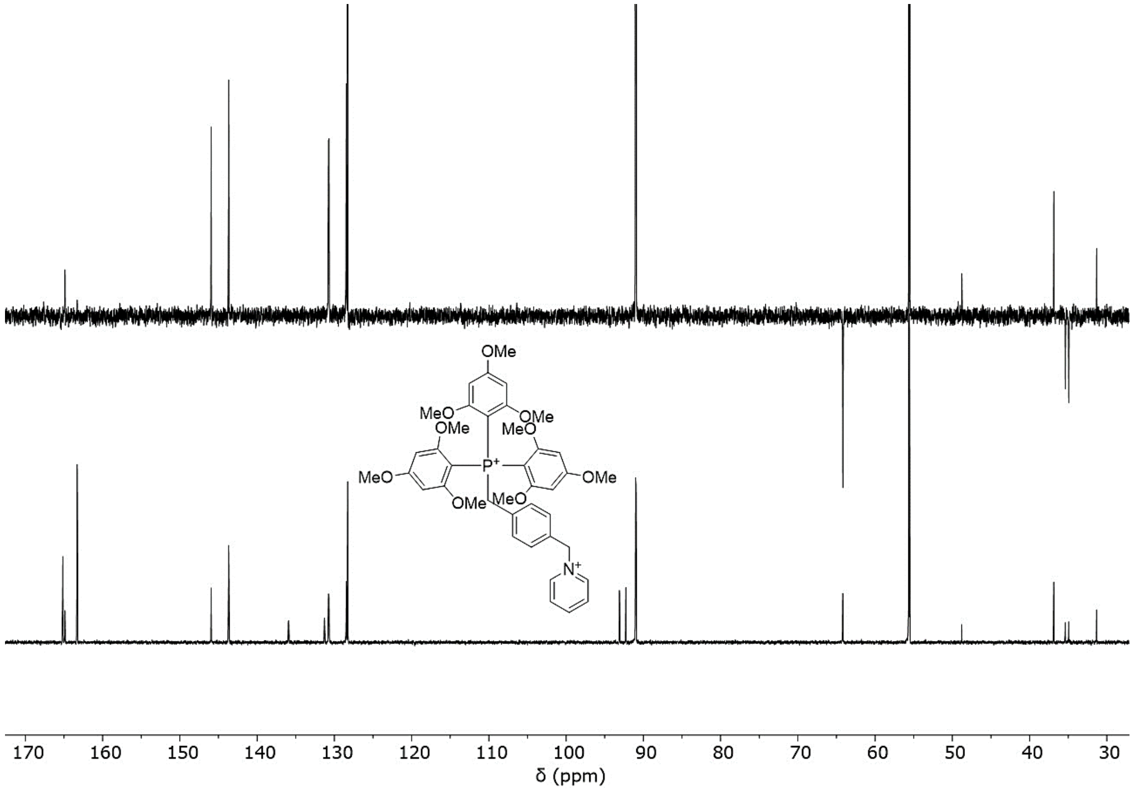

Figure S32: DEPT-135 NMR (126 MHz, D_2O) and $^{13}C\{^1H\}$ NMR (126 MHz, D_2O) spectrum of **5**·2Br.

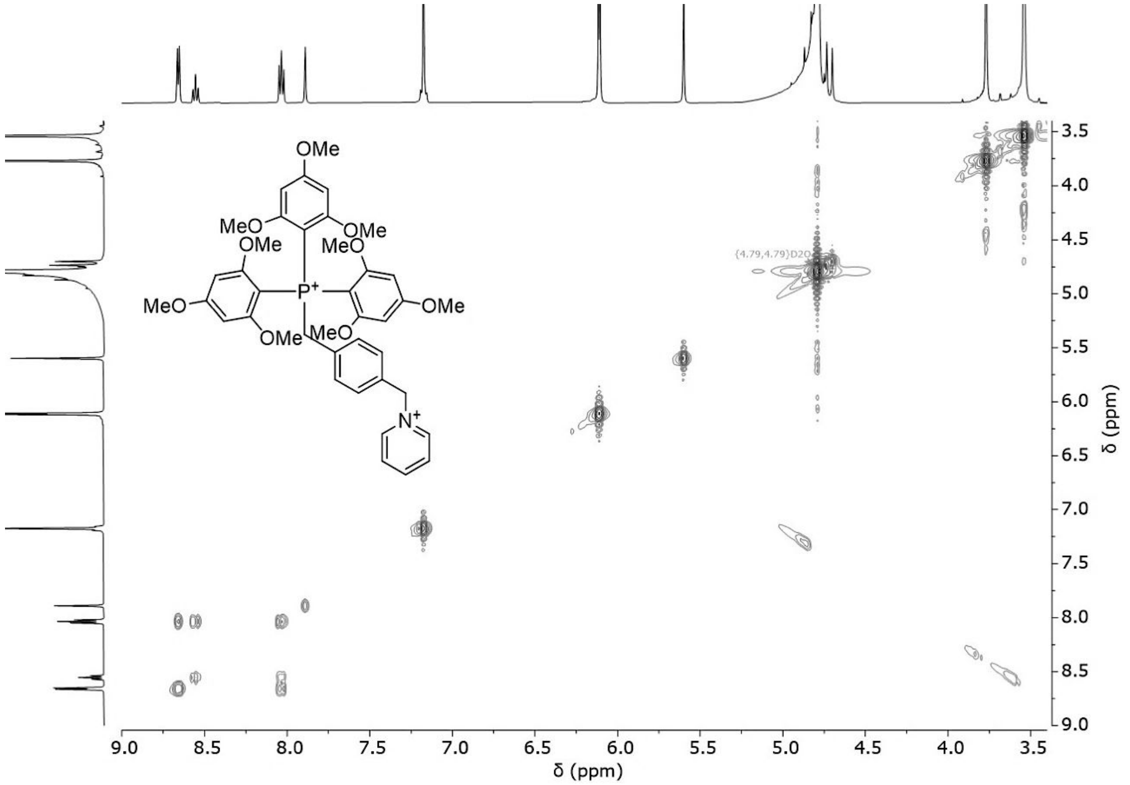

Figure S33: ^1H-^1H COSY (500 MHz, D_2O) spectrum of **5**·2Br.

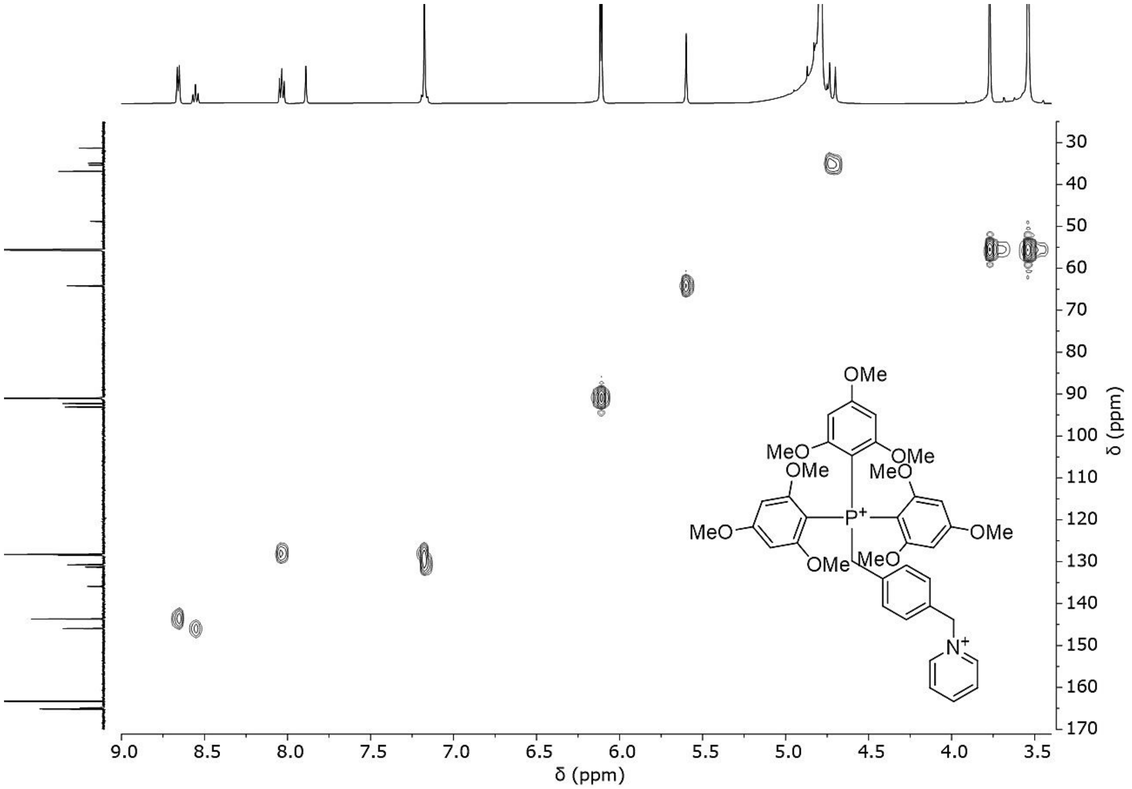

Figure S34: ^{1}H-^{13}C HSQC (500 MHz, D_2O) spectrum of **5**·2Br.

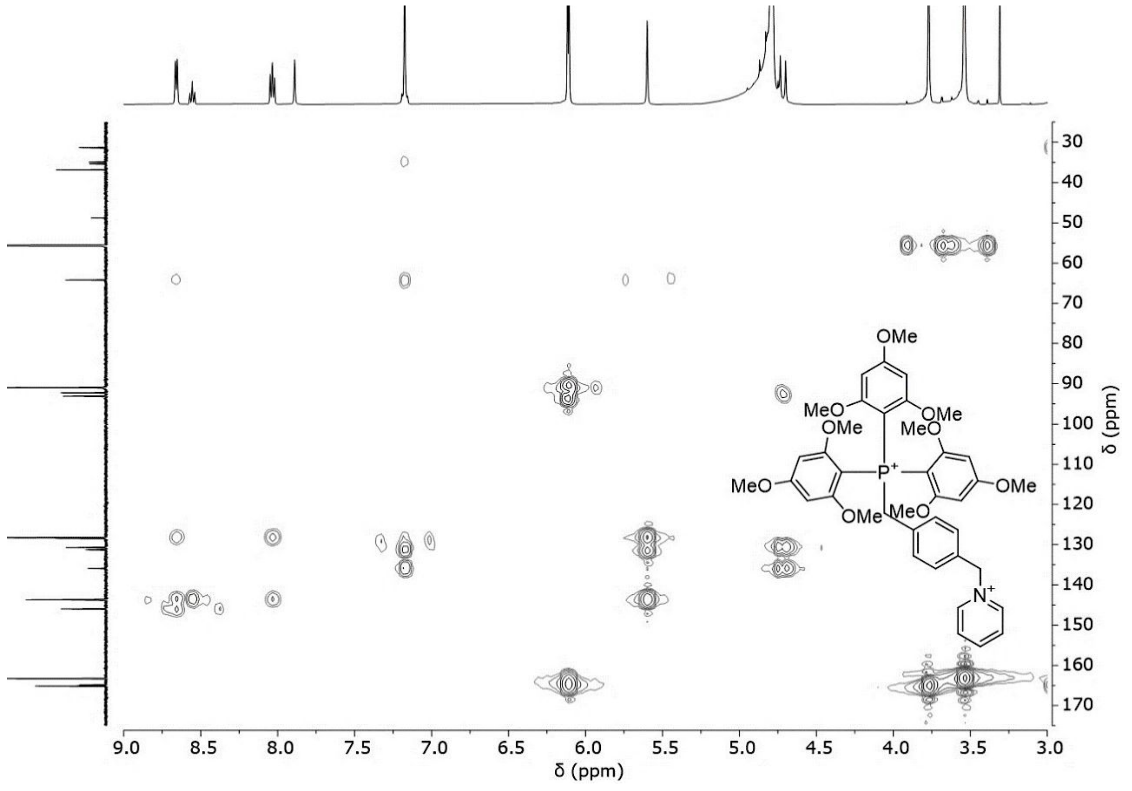

Figure S35: ^{1}H-^{13}C HMBC (500 MHz, D_2O) spectrum of **5**·2Br.

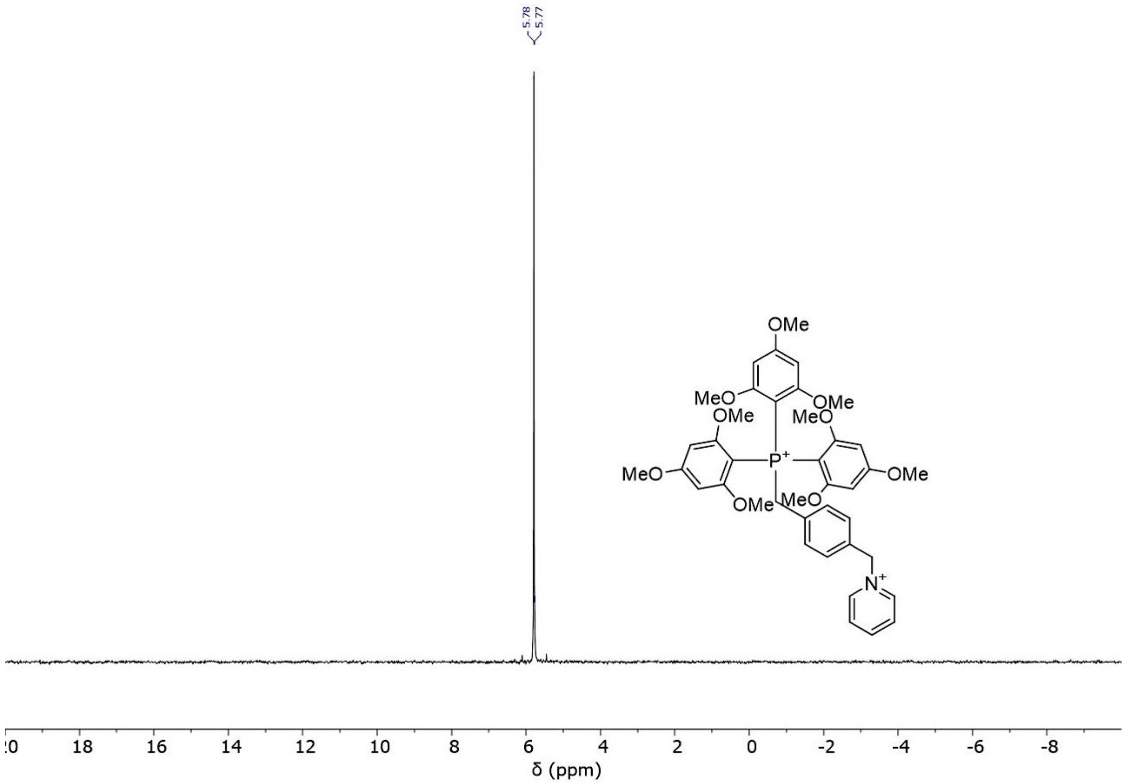

Figure S36: $^{31}P\{^{1}H\}$ NMR (162 MHz, D_2O) spectrum of **5**·2Br.

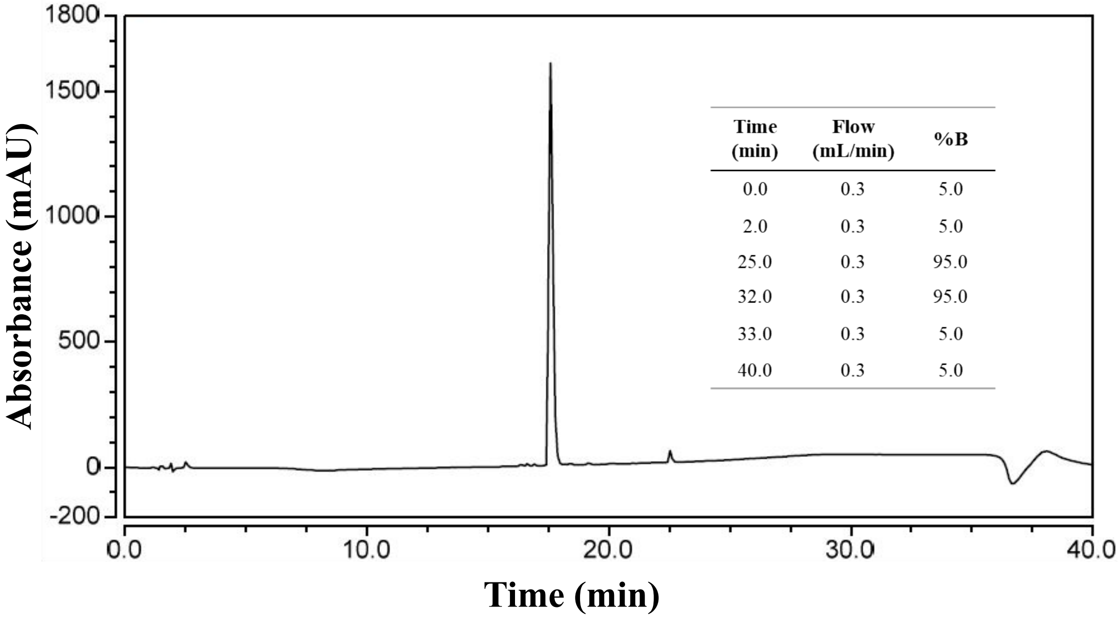

Time (min)	Flow (mL/min)	%B
0.0	0.3	5.0
2.0	0.3	5.0
25.0	0.3	95.0
32.0	0.3	95.0
33.0	0.3	5.0
40.0	0.3	5.0

Figure S37: HPLC chromatogram (220 nm) of $\mathbf{5}^{2+}$ at t_R = 17.6 min (Inset: separation method; A = H_2O + 0.04 % TFA, B = CH_3CN + 0.04 % TFA).

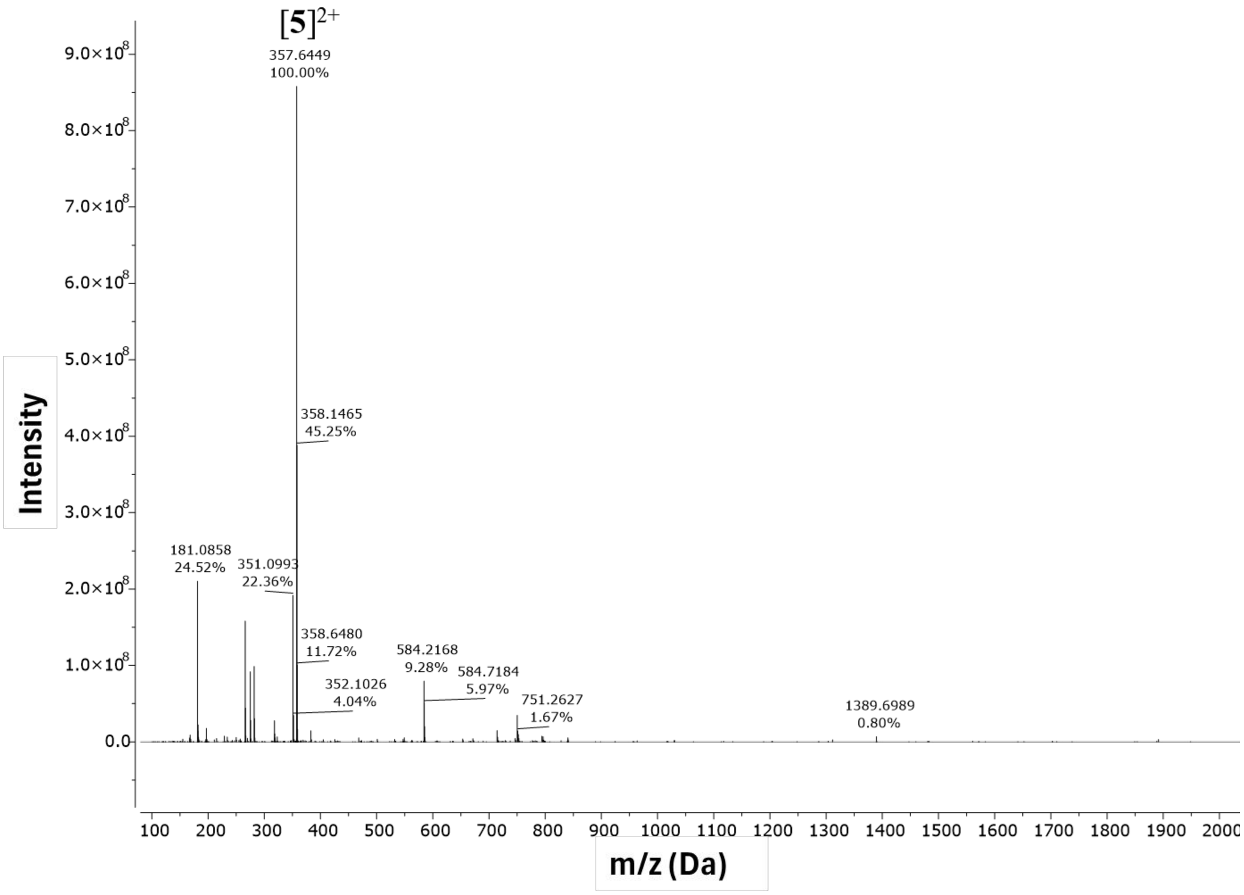

Figure S38: HRMS-ESI spectrum of **5**·2Br.

2.6. Synthesis and characterization data of 6·I

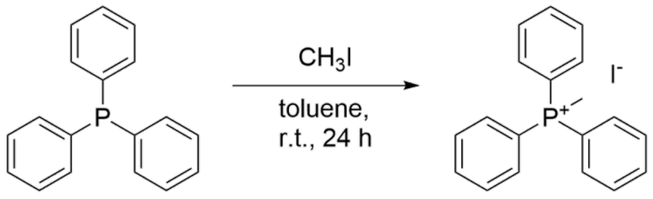

6·I

To a solution of triphenylphosphine (200 mg, 0.792 mmol, 1 eq) in toluene (50 mL) was added iodomethane (296 µL, 675 mg, 4.75 mmol, 6 eq). The mixture was left under stirring at room temperature for 24 hours. The precipitate was filtered under vacuum, washed with toluene (2 × 25 mL) and Et_2O (25 mL) and dried under the vacuum line to leave **6**·I as a white solid (242 mg, 76 %).

^{1}**H NMR** (500 MHz, D_2O) *δ (ppm)*: 7.84 – 7.76 (m, 3H), 7.71 – 7.61 (m, 12H), 2.82 (d, *J* = 14.0 Hz, 3H).^{13}**C{^{1}H} NMR** (101 MHz, D_2O) *δ (ppm)*: 134.9 (d, *J* = 3.1 Hz), 133.2 (d, *J* = 10.7 Hz), 129.9 (d, *J* = 12.9 Hz), 119.2 (d, *J* = 89.6 Hz), 8.0 (d, *J* = 58.3 Hz). ^{31}**P{^{1}H} NMR** (162 MHz, D_2O) *δ (ppm)*: 21.09. **HRMS (ESI)** m/z: [**6**]$^+$ Calcd for $C_{19}H_{18}P^+$ 277.1141; Found 277.1135.

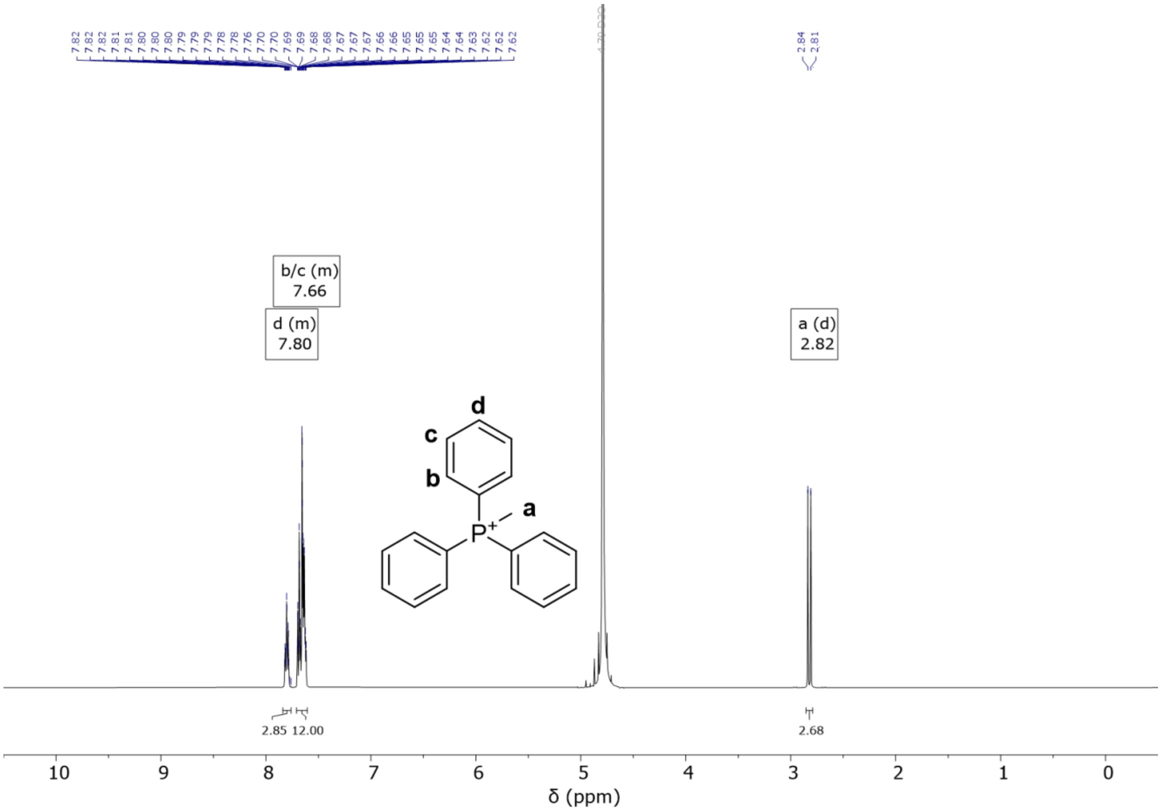

Figure S39: ^{1}H NMR (500 MHz, D_2O) spectrum of **6**·I.

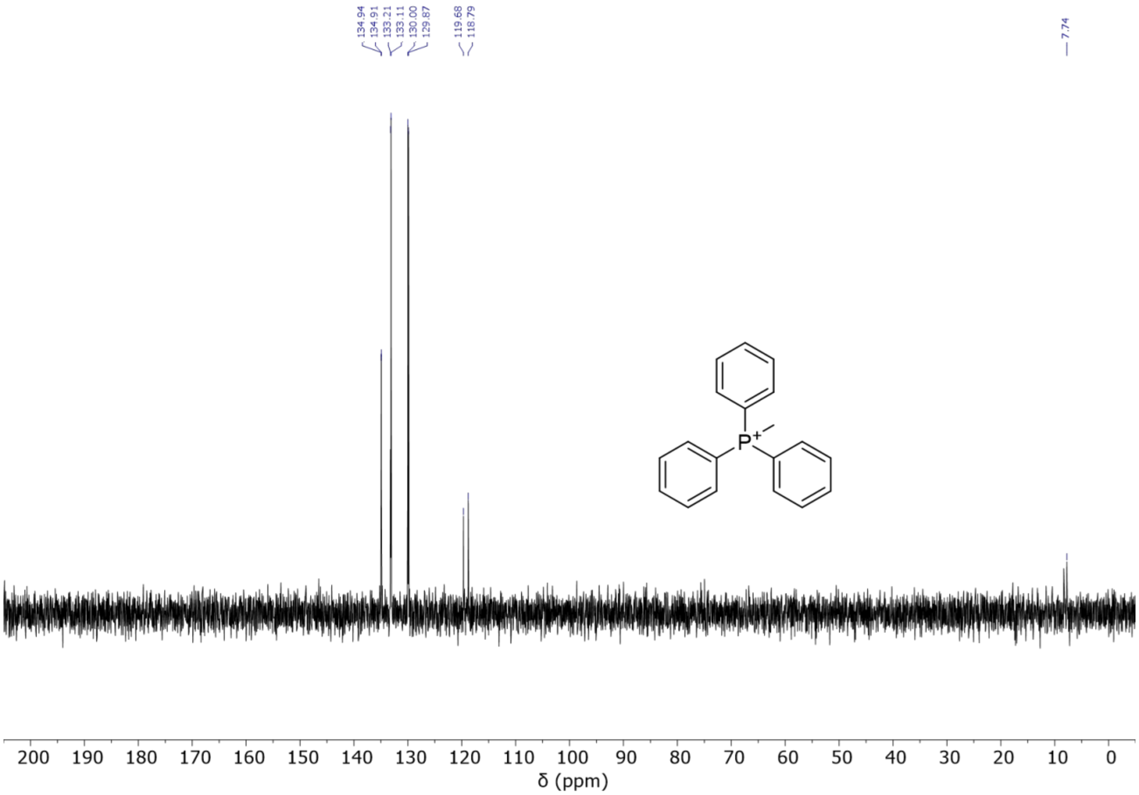

Figure S40: $^{13}C\{^1H\}$ NMR (101 MHz, D_2O) spectrum of **6**·I.

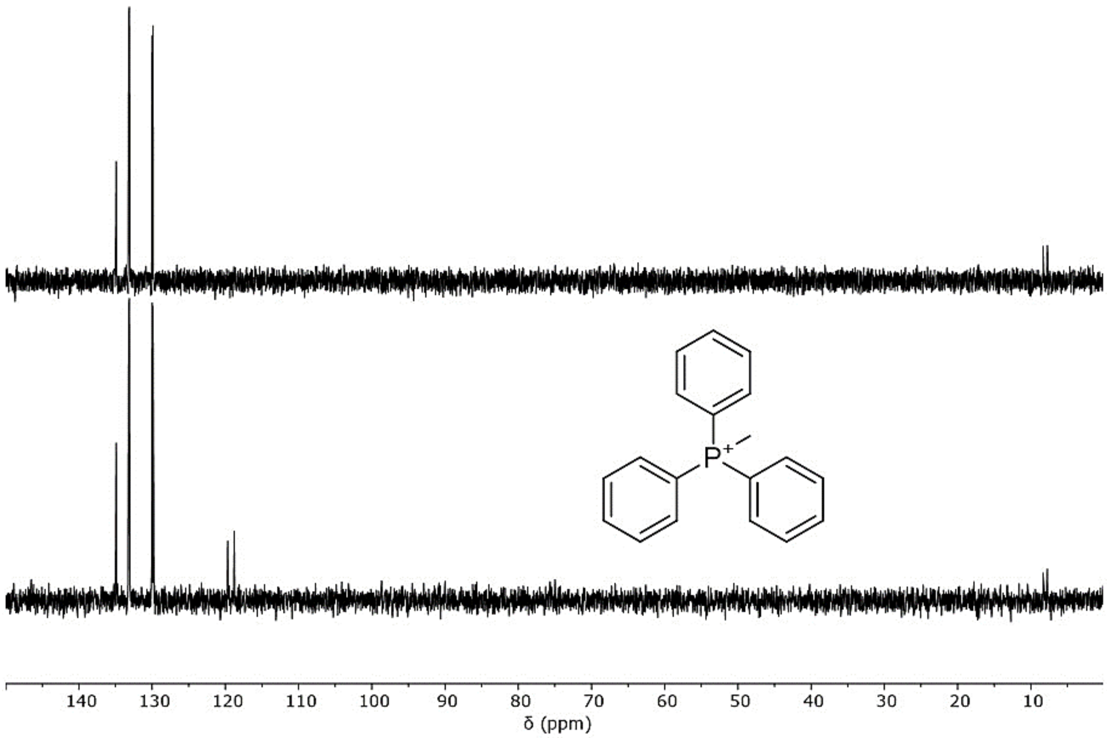

Figure S41: DEPT-135 NMR (101 MHz, D_2O) and $^{13}C\{^1H\}$ NMR (101 MHz, D_2O) spectrum of **6**·I.

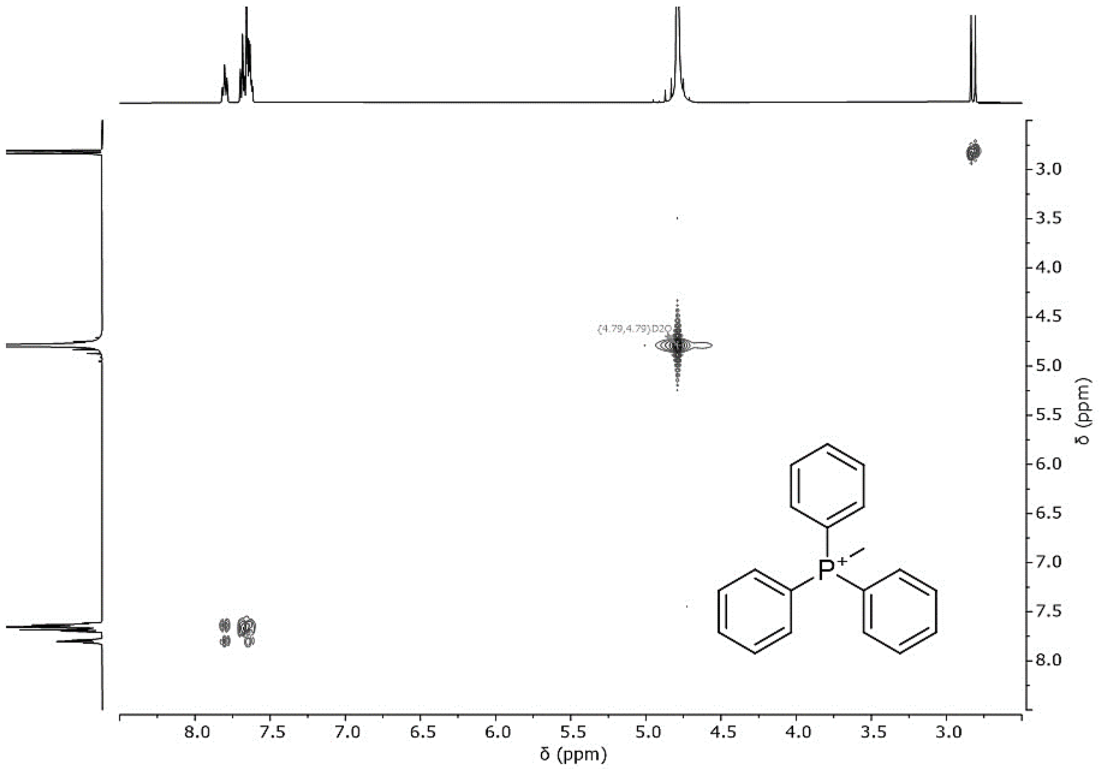

Figure S42: ^{1}H-^{1}H COSY (500 MHz, D_2O) spectrum of **6**·I.

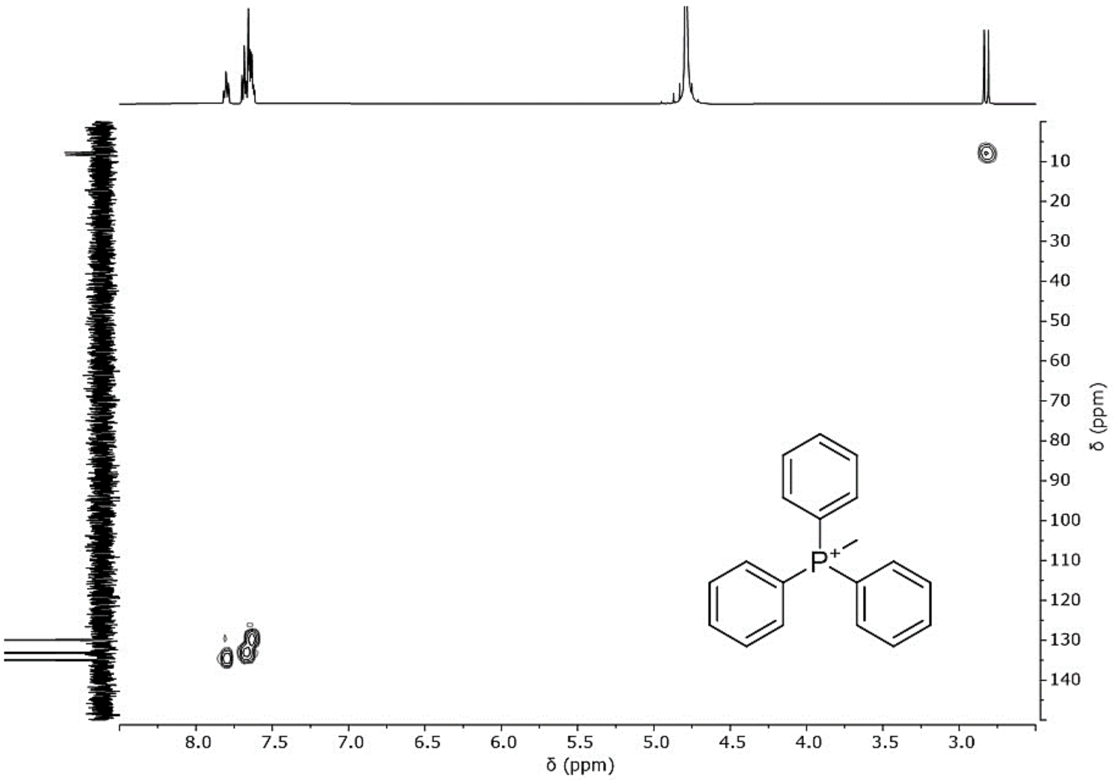

Figure S43: ^{1}H-^{13}C HSQC (500 MHz, D_2O) spectrum of **6**·I.

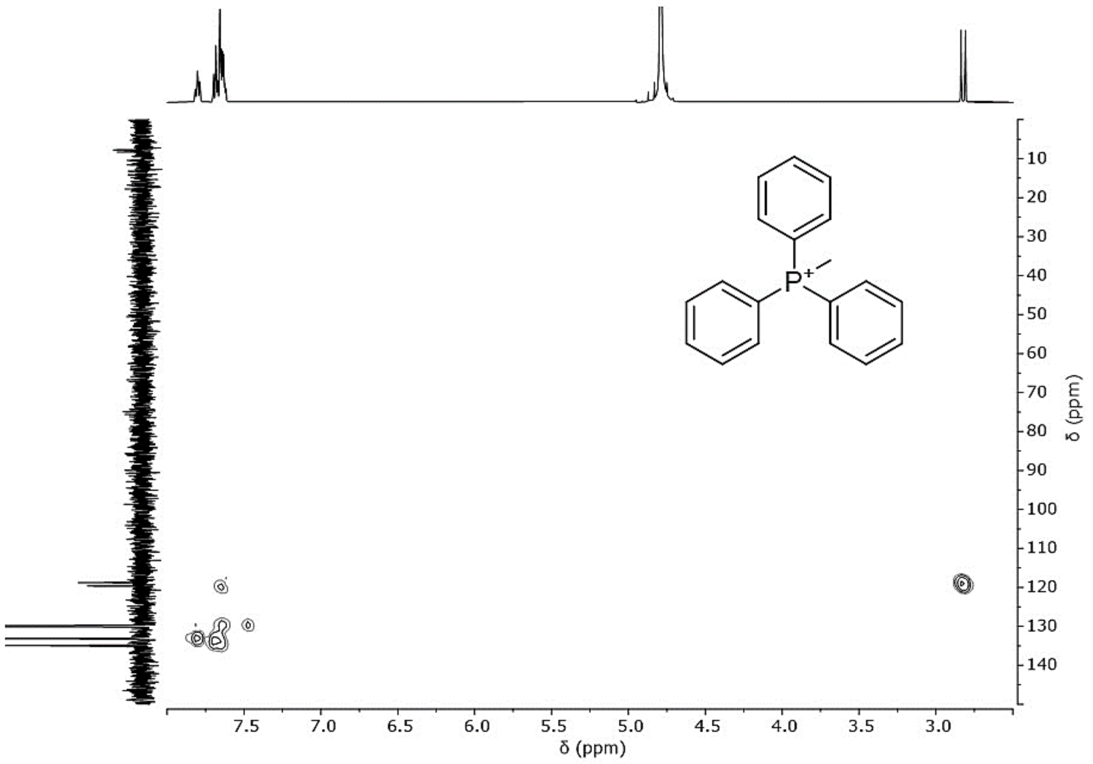

Figure S44: ^{1}H-^{13}C HMBC (500 MHz, D_2O) spectrum of **6**·I.

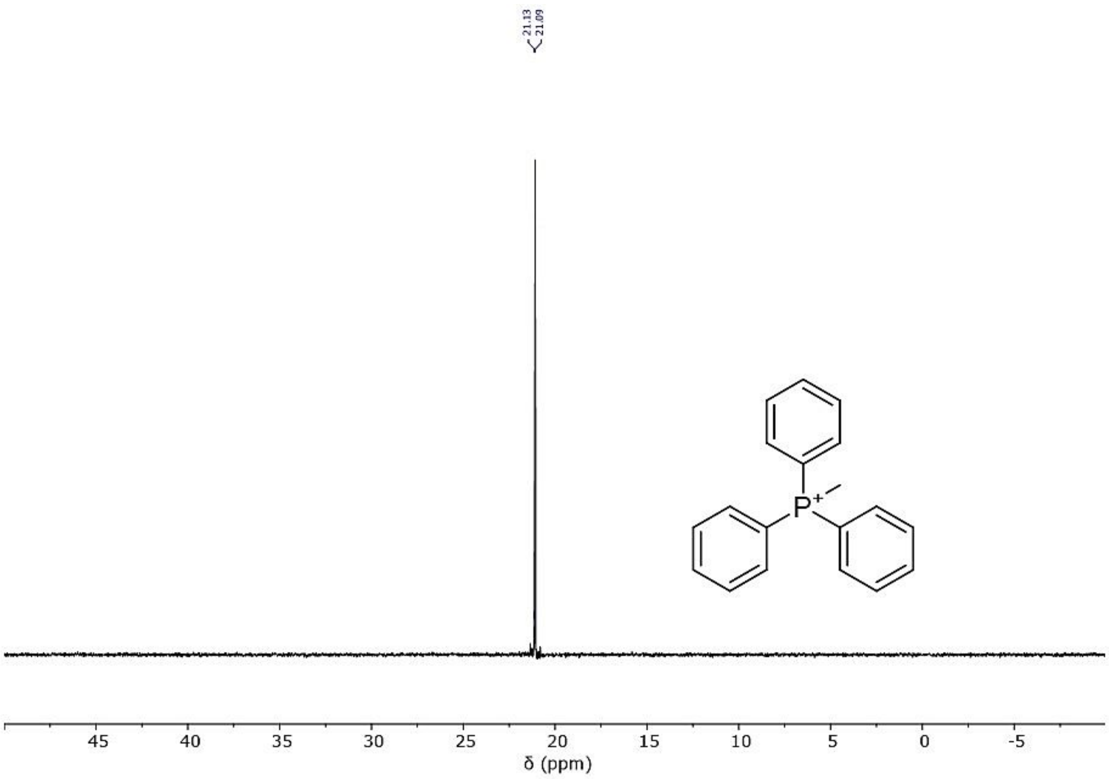

Figure S45: $^{31}P\{^{1}H\}$ NMR (162 MHz, D_2O) spectrum of **6**·I.

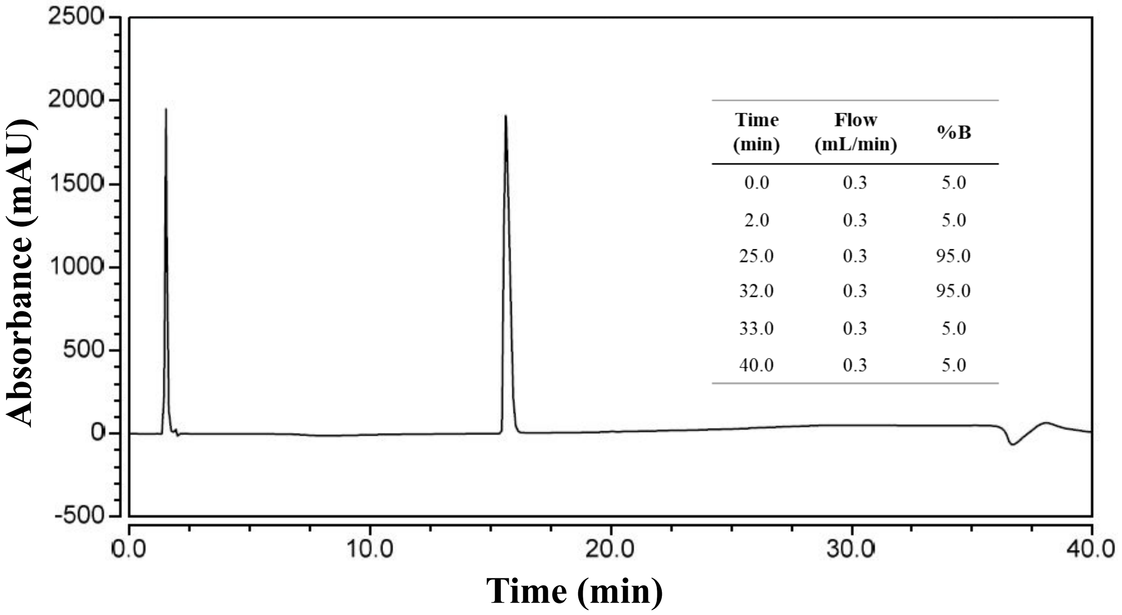

Time (min)	Flow (mL/min)	%B
0.0	0.3	5.0
2.0	0.3	5.0
25.0	0.3	95.0
32.0	0.3	95.0
33.0	0.3	5.0
40.0	0.3	5.0

Figure S46: HPLC chromatogram (220 nm) of **6**$^+$ at t_R = 15.6 min (Inset: separation method; A = H_2O + 0.04 % TFA, B = CH_3CN + 0.04 % TFA).

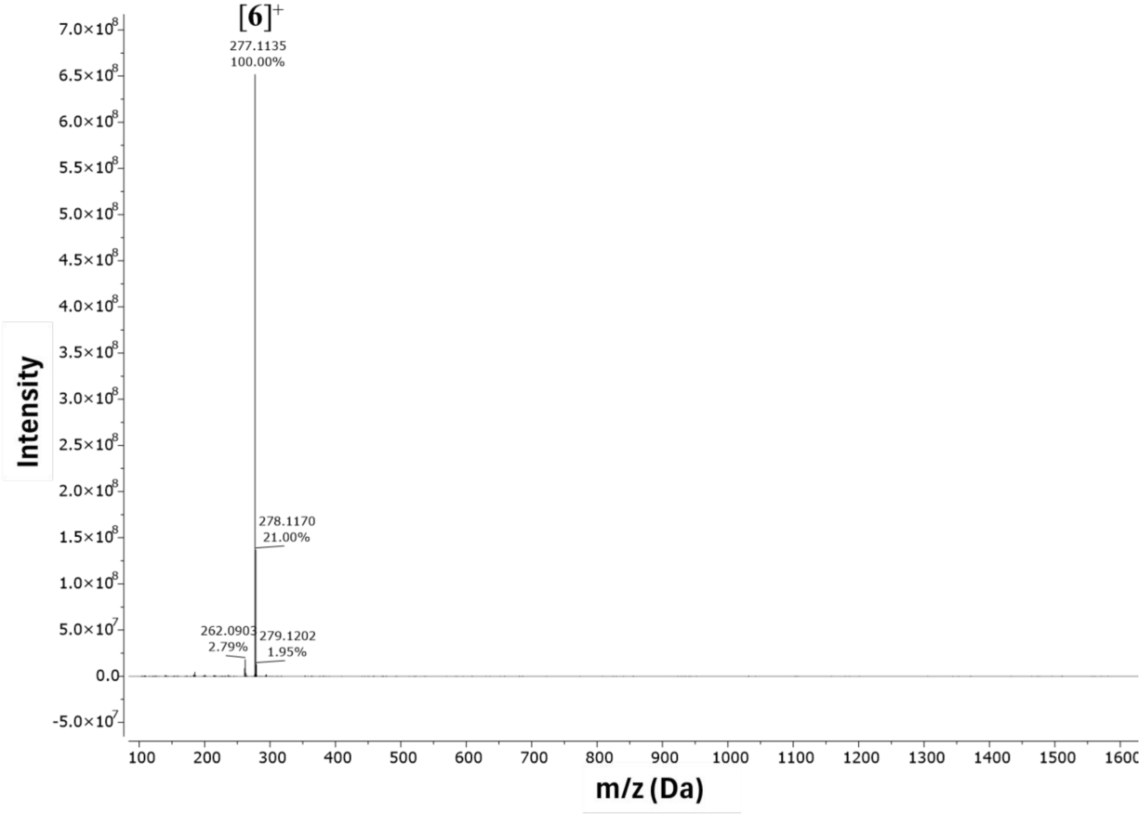

Figure S47: HRMS-ESI spectrum of **6**·I.

2.7. Synthesis and characterization data of 7·Br

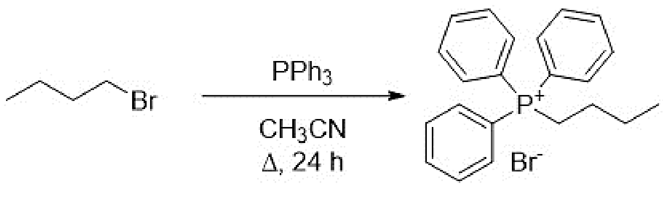

7·Br

To a solution of triphenylphosphine (689 mg, 2.63 mmol, 3 eq) in CH_3CN (15 mL) was added 94 µL of 1-bromobutane (120, 0.876 mmol, 1 eq). The mixture was stirred under reflux in a hot plate stirrer for 24 hours. The solvent was removed to leave a yellow residue which was subjected to flash chromatography (SiO_2, solvent 1: AcOEt, solvent 2: CH_3CN/H_2O MeOH: 4:1:1). The product containing fractions were combined and evaporated to yield **7**·Br as a yellow solid (127 mg, 35 %).

^{1}H NMR (500 MHz, D_2O) *δ (ppm)*: 7.83 (td, *J* = 7.5, 1.8 Hz, 3H), 7.78 – 7.70 (m, 6H), 7.67 (td, *J* = 7.8, 3.4 Hz, 6H), 3.30 – 3.20 (m, 2H), 1.70 – 1.58 (m, 2H), 1.49 (h, *J* = 7.4 Hz, 2H), 0.87 (t, *J* = 7.3 Hz, 3H). **^{13}C{^{1}H} NMR** (126 MHz, D_2O) *δ (ppm)*: 134.8 (d, *J* = 3.1 Hz), 133.5, 129.9, 118.3 (d, *J* = 86.7 Hz), 23.6 (d, *J* = 4.4 Hz), 23.2 (d, *J* = 16.8 Hz), 21.1 (d, *J* = 51.7 Hz), 12.5. **^{31}P{^{1}H} NMR** (162 MHz, D_2O) *δ (ppm)*: 23.29. **HRMS (ESI)** m/z: [**7**]$^+$ Calcd for $C_{22}H_{24}P^+$ 319.1611; Found 319.1610.

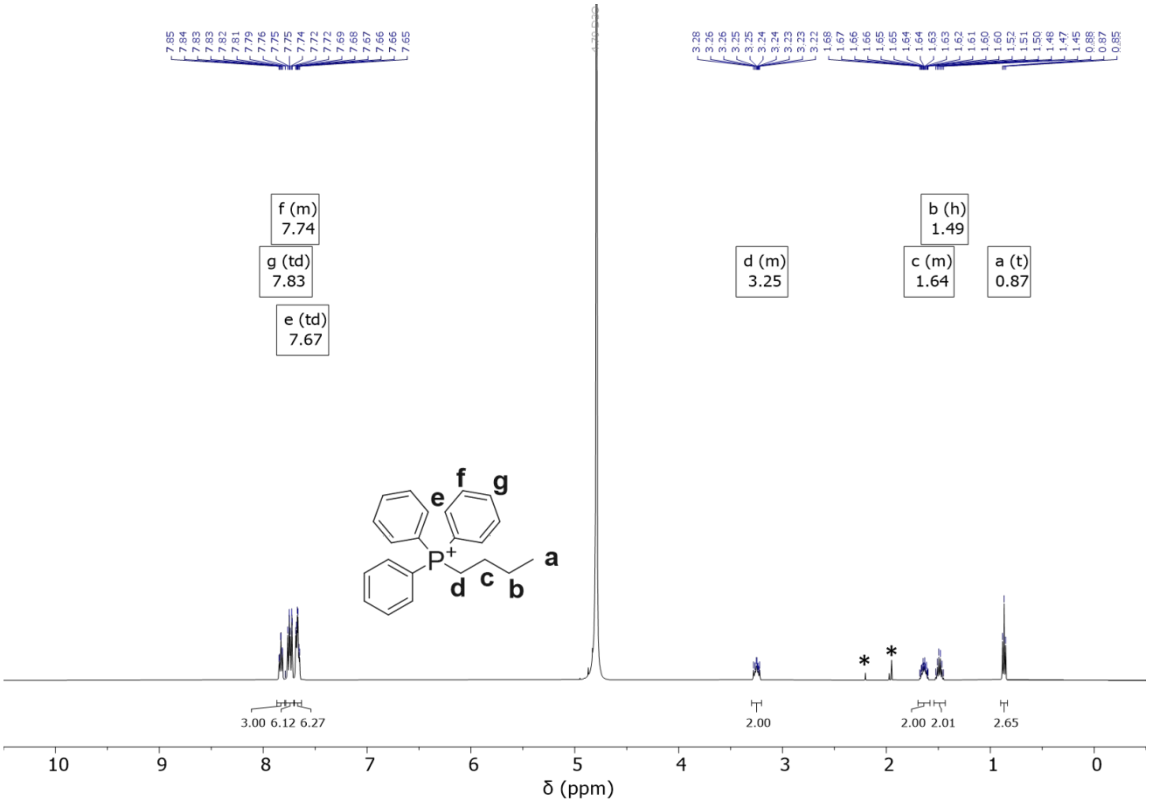

Figure S48: ^{1}H NMR (500 MHz, D_2O) spectrum of **7**·Br. Impurities are marked with *****: CH_3CN (1.98 ppm, s) and acetone (2.22 ppm, s).

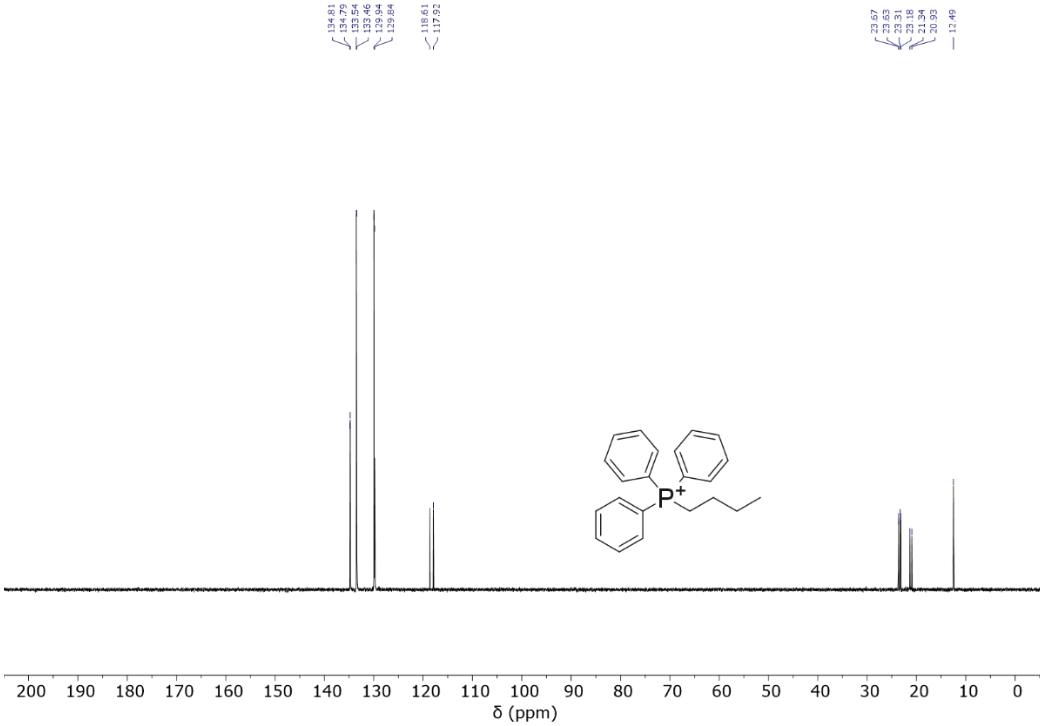

Figure S49: $^{13}C\{^1H\}$ NMR (101 MHz, D_2O) spectrum of **7**·Cl.

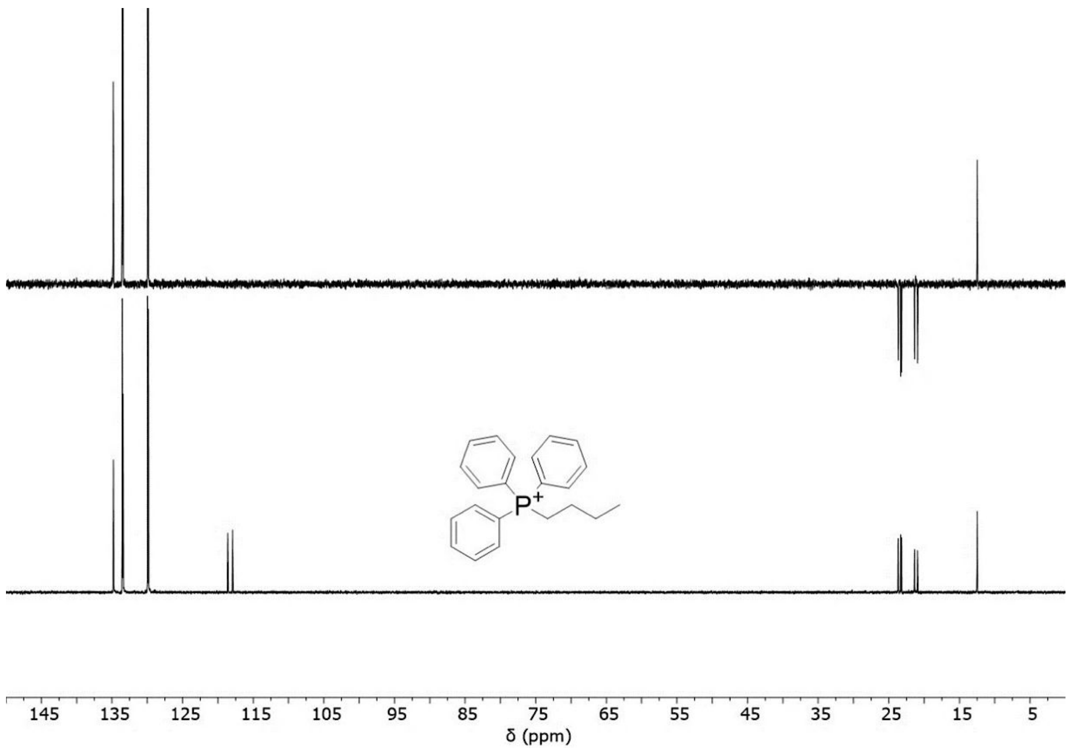

Figure S50: DEPT-135 (101 MHz, D_2O) spectrum (up) and $^{13}C\{^1H\}$ NMR (101 MHz, D_2O) spectrum (down) of **7**·Cl.

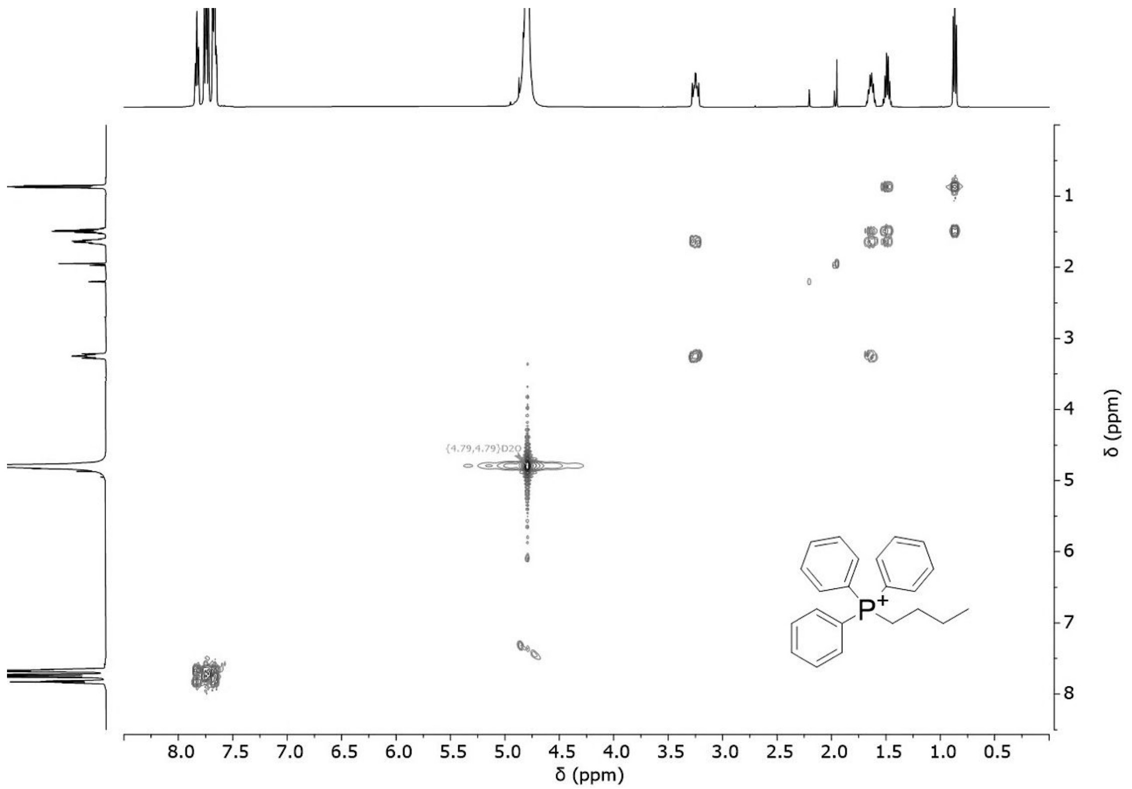

Figure S51: ^1H-^1H COSY (400 MHz, D_2O) spectrum of **7**·Br.

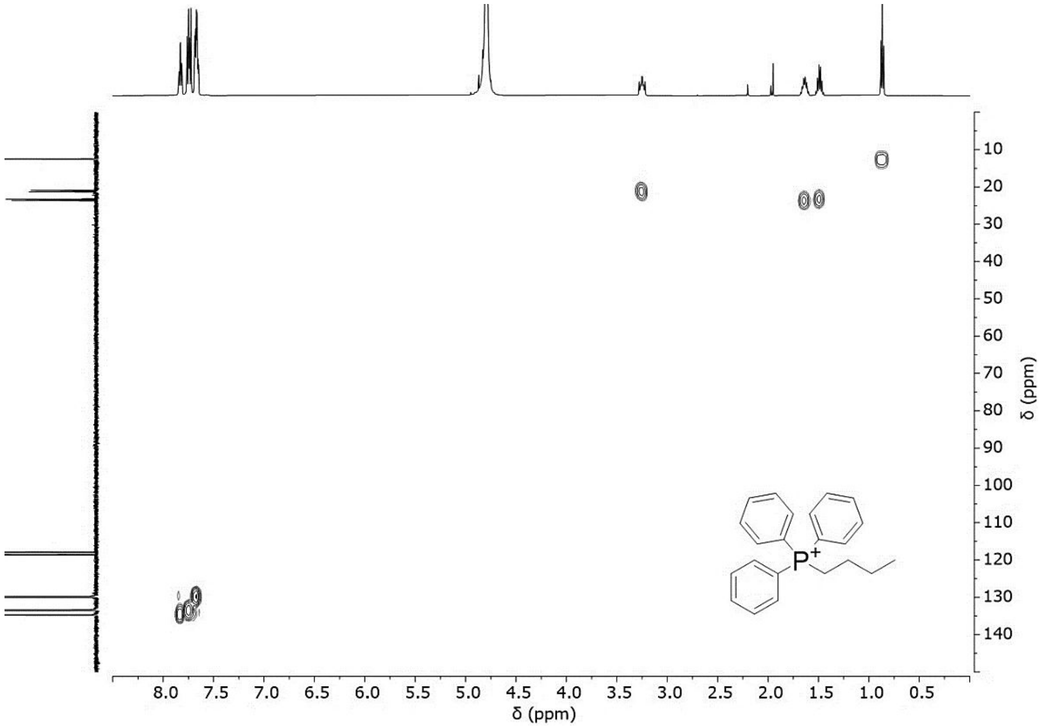

Figure S52: ^{1}H-^{13}C HSQC (400 MHz, D_2O) spectrum of **7**·Br.

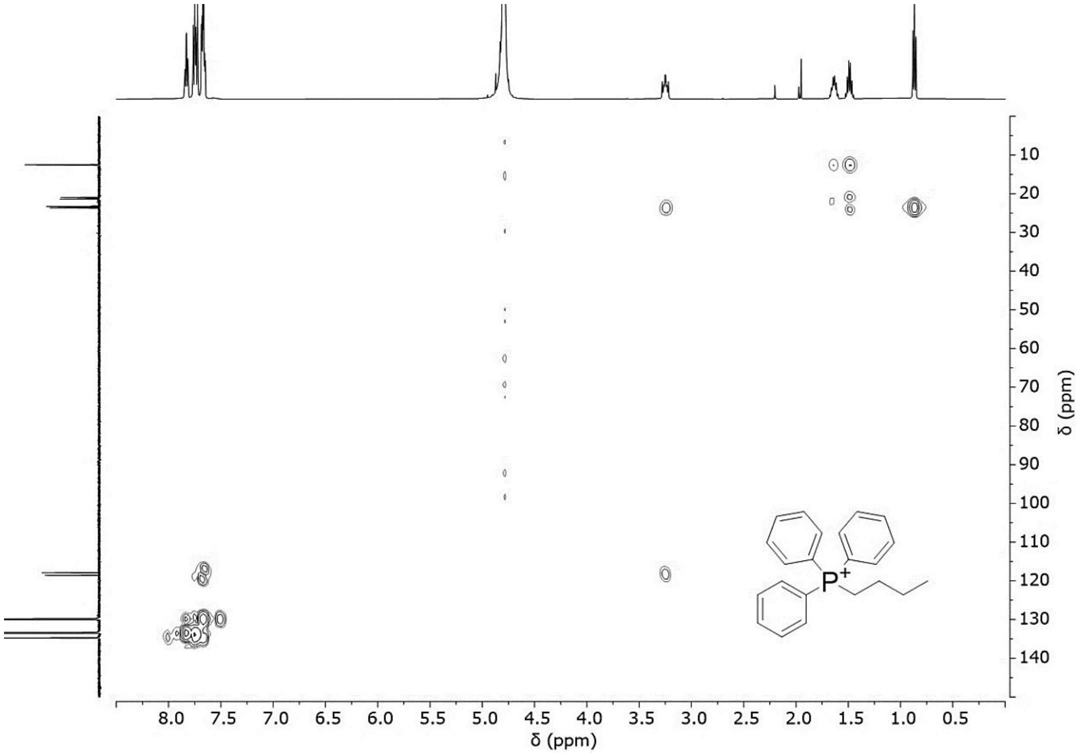

Figure S53: ^{1}H-^{13}C HMBC (400 MHz, D_2O) spectrum of **7**·Br.

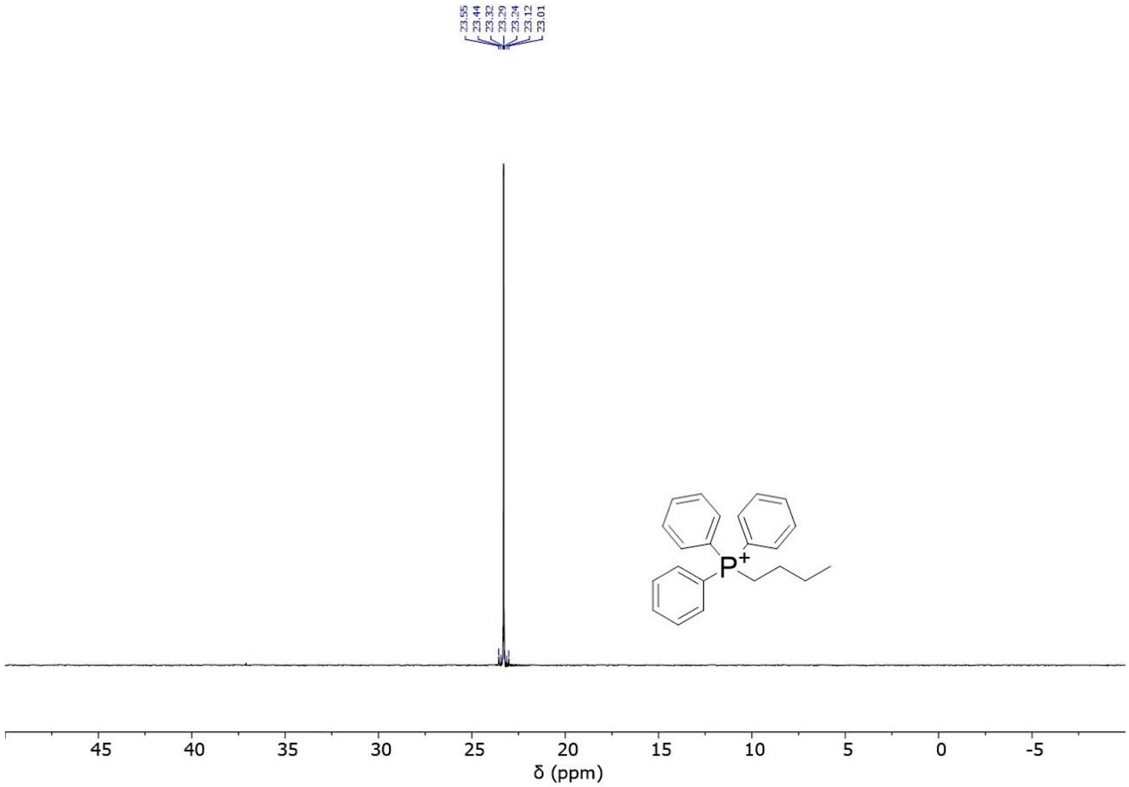

Figure S54: $^{31}P\{^{1}H\}$ NMR (162 MHz, D_2O) spectrum of **7**·Br.

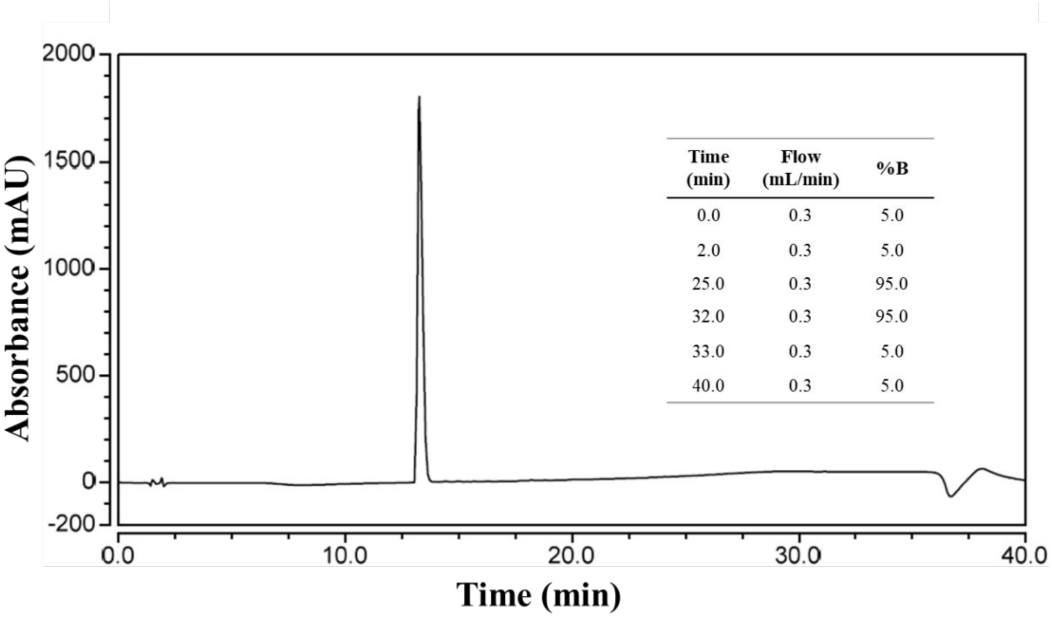

Figure S55: HPLC chromatogram (220 nm) of $\mathbf{6}^{+}$ at t_R = 13.3 min (Inset: separation method; A = H_2O + 0.04 % TFA, B = CH_3CN + 0.04 % TFA).

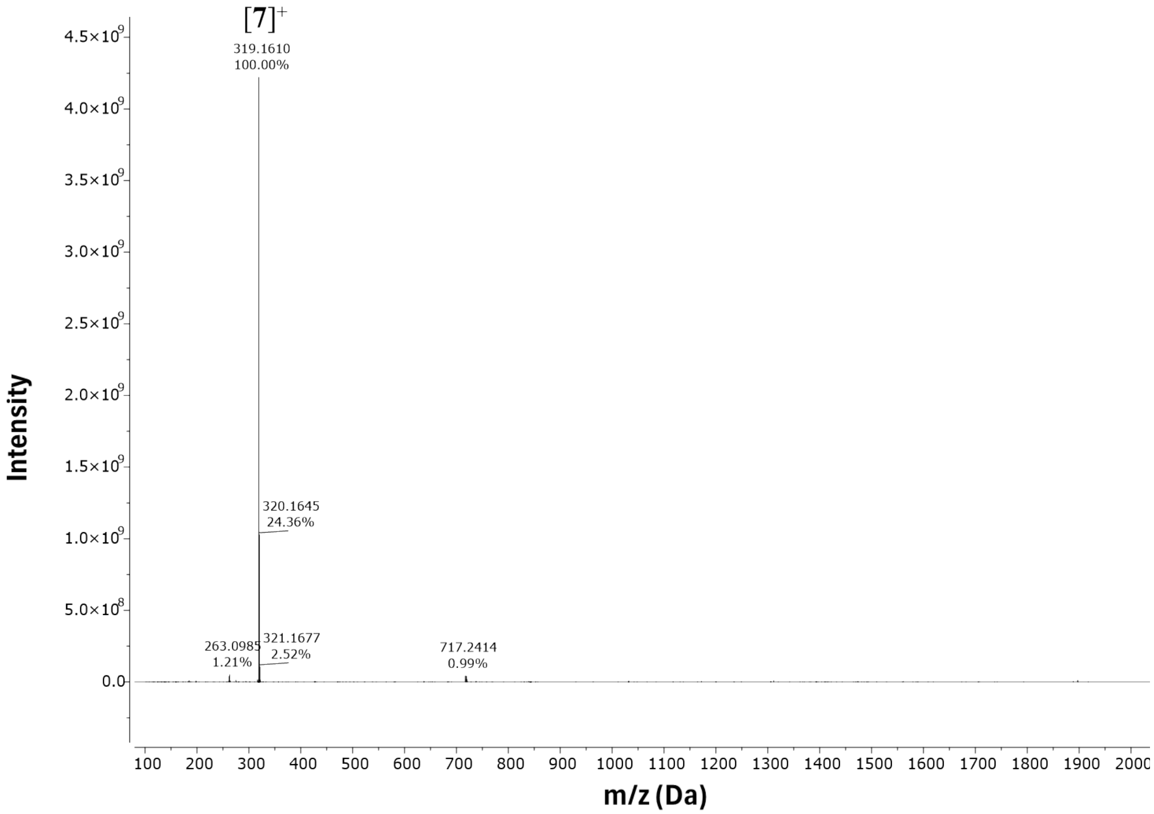

Figure S56: HRMS-ESI spectrum of **7**·Br.

2.8. Synthesis and characterization data of 8·Br

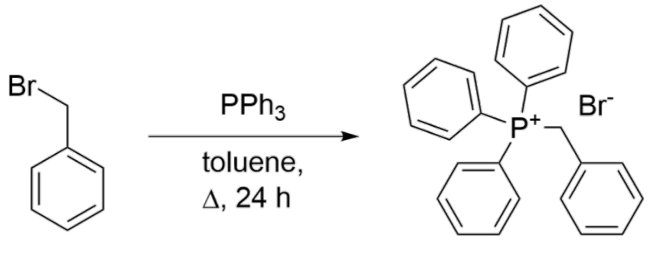

To a solution of triphenylphosphine (200 mg, 0.762 mmol, 1 eq) in toluene (30 mL) was added benzyl bromide (552 µL, 796 mg, 4.57 mmol, 6 eq). The mixture was stirred under reflux in a hot plate stirrer for 24 hours. The precipitate was filtered under vacuum, washed with toluene (2 × 25 mL), dried with Et_2O (25 mL) to yield **8**·Br as a white solid (279 mg, 84 %).

^{1}H NMR (500 MHz, D_2O) *δ (ppm)*: 7.89 – 7.82 (m, 3H), 7.70 – 7.59 (m, 13H), 7.38 – 7.32 (m, 1H), 7.23 (t, *J* = 7.6 Hz, 2H), 6.98 (dd, *J* = 7.8, 2.4 Hz, 2H), 4.75 (d, *J* = 18.2 Hz, 2H). **^{13}C{^{1}H} NMR** (126 MHz, D_2O) *δ (ppm)*: 135.0 (d, *J* = 3.2 Hz), 134.1 (d, *J* = 9.7 Hz), 130.9 (d, *J* = 5.3 Hz), 129.8, 128.9 (d, *J* = 3.2 Hz), 128.5 (d, *J* = 3.7 Hz), 127.2 (d, *J* = 8.3 Hz), 117.2 (d, *J* = 86.6 Hz), 29.6 (d, *J* = 48.7 Hz). **^{31}P{^{1}H} NMR** (162 MHz, D_2O) *δ (ppm)*: 22.32. **HRMS (ESI)** m/z: [**8**]$^+$ Calcd for $C_{25}H_{22}P^+$ 353.1454; Found 353.1450.

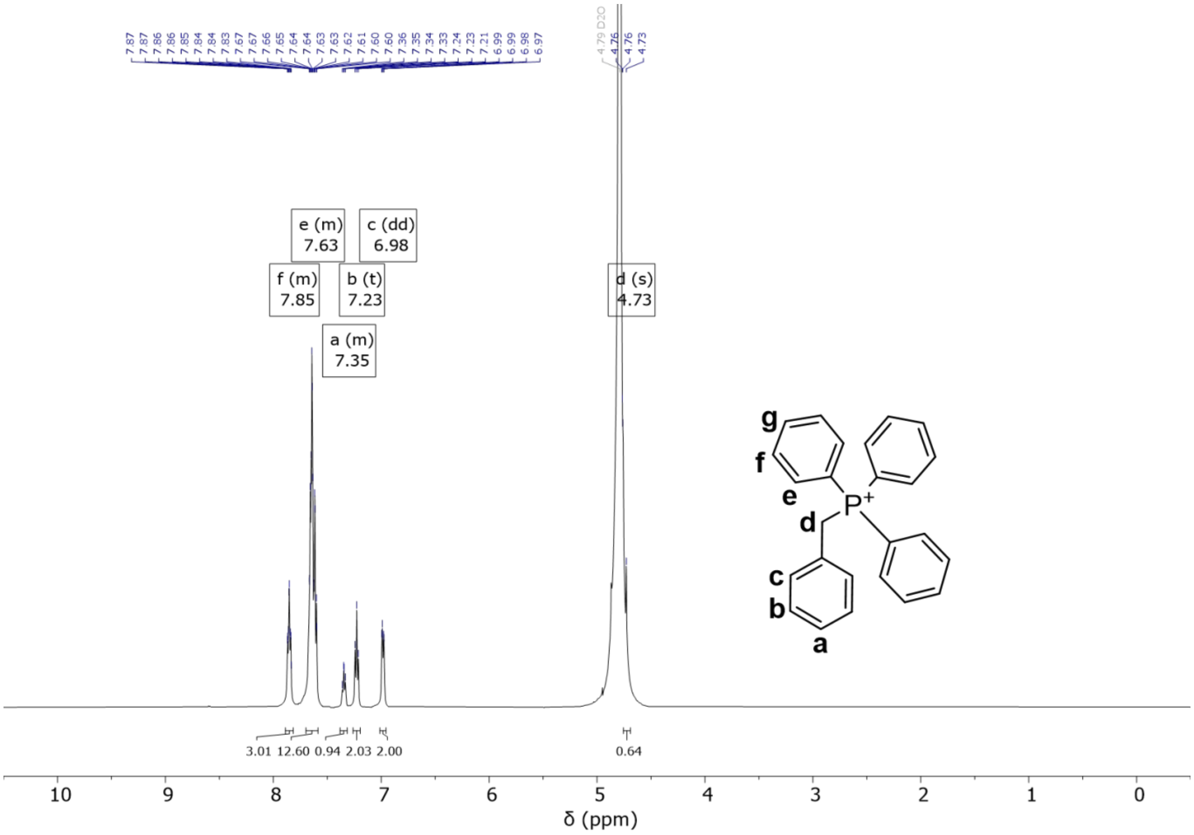

Figure S57: ^{1}H NMR (500 MHz, D_2O) spectrum of **8**·Br.

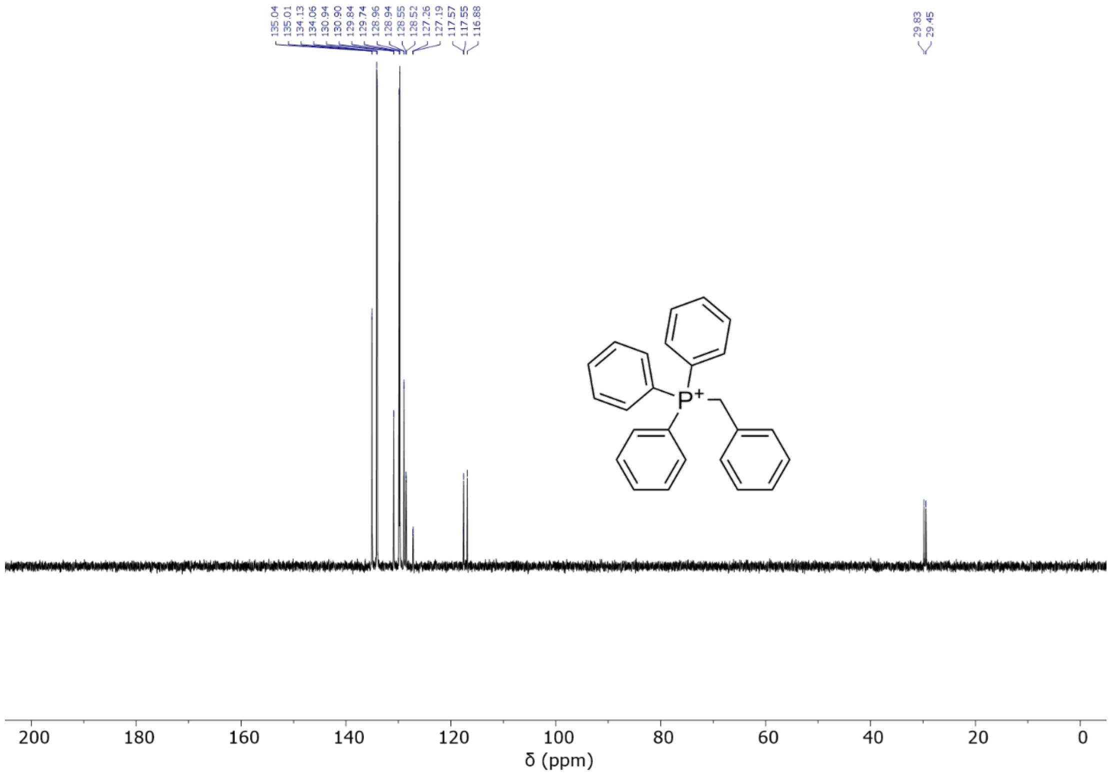

Figure S58: $^{13}C\{^1H\}$ NMR (126 MHz, D_2O) spectrum of **8**·Br.

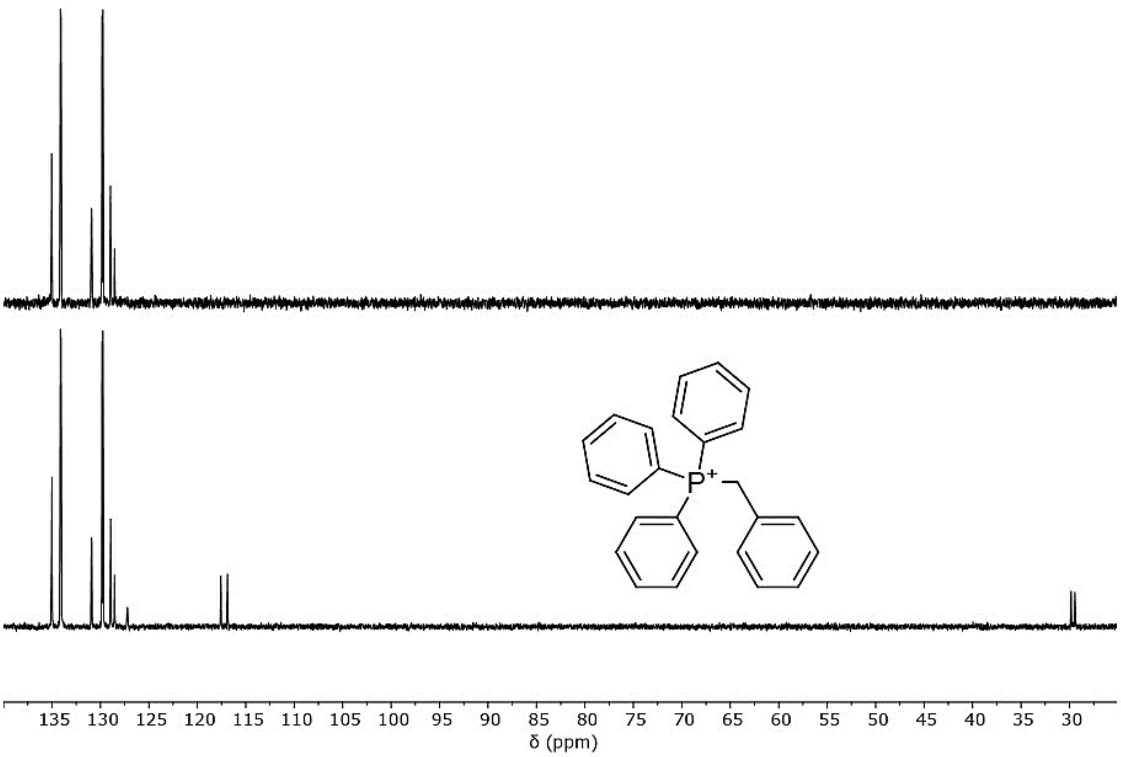

Figure S59: DEPT-135 NMR (126 MHz, D_2O) and $^{13}C\{^1H\}$ NMR (126 MHz, D_2O) spectrum of **8**·Br.

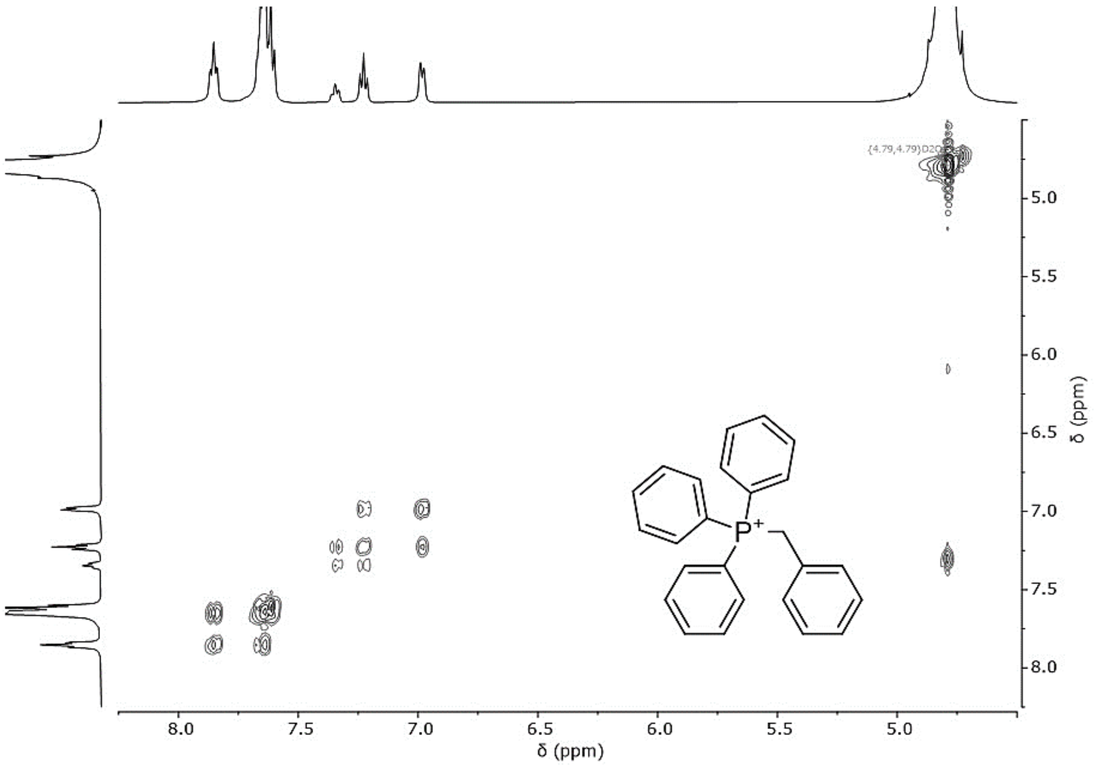

Figure S60: ^{1}H-^{1}H COSY (500 MHz, D_2O) spectrum of **8**·Br.

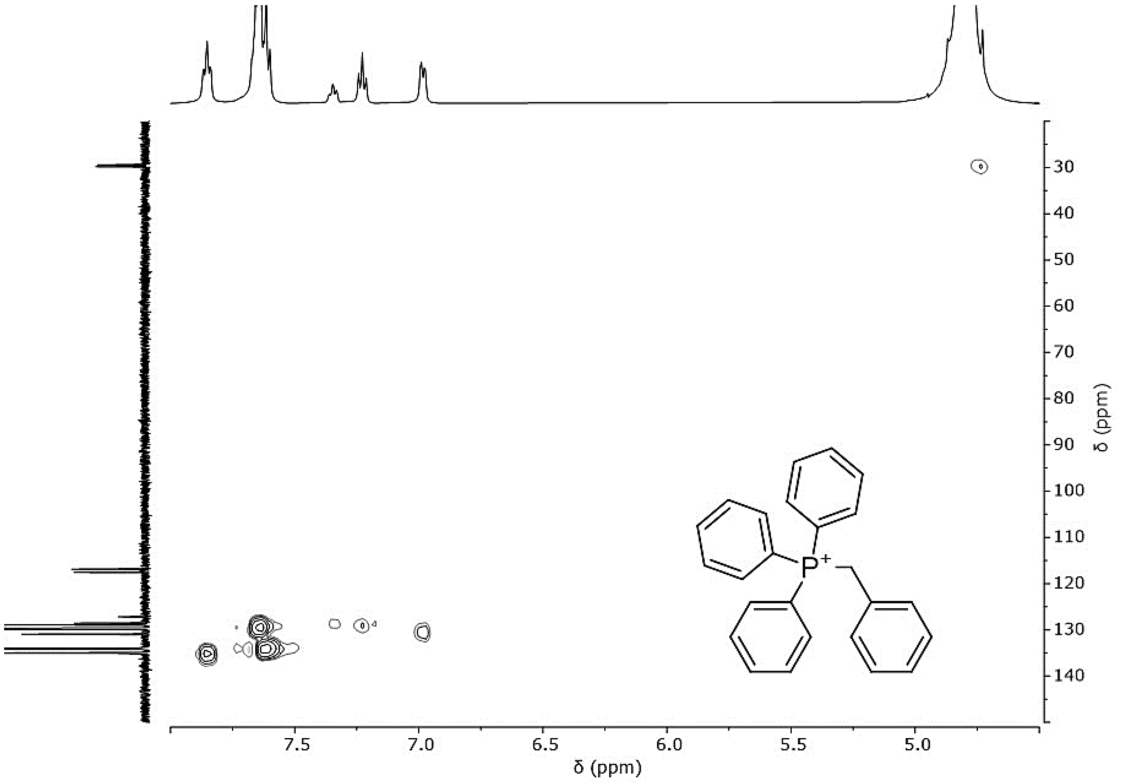

Figure S61: ^{1}H-^{13}C HSQC (500 MHz, D_2O) spectrum of **8**·Br.

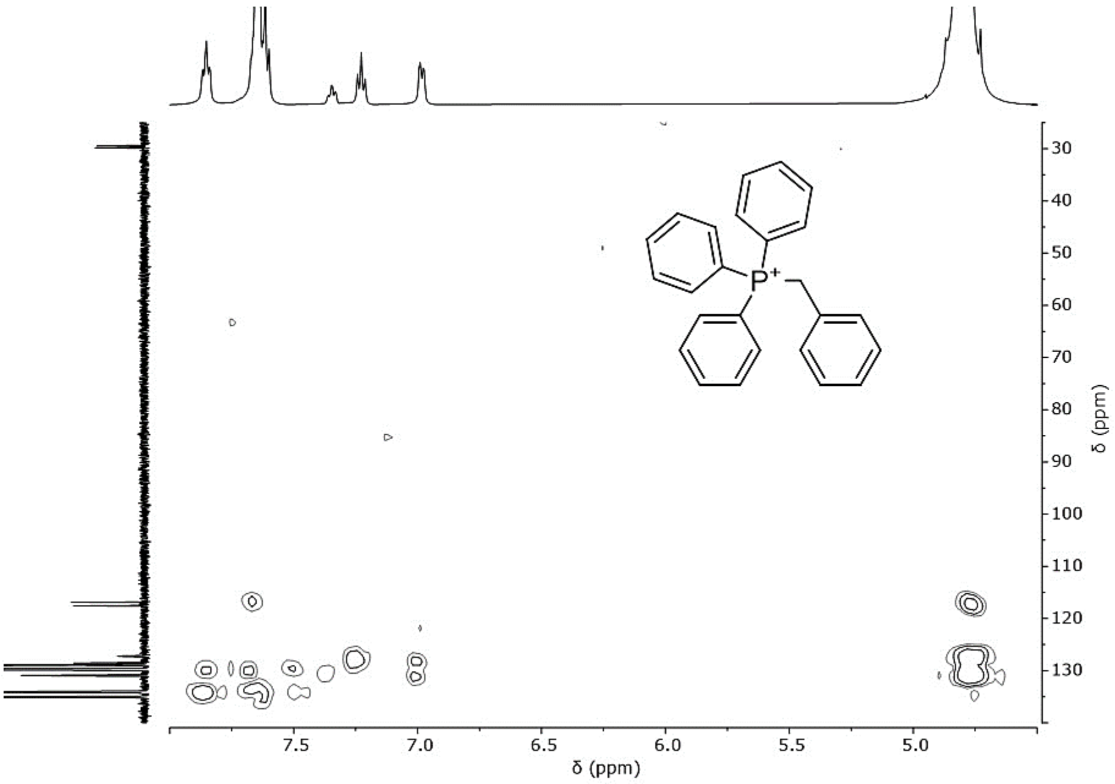

Figure S62: ^{1}H-^{13}C HMBC (500 MHz, D_2O) spectrum of **8**·Br.

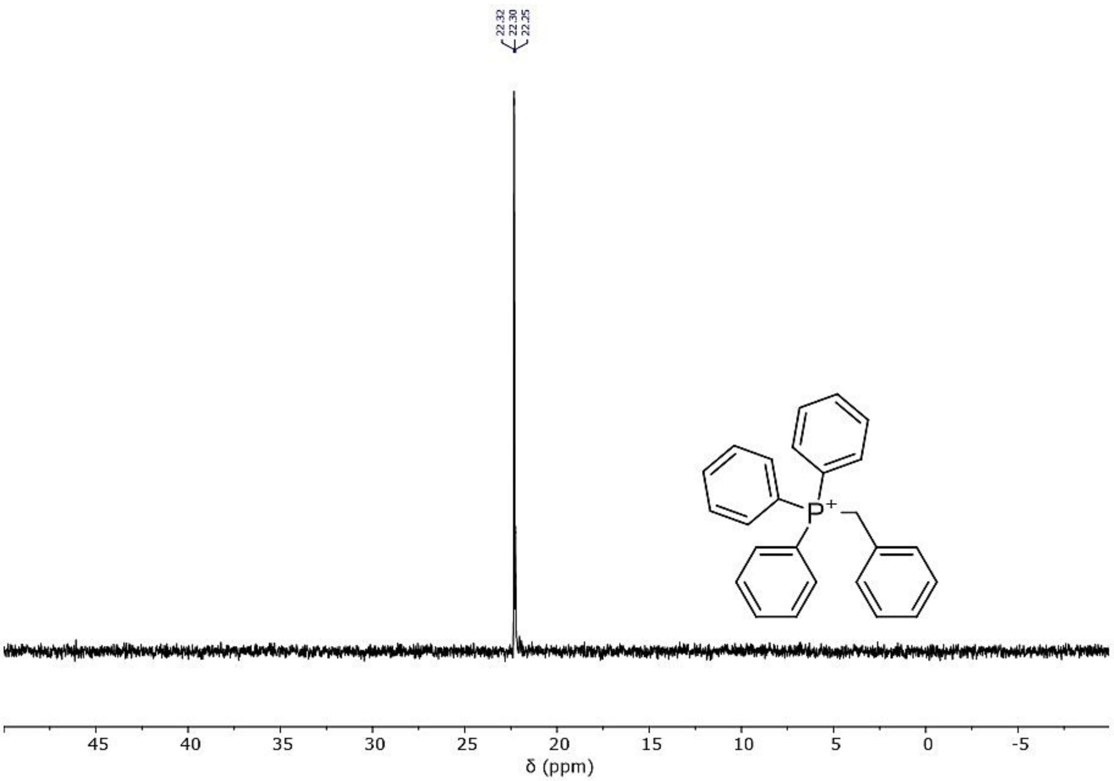

Figure S63: $^{31}P\{^{1}H\}$ NMR (162 MHz, D_2O) spectrum of **8**·Br.

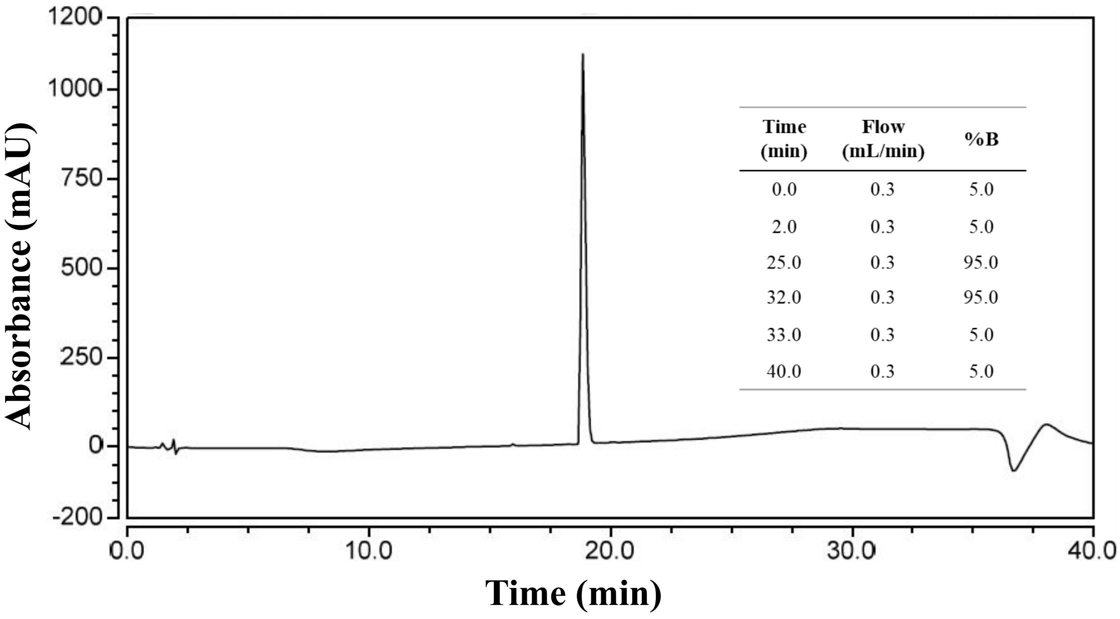

Figure S64: HPLC chromatogram (220 nm) of **8**$^+$ at t_R = 18.9 min (Inset: separation method; A = H_2O + 0.04 % TFA, B = CH_3CN + 0.04 % TFA).

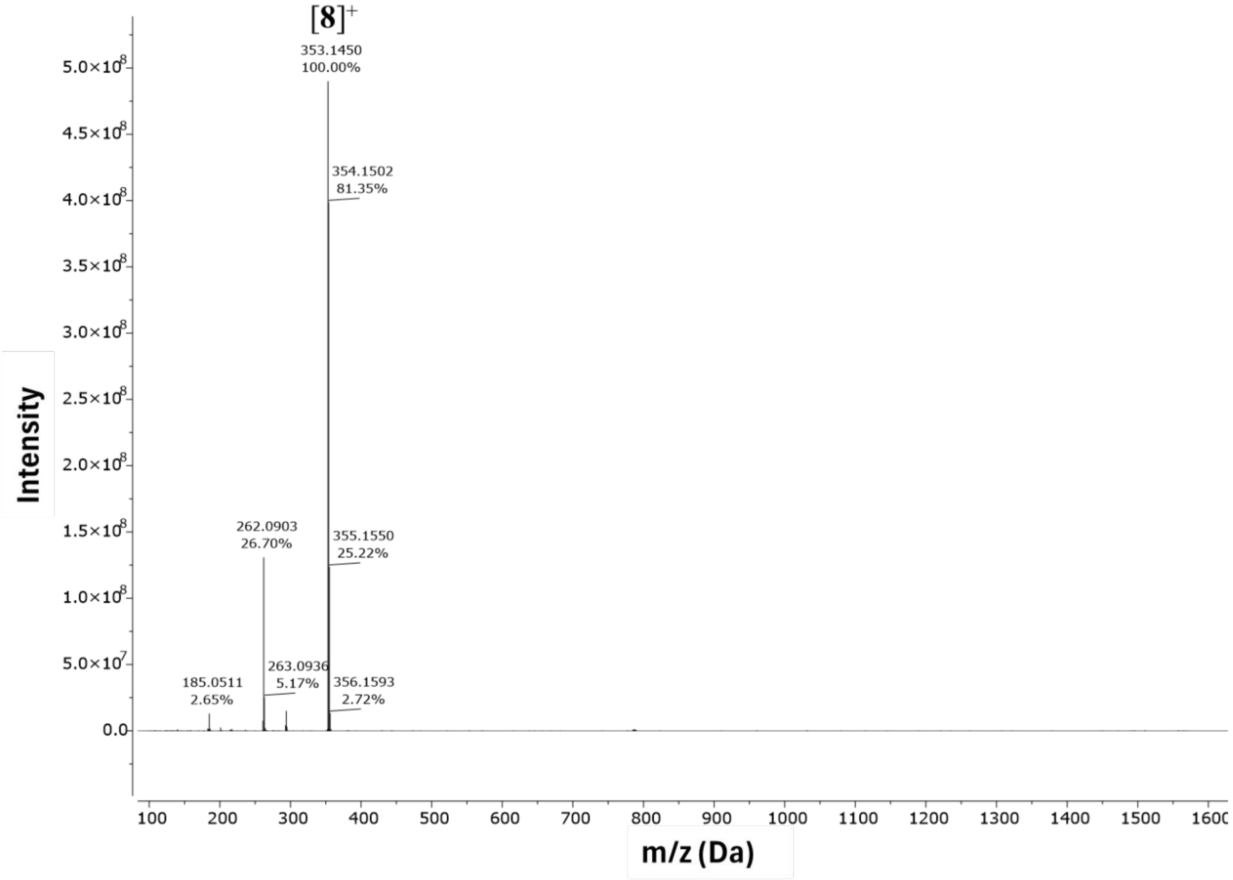

Figure S65: HRMS-ESI spectrum of **8**·Br.

3. HOST-GUEST CHEMISTRY

3.1. NMR study of the interaction of 2·2Br and CB[8]

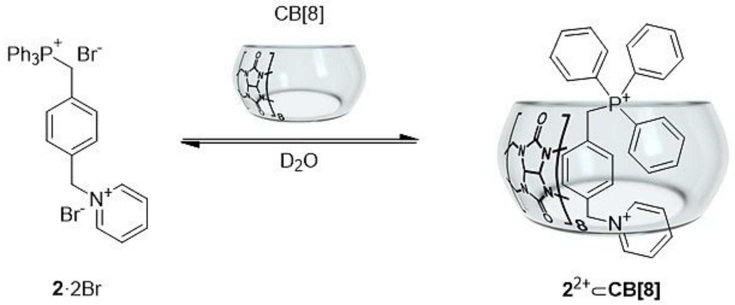

Firstly, a 2 mM stock solution of **2**·2Br in D_2O was prepared. To 2 mL of this solution was added CB[8] in excess and the mixture was heated and sonicated until saturation. The suspension was filtered (nylon, 0.2 µm) to obtain a solution of stoichiometry 1:1 of **2**·Br and CB[8].

^{1}H NMR (500 MHz, D_2O) *δ (ppm)*: 8.76 (tt, *J* = 7.3, 1.9 Hz, 1H), 8.34 – 8.25 (m, 4H), 8.00 – 7.92 (m, 4H), 7.84 (dt, *J* = 5.4, 2.8 Hz, 6H), 6.70 (dd, *J* = 12.7, 7.7 Hz, 2H), 6.43 (d, *J* = 8.0 Hz, 2H), 6.39 (dd, *J* = 8.4, 2.5 Hz, 2H), 6.20 (td, *J* = 7.8, 3.2 Hz, 2H), 5.91 (s, 4H), 5.82 (t, *J* = 7.5 Hz, 1H), 5.65 (d, *J* = 15.2 Hz, 8H), 5.50 (s, 16H), 5.23 (s, 2H), 4.58 (d, *J* = 12.8 Hz, 2H), 4.26 (d, *J* = 15.3 Hz, 8H), 4.14 (d, *J* = 15.2 Hz, 8H). **^{13}C{^{1}H} NMR** (101 MHz, D_2O) *δ(ppm)*: 156.8, 156.3, 147.5, 142.5, 134.6, 134.3, 133.5, 133.4, 131.4, 131.3, 130.5, 130.4, 129.8, 127.4, 126.8, 126.7, 71.9, 65.0, 53.7, 53.5. **^{31}P{^{1}H} NMR** (162 MHz, D2O) *δ (ppm)*: 23.94. **HRMS (ESI)** m/z: [**2** + **CB8**]$^{2+}$ Calcd for $C_{79}H_{76}N_{33}O_{16}P^{2+}$ 886.7937; Found 886.7949.

Then, the following NMR tubes were prepared:

- Tube 1: 500 µL of 2mM stock solution of **2**·2Br.
- Tube 2: 250 µL of 2mM stock solution of **2**·2Br + 250 µL of the solution of stoichiometry 1:1 of 2·2Br and CB[8].
- Tube 3: 500 µL of the solution of stoichiometry 1:1 of **2**·Br and CB[8].

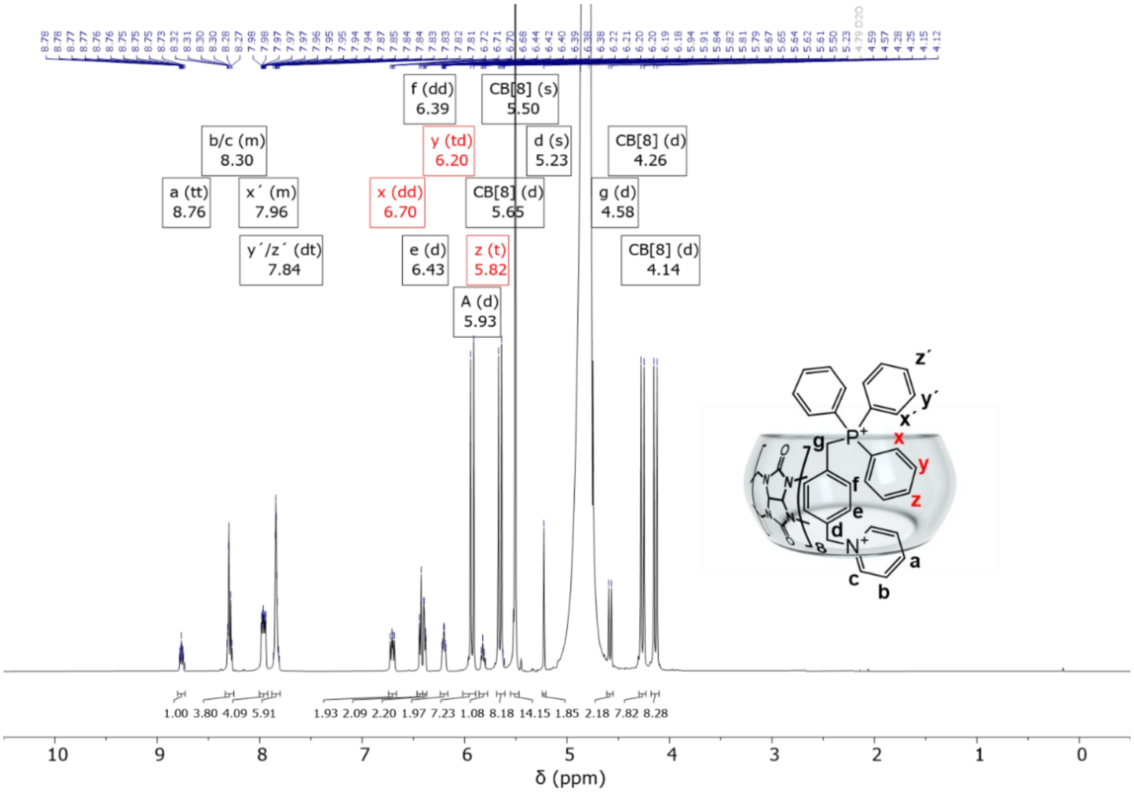

Figure S66: ^{1}H NMR (500 MHz, D_2O) spectrum of $\mathbf{2^{2+}}$□**CB[8]**.

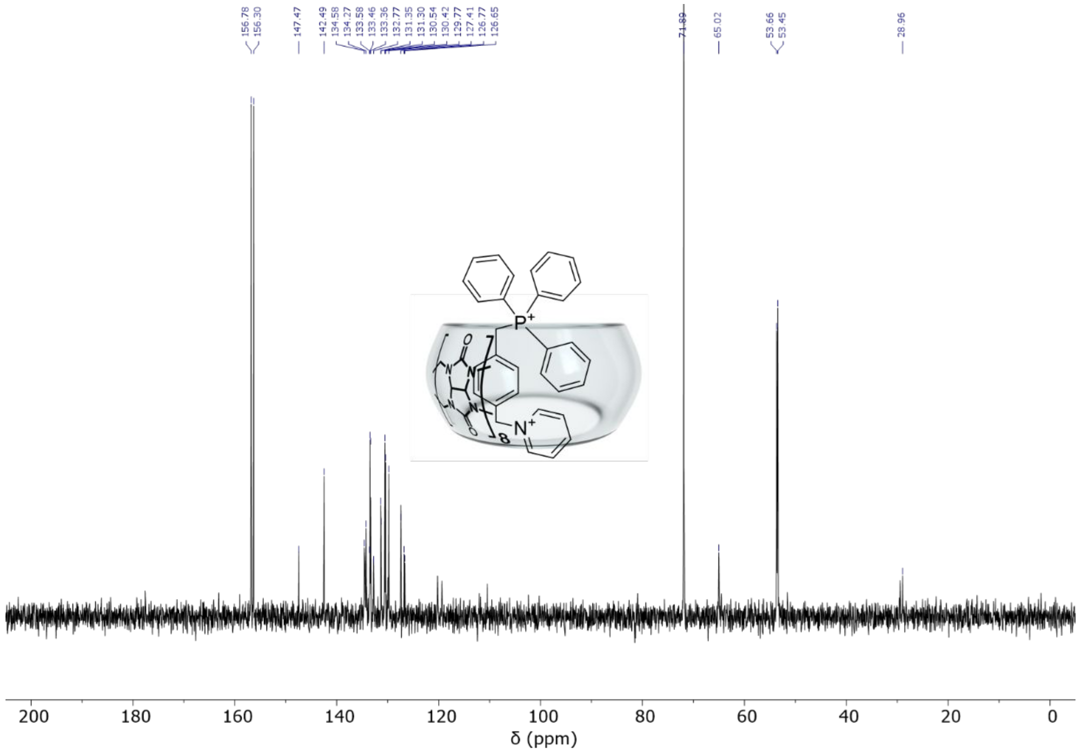

Figure S67: $^{13}C\{^{1}H\}$ NMR (101 MHz, D_2O) spectrum of $\mathbf{2^{2+}}$□**CB[8]**.

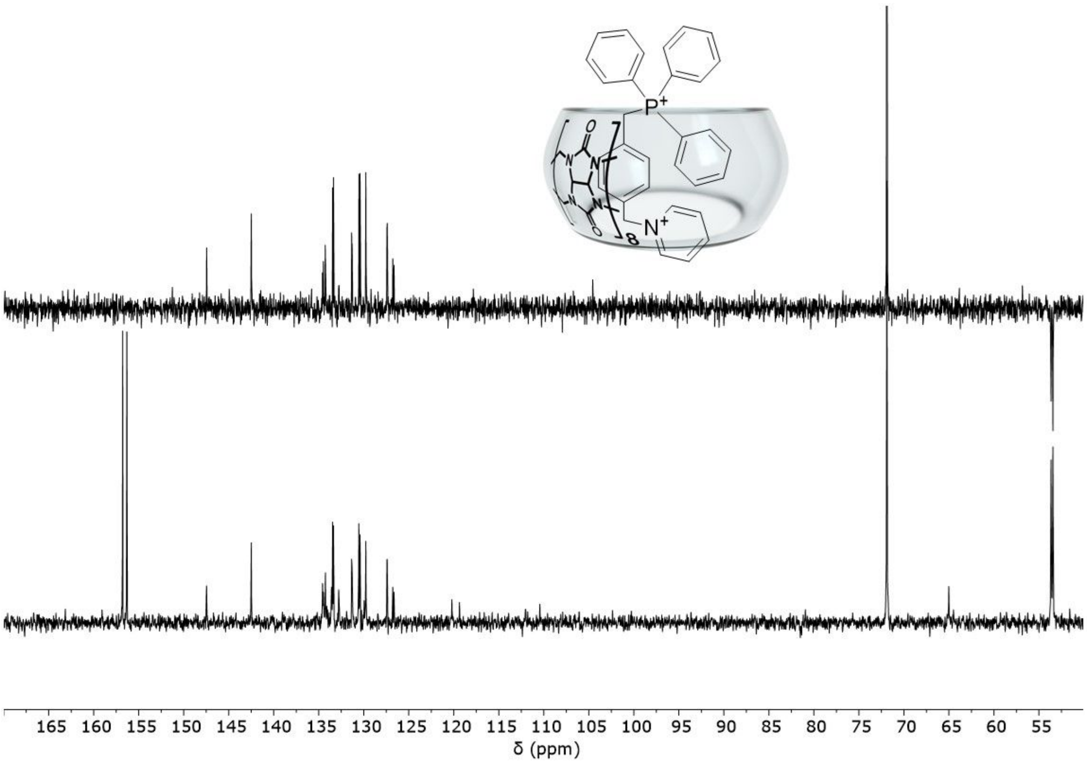

Figure S68: DEPT-135 (101 MHz, D_2O) spectrum (up) and $^{13}C\{^1H\}$ NMR (101 MHz, D_2O) spectrum (down) of **2**$^{2+}$□**CB[8]**.

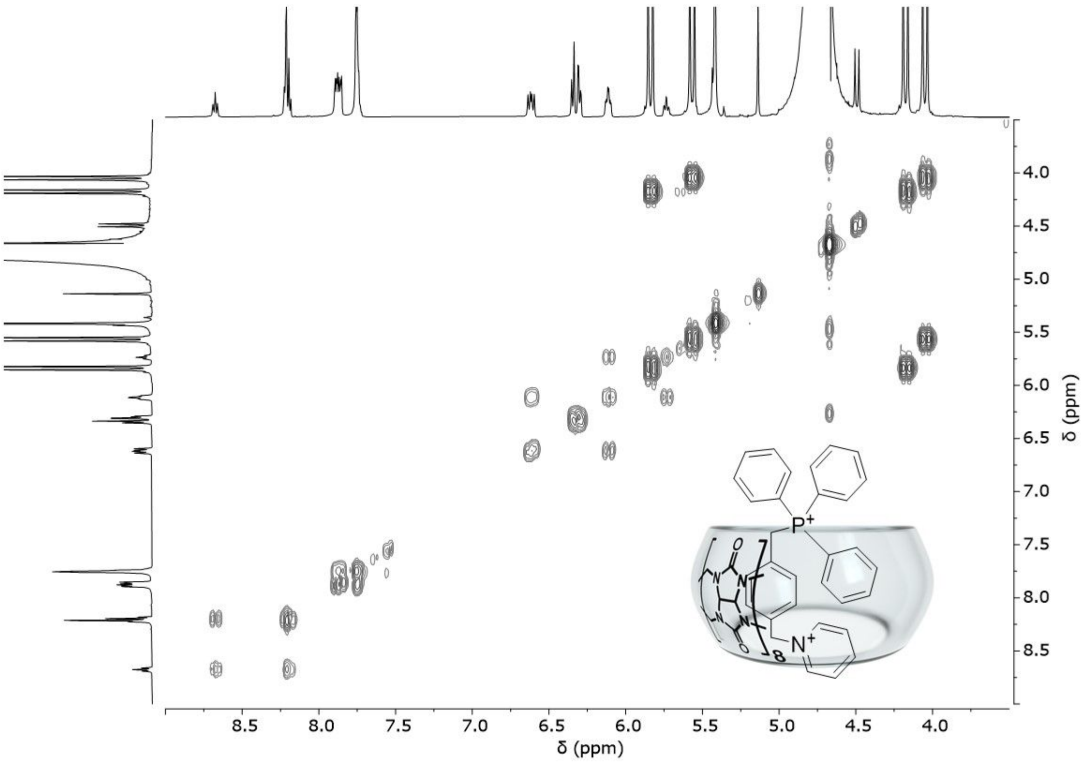

Figure S 69: ^1H-^1H COSY (500 MHz, D_2O) spectrum of **2**$^{2+}$□**CB[8]**.

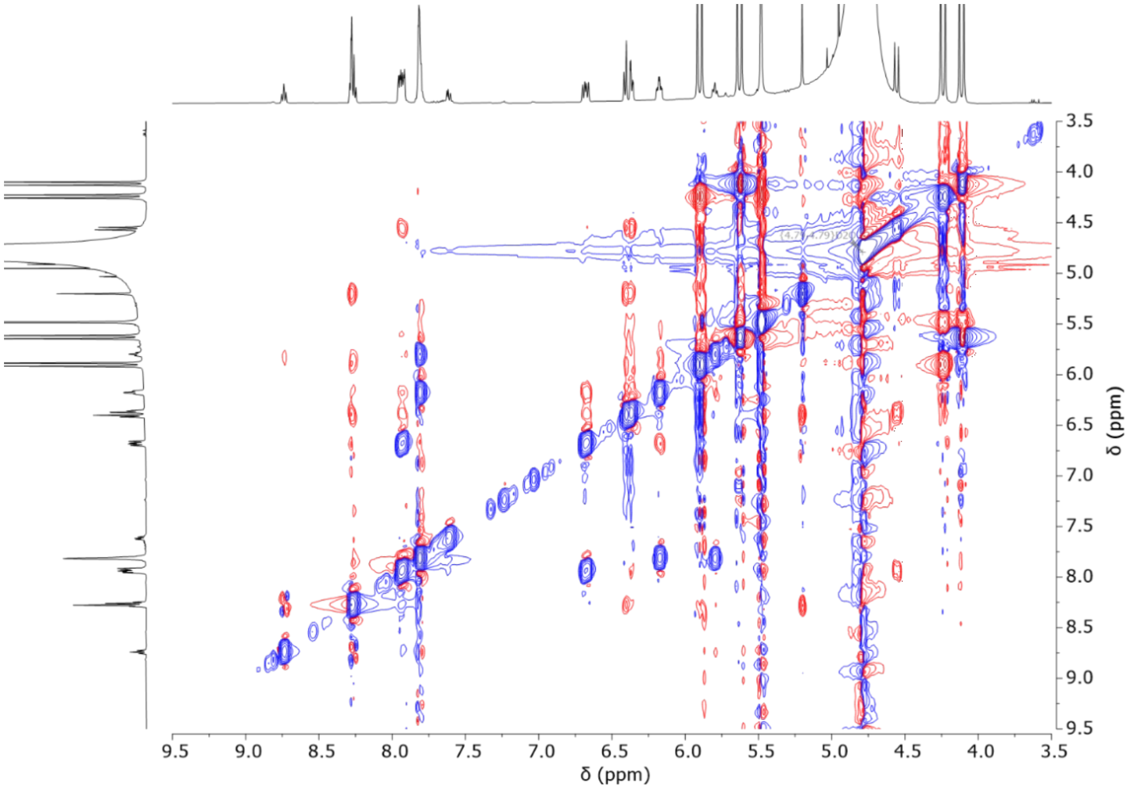

Figure S70: ^{1}H-^{1}H ROESY/EXSY (500 MHz, D_2O) spectrum of **2**$^{2+}$□**CB[8]**. Red cross peaks indicate ROE correlations and blue cross peaks indicate EXSY correlations.

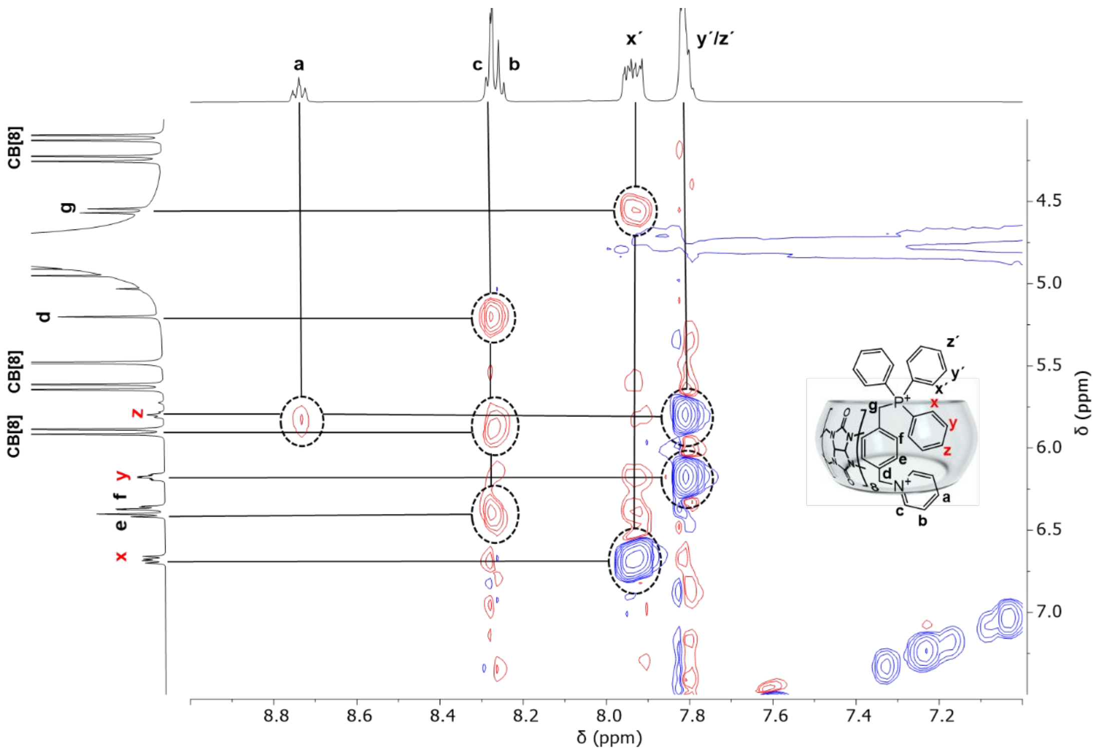

Figure S71: Partial ^1H-^1H ROESY/EXSY (500 MHz, D_2O) spectrum of **2**$^{2+}$□**CB[8]**. Red cross peaks indicate ROE correlations and blue cross peaks indicate EXSY correlations.

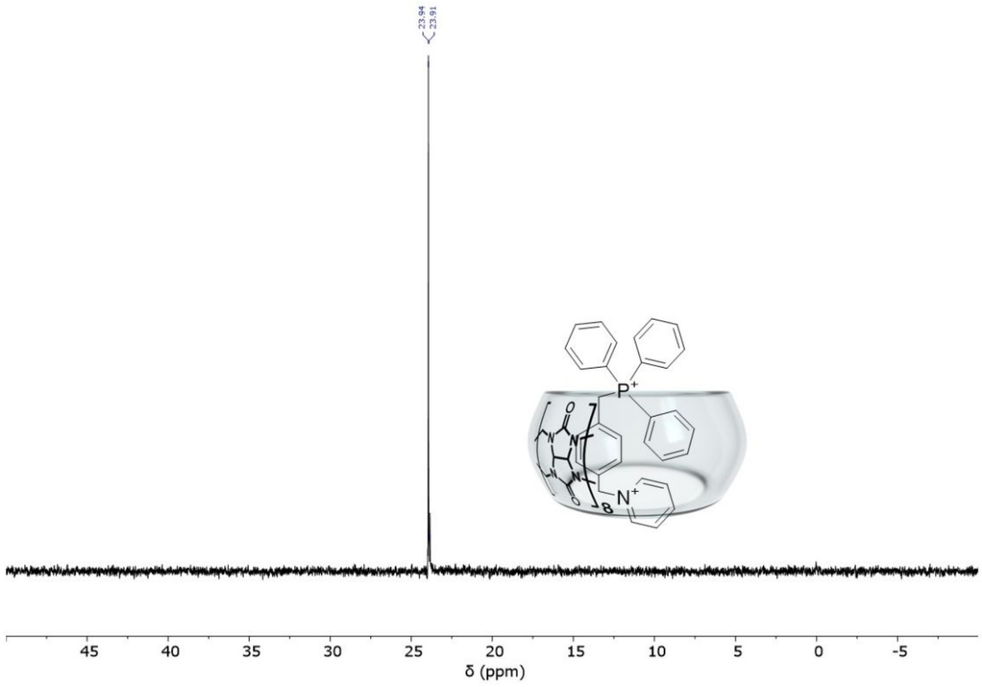

Figure S72: $^{31}P\{^1H\}$ NMR (162 MHz, D_2O) spectrum of **2**$^{2+}$□**CB[8]**.

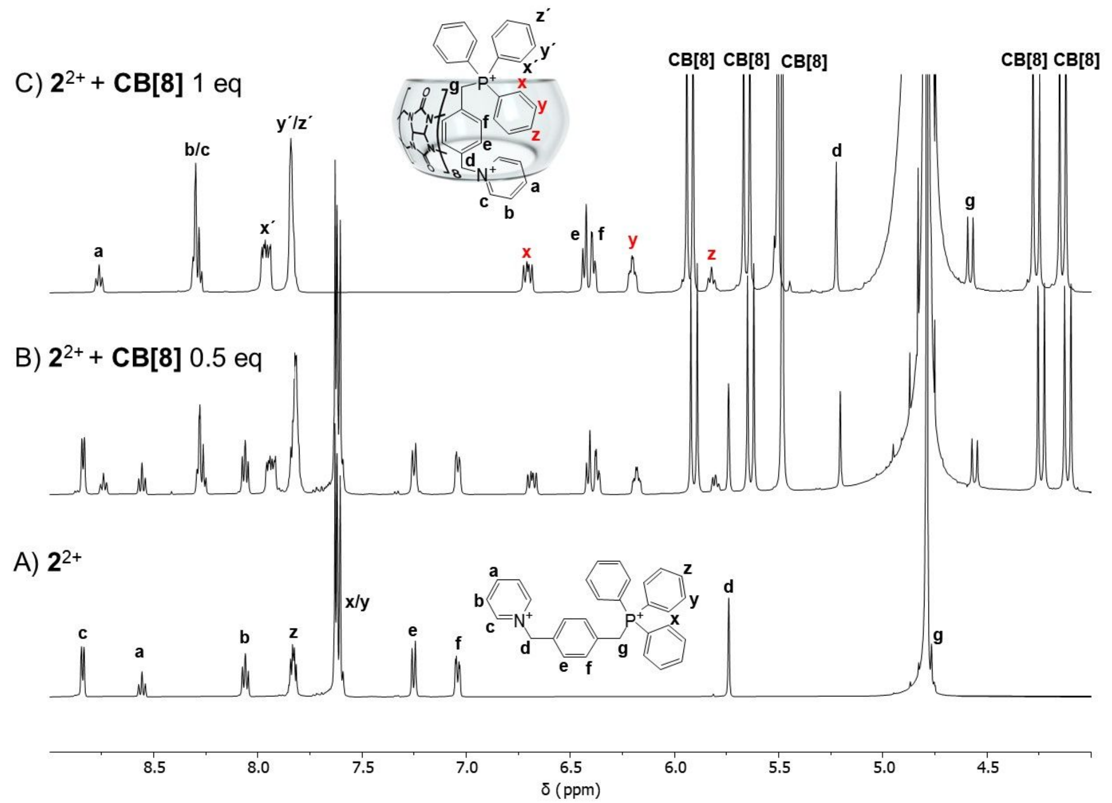

Figure S73: Partial ^{1}H NMR (500 MHz, D_2O) spectrum of: A) $\mathbf{2}^{2+}$; B) $\mathbf{2}^{2+}$ + **CB[8]** 0.5 eq; and C) $\mathbf{2}^{2+}$ + **CB[8]** 1 eq.

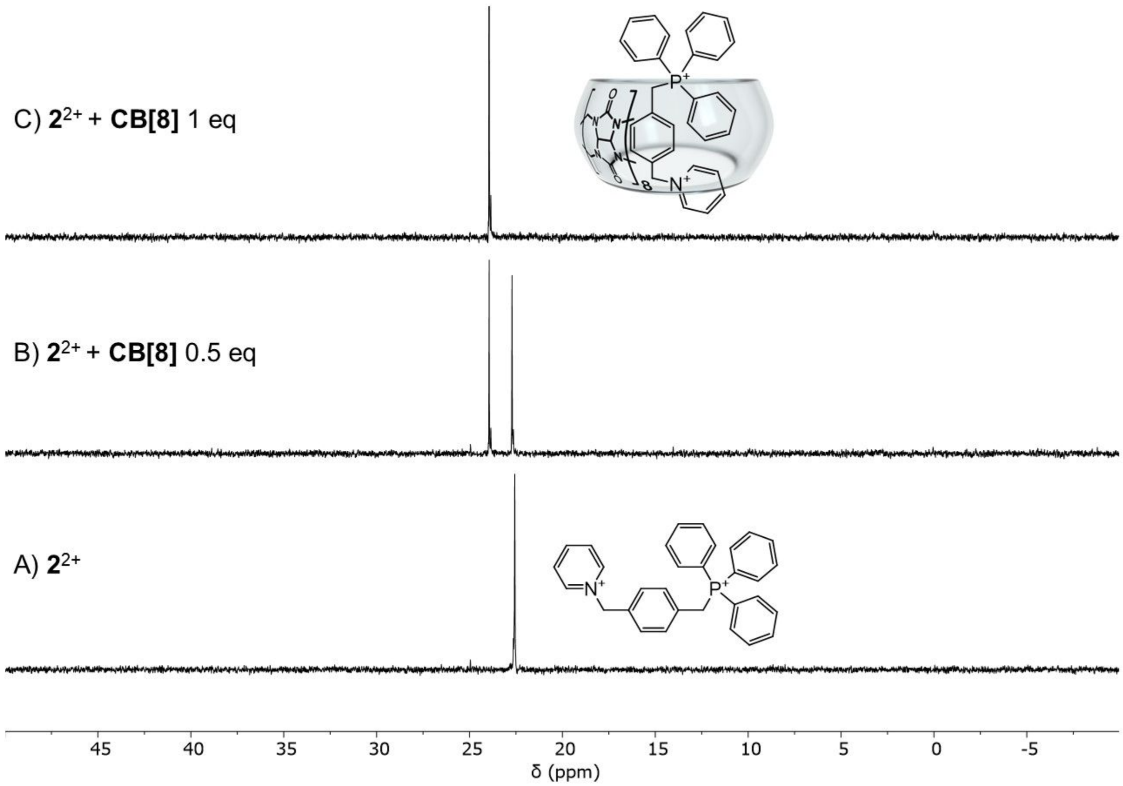

Figure S74: $^{31}P\{^{1}H\}$ NMR (162 MHz, D_2O) spectrum of: A) 2^{2+}; B) 2^{2+} + **CB[8]** 0.5 eq; and C) 2^{2+} + **CB[8]** 1 eq.

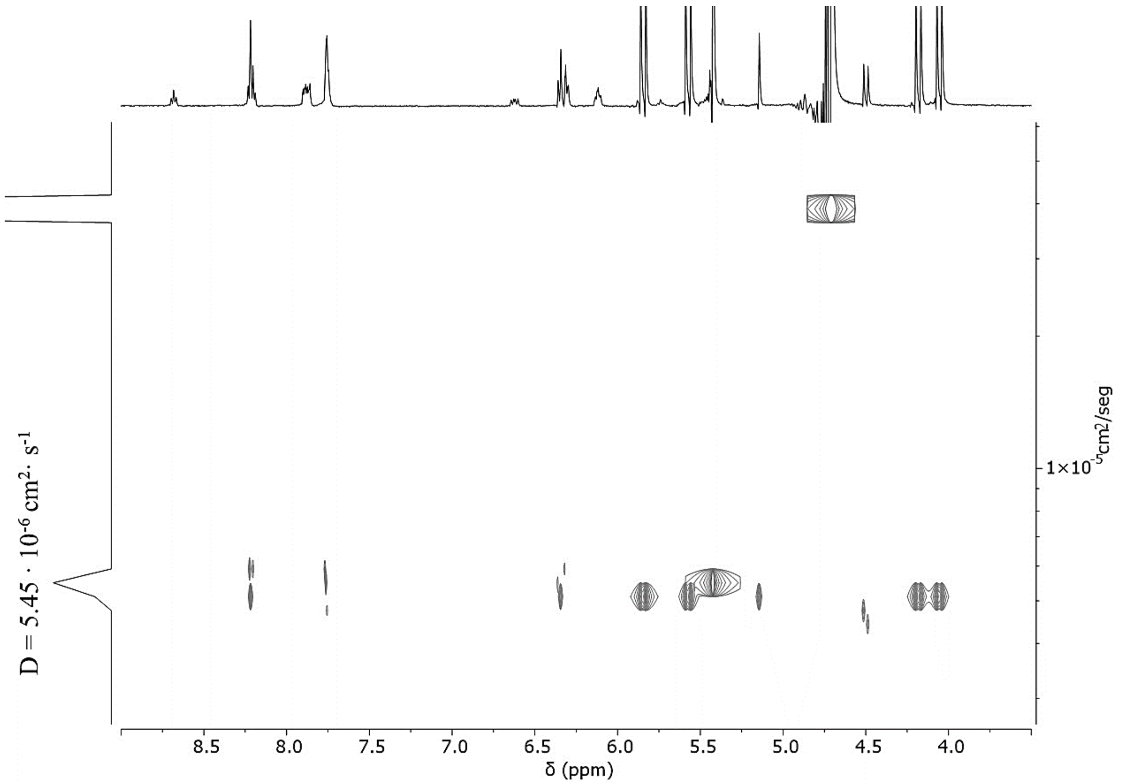

Figure S75: DOSY (500 MHz, D_2O, 298 K) spectrum of 2^{2+}□**CB[8]**.

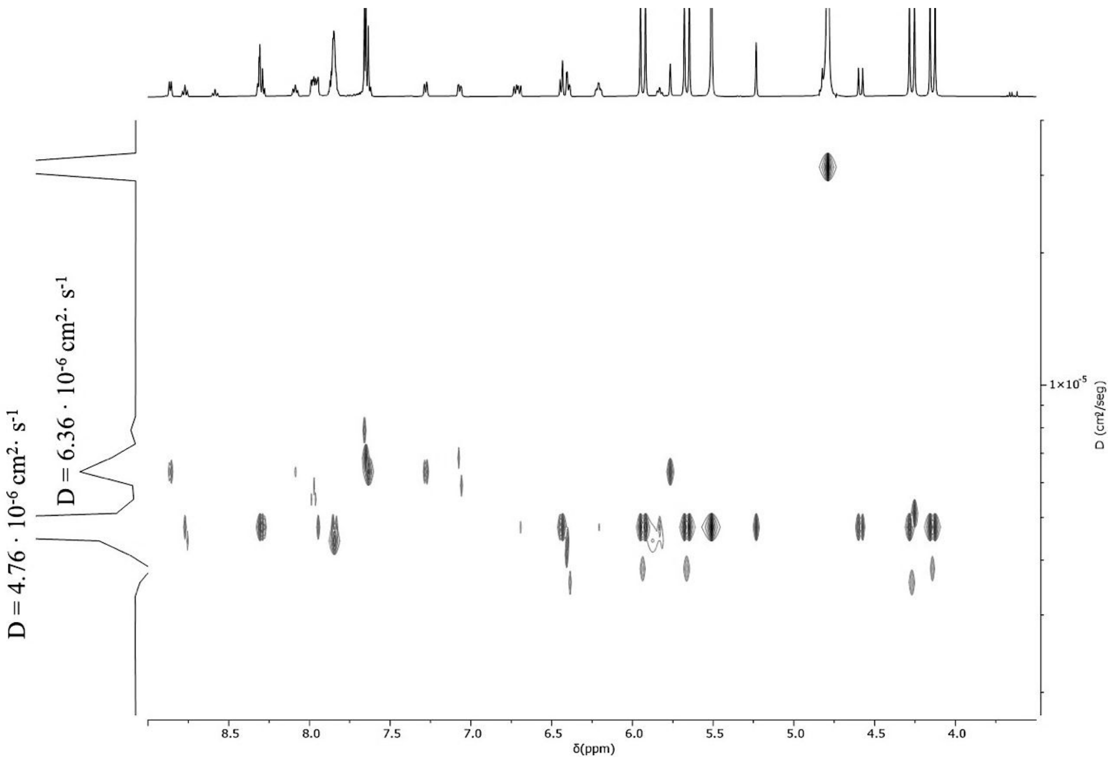

Figure S76: DOSY (500 MHz, D_2O, 298 K) spectrum of **2**$^{2+}$ and **CB[8]** in a 2:1 molar ratio.

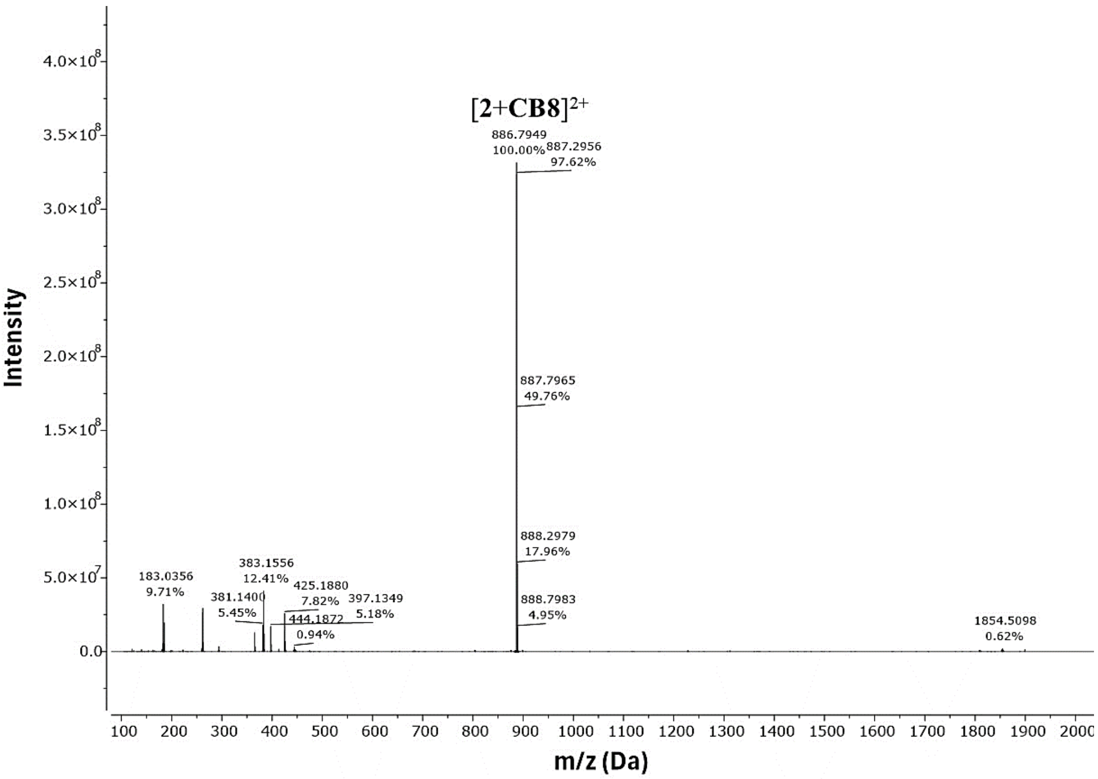

Figure S77: HRMS-ESI spectrum of **2**$^{2+}$□**CB[8]**.

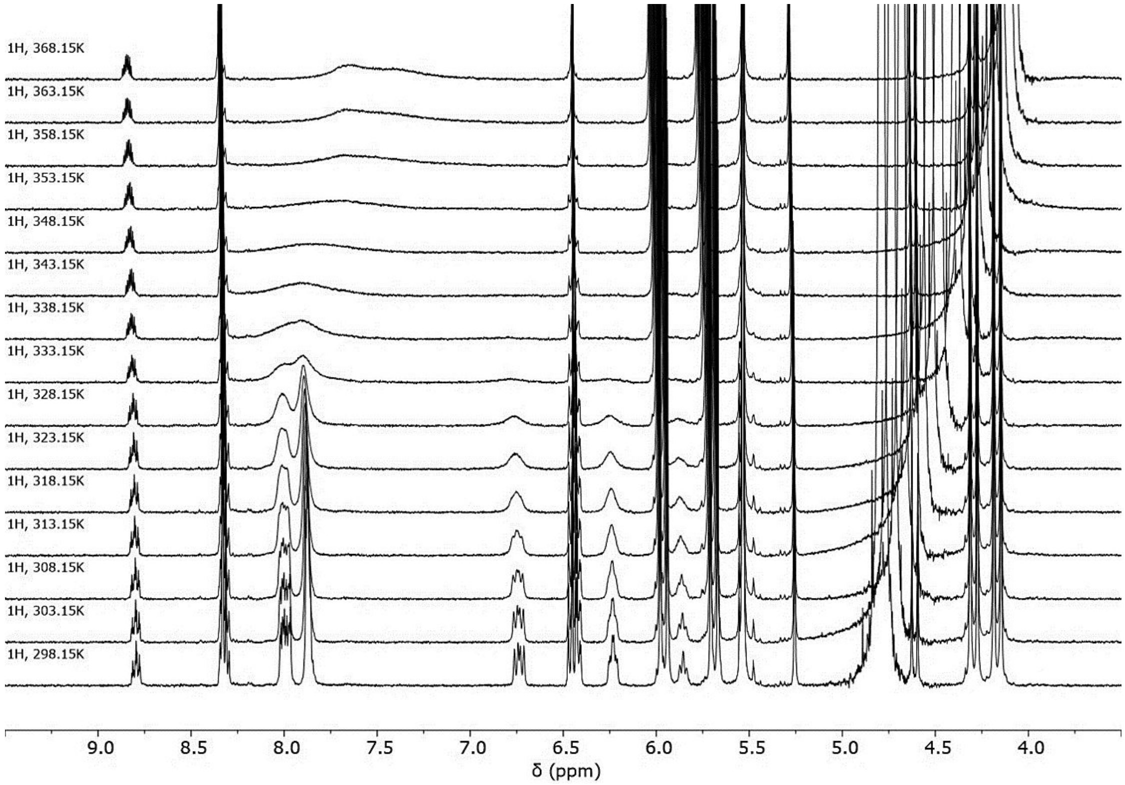

Figure S78: VT-^{1}H-NMR (400 MHz, D_2O) stacked spectra of **2**$^{2+}$□**CB[8]**.

3.2. NMR study of the interaction of 3·2TFA and CB[8]

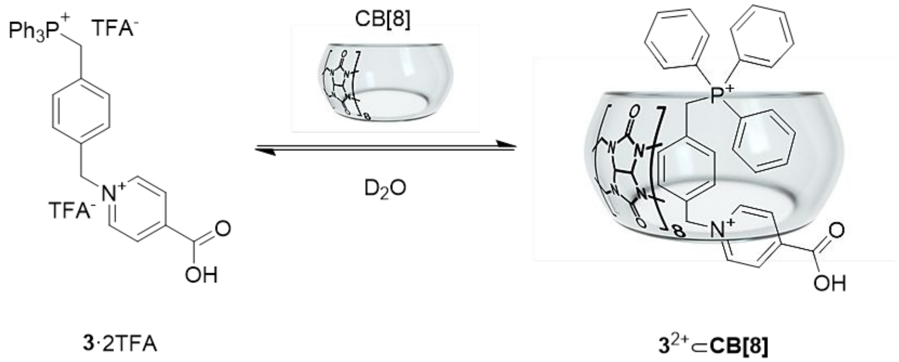

Firstly, the system was studied at pD 3 and pD 12, following the same procedure described for **2**$^{2+}$□**CB[8]** (with the corresponding phosphate buffer solutions 20 mM). The NMR comparison between the complexes with the protonated and deprotonated forms showed no remarkable differences (**Figure S81**).

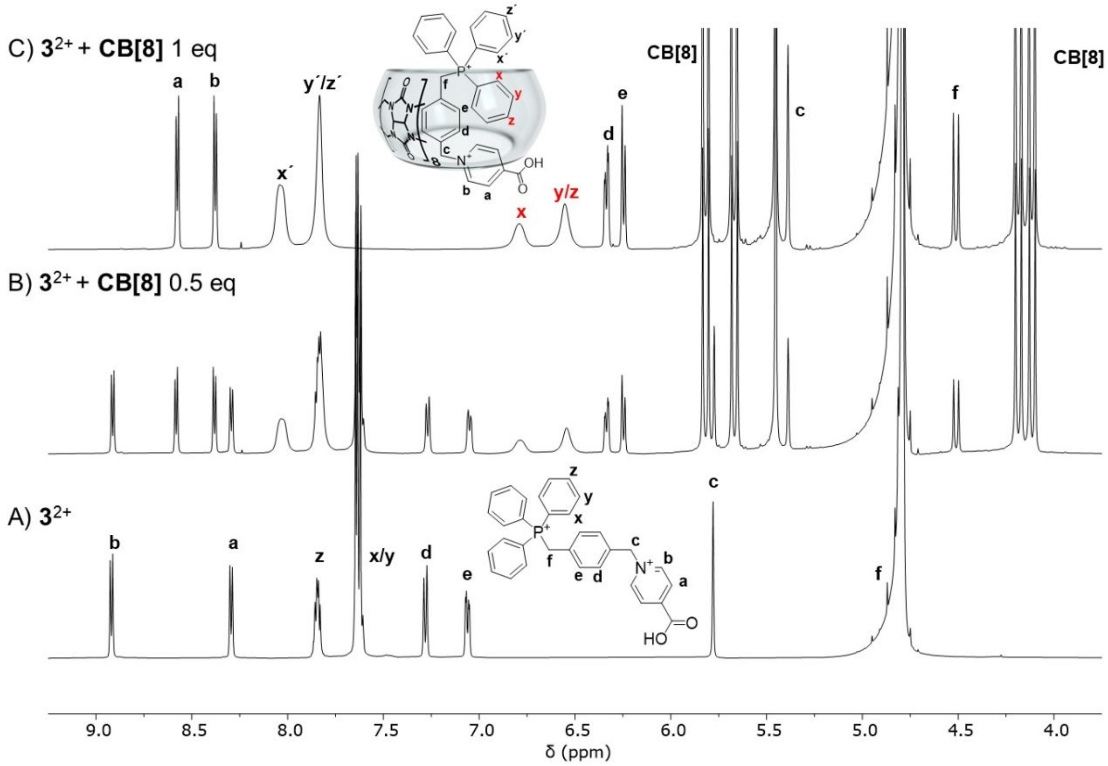

Figure S79: Partial ^{1}H NMR (400 MHz, D_2O) spectrum of: A) **3**$^{2+}$; B) **3**$^{2+}$ + **CB[8]** 0.5 eq; and C) **3**$^{2+}$ + **CB[8]** 1 eq at pD 3.

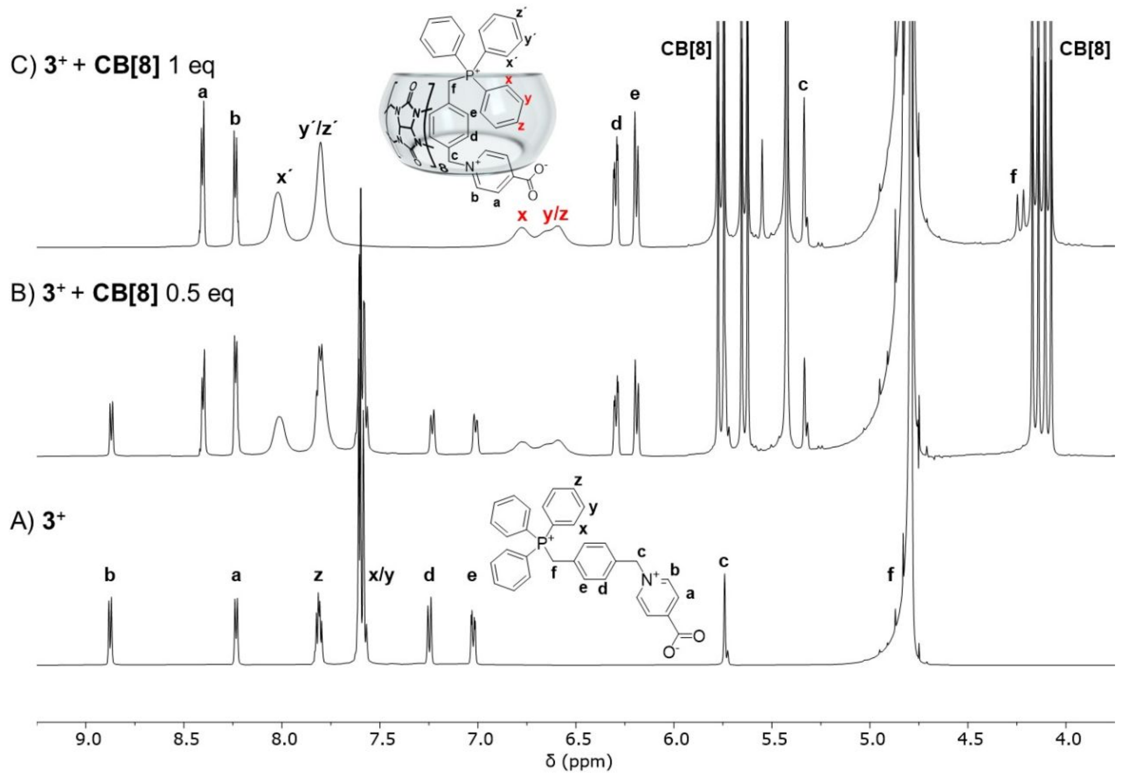

Figure S80: Partial ^{1}H NMR (400 MHz, D_2O) spectrum of: A) **3**$^{+}$; B) **3**$^{+}$ + **CB[8]** 0.5 eq; and C) **3**$^{+}$ + **CB[8]** 1 eq at pD 12.

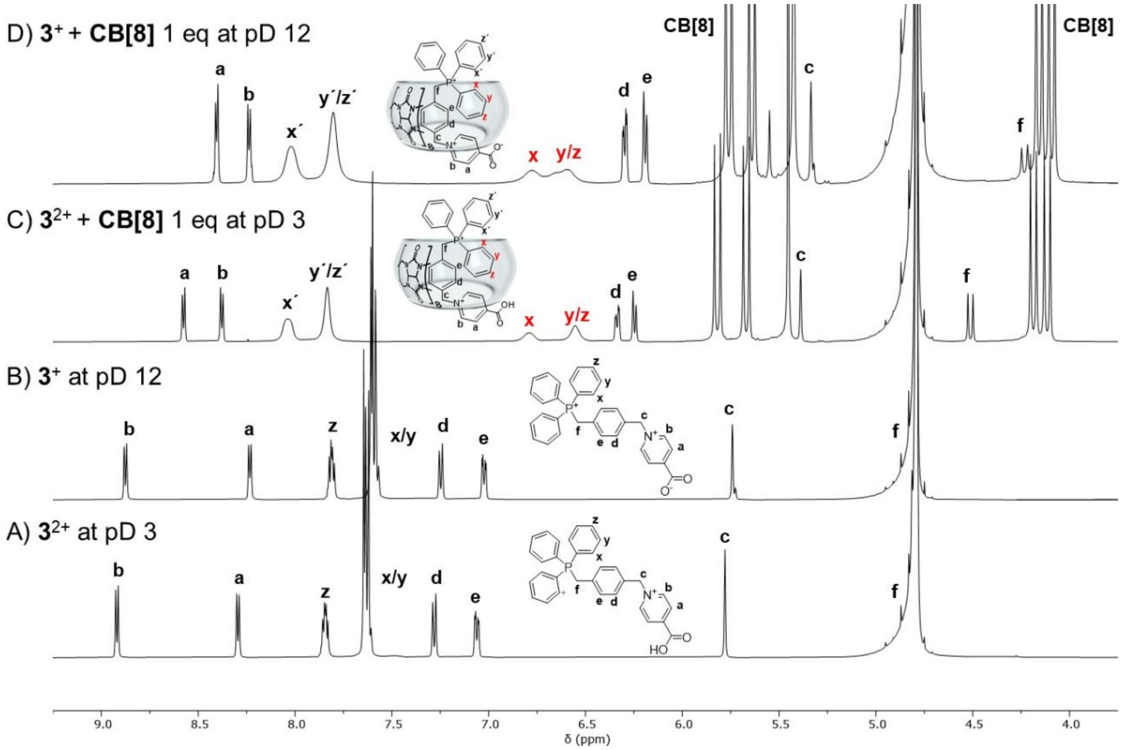

Figure S81: Partial ^{1}H NMR (400 MHz, D_2O) spectrum of: A) $\mathbf{3}^{2+}$ at pD 3; B) $\mathbf{3}^{+}$ at pD 12; C) $\mathbf{3}^{2+}$ + **CB[8]** 1 eq; and D) $\mathbf{3}^{+}$ + **CB[8]** 1 eq at pD 12.

Since no apparent differences in the interaction with CB[8] at pD 3 and pD 12 were observed, the complex $\mathbf{3}^{2+}$□**CB[8]** was fully characterized in D_2O following the same procedure described for $\mathbf{2}^{2+}$□**CB[8]**.

^{1}H NMR (400 MHz, D_2O) *δ (ppm)*: 8.46 (d, *J* = 6.6 Hz, 2H), 8.31 (d, *J* = 6.7 Hz, 2H), 8.15 – 7.99 (m, 4H), 7.86 (s, 7H), 6.94 – 6.57 (m, 5H), 6.34 (dd, *J* = 8.3, 2.6 Hz, 2H), 6.24 (d, *J* = 7.9 Hz, 2H), 5.83 (d, *J* = 15.3 Hz, 9H), 5.71 (d, *J* = 15.2 Hz, 8H), 5.48 (s, 16H), 5.40 (s, 2H), 4.51 (d, *J* = 13.0 Hz, 2H), 4.17 (dd, *J* = 23.4, 15.3 Hz, 17H). **^{13}C{^{1}H} NMR** (101 MHz, D_2O) *δ (ppm)*: 156.6, 156.3, 135.2, 134.1 (d, *J* = 9.7 Hz), 130.6, 129.9 (d, *J* = 12.3 Hz), 127.6, 71.9, 53.4. **^{31}P{^{1}H} NMR** (162 MHz, D_2O) *δ (ppm)*: 24.82. **HRMS (ESI)** m/z: [**3** + **CB8**]$^{2+}$ Calcd for $C_{80}H_{76}N_{33}O_{18}P^{2+}$ 908.7887; Found 908.7893.

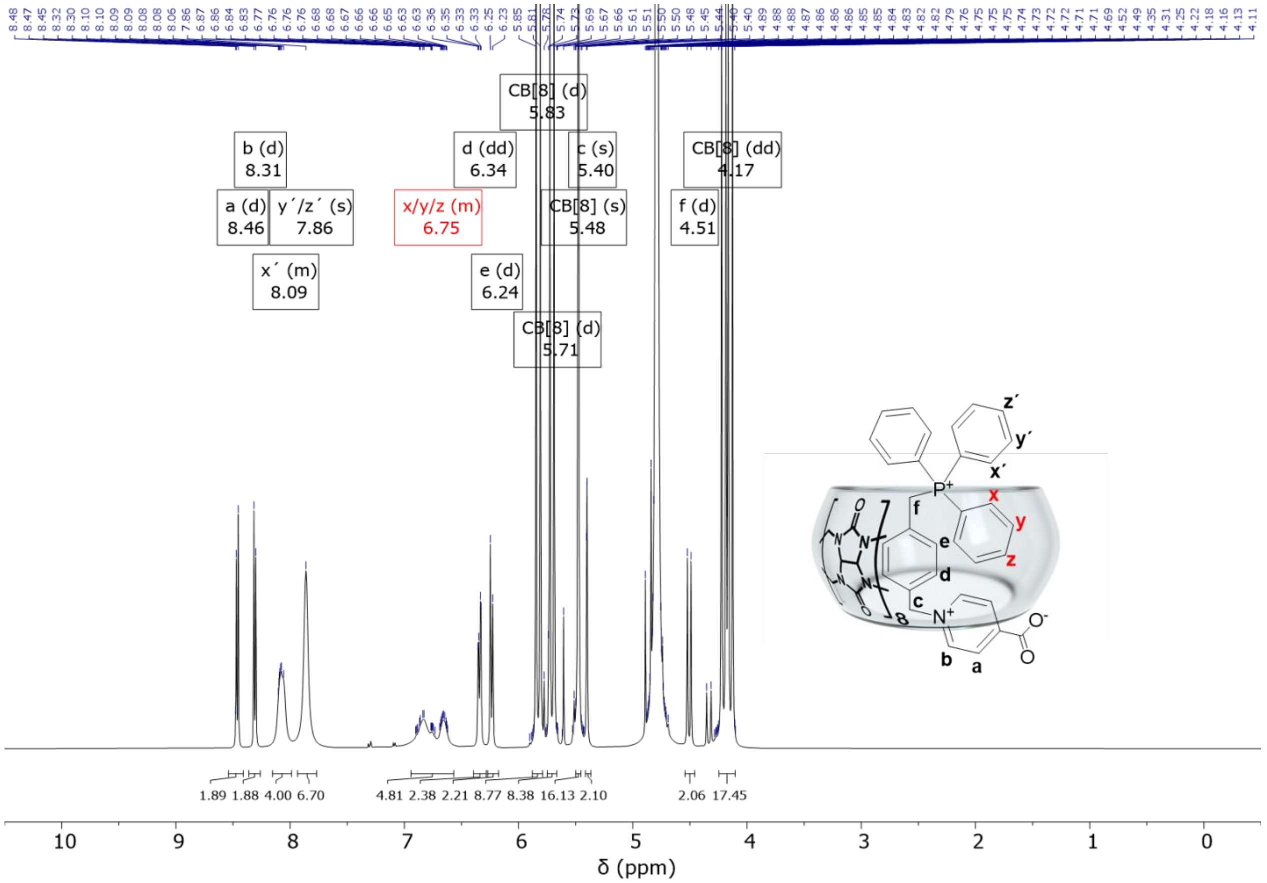

Figure S82: ^{1}H NMR (400 MHz, D_2O) spectrum of **3**$^{2+}$□**CB[8]**.

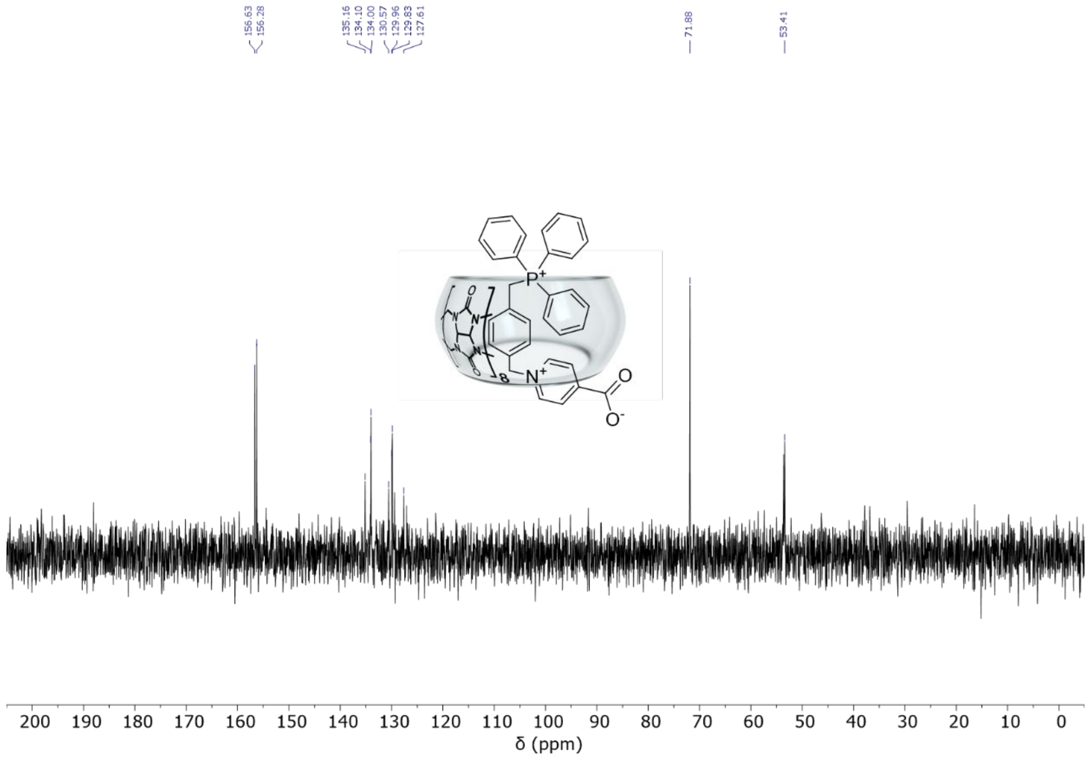

Figure S83: $^{13}C\{^{1}H\}$ NMR (101 MHz, D_2O) spectrum of **3^{2+}□CB[8]**.

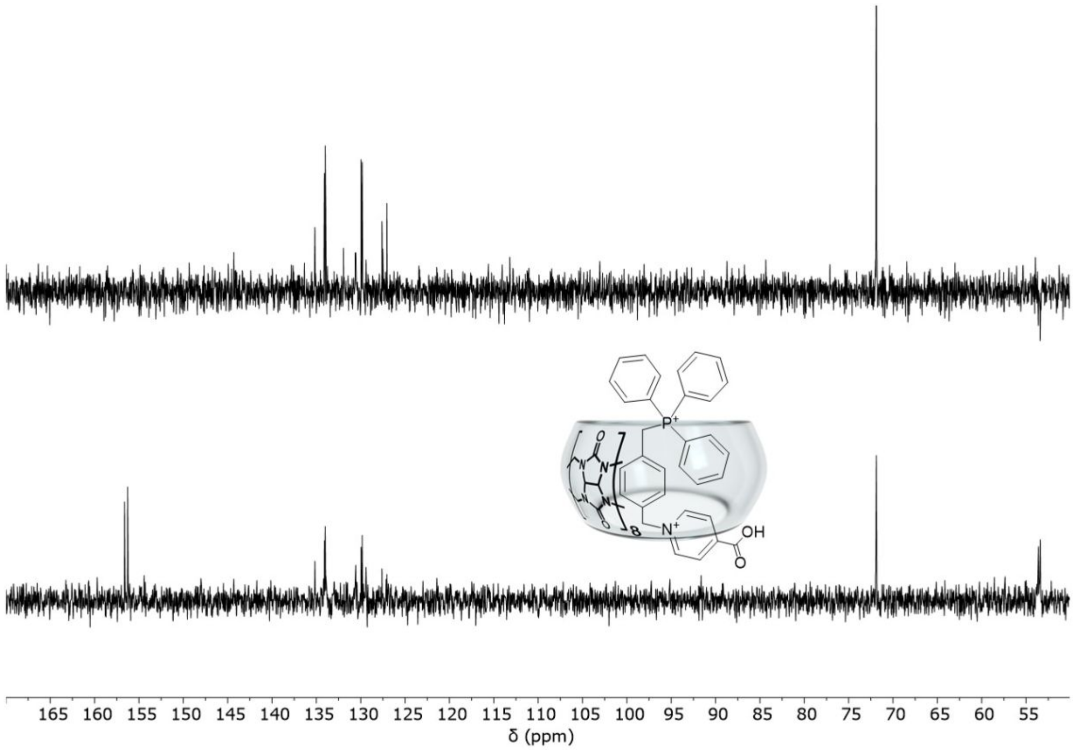

Figure S84: DEPT-135 (101 MHz, D_2O) spectrum (up) and $^{13}C\{^{1}H\}$ NMR (101 MHz, D_2O) spectrum (down) of **3^{2+}□CB[8]**.

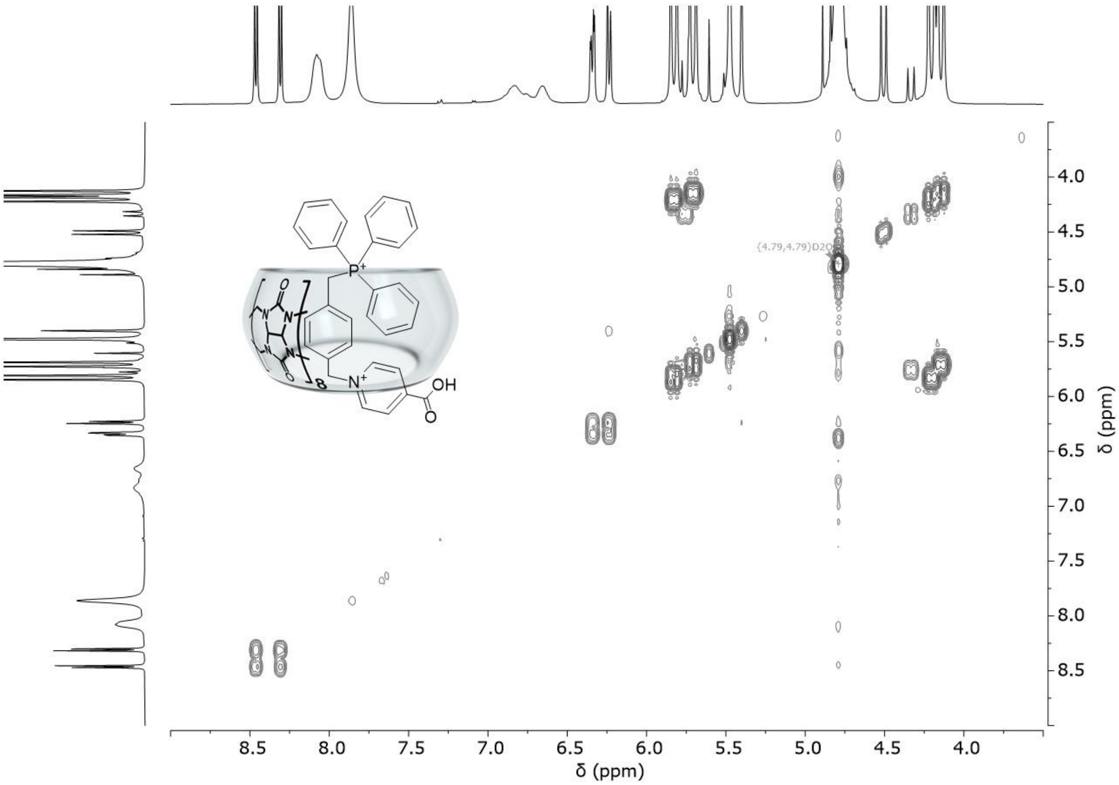

Figure S85: ^{1}H-^{1}H COSY (500 MHz, D_2O) spectrum of **3**$^{2+}$□**CB[8]**.

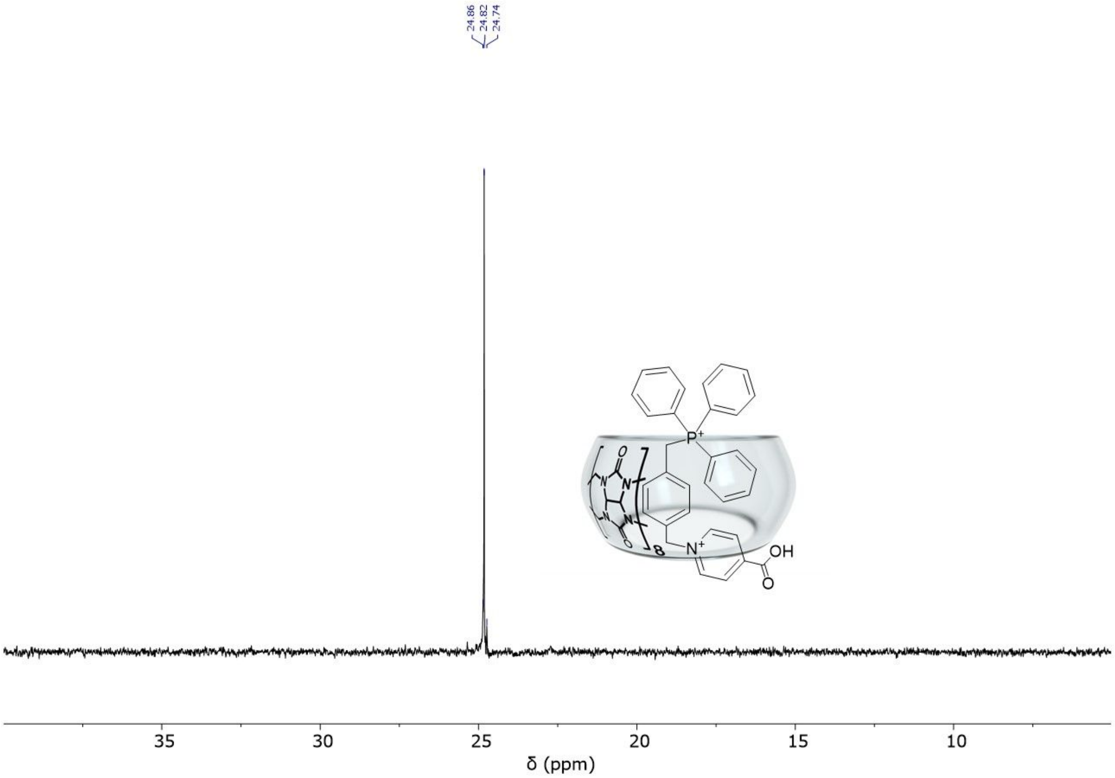

Figure S86: $^{31}P\{^1H\}$ NMR (162 MHz, D_2O) spectrum of **3**$^{2+}$□**CB[8]**.

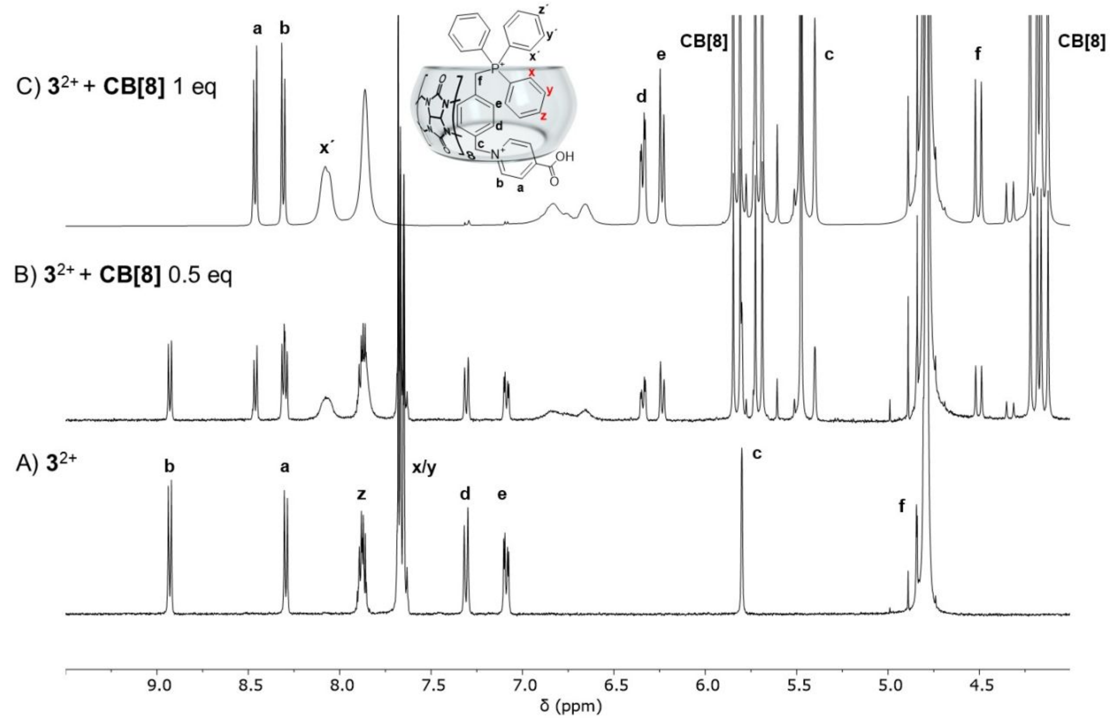

Figure S87: Partial ^{1}H NMR (400 MHz, D_2O) spectrum of: A) $\mathbf{3}^{2+}$; B) $\mathbf{3}^{2+}$ + **CB[8]** 0.5 eq; and C) $\mathbf{3}^{2+}$ + **CB[8]** 1 eq.

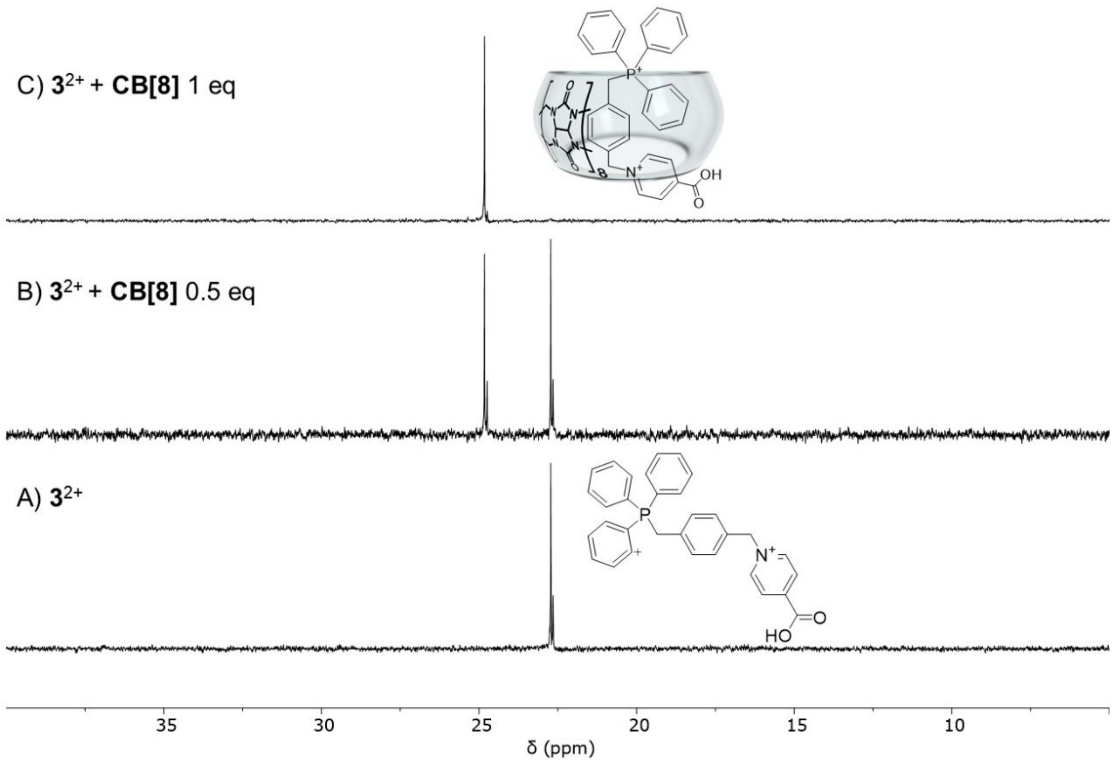

Figure S88: $^{31}P\{^{1}H\}$ NMR (162 MHz, D_2O) spectrum of: A) $\mathbf{3}^{2+}$; B) $\mathbf{3}^{2+}$ + **CB[8]** 0.5 eq; and C) $\mathbf{3}^{2+}$ + **CB[8]** 1 eq.

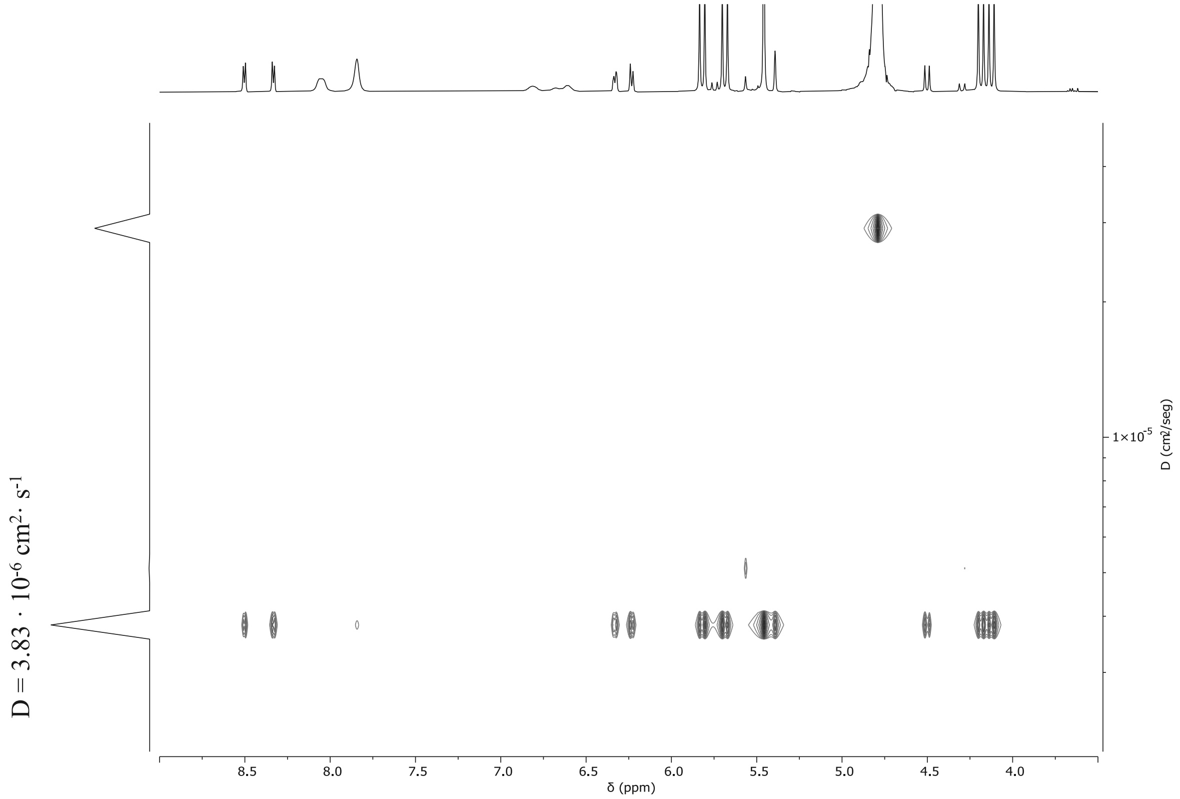

Figure S89: DOSY (500 MHz, D_2O, 298 K) spectrum of **3^{2+}**□**CB[8]**.

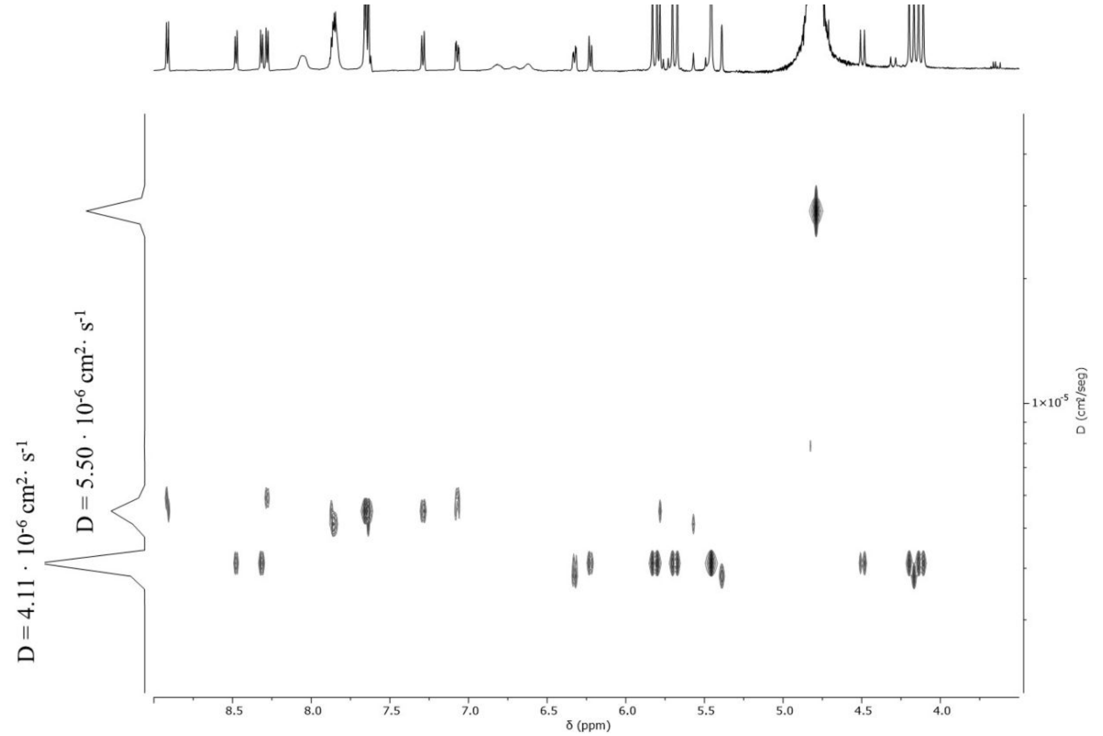

Figure S90: DOSY (500 MHz, D_2O, 298 K) spectrum of **3^{2+}** and **CB[8]** in a 2:1 molar ratio.

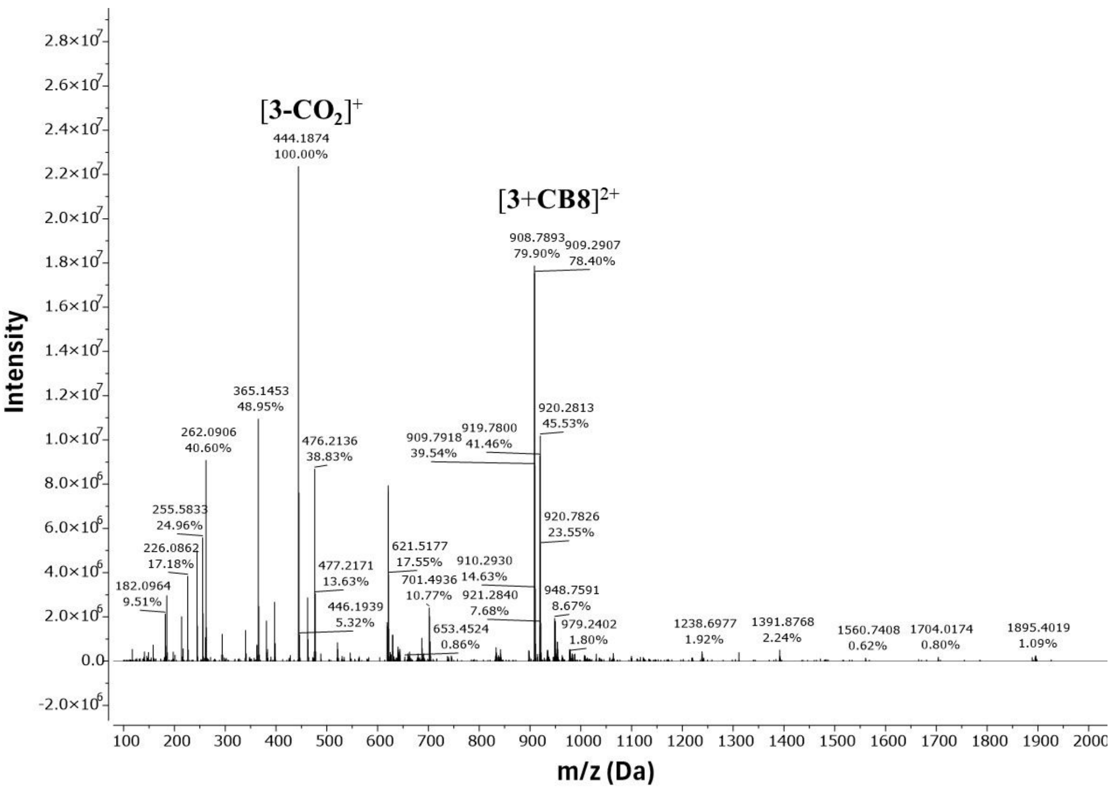

Figure S91: HRMS-ESI spectrum of $\mathbf{3}^{2+}$□**CB[8]**.

3.3. NMR study of the interaction of 4·3Cl and CB[8]

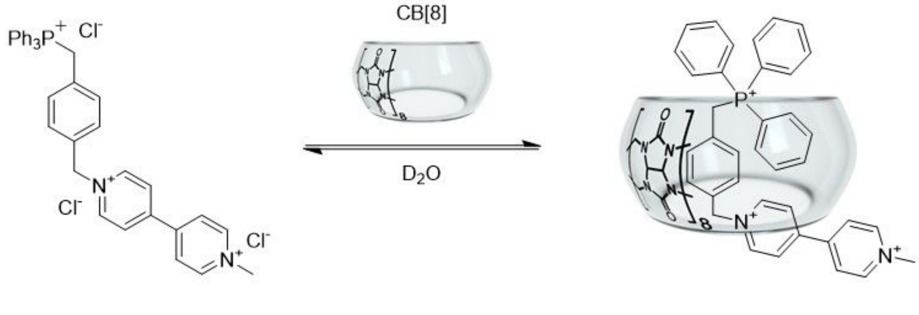

4·3Cl $\qquad$ $\mathbf{4}^{3+}\subset$**CB[8]**

Same procedure described for **2**$^{2+}$□**CB[8]**.

^{1}H NMR (500 MHz, D_2O) *δ (ppm)*: 9.15 (d, *J* = 6.4 Hz, 2H), 8.87 (d, *J* = 6.3 Hz, 2H), 8.62 (d, *J* = 6.3 Hz, 2H), 8.51 (d, *J* = 6.4 Hz, 2H), 8.04 (s, 4H), 7.84 (s, 5H), 6.79 (s, 2H), 6.48 (s, 2H), 6.42 (d, *J* = 6.4 Hz, 2H), 6.35 (d, *J* = 7.8 Hz, 2H), 6.30 (s, 1H), 5.81 (d, *J* = 15.3 Hz, 7H), 5.66 (d, *J* = 15.1 Hz, 7H), 5.47 (s, 13H), 5.43 (s, 2H), 4.55 (s, 3H), 4.54 (d, *J* = 12.8 Hz, 2H), 4.21 (d, *J* = 15.3 Hz, 7H), 4.12 (d, *J* = 15.1 Hz, 7H). **^{13}C{^{1}H} NMR** (126 MHz, D_2O) *δ (ppm)*: 156.8, 156.20, 151.3, 148.9, 146.1, 144.7, 134.2, 132.9, 132.0, 131.9, 131.2, 130.7, 127.8, 127.3, 64.3, 53.6, 53.3, 48.2, 28.6, 28.2. **HRMS (ESI)** m/z: [**4**+**CB8**]$^{3+}$ Calcd for 622.2134; Found 622.2137; [**4**+**CB8**+Cl]$^{2+}$ Calcd for $C_{85}H_{82}ClN_{34}O_{16}P^{2+}$ 950.8049; Found 950.8065.

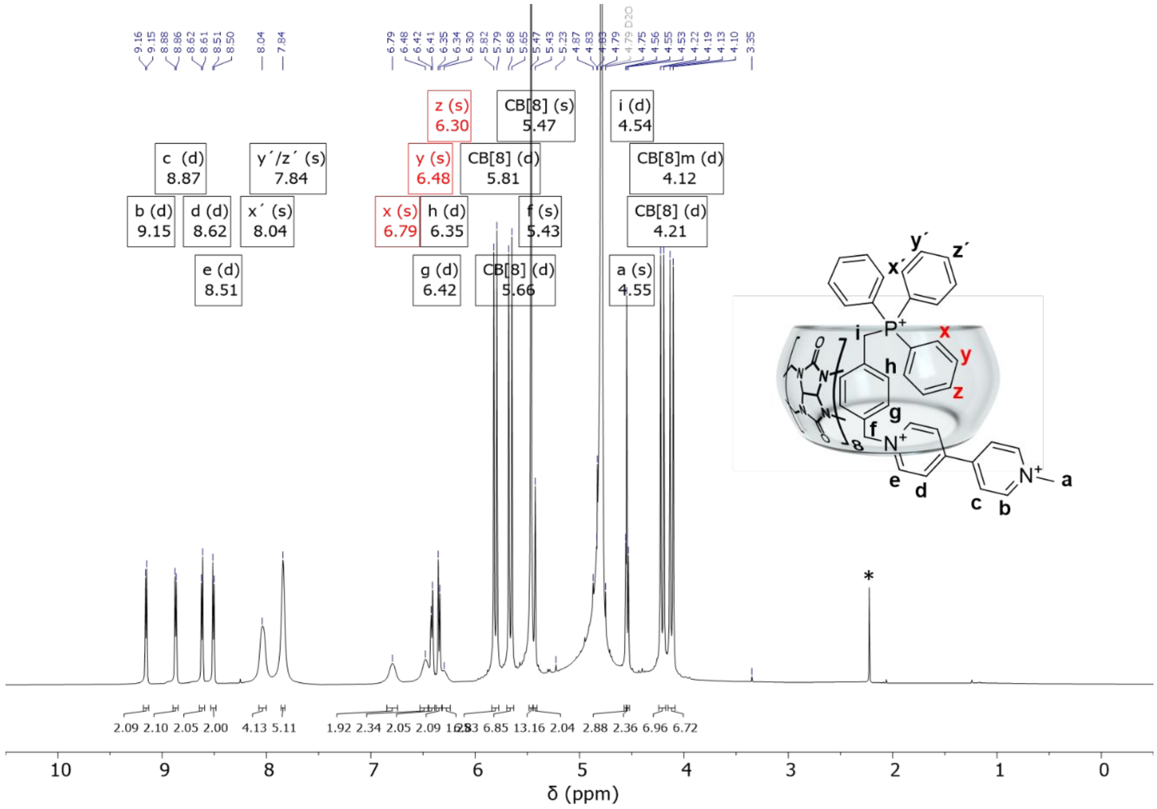

Figure S92: ^{1}H NMR (500 MHz, D_2O) spectrum of **4**$^{3+}$□**CB[8]**. Impurities are marked with *****: acetone (2.22 ppm, s).

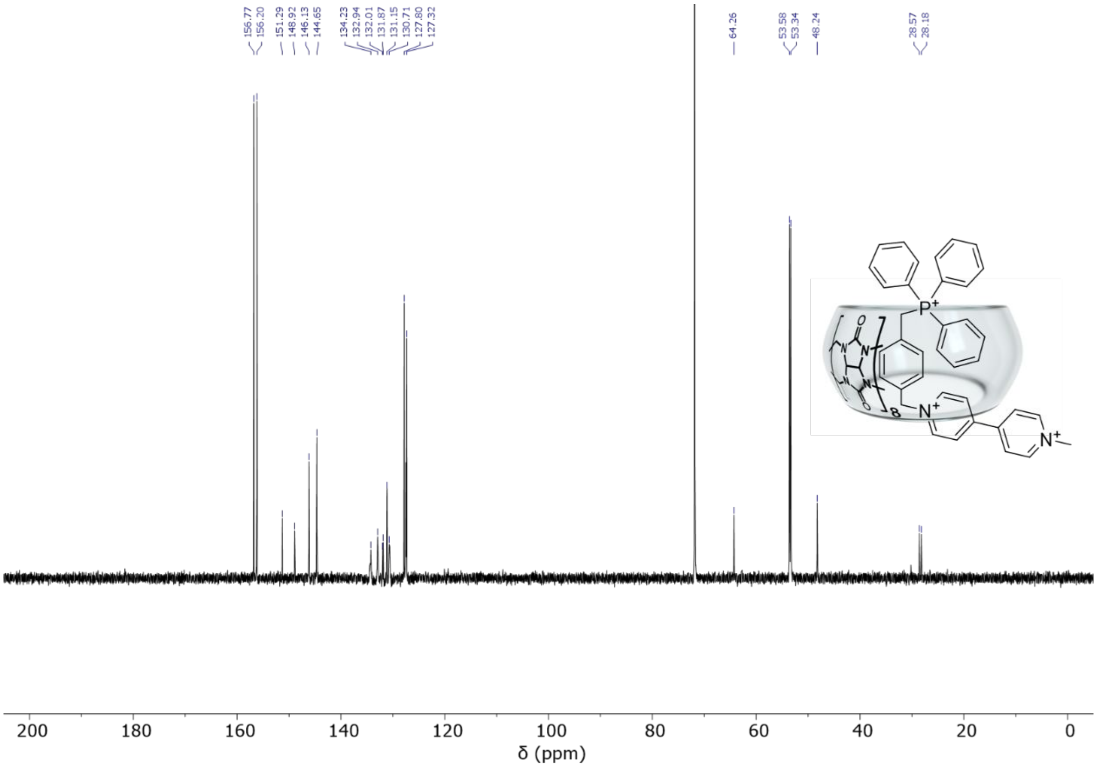

Figure S93: ^{13}C NMR (126 MHz, D_2O) spectrum of **4^{3+}□CB[8]**.

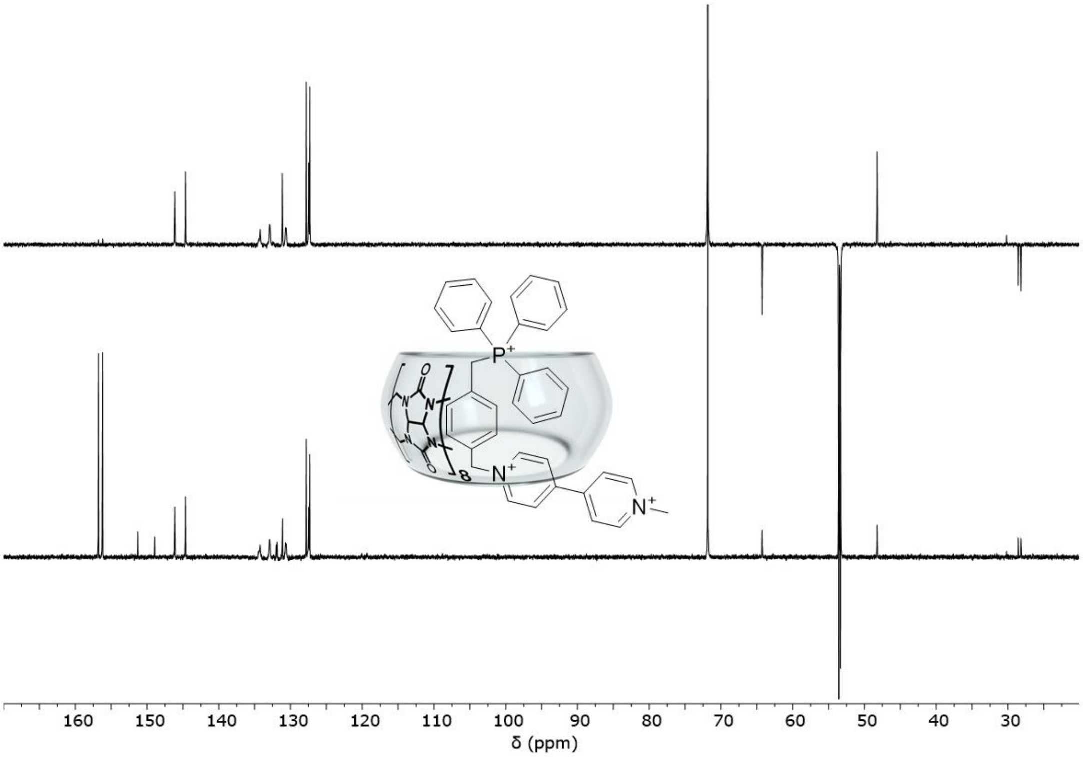

Figure S94: DEPT-135 (126 MHz, D_2O) spectrum (up) and $^{13}C\{^1H\}$ NMR (126 MHz, D_2O) spectrum (down) of **4^{3+}□CB[8]**.

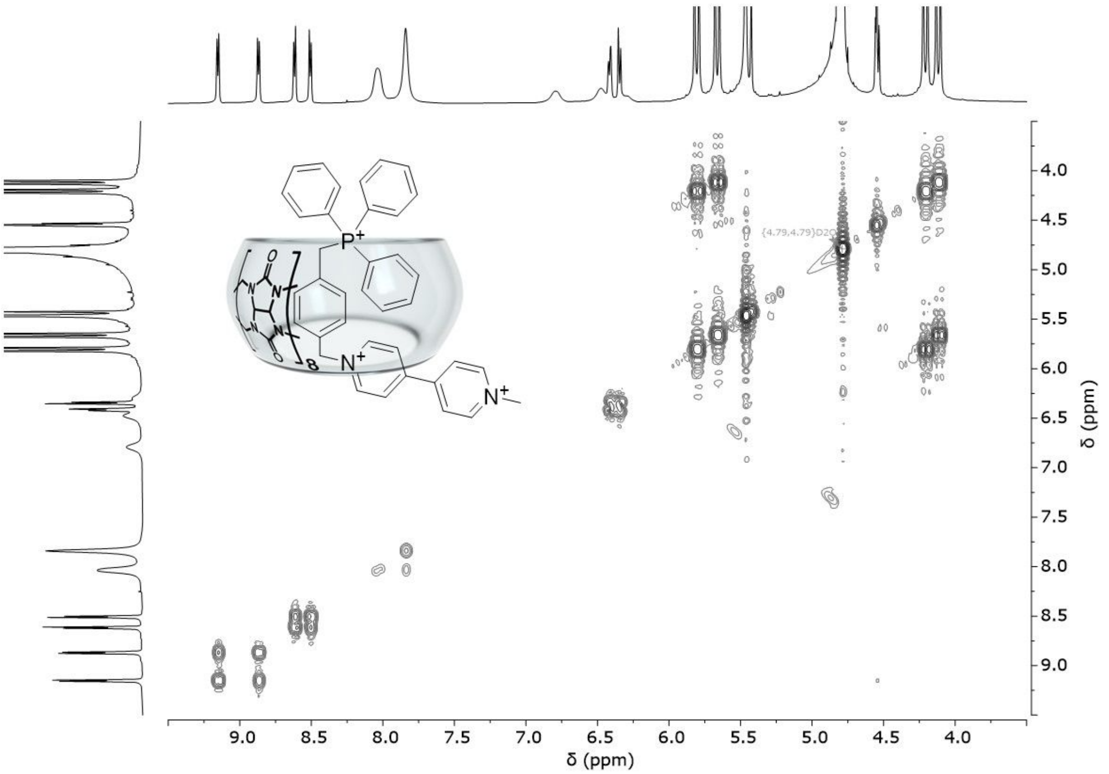

Figure S95: ^1H-^1H COSY (500 MHz, D_2O) spectrum of $\mathbf{4}^{3+}$□**CB[8]**.

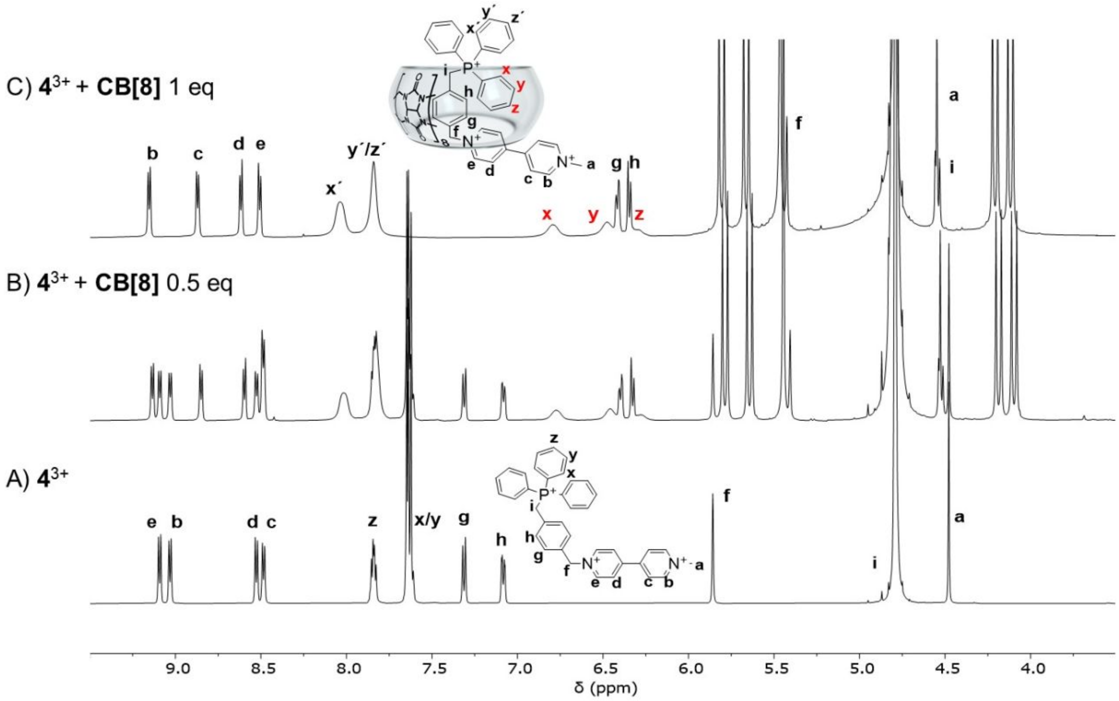

Figure S96: Partial ^1H NMR (500 MHz, D_2O) spectrum of: A) $\mathbf{4}^{3+}$; B) $\mathbf{4}^{3+}$ + **CB[8]** 0.5 eq**;** and C) $\mathbf{4}^{3+}$ + **CB[8]** 1 eq.

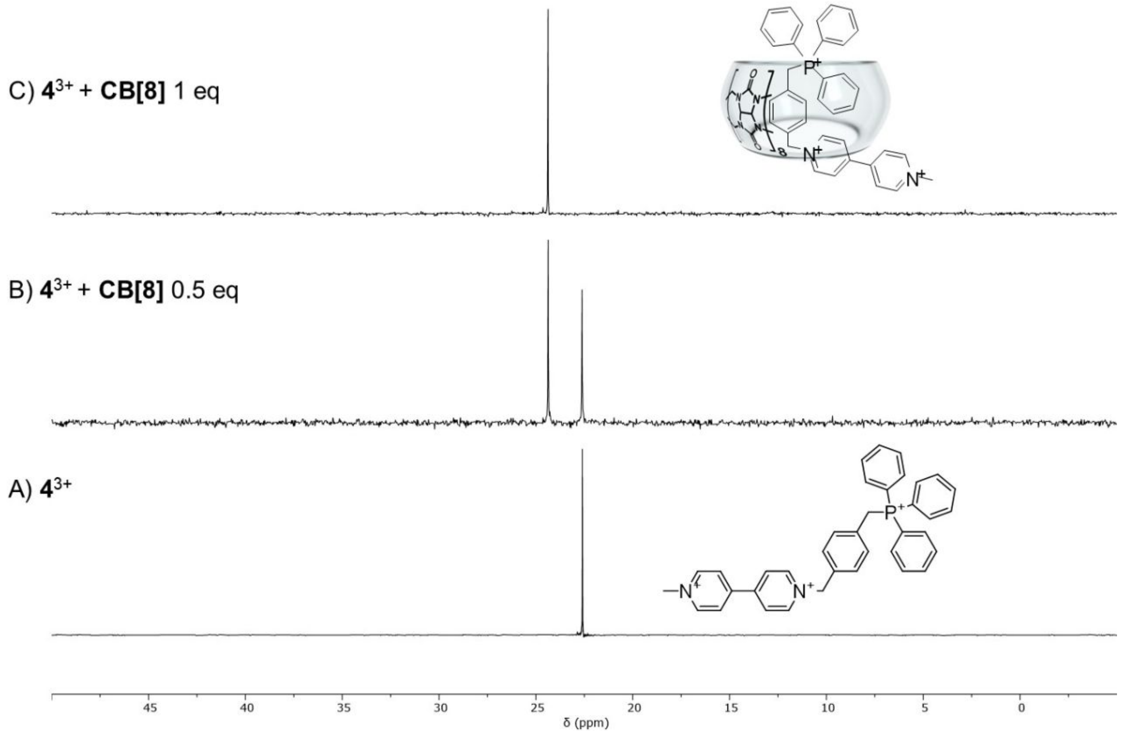

Figure S97 $^{31}P\{^{1}H\}$ NMR (162 MHz, D_2O) spectrum of: A) $\mathbf{4}^{3+}$; B) $\mathbf{4}^{3+}$ + **CB[8]** 0.5 eq; and C) $\mathbf{4}^{3+}$ + **CB[8]** 1 eq.

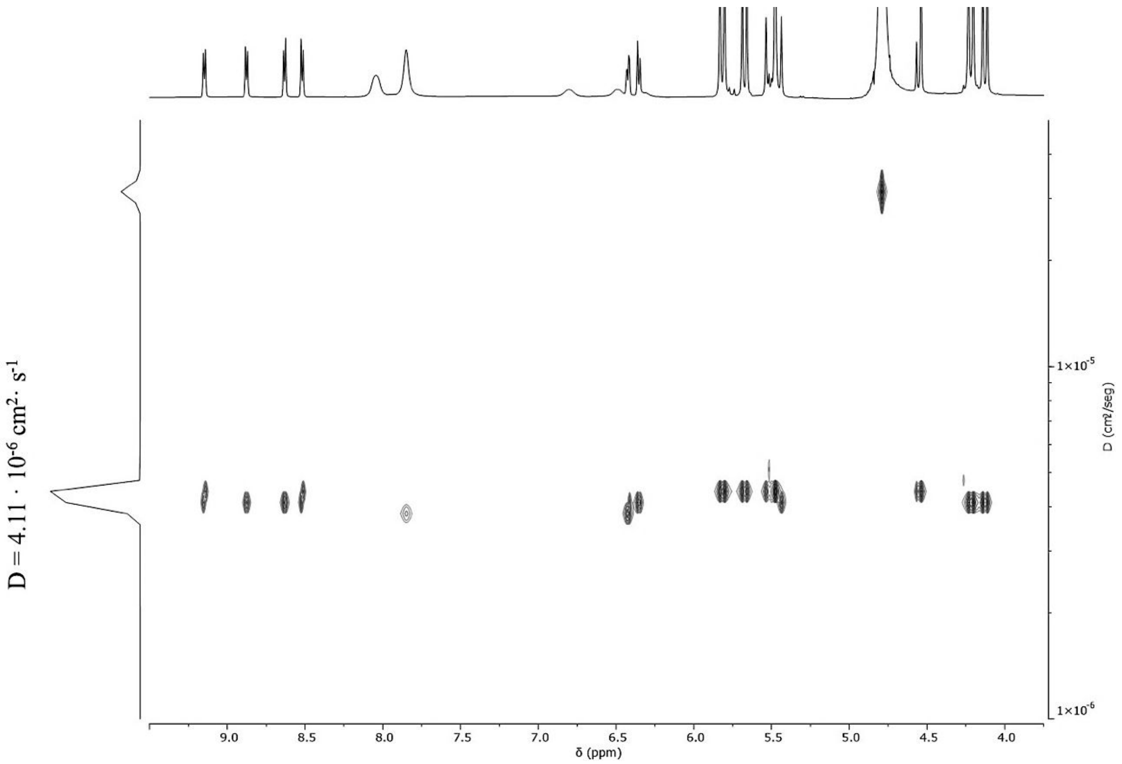

Figure S98: DOSY (500 MHz, D_2O, 298 K) spectrum of $\mathbf{4}^{3+}$□**CB[8]**.

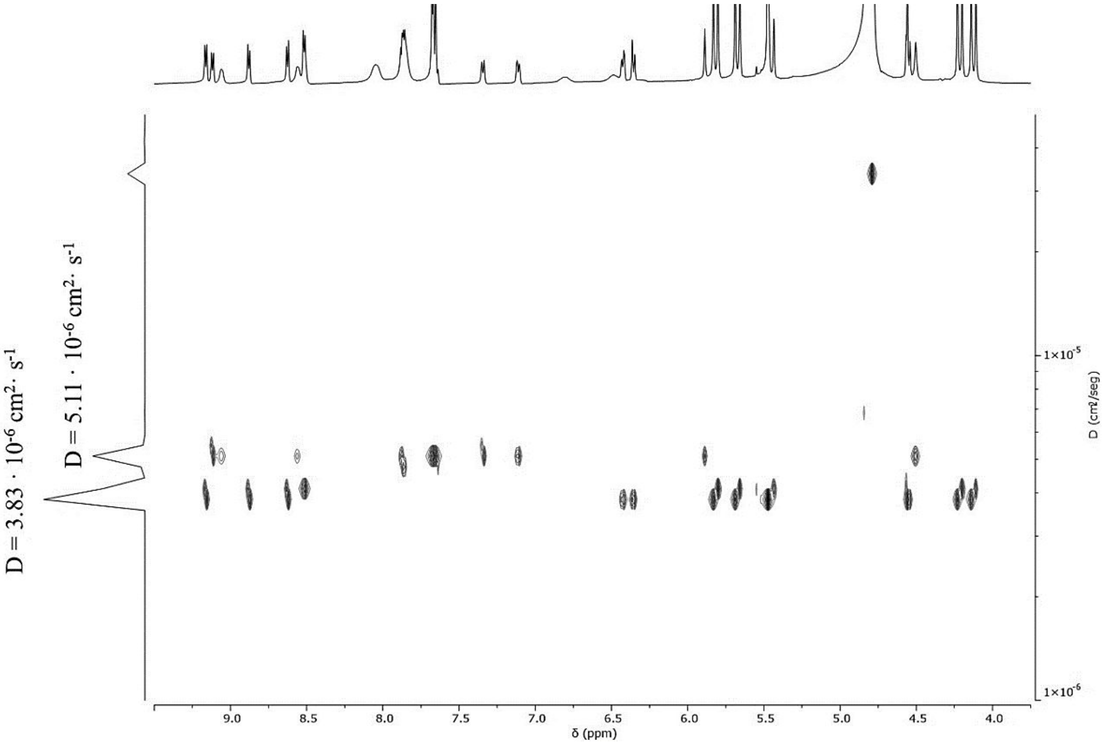

Figure S99: DOSY (500 MHz, D_2O, 298 K) spectrum of $\mathbf{4}^{3+}$ and **CB[8]** in a 2:1 molar ratio.

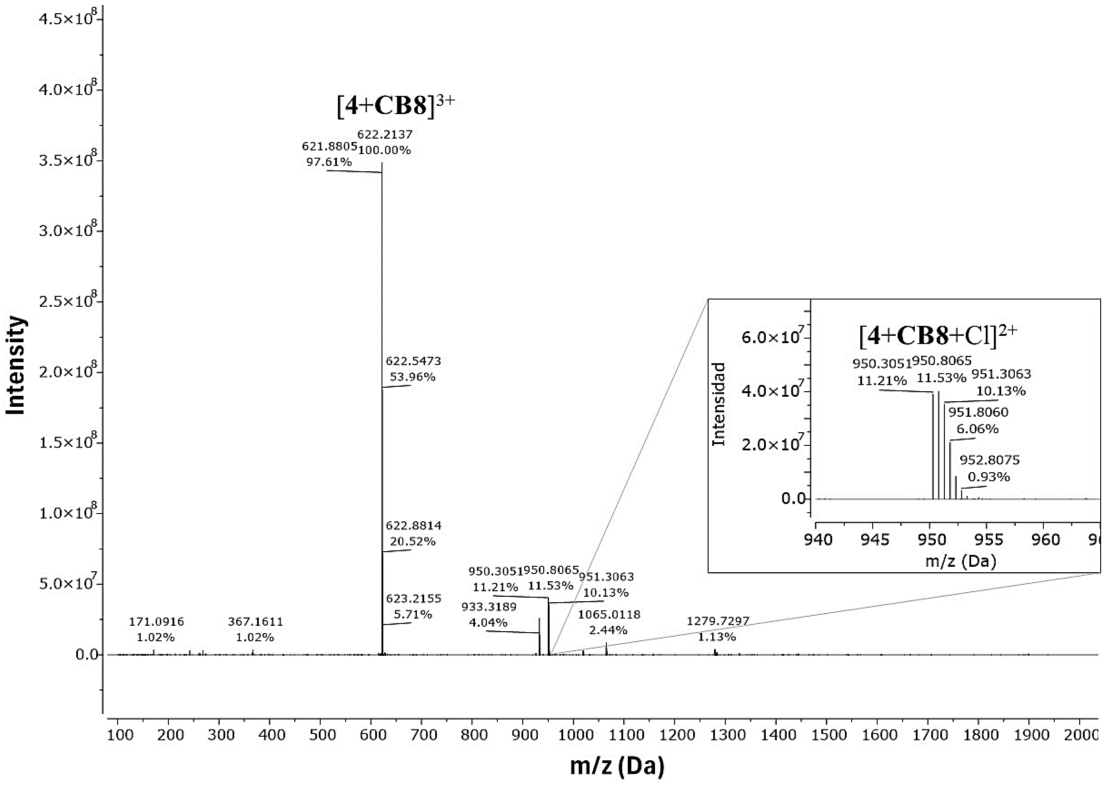

Figure S100: HRMS-ESI spectrum of $\mathbf{4}^{3+}$□**CB[8]**.

3.4. NMR study of the interaction of 5·2Br and CB[8]

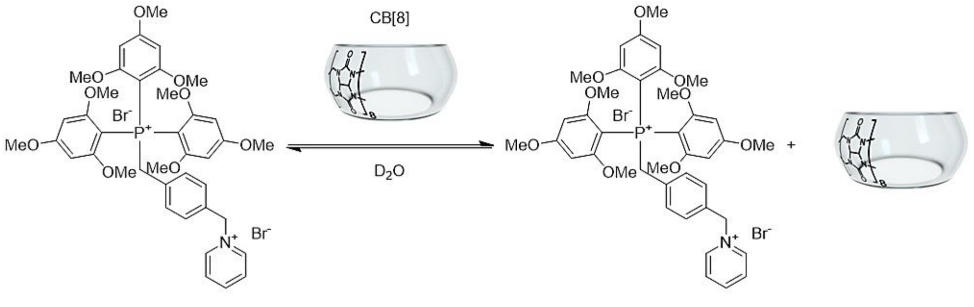

5·2Br **5**·2Br **CB[8]**

Firstly, a 2 mM stock solution of **5**·2Br in D_2O was prepared. To 2 mL of this solution was added CB[8] in excess and the mixture was heated and sonicated until saturation. The suspension was filtered (nylon, 0.2 µm) and a ^{1}H-NMR spectrum was registered.

^{1}H NMR (400 MHz, D_2O) *δ (ppm)*: 8.71 (t, *J* = 7.5 Hz, 2H), 8.66 – 8.53 (m, 1H), 8.15 – 8.03 (m, 2H), 7.26 (s, 3H), 6.23 (d, *J* = 4.7 Hz, 6H), 5.84 (d, *J* = 15.3 Hz, 1H), 5.66 (d, *J* = 4.3 Hz, 2H), 5.58 (s, 1H), 4.83 (d, *J* = 5.7 Hz, 2H), 4.24 (t, *J* = 14.7 Hz, 1H), 3.87 (s, 8H), 3.63 (s, 16H).

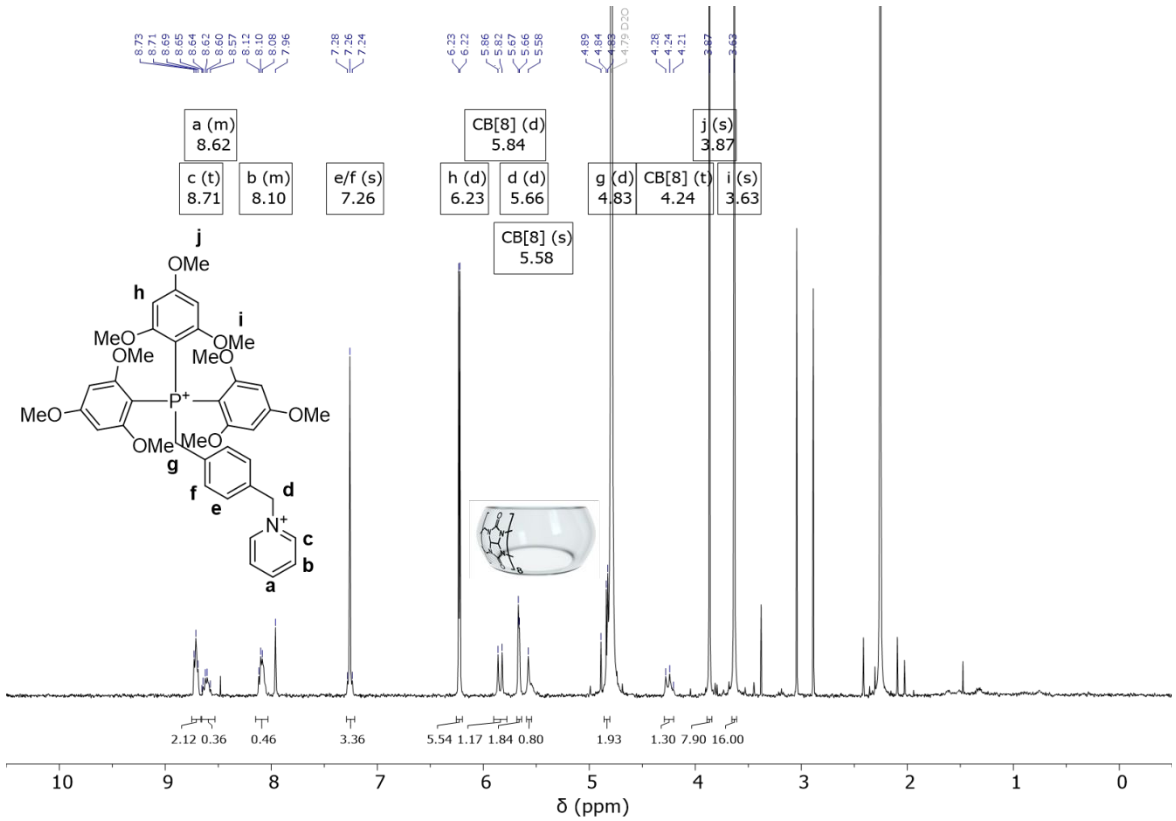

Figure S101: ^{1}H NMR (400 MHz, D_2O) spectrum of **5**·2Br and **CB[8]**.

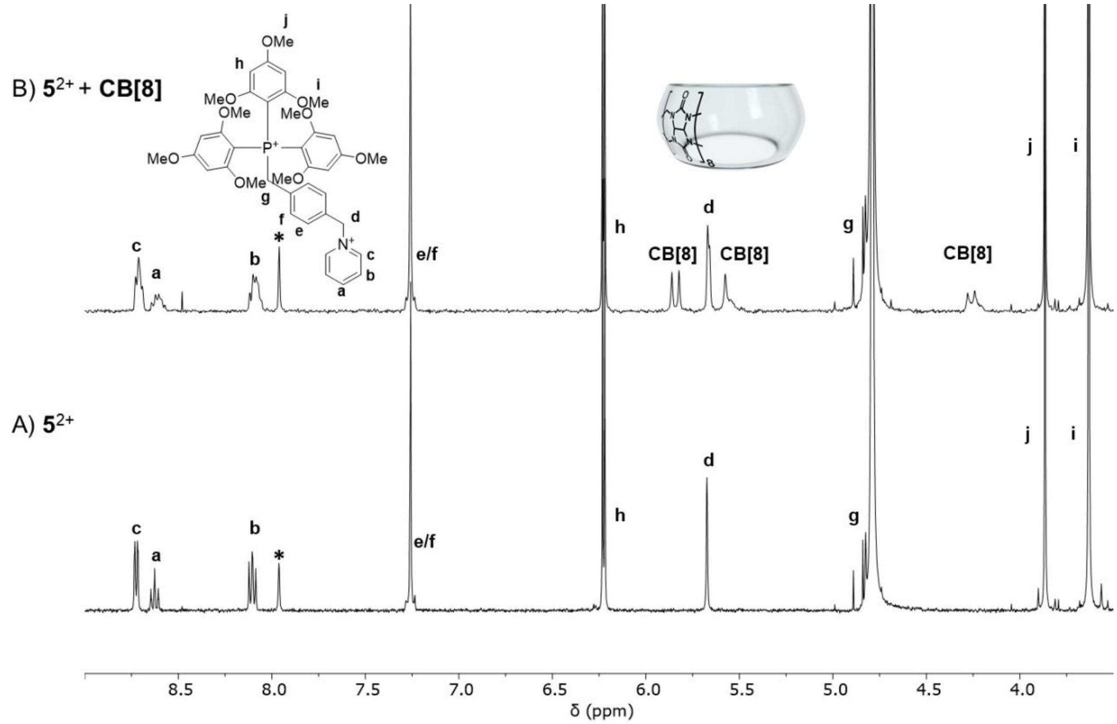

Figure S102: Partial ^{1}H NMR (500 MHz, D_2O) spectrum of: A) $\mathbf{5}^{2+}$; and B) $\mathbf{5}^{2+}$ + **CB[8].** Impurities are marked with ***.**

3.5. NMR study of the interaction between 6·I and CB[8]

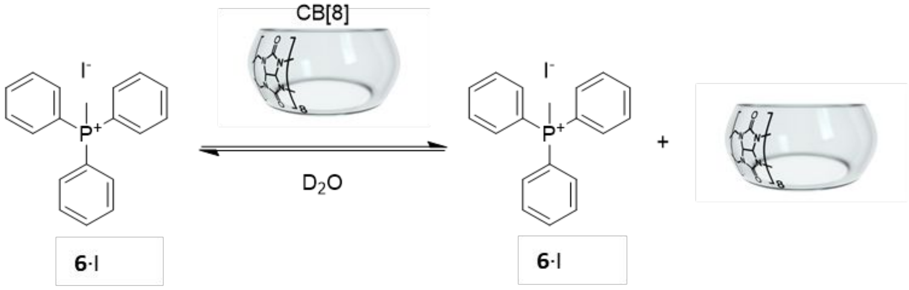

Same procedure described for **5**·2Br.

^{1}H NMR (400 MHz, D_2O) *δ (ppm)*: 7.90 – 7.81 (m, 3H), 7.78 – 7.62 (m, 12H), 5.80 (d, *J* = 15.3 Hz, 1H), 5.53 (s, 1H), 4.25 (t, *J* = 14.4 Hz, 1H), 2.88 (d, *J* = 13.9 Hz, 3H). **^{31}P{^{1}H} NMR** (162 MHz, D_2O) *δ (ppm)*: 21.10.

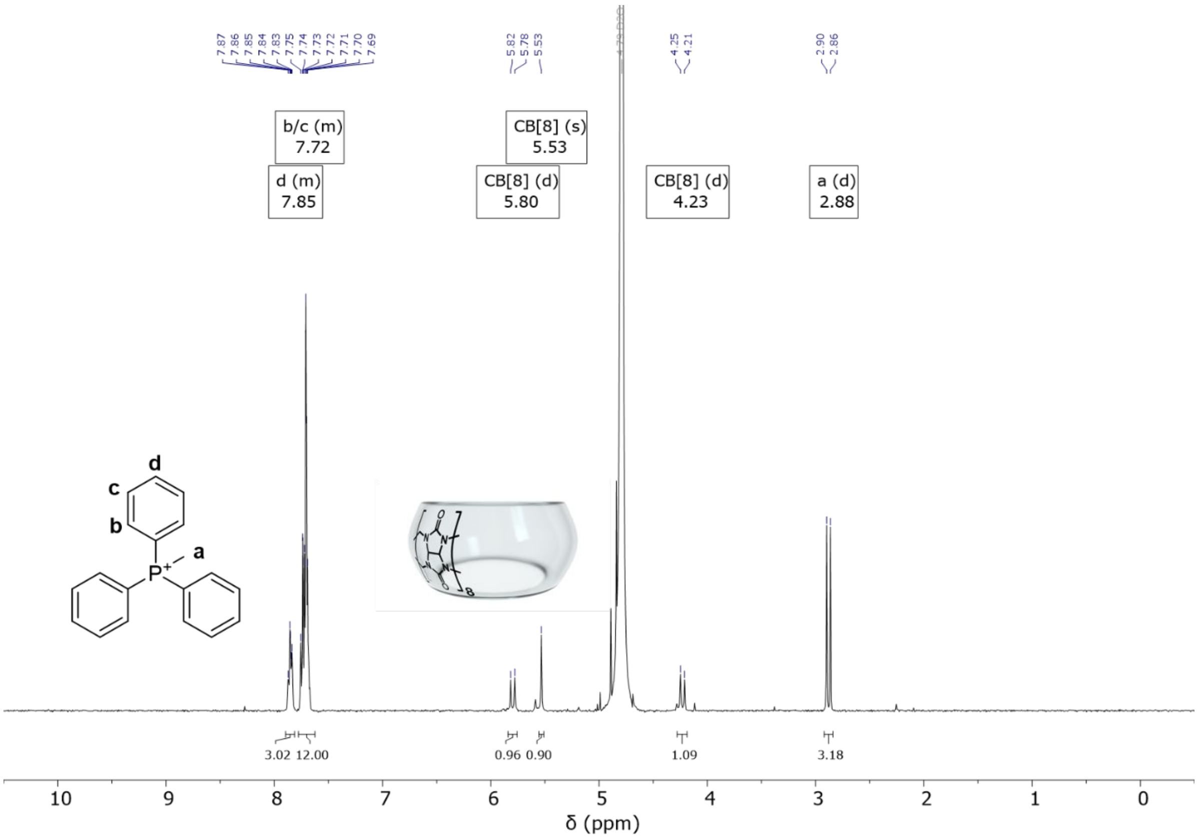

Figure S103: ^{1}H NMR (400 MHz, D_2O) spectrum of **6**·I and **CB[8]**.

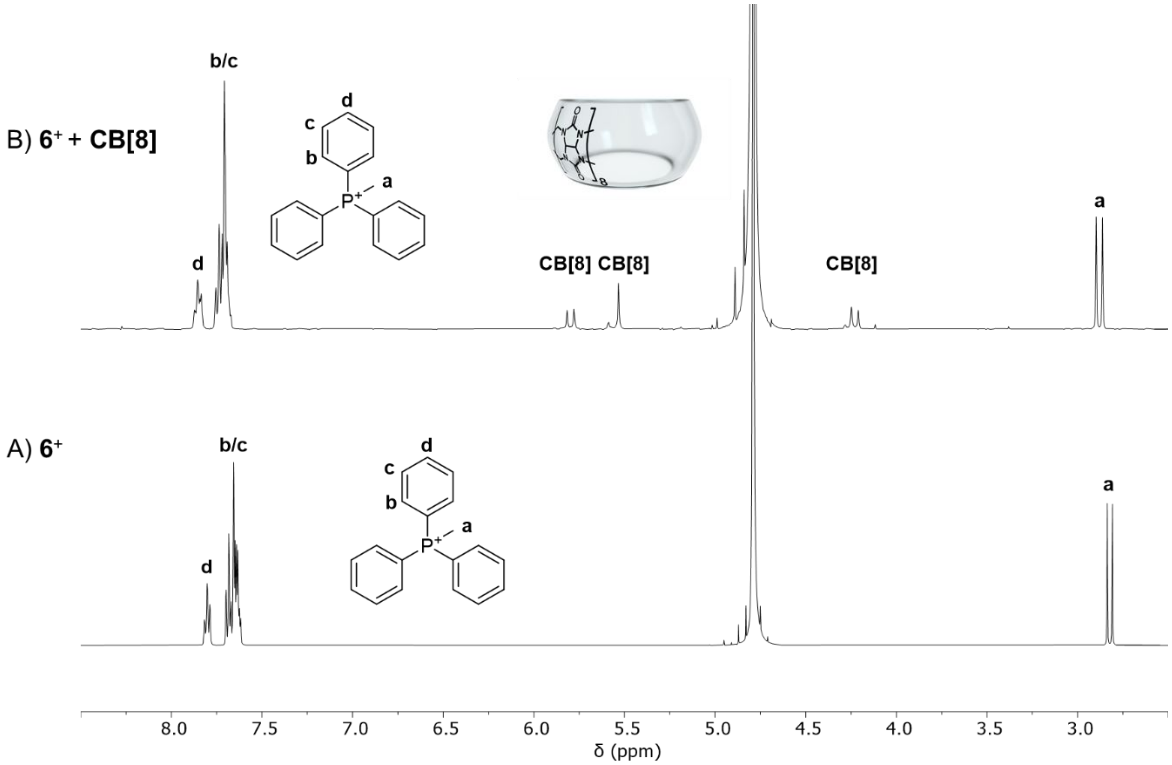

Figure S104: Partial ^{1}H NMR (500 MHz, D_2O) spectrum of: A) **6**$^{+}$; and B) **6**$^{+}$ + **CB[8].**

3.6. NMR study of the interaction between 7·Br and CB[8]

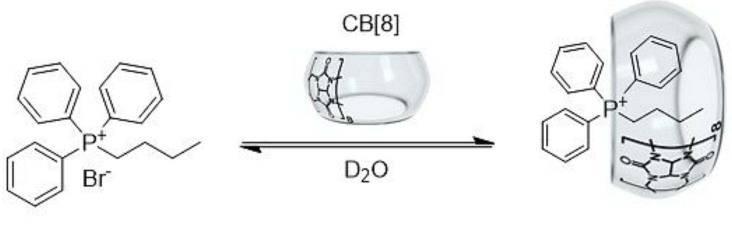

7·Br **7**$^{+}$⊂**CB[8]**

Same procedure described for **2**$^{2+}$□**CB[8]**.

^{1}H NMR (500 MHz, D$_2$O) *δ (ppm)*: 7.56 (d, *J* = 69.3 Hz, 15H), 5.75 (d, *J* = 15.3 Hz, 15H), 5.47 (s, 15H), 4.16 (d, *J* = 15.3 Hz, 14H), 2.72 (dt, *J* = 13.3, 6.0 Hz, 2H), 1.07 (s, 2H), 0.23 (d, *J* = 36.8 Hz, 1H), -0.05 (s, 3H). **^{13}C{^{1}H} NMR** (126 MHz, D$_2$O) *δ (ppm)*: 156.5, 134.0, 132.9, 129.6 (d, *J* = 12.6 Hz), 71.9, 53.5, 22.7 (d, *J* = 4.3 Hz), 21.9, 19.2, 18.8, 11.7. **^{31}P{^{1}H} NMR** (162 MHz, D$_2$O) *δ (ppm)*: 25.61.

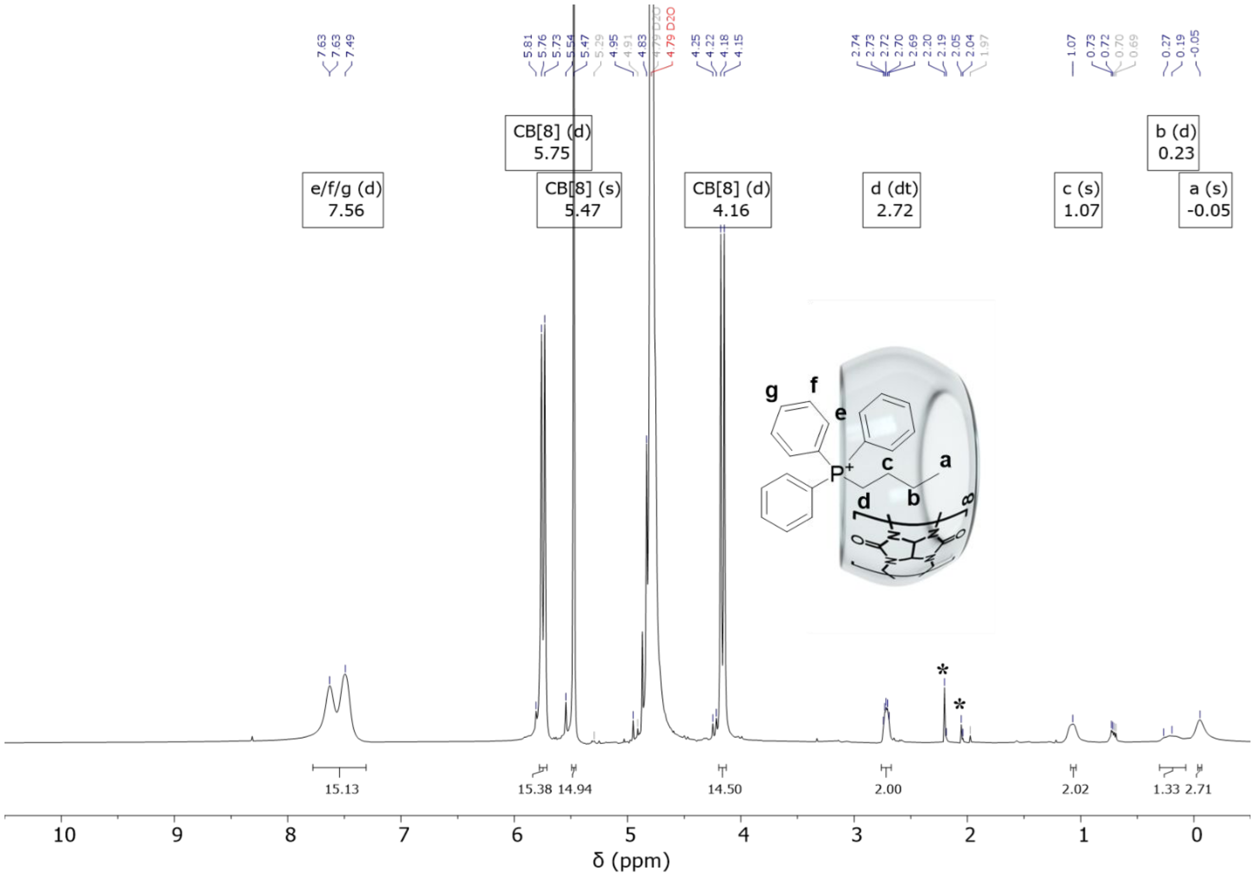

Figure S105: ^{1}H NMR (500 MHz, D$_2$O) spectrum of **7**$^{+}$□**CB[8]**. Impurities are marked with *****: CH_3CN (1.98 ppm, s) and acetone (2.22 ppm, s).

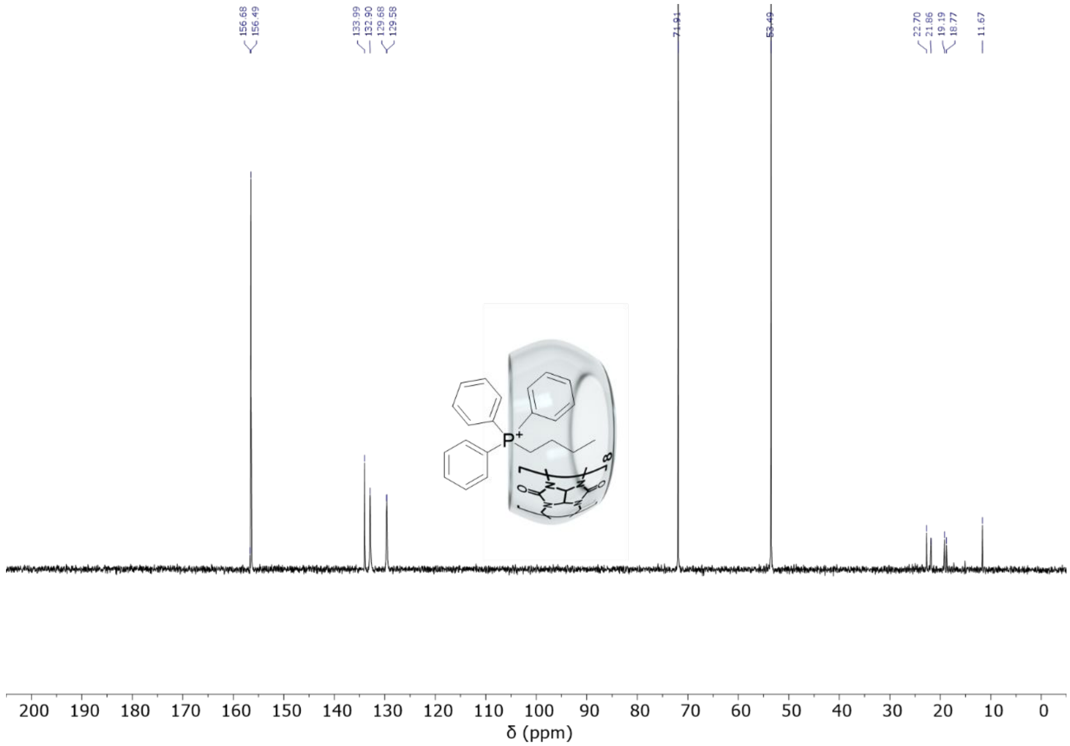

Figure S106: $^{13}C\{^{1}H\}$ NMR (126 MHz, D_2O) spectrum of 7^{+}□**CB[8]**.

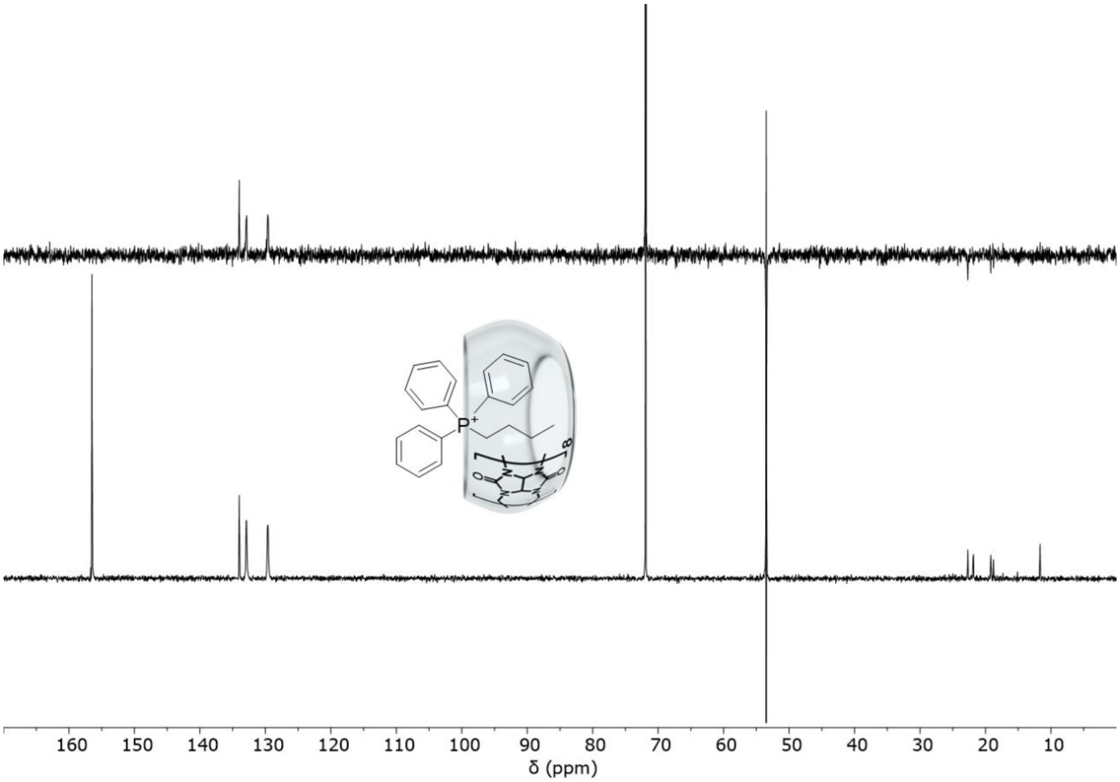

Figure S107: DEPT-135 (126 MHz, D_2O) spectrum (up) and $^{13}C\{^{1}H\}$ NMR (126 MHz, D_2O) spectrum (down) of **7^{+}□CB[8].**

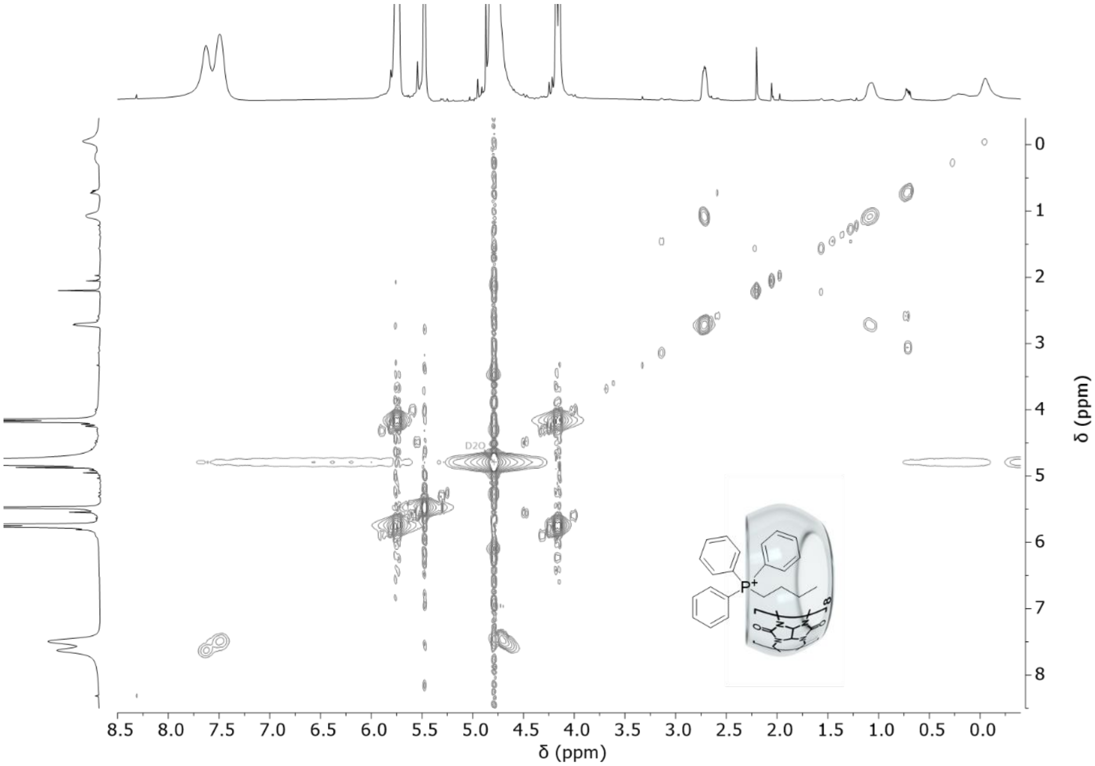

Figure S108: ^{1}H-^{1}H COSY (500 MHz, D_2O) spectrum of **7**$^{+}$□**CB[8]**.

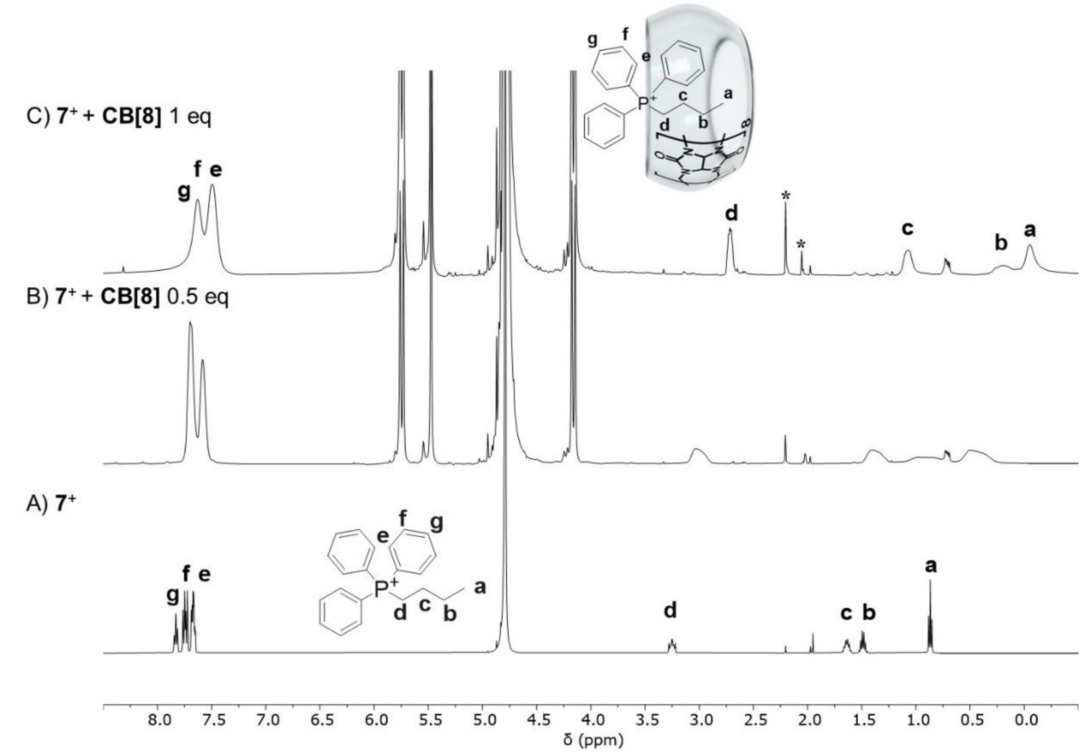

Figure S109: Partial ^{1}H NMR (400 MHz, D_2O) spectrum of: A) **7**$^{+}$; B) **7**$^{+}$ + 0.5 eq of **CB[8];** and C) **7**$^{+}$ + 1 eq of **CB[8]**. Impurities are marked with *****: CH_3CN (1.98 ppm, s) and acetone (2.22 ppm, s).

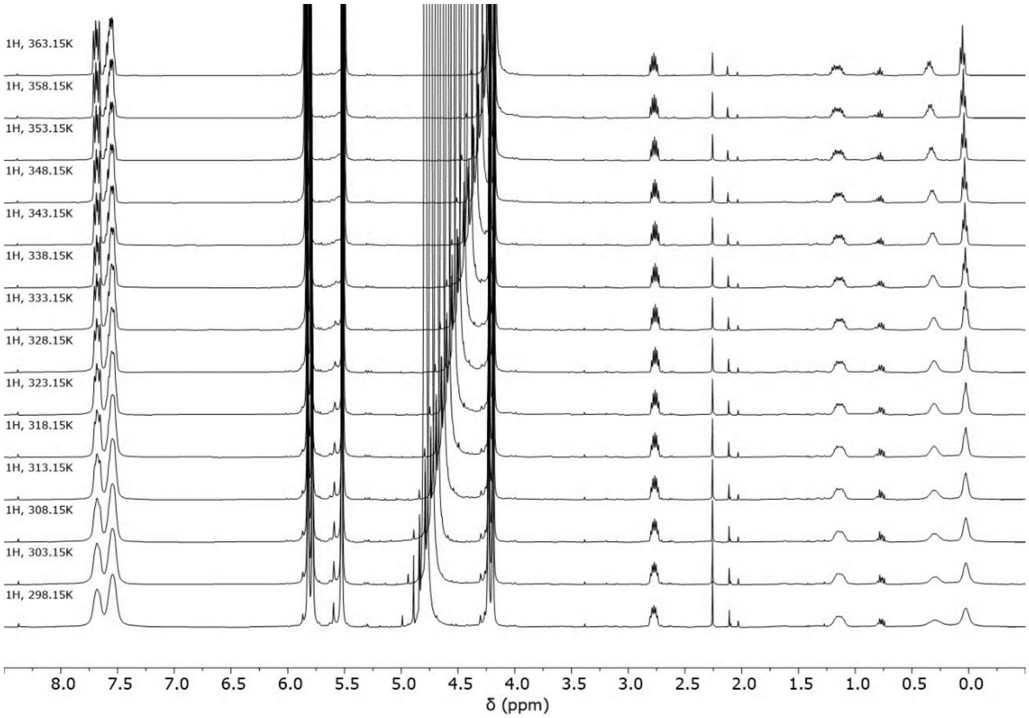

Figure S110: VT-^{1}H-NMR (400 MHz, D_2O) stacked spectra of **7^{+}□CB[8]**.

3.7. NMR study of the interaction between 8·Br and CB[8]

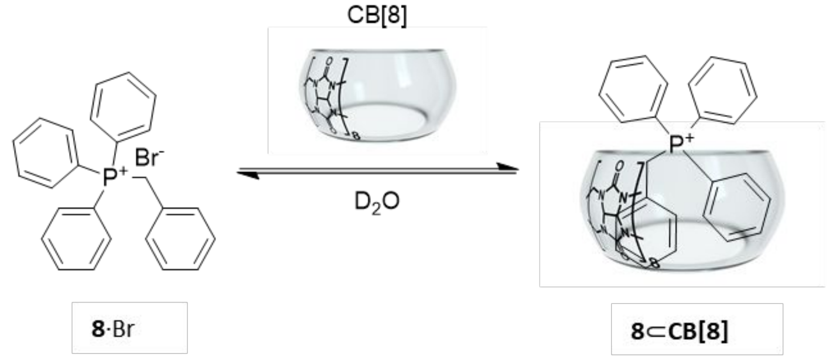

Same procedure described for **2**$^{2+}$□**CB[8]**.

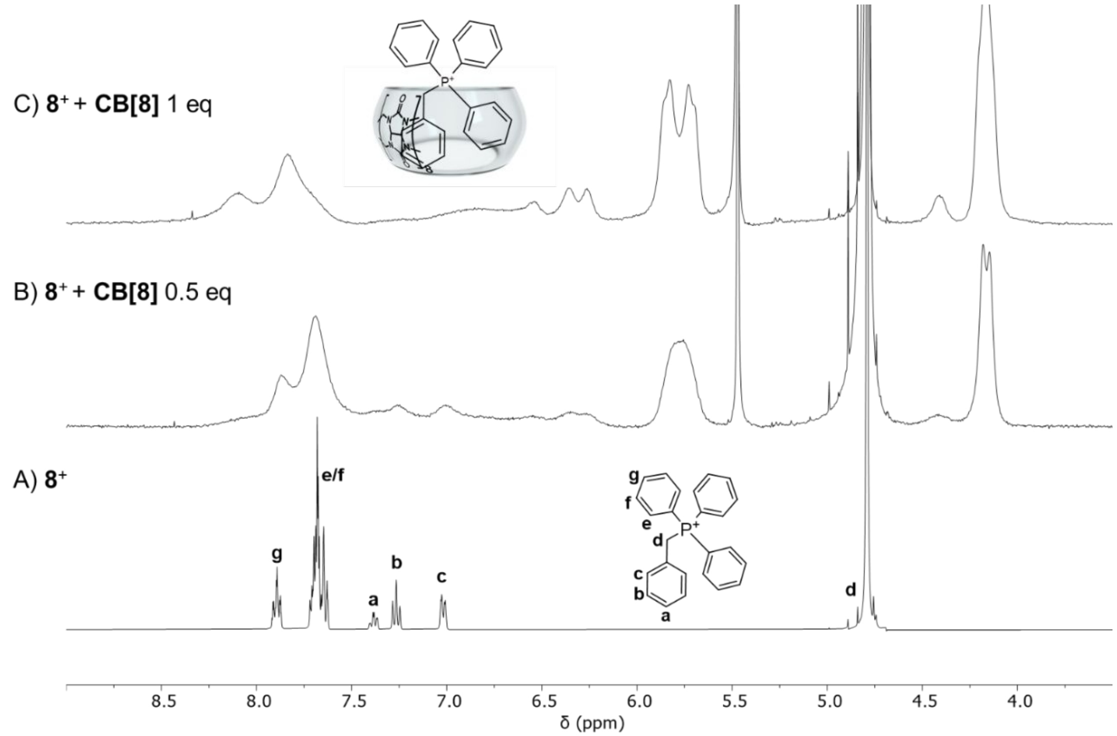

Figure S111: Partial ^{1}H NMR (400 MHz, D_2O) spectrum of: A) $\mathbf{8}^{+}$; B) $\mathbf{8}^{+}$ + 0.5 eq of **CB[8];** and C) $\mathbf{8}^{+}$ + 1 eq of **CB[8]**; at 298.15 K.

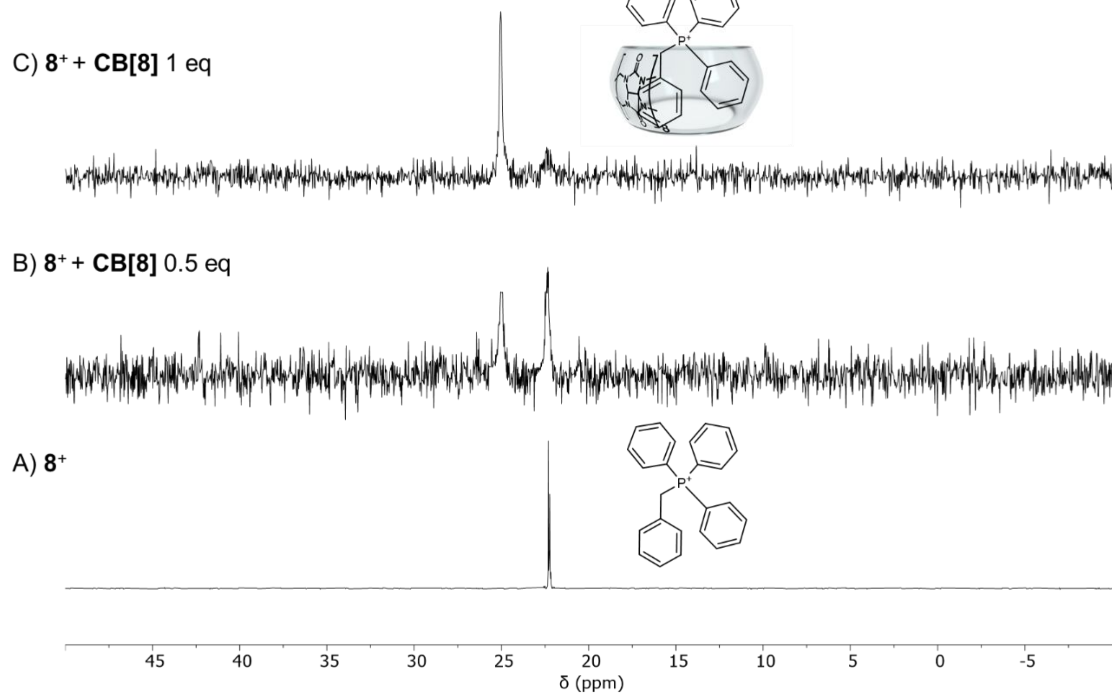

Figure S112: $^{31}P\{^1H\}$ NMR (162 MHz, D_2O) spectrum of: A) **8**$^+$; B) **8**$^+$ + 0.5 eq of **CB[8];** and C) **8**$^+$ + 1 eq of **CB[8]**; at 298.15 K.

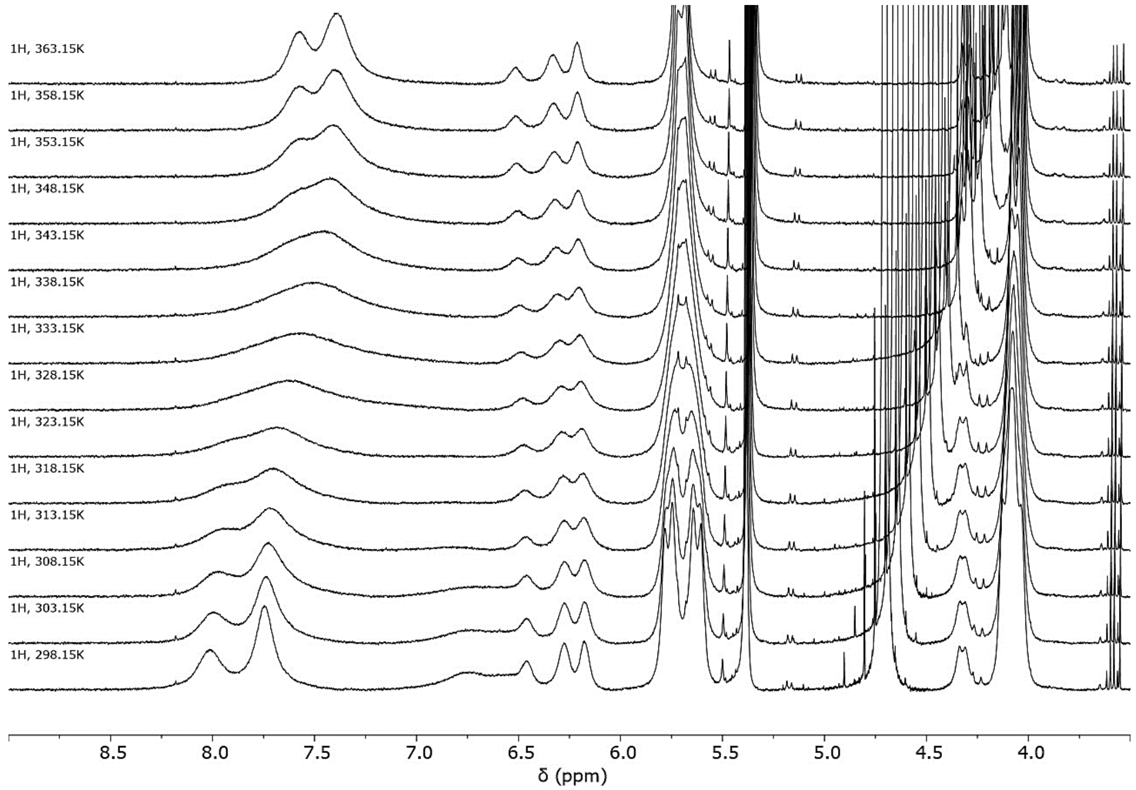

Figure S113: VT-^1H-NMR (400 MHz, D_2O) stacked spectra of **8**$^+$□**CB[8]**.

Due to the challenges in the characterization of the inclusion complex in D_2O at room temperature, we opted to modify the previous protocol using a stock solution of **8**$^+$ containing NaCl 1M. This adjustment allows the performance of NMR experiments at lower temperatures (near 0ºC).

^{1}H NMR (400 MHz, D_2O, 278.15 K) *δ (ppm)*: 7.93 (s, 4H), 7.65 (s, 6H), 6.70 (s, 2H), 6.51 (s, 1H), 6.44 (s, 2H), 6.21 (s, 1H), 6.12 (s, 4H), 5.61 (d, *J* = 15.3 Hz, 10H), 5.48 (d, *J* = 14.9 Hz, 10H), 5.40 (s, 16H), 4.29 (d, *J* = 13.0 Hz, 2H), 4.07 (d, *J* = 15.0 Hz, 17H).**^{31}P{^{1}H} NMR** (162 MHz, D_2O, 298 K) *δ (ppm)*: 25.07.

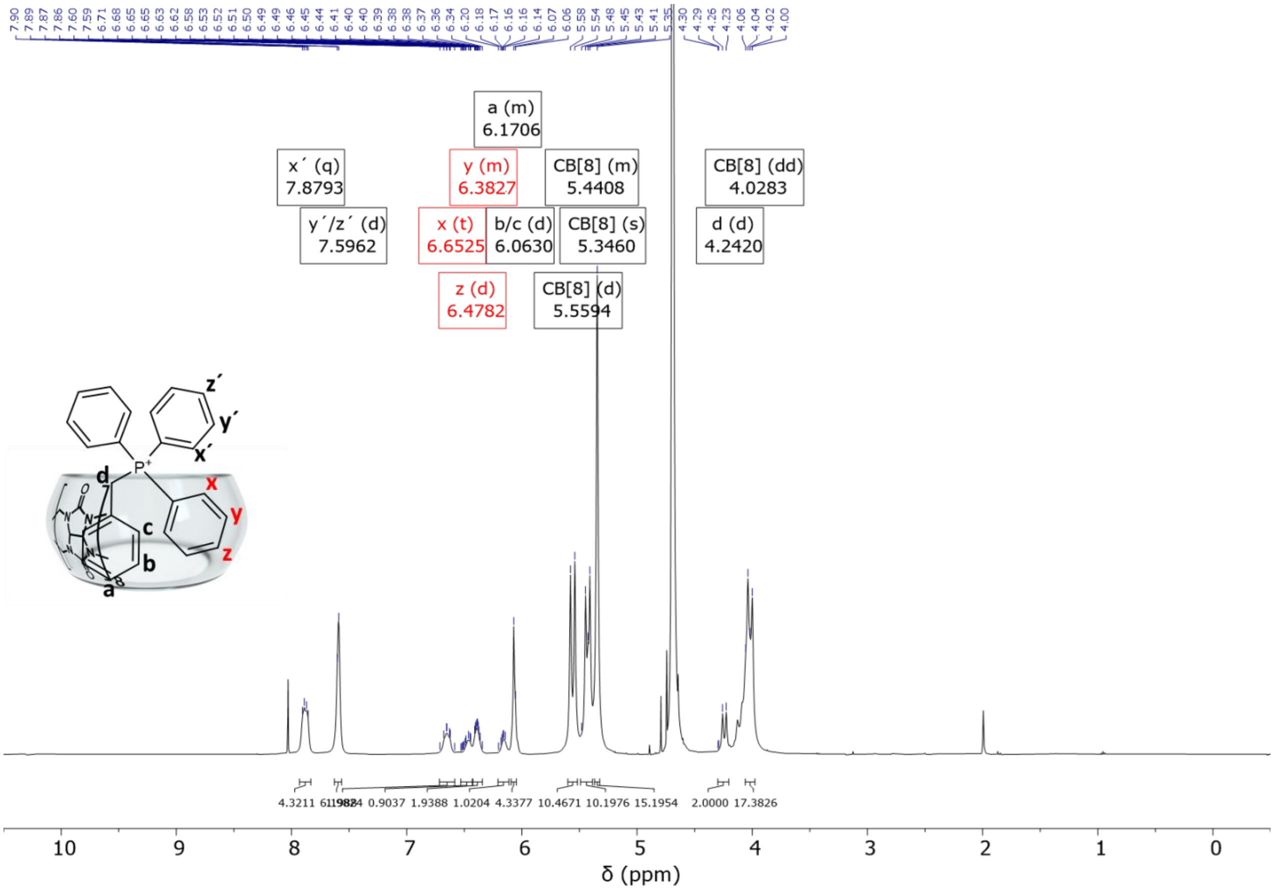

Figure S114: ^{1}H NMR (400 MHz, D_2O) spectrum of **8**$^+$□**CB[8]** (NaCl 1M) at 278.15 K.

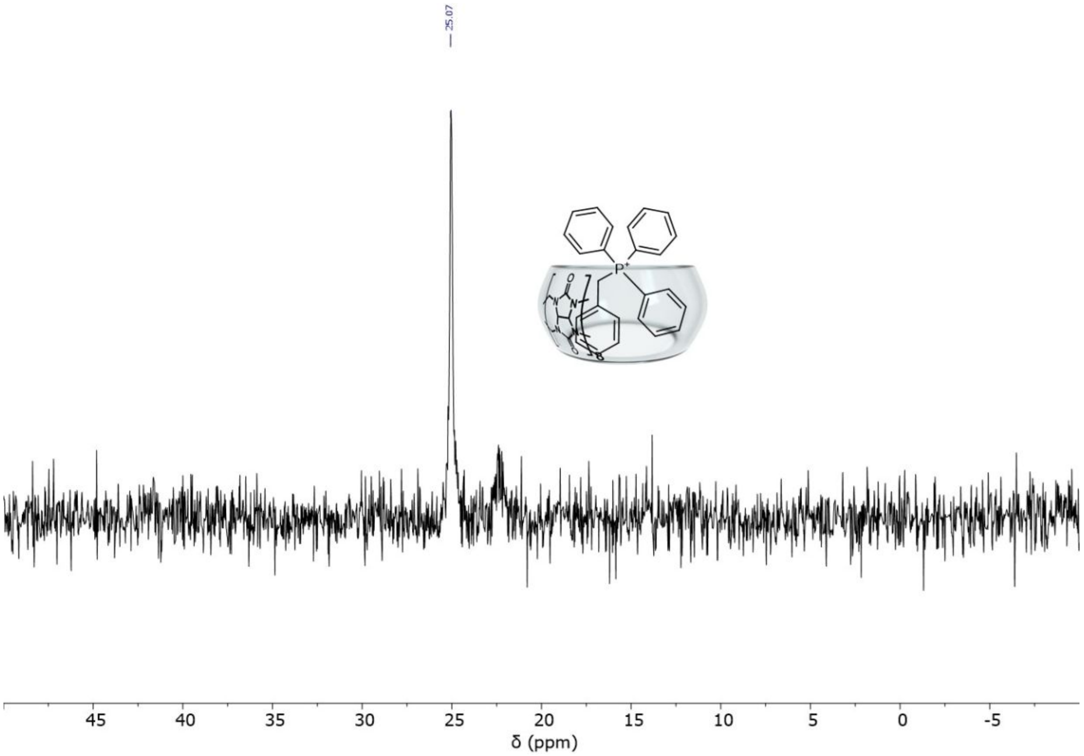

Figure S115: $^{31}P\{^1H\}$ NMR (162 MHz, D_2O, 298.15 K) spectrum of **8**$^+$□**CB[8]**.

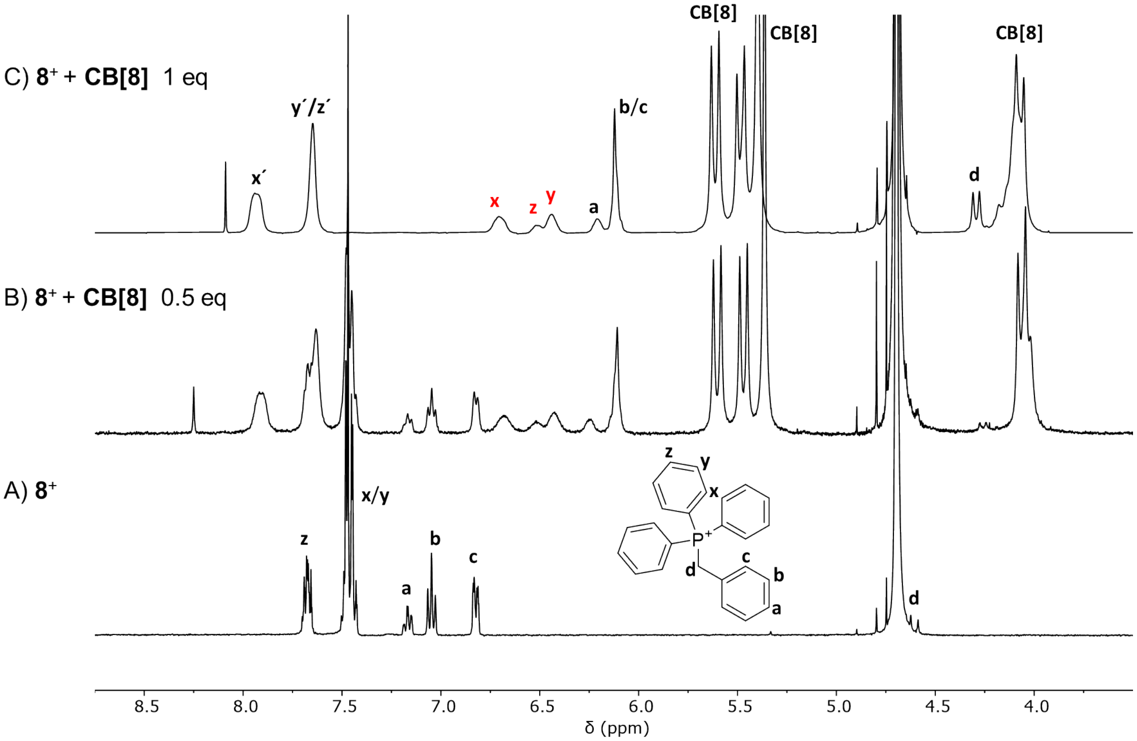

Figure S116: Partial ^1H NMR (400 MHz, D_2O, 278.15 K) spectrum of: A) **8**$^+$; B) **8**$^+$ + 0.5 eq of **CB[8];** and C) **8**$^+$ + 1 eq of **CB[8]**.

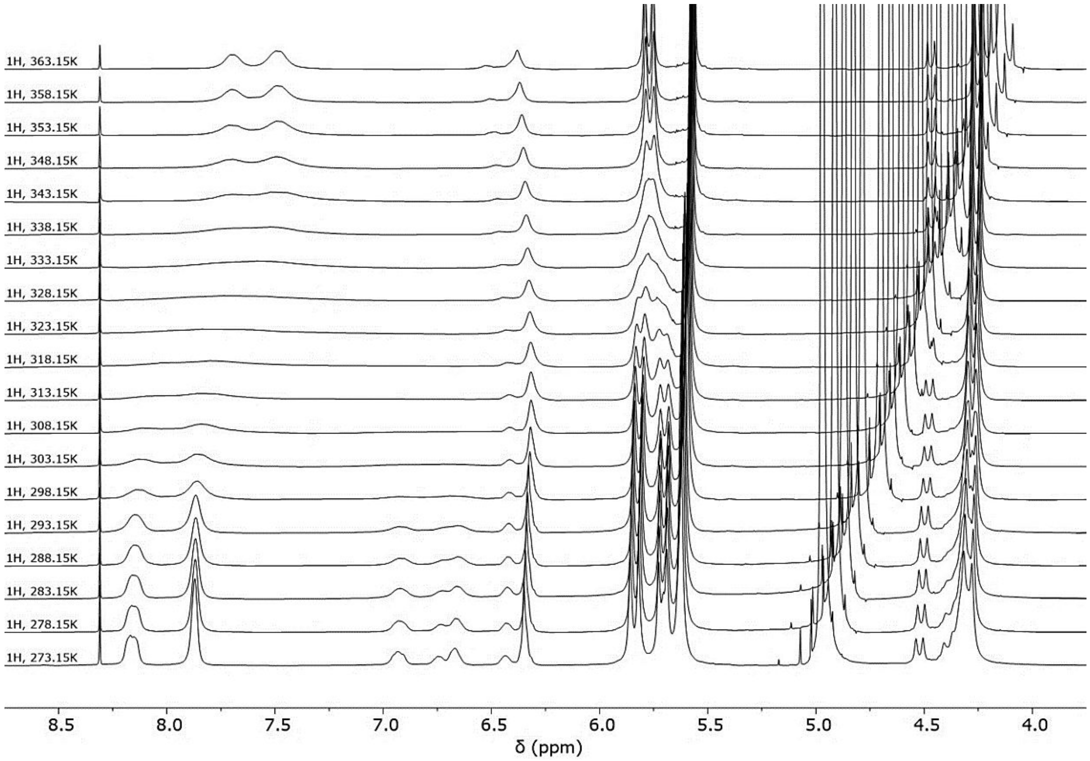

Figure S117: VT-^{1}H-NMR (400 MHz, D_2O) stacked spectra of **8⁺□CB[8]** in a 1M NaCl solution.

3.8. NMR study of the interaction between 2·2Br and CB[7]

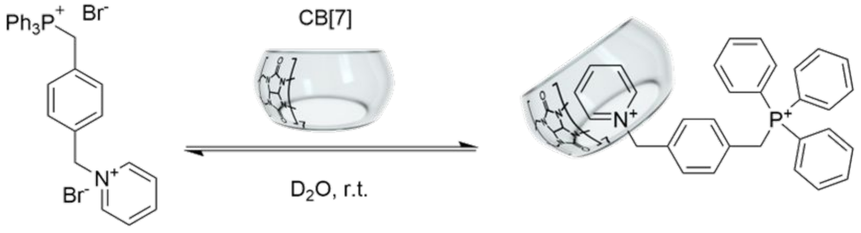

2·2Br   **2**$^{2+}$⊂**CB[7]**

300 µL of **2**·2Br at 2 mM and CB7 at 2 mM in D_2O are mixed at room temperature, reaching a concentration of 1 mM for both compounds. Subsequently, the spectra are recorded.

^{1}H NMR (400 MHz, D_2O) *δ (ppm)*: 8.11 – 8.01 (m, 3H), 7.94 – 7.85 (m, 3H), 7.79 – 7.67 (m, 12H), 7.56 – 7.38 (m, 2H), 7.24 (s, 4H), 5.77 (d, *J* = 15.4 Hz, 14H), 5.56 (s, 14H), 5.39 (s, 2H), 4.94 – 4.87 (m, 2H), 4.27 (d, *J* = 15.4 Hz, 14H).

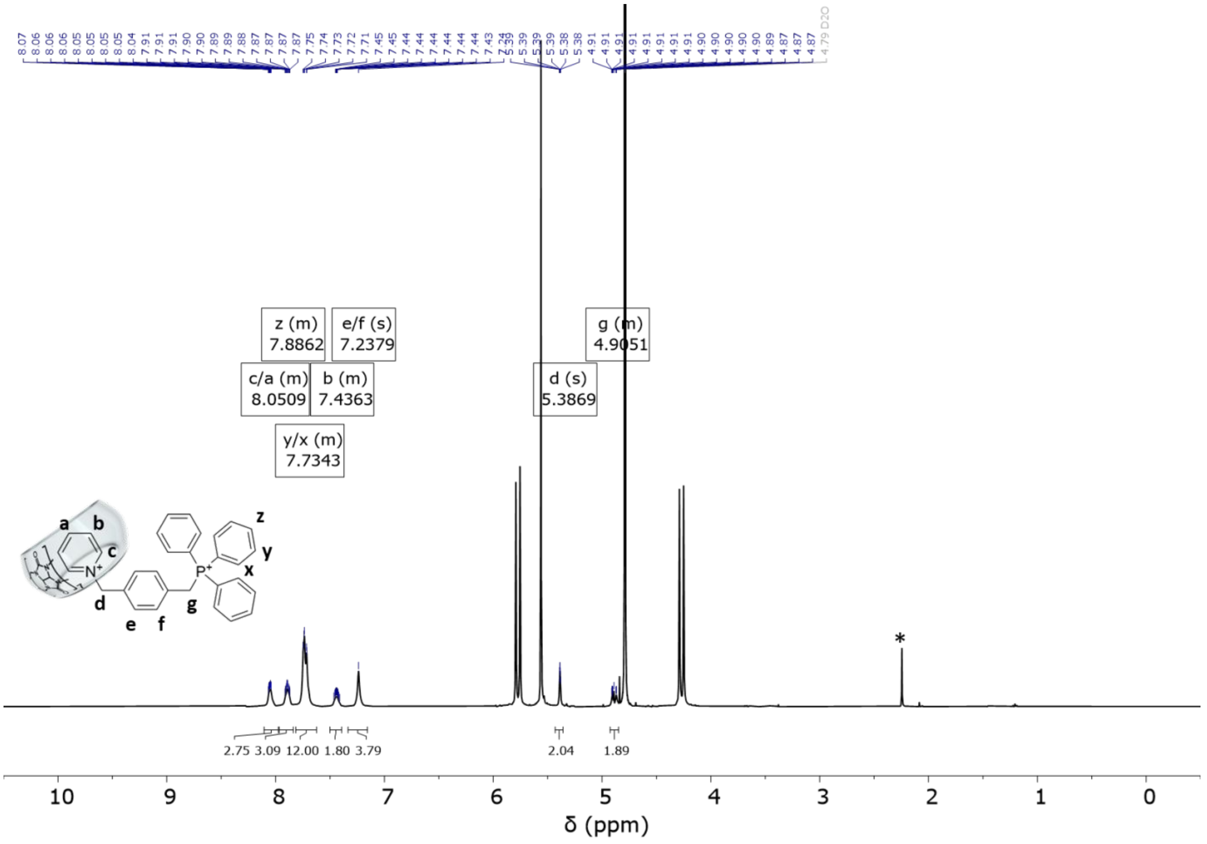

Figure S118: ^{1}H NMR (400 MHz, D_2O) spectrum of **2**$^{2+}$□**CB[7]**. Impurities are marked with *****: acetone (2.22 ppm, s).

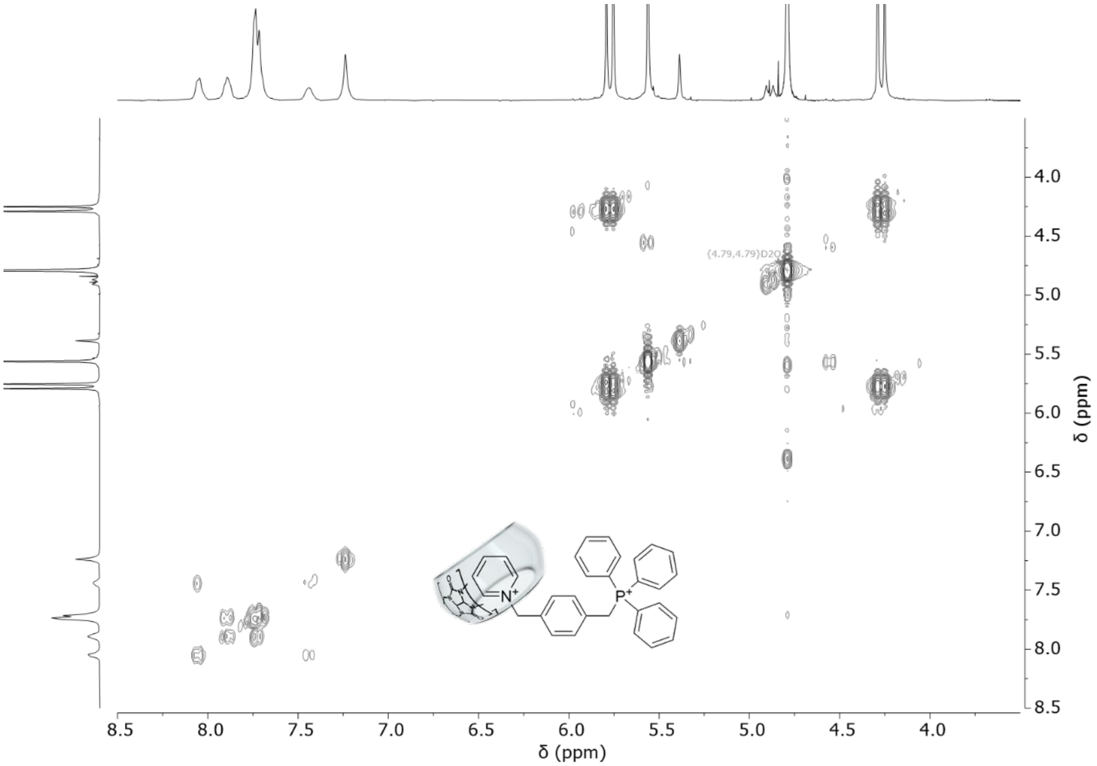

Figure S119: ^{1}H-^{1}H COSY (500 MHz, D_2O) spectrum of **2**$^{2+}$⊂**CB[7]**.

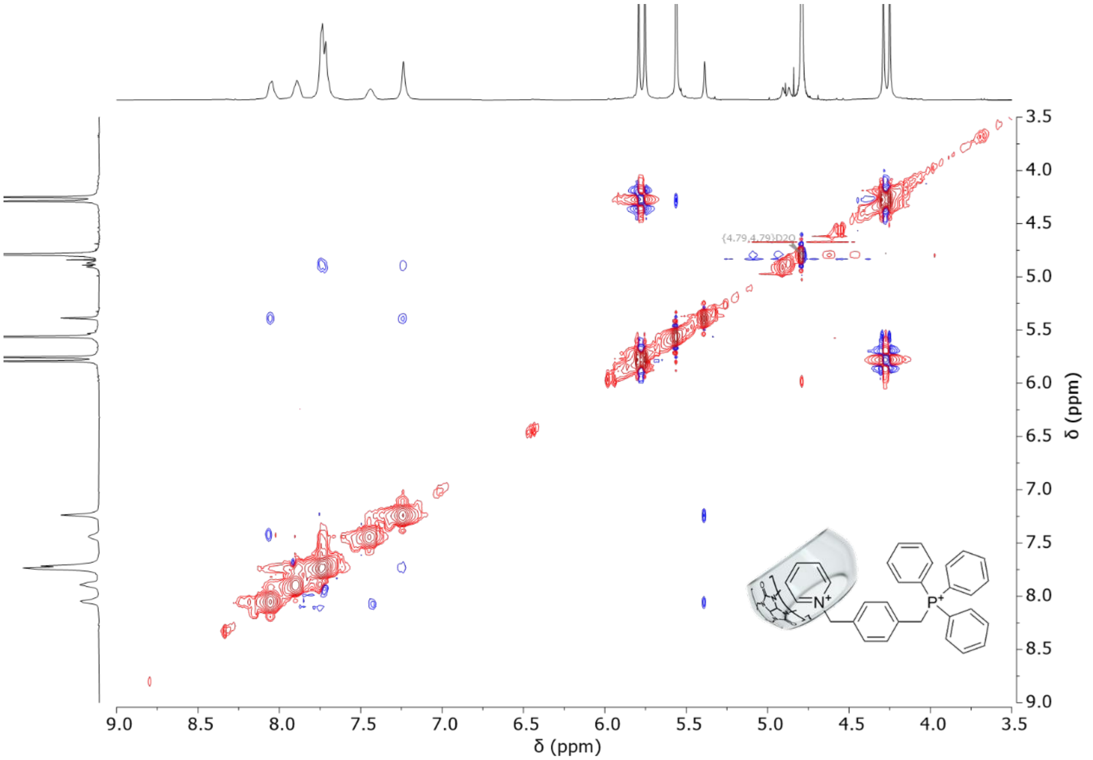

Figure S120: ^{1}H-^{1}H NOESY (400 MHz, D_2O) spectrum of **2**$^{2+}$⊂**CB[7]**. Blue cross peaks indicate NOE correlations.

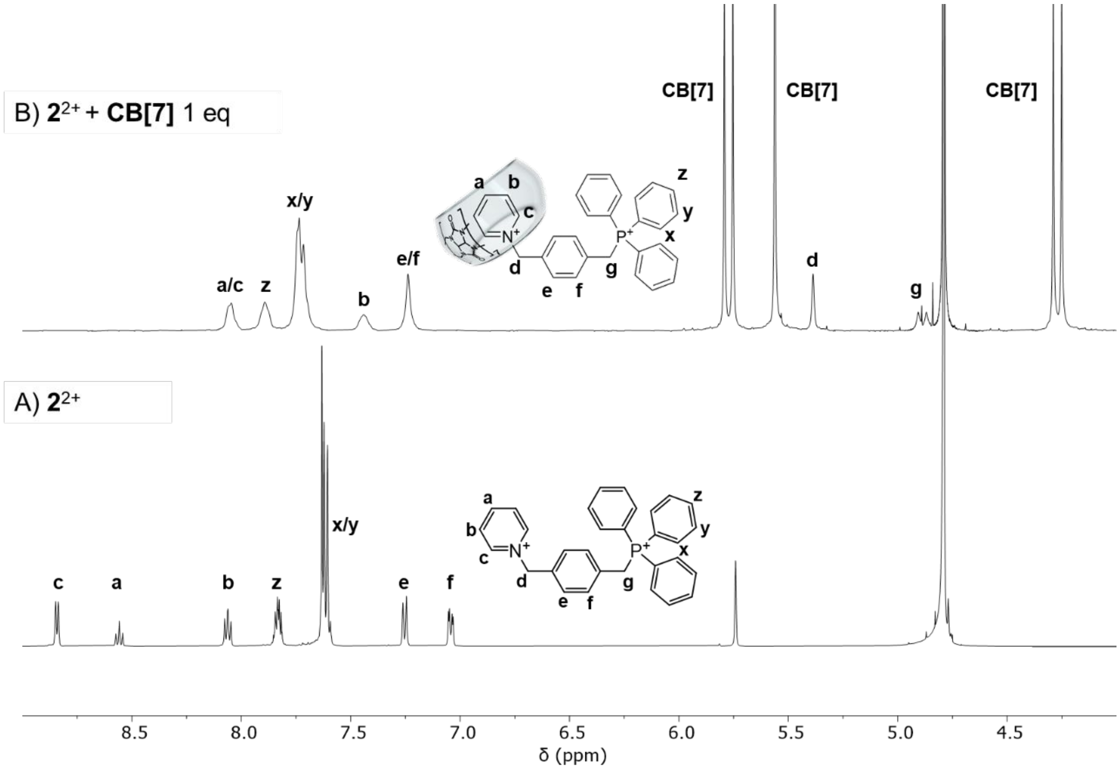

Figure S121: Partial ^{1}H NMR (400 MHz, D_2O) spectrum of: A) **2**$^{2+}$; B) **2**$^{2+}$ + 1 eq of **CB[7]**.

3.9. NMR study of the interaction between 3H·2Br/3·Br and CB[7]

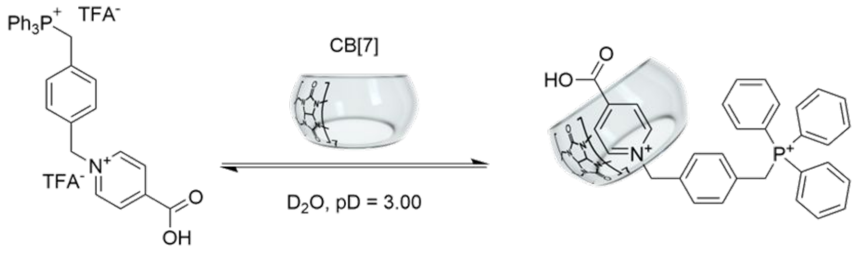

3H·2TFA   3H^{2+}⊂**CB[7]**

For the study with **3**H^{2+}, two stock solutions of **3**H·2TFA and CB7 at 2 mM are prepared in phosphate buffer 20 mM at pD3. 300 μL of both solutions are mixed at room temperature, reaching a final concentration of 1 mM for both compounds. Subsequently, the spectra are recorded.

^{1}H NMR (500 MHz, D_2O) *δ (ppm)*: 8.92 – 8.81 (m, 2H), 8.27 (s, 2H), 7.94 – 7.80 (m, 3H), 7.66 (s, 12H), 7.37 – 7.27 (m, 2H), 7.18 – 7.06 (m, 2H), 5.82 (s, 2H), 5.75 (s, 14H), 5.54 (s, 14H), 4.24 (d, *J* = 15.5 Hz, 14H).

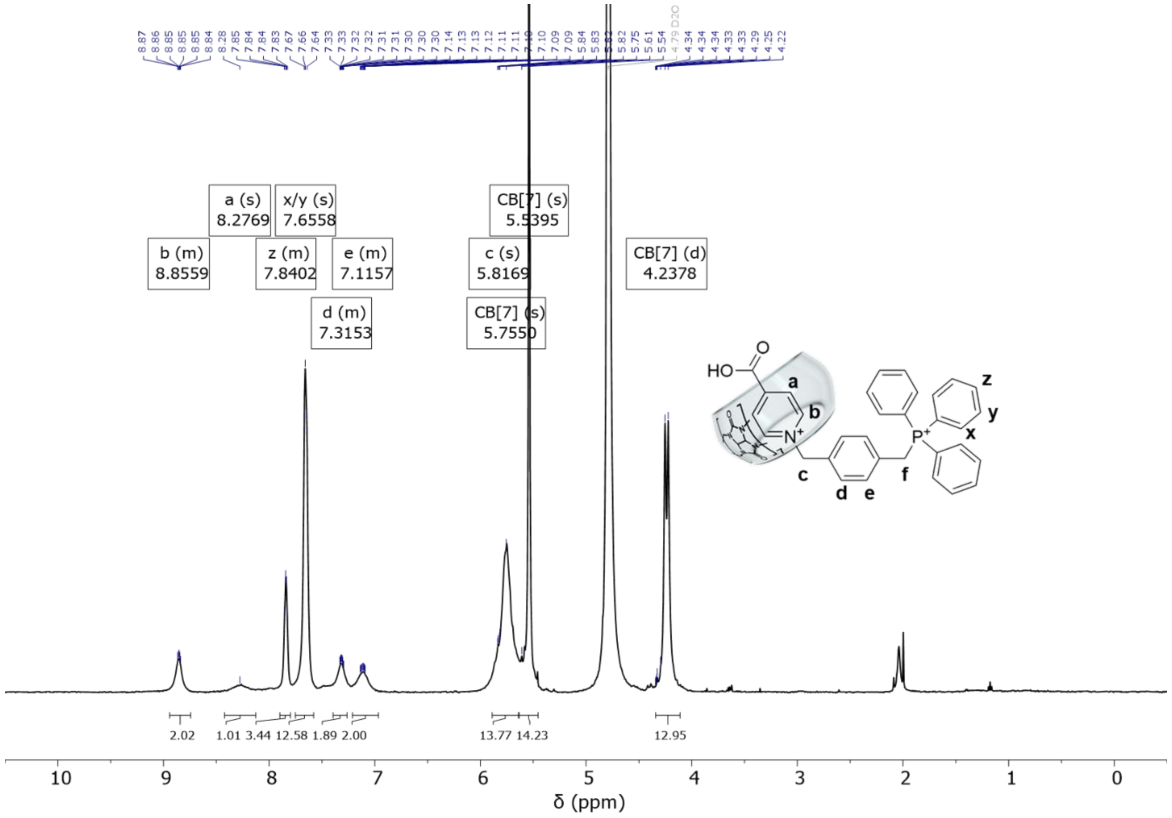

Figure S122: ^{1}H NMR (500 MHz, D_2O) spectrum of **3**H^{2+}□**CB[7]**.

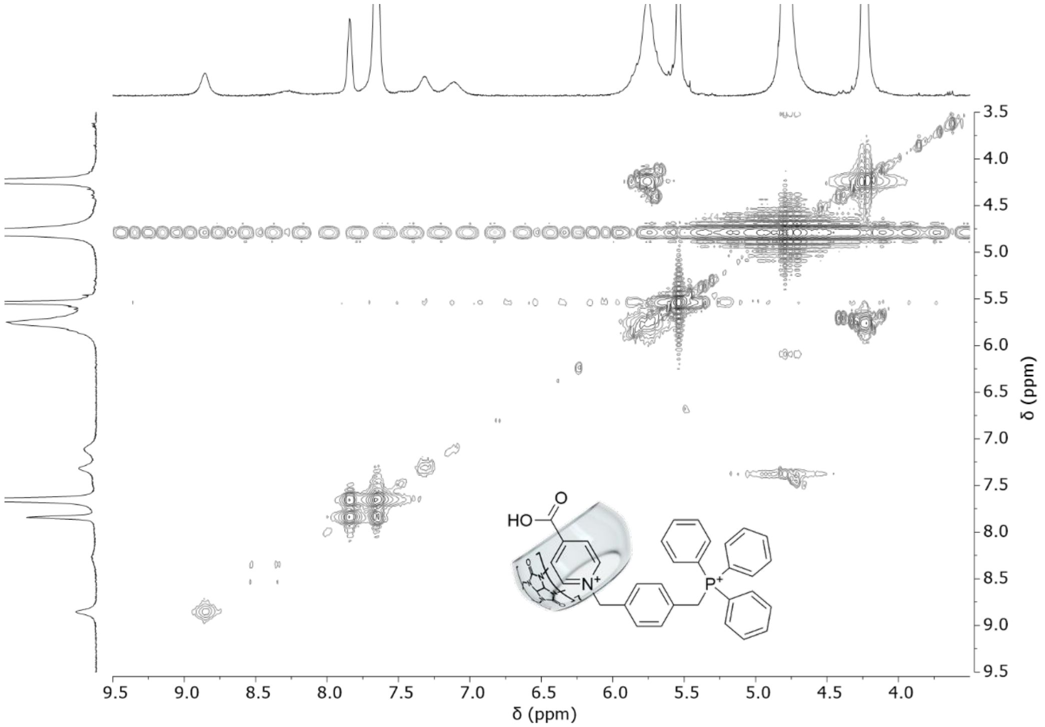

Figure S123: ^{1}H-^{1}H COSY (500 MHz, D_2O) spectrum of **3**H^{2+}□**CB[7]**.

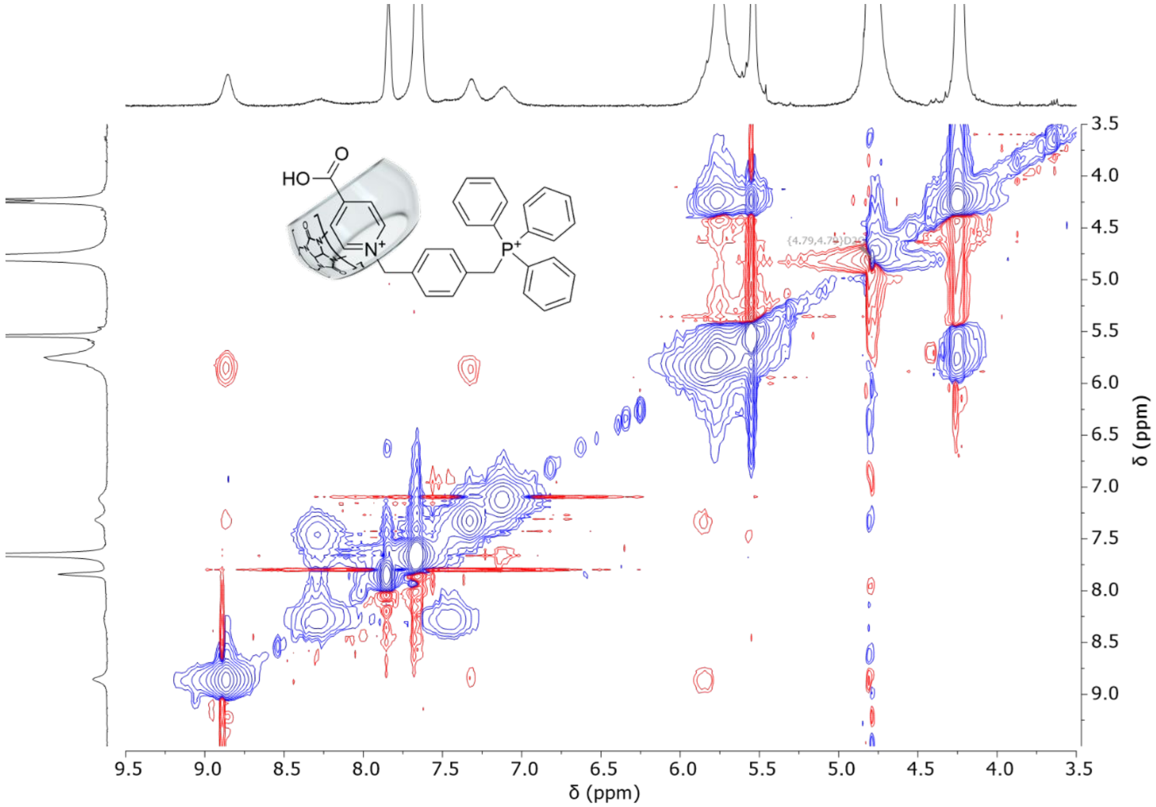

Figure S124: ^{1}H-^{1}H NOESY (400 MHz, D_2O) spectrum of **3**H^{2+}□**CB[7]**. Red cross peaks indicate NOE correlations.

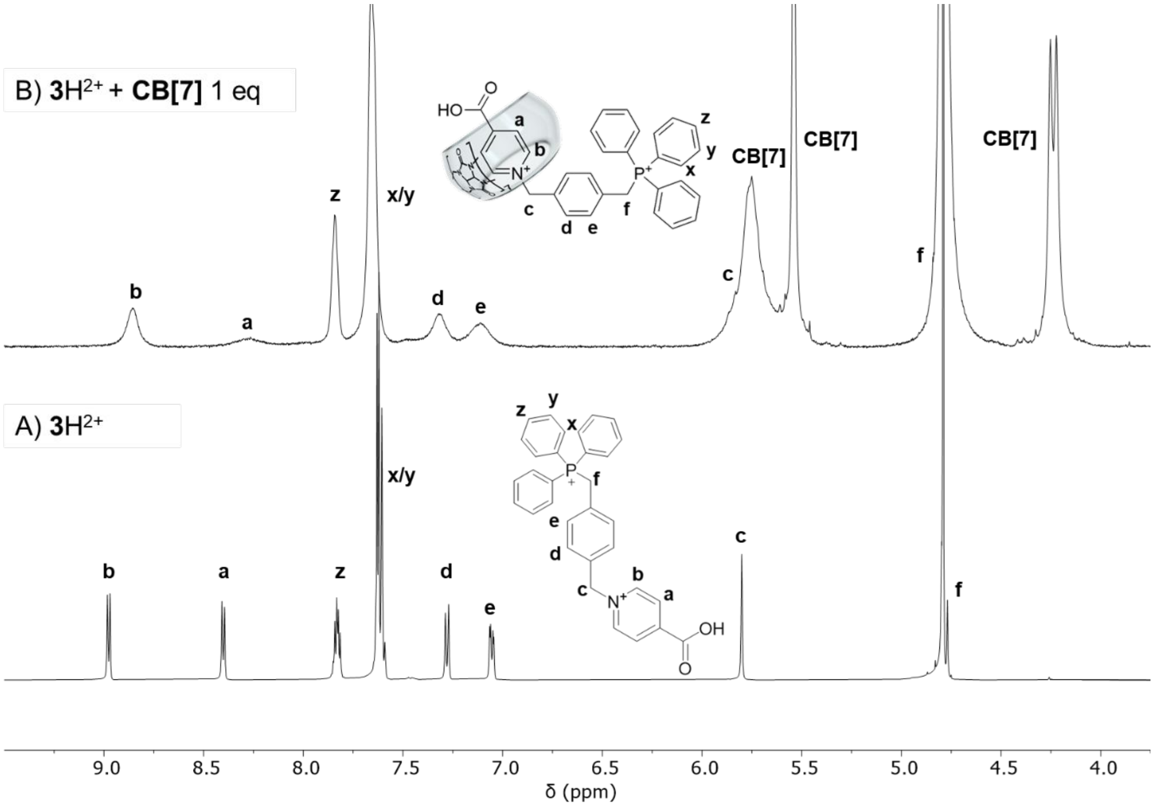

Figure S125: Partial ^{1}H NMR (500 MHz, D_2O) spectrum of: A) **3**H^{2+}; B) **3**H^{2+} + 1 eq of **CB[7]**.

For the study with **3**$^{+}$, the same procedure used at pD 3 is employed, but using phosphate buffer 20 mM at pD 12.

^{1}H NMR (300 MHz,D_2O) *δ (ppm)*: 8.89 (d, *J* = 6.6 Hz, 1H), 8.27 (d, *J* = 5.9 Hz, 1H), 7.82 (d, *J* = 7.6 Hz, 1H), 7.66 – 7.56 (m, 7H), 7.31 – 7.19 (m, 0H), 7.06 – 6.94 (m, 0H), 5.77 (d, *J* = 15.5 Hz, 13H), 5.57 (s, 12H), 5.48 (d, *J* = 9.0 Hz, 1H), 4.26 (d, *J* = 15.4 Hz, 13H).

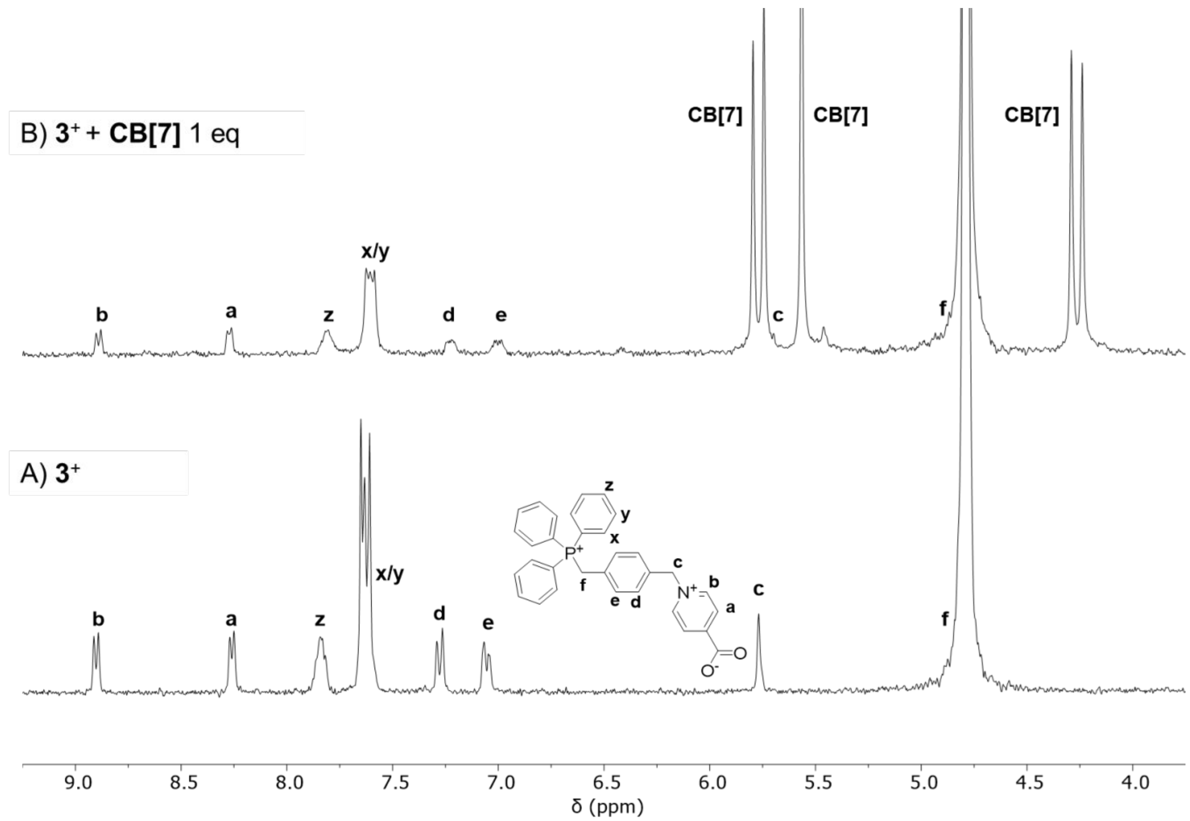

Figure S126: Partial ^1H NMR (300 MHz, D_2O) spectrum of: A) **3**$^+$; B) **3**$^+$ + 1 eq of **CB[7]** at pD = 12.00.

3.10. NMR study of the interaction between **4**·3Cl and CB[7]

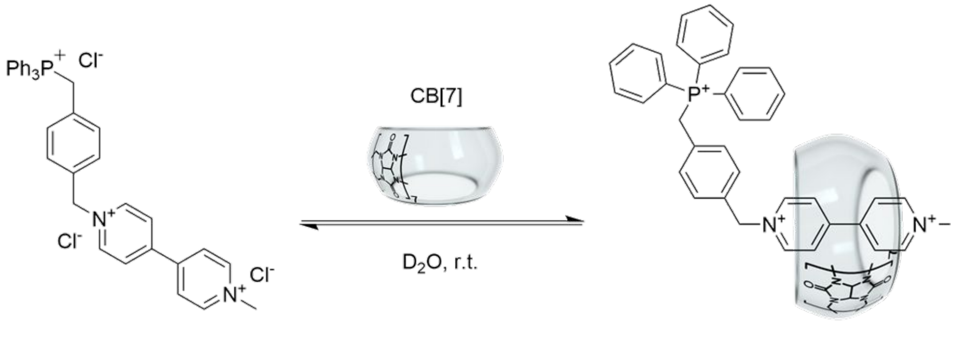

4·3Cl $\qquad$ **4**$^{3+}$⊂**CB[8]**

The same procedure used for **2**·2Br is carried out.

^{1}H NMR (400 MHz, D_2O) *δ (ppm)*: 9.12 – 9.01 (m, 2H), 8.97 – 8.84 (m, 2H), 7.88 (dq, *J* = 6.8, 2.3 Hz, 3H), 7.77 – 7.64 (m, 12H), 7.60 – 7.51 (m, 2H), 7.32 – 7.19 (m, 2H), 7.10 – 7.02 (m, 2H), 7.01 – 6.91 (m, 2H), 6.14 – 6.02 (m, 2H), 5.74 (d, *J* = 15.5 Hz, 7H), 5.60 (d, *J* = 15.4 Hz, 7H), 5.54 (s, 14H), 4.99 – 4.86 (m, 2H), 4.67 – 4.61 (m, 2H), 4.24 (dd, *J* = 21.6, 15.4 Hz, 14H).

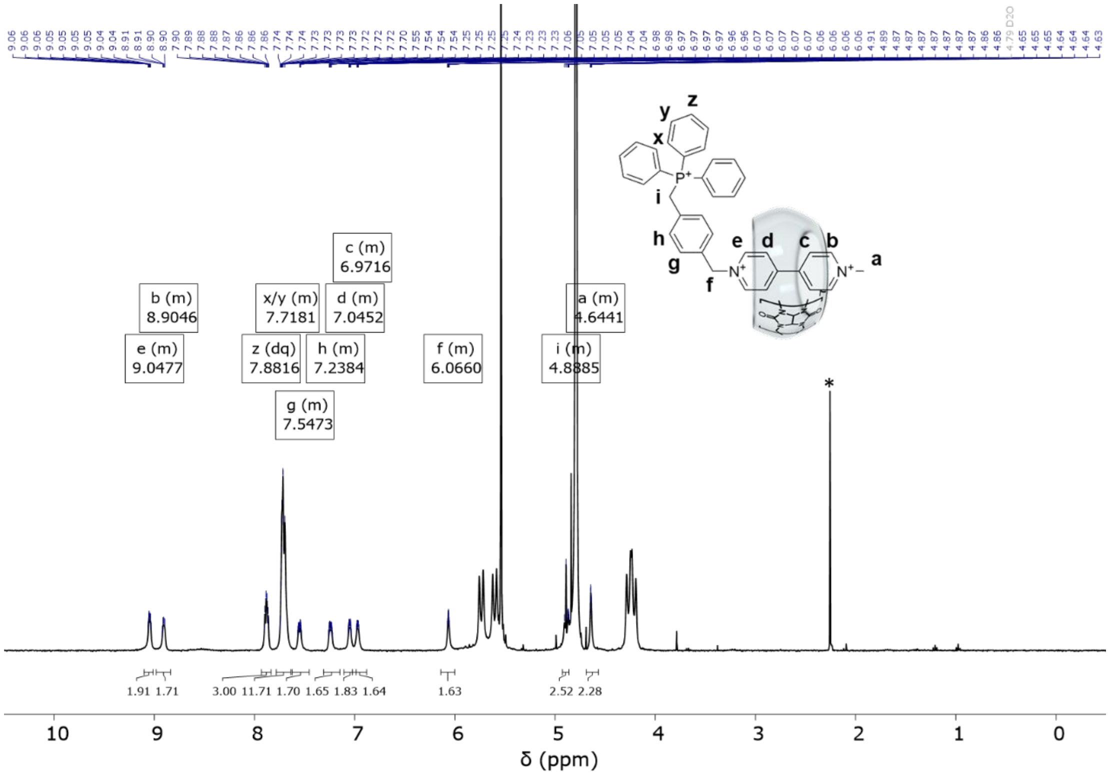

Figure S127: ^{1}H NMR (400 MHz, D_2O) spectrum of **4**$^{3+}$□**CB[7]**. Impurities are marked with *****: acetone (2.22 ppm, s).

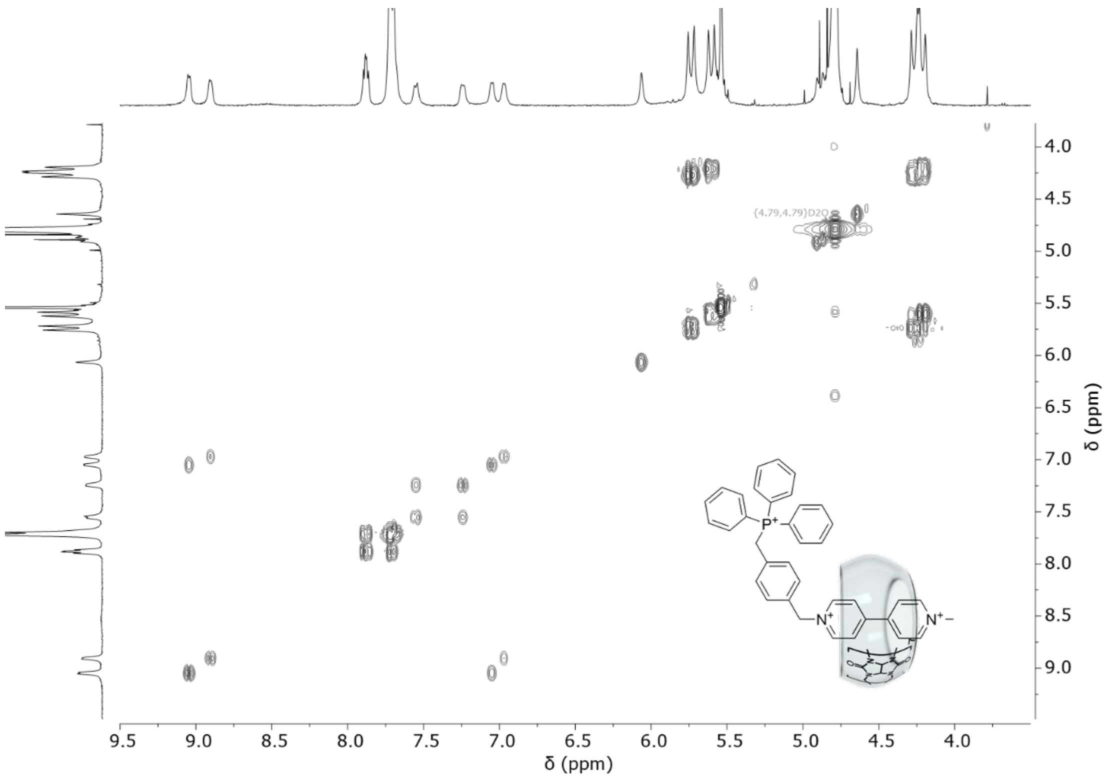

Figure S128: ^{1}H-^{1}H COSY (400 MHz, D_2O) spectrum of **4^{3+}□CB[7]**.

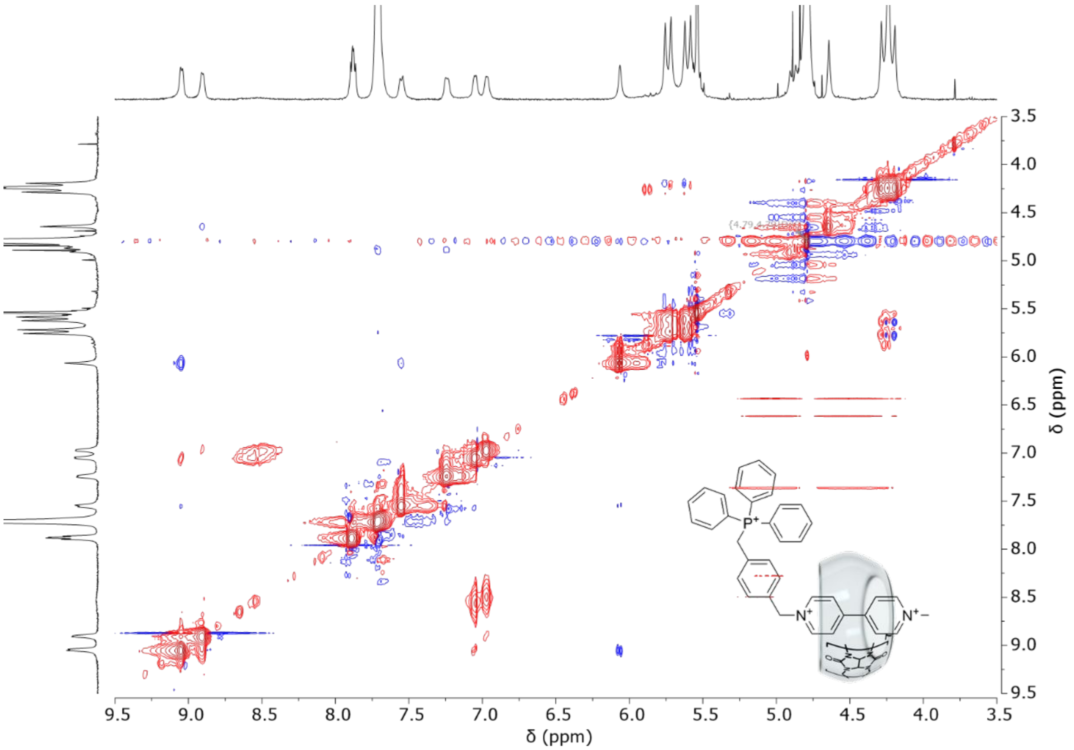

Figure S129: ^{1}H-^{1}H NOESY (400 MHz, D_2O) spectrum of **4^{3+}□CB[7]**. Red cross peaks indicate NOE correlations.

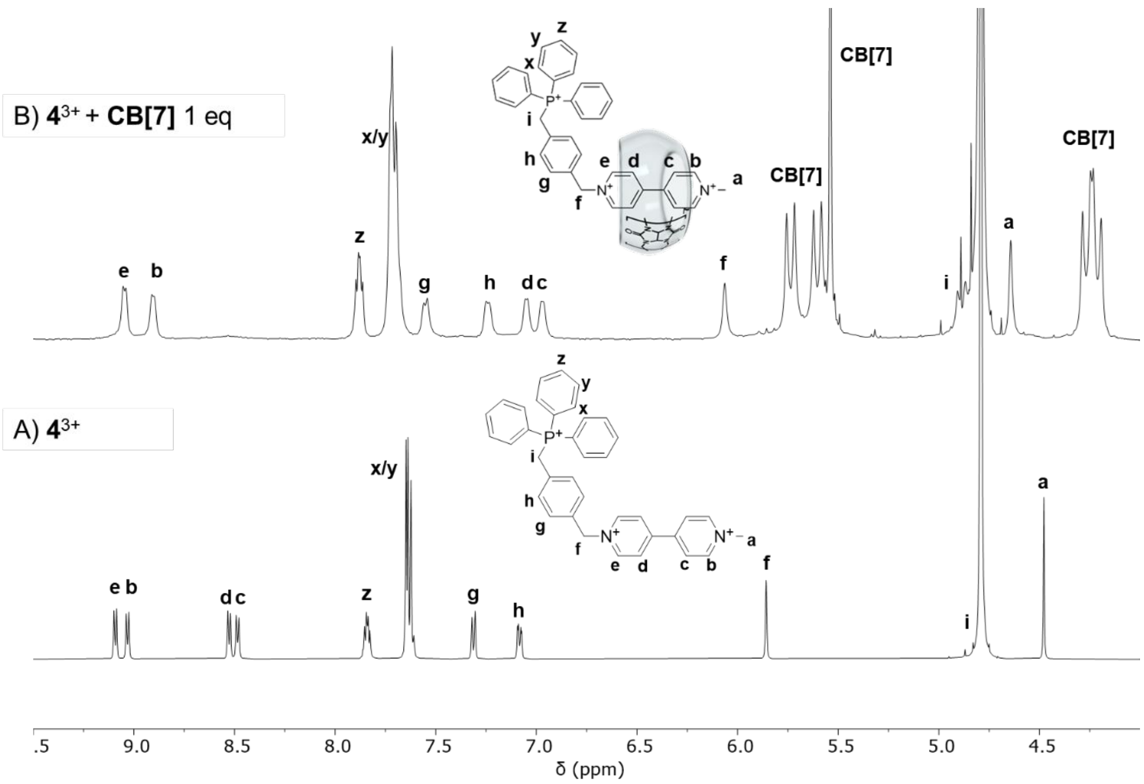

Figure S130: Partial ^{1}H NMR (400 MHz, D_2O) spectrum of: A) **4**$^{3+}$; B) **4**$^{3+}$ + 1 eq of **CB[7]**.

3.11. NMR study of the interaction between 5·2Br and CB[7]

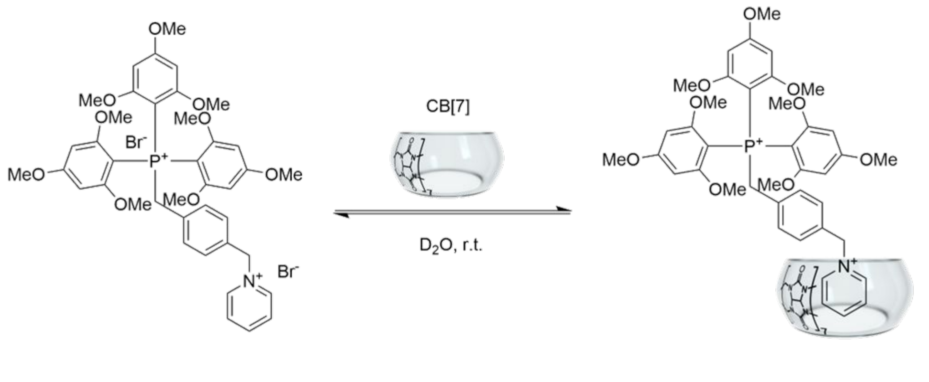

5·2Br $\qquad$ **5**$^{2+}$⊂**CB[7]**

The same procedure used for **2**·2Br is carried out.

^{1}H NMR (500 MHz, D_2O) *δ (ppm)*: 8.29 (d, *J* = 6.1 Hz, 2H), 8.08 (t, *J* = 7.9 Hz, 2H), 7.53 – 7.45 (m, 2H), 7.32 (q, *J* = 8.0 Hz, 4H), 6.28 (d, *J* = 4.7 Hz, 6H), 5.80 (d, *J* = 15.4 Hz, 14H), 5.58 (s, 14H), 5.51 (s, 2H), 4.29 (d, *J* = 15.4 Hz, 14H), 3.89 (s, 9H), 3.65 (s, 18H).

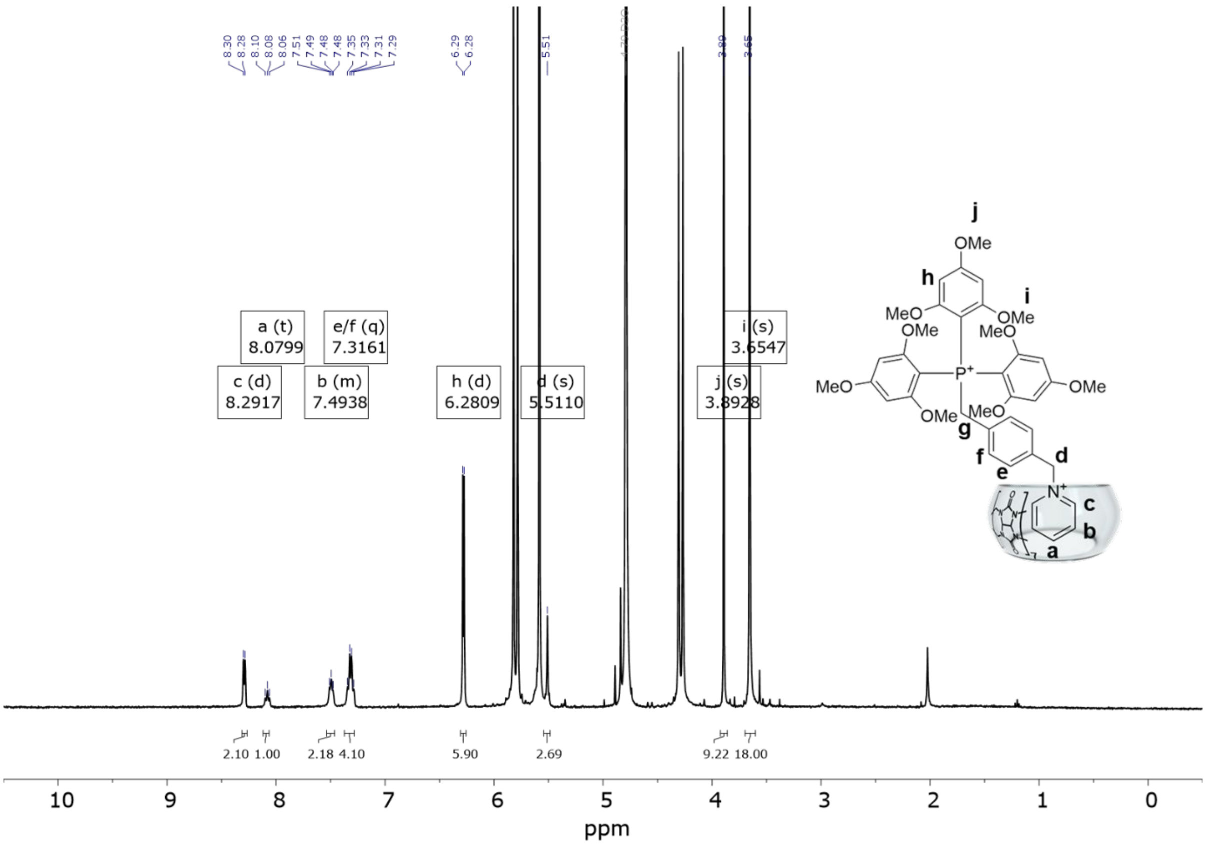

Figure S131: ^{1}H NMR (500 MHz, D_2O) spectrum of **5**$^{2+}$□**CB[7]**.

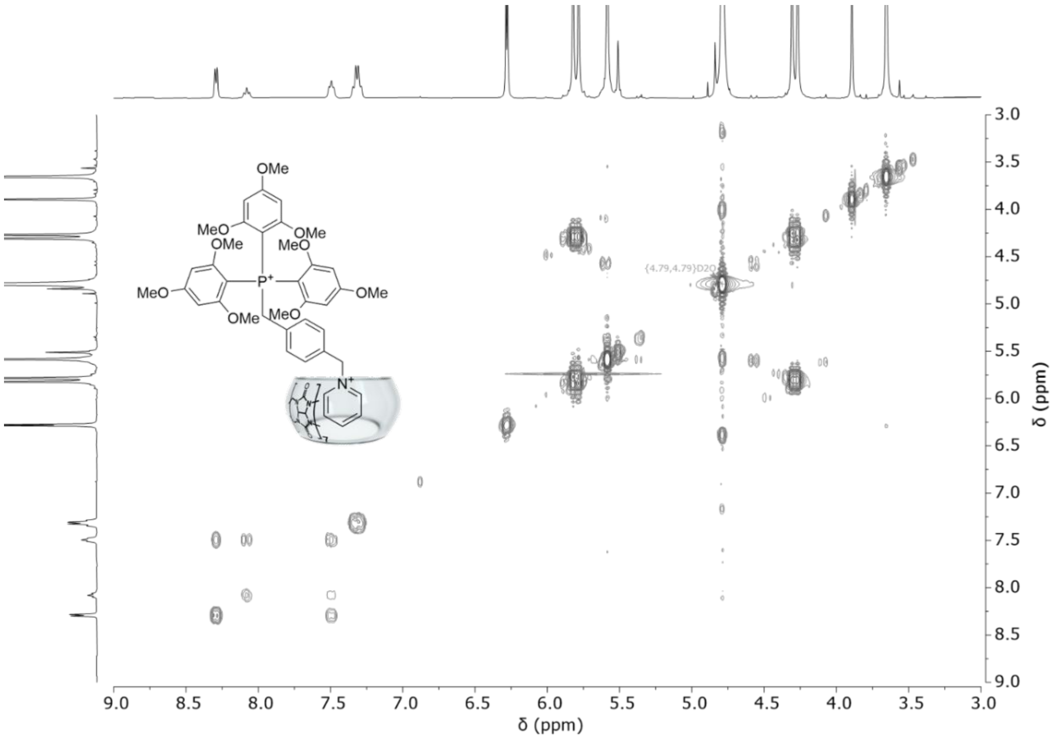

Figure S132: ^1H-^1H COSY (500 MHz, D_2O) spectrum of 5^{2+}□**CB[7]**.

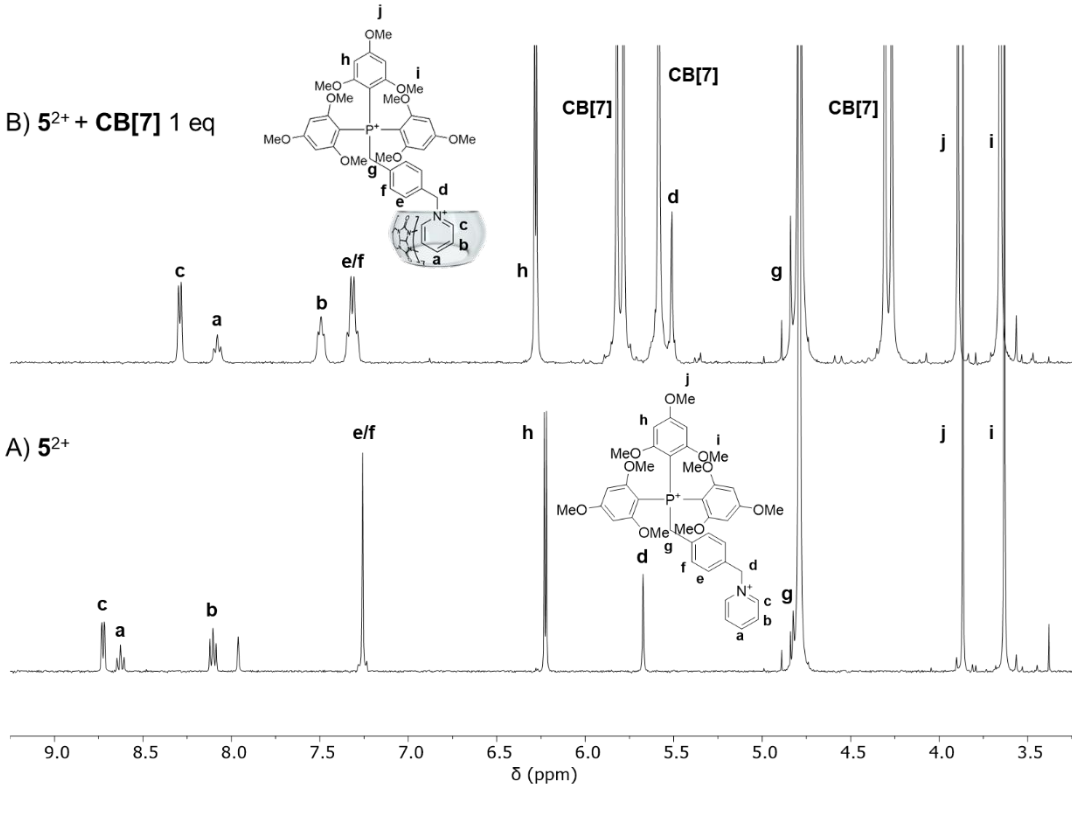

Figure S133: Partial ^1H NMR (400 MHz, D_2O) spectrum of: A) 5^{2+}; B) 5^{2+} + 1 eq of **CB[7]**.

3.12. NMR study of the interaction between 6·I and CB[7]

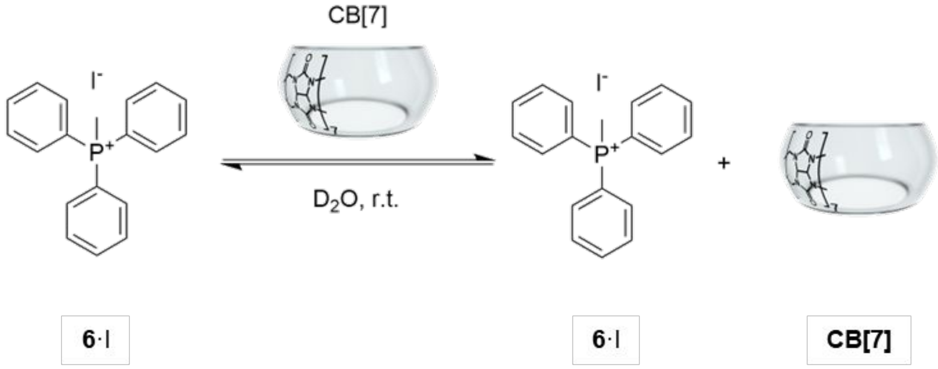

The same procedure used for **2**·2Br is carried out.

¹H NMR (300 MHz, D_2O) *δ (ppm)*: 7.83 (d, *J* = 7.4 Hz, 3H), 7.72 (d, *J* = 13.2 Hz, 12H), 5.79 (d, *J* = 15.4 Hz, 16H), 5.56 (s, 15H), 4.26 (d, *J* = 15.4 Hz, 15H), 2.88 (d, *J* = 14.0 Hz, 3H).

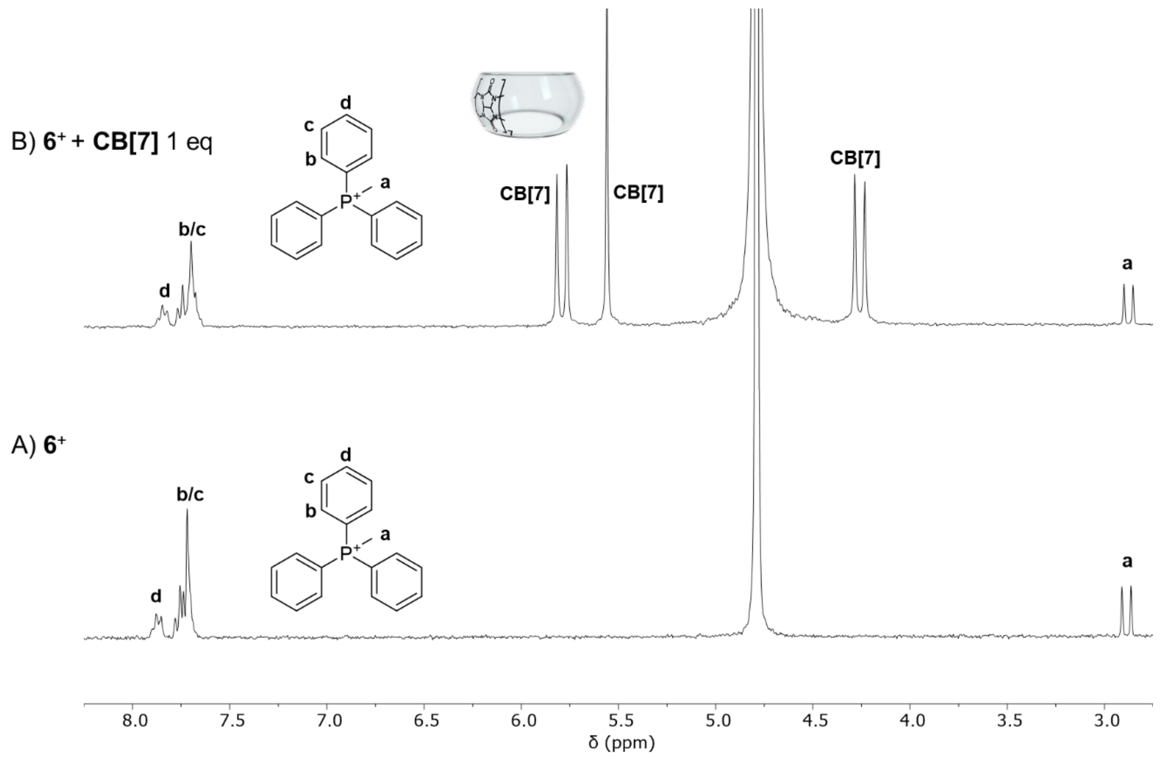

Figure S134: Partial ¹H NMR (300 MHz, D_2O) spectrum of: A) **6**⁺; B) **6**⁺ + 1 eq of **CB[7]**.

3.13. NMR study of the interaction between 7·Br and CB[7]

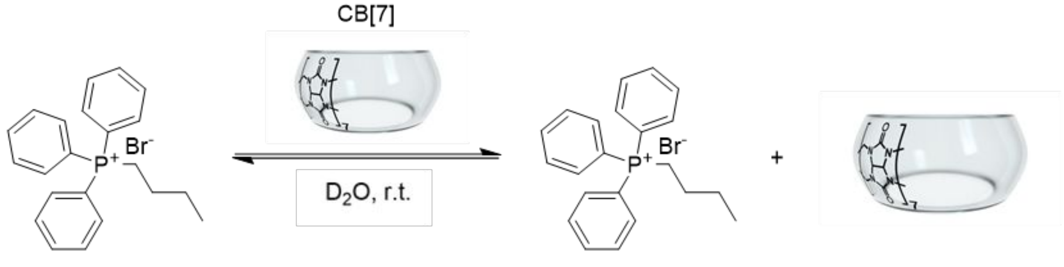

7·Br **7**·Br **CB[7]**

The same procedure used for **2**·2Br is carried out.

¹H NMR (300 MHz, D_2O) *δ (ppm)*: 7.87 – 7.54 (m, 12H), 5.76 (d, *J* = 15.5 Hz, 14H), 5.52 (s, 14H), 4.22 (d, *J* = 15.4 Hz, 14H), 3.31 – 3.08 (m, 2H), 1.73 – 1.49 (m, 2H), 1.46 – 1.30 (m, 2H), 0.87 – 0.69 (m, 3H).

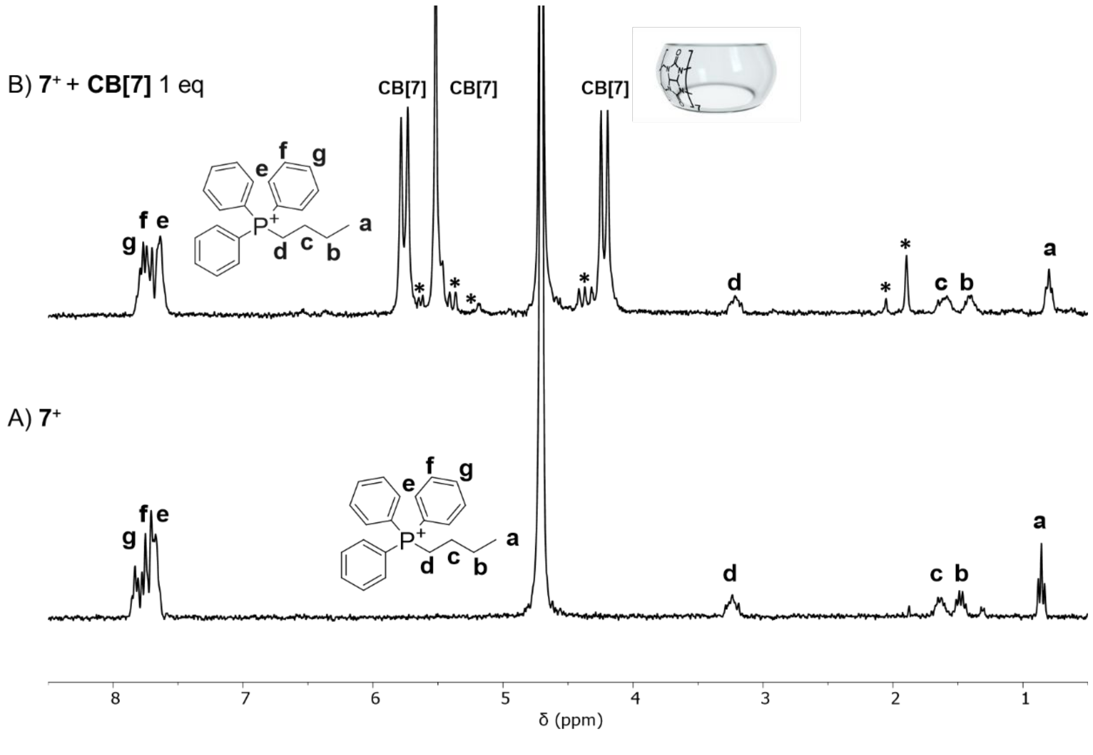

Figure S135: Partial ¹H NMR (300 MHz, D_2O) spectrum of: A) $\mathbf{7}^+$; B) $\mathbf{7}^+$ + 1 eq of **CB[7]**. Impurities are marked with *****.

3.14. NMR study of the interaction between 8·Br and CB[7]

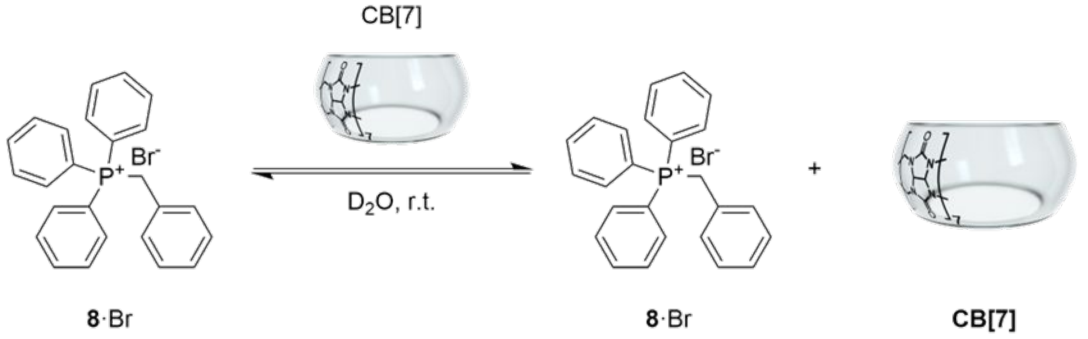

The same procedure used for **2**·2Br is carried out.

^{1}H NMR (300 MHz, D_2O) *δ (ppm)*: 7.79 (s, 3H), 7.61 (d, *J* = 8.0 Hz, 12H), 7.29 (s, 1H), 7.18 (s, 2H), 6.96 (s, 2H), 5.76 (d, *J* = 15.4 Hz, 12H), 5.50 (s, 14H), 4.30 (dd, *J* = 55.4, 14.6 Hz, 14H).

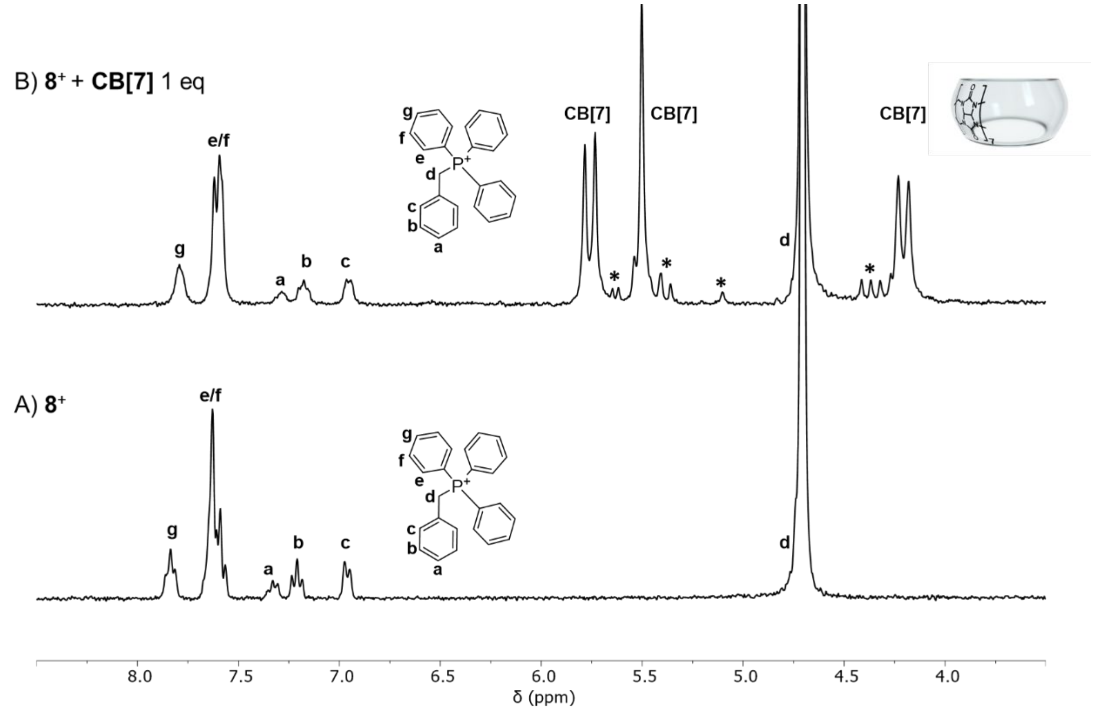

Figure S136: Partial ^{1}H NMR (300 MHz, D_2O) spectrum of: A) **8**$^+$; B) **8**$^+$ + 1 eq of **CB[7]**. Impurities are marked with ***.**

3.15. NMR study of the interaction between 6·I and CB[6]

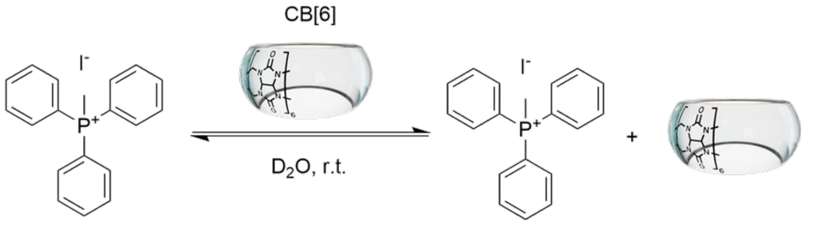

As with CB8, the solubility of CB6 in water is very low. Therefore, an excess of CB6 is added to a 2 mM solution of **6**·I. The resulting suspension is sonicated and heated at 50 ºC in an ultrasonic bath for 1 hour. After this time, the sample is filtered (nylon, 0.2 µm) and the spectrum of the solution is recorded.

^{1}H NMR (300 MHz, D_2O) *δ (ppm)*: 7.83 (d, *J* = 7.2 Hz, 3H), 7.76 – 7.65 (m, 12H), 5.78 (d, *J* = 15.3 Hz, 1H), 5.51 (s, 1H), 4.21 (d, *J* = 15.3 Hz, 1H), 2.87 (d, *J* = 13.9 Hz, 3H).

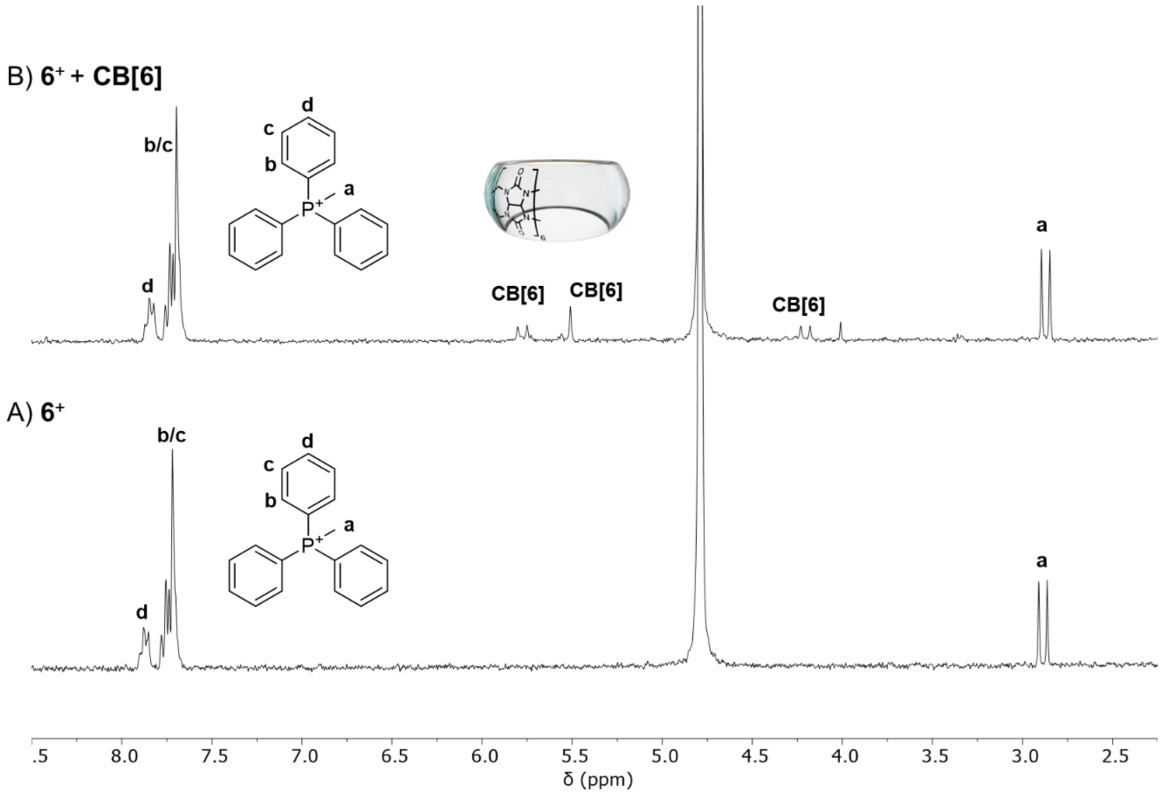

Figure S137: Partial ^{1}H NMR (300 MHz, D_2O) spectrum of: A) **6**$^+$; B) **6**$^+$ + **CB[6]**.

3.16. NMR study of the interaction between 7·Br and CB[6]

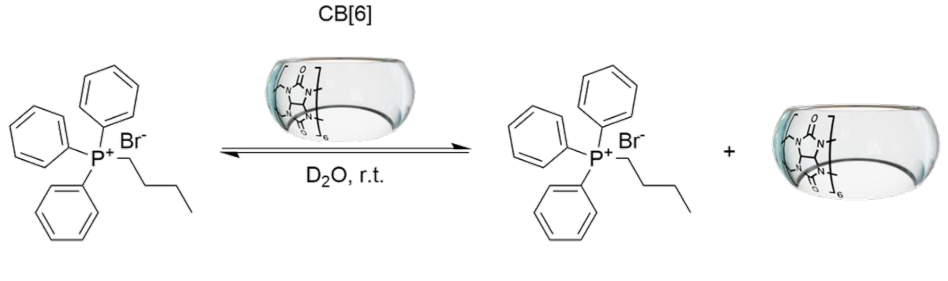

The same procedure used for **6**·I is carried out.

^{1}H NMR (300 MHz, D_2O) *δ (ppm)*: 7.92 – 7.67 (m, 15H), 5.76 (dd, *J* = 15.9, 7.6 Hz, 1H), 5.57 (s, 1H), 4.30 (d, *J* = 15.3 Hz, 1H), 3.40 – 3.13 (m, 2H), 1.67 (q, *J* = 8.1, 7.7 Hz, 2H), 1.50 (q, *J* = 7.4 Hz, 2H), 0.88 (t, *J* = 7.3 Hz, 3H).

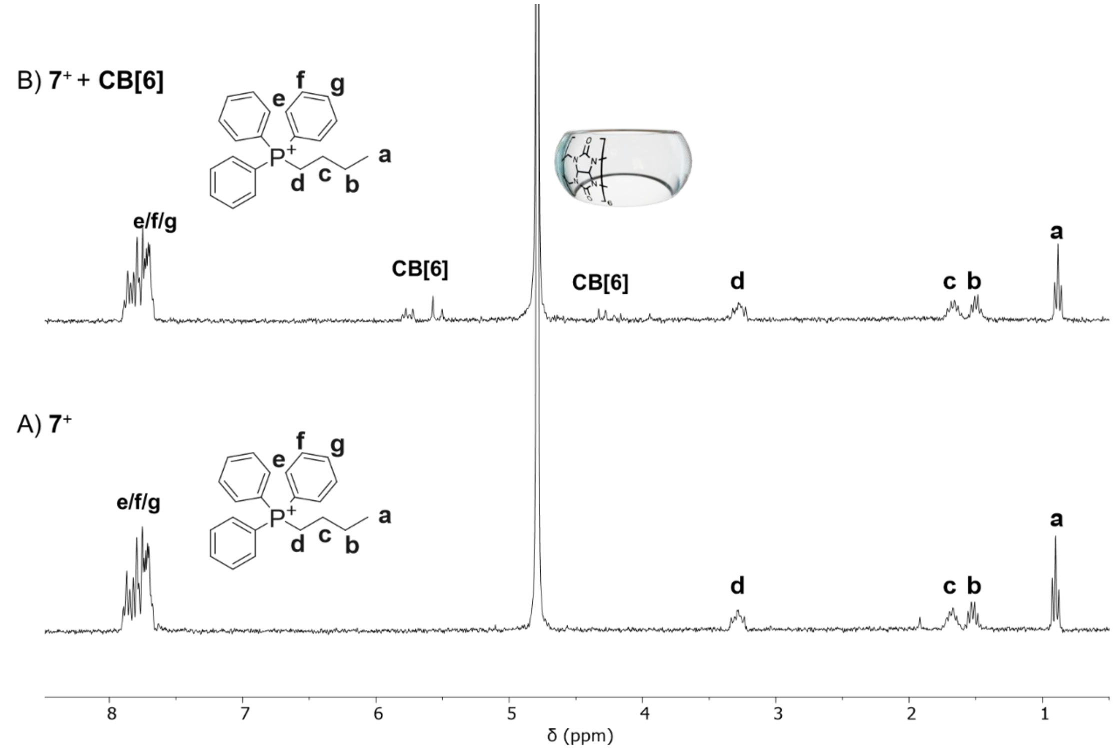

Figure S138: Partial ^{1}H NMR (300 MHz, D_2O) spectrum of: A) **7**$^+$; B) **7**$^+$ + **CB[6]**.

3.17. NMR study of the interaction between 8·Br and CB[6]

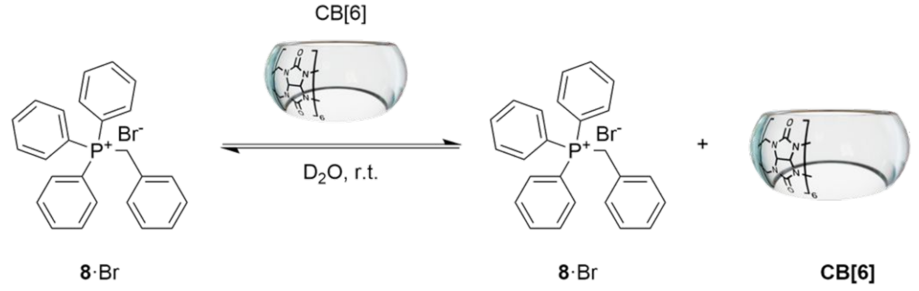

The same procedure used for **6**·I is carried out.

^{1}H NMR (300 MHz, D_2O) *δ (ppm)*: 7.87 (s, 3H), 7.67 (s, 12H), 7.36 (s, 1H), 7.24 (s, 2H), 7.00 (s, 2H), 5.76 (d, *J* = 13.2 Hz, 1H), 5.45 (s, 1H), 4.15 (d, *J* = 15.3 Hz, 1H).

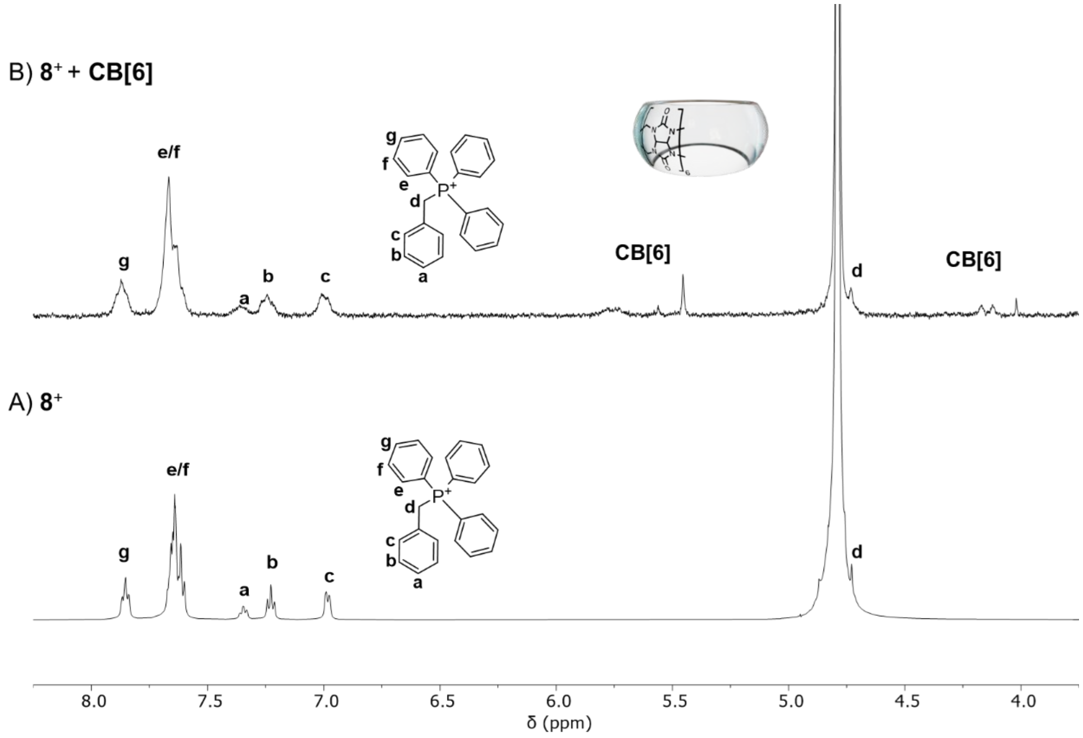

Figure S139: Partial ^{1}H NMR (300 MHz, D_2O) spectrum of: A) **8**$^+$; B) **8**$^+$ + **CB[6]**.

4. ISOTHERMAL TITRATION CALORIMETRY EXPERIMENTS

ITC experiments were performed using a Model Nano-ITC instrument (TA Instruments, USA) at 298 K. The estimated binding parameters were determined from ITC data using NanoAnalyze software provided by the manufacturer and the data fitting was done according to an independent binding model. All guest solutions were prepared using phosphate buffer 20 mM (pH=7.00). The concentration of this solutions was determined through UV-Vis spectroscopy. CB[8] solutions were prepared in phosphate buffer 20 mM (pH=7.00) and the concentration was assessed as previously reported by Kaifer *et al.*[1] The experiments were conducted by triplicate and the mean values of K_a, ΔG and n are shown in **Table S1**.

Table S1: Mean values of K_a, ΔG and n obtained from the ITC experiments of the different complexes.

Guest	Host	K_a (M^{-1})	ΔG ($kcal·mol^{-1}$)	n
**2^{2+}**	**CB[8]**	1.24E+07	-9.58	1.09
**3^{2+}**		2.40E+07	-10.07	1.25
**4^{3+}**		6.50E+06	-9.29	1.04
**5^{+}**		nb	nb	nb
**6^{+}**		7.34E+04	-6.62	1.17
**7^{+}**		2.15E+05	-7.27	1.48
**8^{+}**		nb	nb	nb
**2^{2+}**	**CB[7]**	3.32E+04	-6.32	0.95
**4^{3+}**		2.67E+06	-37.45	1.5

4.1. ITC data of complex 2^{2+}□CB[8]

Assessed concentration of host (CB[8]) in the cell was 170.5 µM. Assessed concentration of guest $\mathbf{2}^{2+}$ in the cell was 3.01 mM (ε_{260nm} = 5297 $M^{-1}\cdot cm^{-1}$).

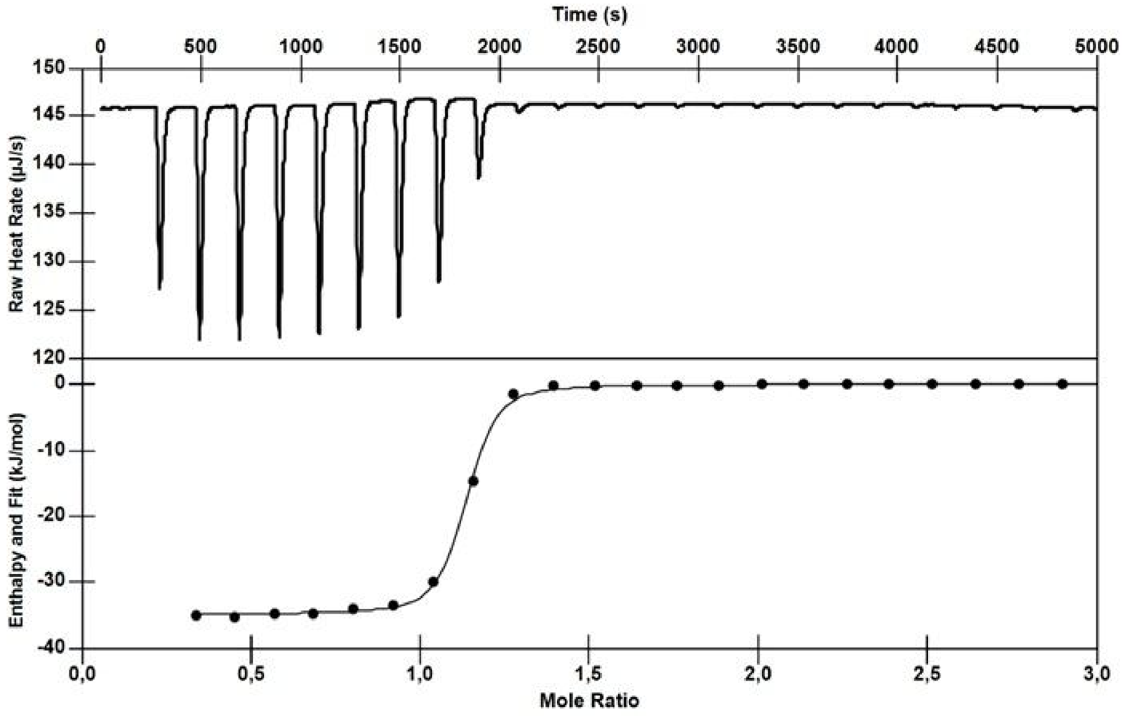

Figure S140: Titration and fitting graphics of **2^{2+}□CB[8].**

Table S2: Titration data of **2^{2+}□CB[8].**

Injection	Y: Area Data (μJ)	Injection Volume (μL)	Moles (Syringe)	Moles (Cell)	X: Mole Ratio	Cell Volume (μL)	Independent Model	Residual
1	8.0892	6	1.8072E-08	1.6095E-07	0.1123	950	-629.4619	-
2	-490.8560	6	3.6030E-08	1.5994E-07	0.2253	950	-629.2241	9.2610
3	-634.7074	6	5.3874E-08	1.5893E-07	0.3390	950	-628.8759	-5.8315
4	-637.5975	6	7.1606E-08	1.5792E-07	0.4534	950	-628.3364	-9.2610
5	-630.8717	6	8.9226E-08	1.5692E-07	0.5686	950	-627.4340	-3.4377
6	-627.8076	6	1.0673E-07	1.5593E-07	0.6845	950	-625.7501	-2.0574
7	-616.6185	6	1.2413E-07	1.5495E-07	0.8011	950	-622.0324	5.4139
8	-604.1089	6	1.4142E-07	1.5397E-07	0.9185	950	-611.0420	6.9330
9	-541.9978	6	1.5860E-07	1.5300E-07	1.0366	950	-553.7605	11.7627
10	-265.5903	6	1.7567E-07	1.5203E-07	1.1555	950	-256.9617	-8.6286
11	-26.8998	6	1.9263E-07	1.5107E-07	1.2751	950	-45.7561	18.8563
12	-5.0941	6	2.0949E-07	1.5012E-07	1.3955	950	-14.0209	8.9268
13	-1.4458	6	2.2624E-07	1.4917E-07	1.5166	950	-6.5663	5.1205
14	-2.6329	6	2.4288E-07	1.4823E-07	1.6386	950	-3.7735	1.1406
15	-1.5499	6	2.5942E-07	1.4729E-07	1.7613	950	-2.4390	0.8891
16	-1.7428	6	2.7585E-07	1.4636E-07	1.8847	950	-1.7011	-0.0417
17	-1.0382	6	2.9218E-07	1.4544E-07	2.0090	950	-1.2513	0.2131
18	0.6710	6	3.0841E-07	1.4452E-07	2.1341	950	-0.9573	1.6283
19	0.3991	6	3.2453E-07	1.4360E-07	2.2599	950	-0.7548	1.1539
20	-0.0033	6	3.4055E-07	1.4270E-07	2.3865	950	-0.6096	0.6064
21	0.1596	6	3.5647E-07	1.4180E-07	2.5140	950	-0.5020	0.6617
22	2.2161	6	3.7230E-07	1.4090E-07	2.6423	950	-0.4201	2.6362
23	2.8420	6	3.8802E-07	1.4001E-07	2.7713	950	-0.3564	3.1984
24	1.2317	6	4.0364E-07	1.3913E-07	2.9012	950	-0.3059	1.5375
25	-0.5374	6	4.1916E-07	1.3825E-07	3.0320	950	-0.2651	-0.2723

Table S3: Thermodynamic data of the replicate 1 of **2**$^{2+}$□**CB[8]**.

Models	Variable	Value	Error
Independent	Kd (M)	2.23E-07	5.02E-08
	n	1.079	0.004
	ΔH (kJ/mol)	-34.88	0.324
	Ka (M^{-1})	4.48E+06	
	-TΔS (kJ/mol)	-3.085	
	ΔG (kJ/mol)	-37.96	
	ΔS (J/mol·K)	10.35	
	Confidence Level (%)	95	

Table S4: Thermodynamic data of the replicate 2 of **2**$^{2+}$□**CB[8]**.

Models	Variable	Value	Error
Independent	Kd (M)	8.99E-08	4.36E-08
	n	0.997	0.005
	ΔH (kJ/mol)	-35.99	0.541
	Ka (M^{-1})	1.11E+07	
	-TΔS (kJ/mol)	-76.21	
	ΔG (kJ/mol)	-40.22	
	ΔS (J/mol·K)	255.6	
	Confidence Level (%)	95	

Table S5: Thermodynamic data of the replicate 3 of **2^{2+}□CB[8]**.

Models	Variable	Value	Error
Independent	Kd (M)	5.07E-08	1.54E-08
	n	1.187	0.003
	ΔH (kJ/mol)	-35.24	0.285
	Ka (M^{-1})	1.97E+07	
	-TΔS (kJ/mol)	-6.403	
	ΔG (kJ/mol)	-41.64	
	ΔS (J/mol·K)	21.48	
	Confidence Level (%)	95	

4.2. ITC data of complex 3^{2+}□CB[8]

Assessed concentration of host (**CB[8]**) in the cell was 121.7 µM. Assessed concentration of guest **3**$^{2+}$ in the cell was 2.95 mM (ε_{267nm} = 7216 $M^{-1}cm^{-1}$).

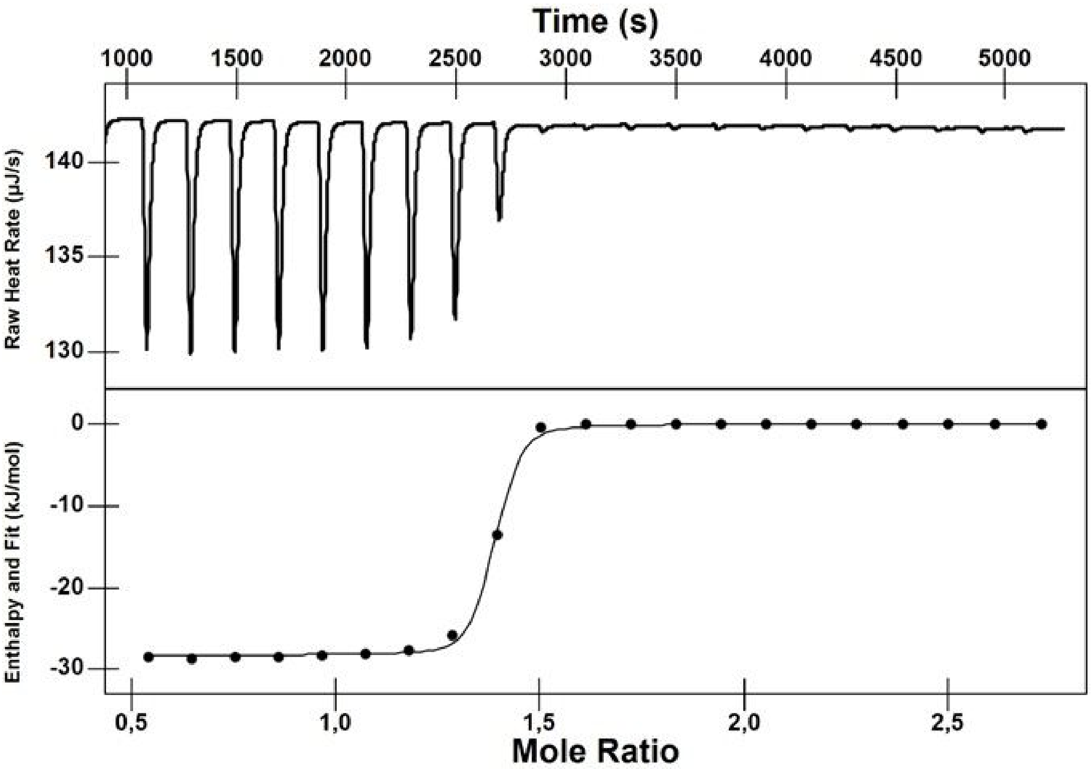

Figure S141: Titration and fitting graphics of **3**$^{2+}$□**CB[8].**

Table S6: Titration data of one of the replicates of **3^{2+}□CB[8]**.

Injection	Y: Area Data (μJ)	Injection Volume (μL)	Moles (Syringe)	Moles (Cell)	X: Mole Ratio	Cell Volume (μL)	Independent Model	Residual
1	9.31927	0.86	2.55E-09	1.16E-07	0.02204	950	-71.8296	-
2	9.50994	4	1.44E-08	1.15E-07	0.12497	950	-334.0807	-
3	9.26464	4	2.62E-08	1.15E-07	0.22834	950	-334.0597	-
4	9.09566	4	3.79E-08	1.14E-07	0.33215	950	-334.0320	-
5	-36.25959	4	4.96E-08	1.14E-07	0.43640	950	-333.9947	-
6	-336.45756	4	6.12E-08	1.13E-07	0.54108	950	-333.9424	-2.5151
7	-338.21265	4	7.28E-08	1.13E-07	0.64621	950	-333.8662	-4.3465
8	-336.47232	4	8.43E-08	1.12E-07	0.75179	950	-333.7485	-2.7238
9	-336.71122	4	9.58E-08	1.12E-07	0.85781	950	-333.5526	-3.1586
10	-333.93306	4	1.07E-07	1.11E-07	0.96428	950	-333.1897	-0.7434
11	-331.20037	4	1.19E-07	1.11E-07	1.07120	950	-332.3948	1.1944
12	-326.37834	4	1.30E-07	1.10E-07	1.17857	950	-330.0506	3.6722
13	-306.24916	4	1.41E-07	1.10E-07	1.28639	950	-316.2153	9.9661
14	-159.92073	4	1.53E-07	1.09E-07	1.39467	950	-156.9507	-2.9700
15	-4.13091	4	1.64E-07	1.09E-07	1.50341	950	-15.1754	11.0445
16	1.49478	4	1.75E-07	1.08E-07	1.61261	950	-3.7598	5.2546
17	0.45593	4	1.86E-07	1.08E-07	1.72227	950	-1.6638	2.1197
18	0.84359	4	1.97E-07	1.08E-07	1.83240	950	-0.9333	1.7769
19	0.56062	4	2.08E-07	1.07E-07	1.94299	950	-0.5957	1.1563
20	0.92439	4	2.19E-07	1.07E-07	2.05404	950	-0.4125	1.3369
21	-0.19064	4	2.30E-07	1.06E-07	2.16557	950	-0.3021	0.1114
22	-0.33488	4	2.41E-07	1.06E-07	2.27757	950	-0.2305	-0.1044
23	-0.14230	4	2.52E-07	1.05E-07	2.39004	950	-0.1815	0.0392
24	0.12559	4	2.62E-07	1.05E-07	2.50299	950	-0.1465	0.2721
25	-0.42378	4	2.73E-07	1.04E-07	2.61642	950	-0.1206	-0.3032
26	-0.51687	4	2.84E-07	1.04E-07	2.73032	950	-0.1010	-0.4159

Table S7 Thermodynamic data of the replicate 1 of **3**$^{2+}$□**CB[8]**.

**Models**	**Variable**	**Value**	**Error**
Independent	Kd (M)	4.66E-08	2.01E-08
	n	1.337	0.004
	ΔH (kJ/mol)	-28.23	0.273
	Ka (M^{-1})	2.15E+07	
	-TΔS (kJ/mol)	-13.62	
	ΔG (kJ/mol)	-41.85	
	ΔS (J/mol·K)	45.7	
	Confidence Level (%)	95	

Table S8: Thermodynamic data of the replicate 2 of **3**$^{2+}$□**CB[8]**.

**Models**	**Variable**	**Value**	**Error**
Independent	Kd (M)	3.61E-08	5.72E-08
	n	1.203	0.014
	ΔH (kJ/mol)	-28.04	1.107
	Ka (M^{-1})	2.77E+07	
	-TΔS (kJ/mol)	-14.44	
	ΔG (kJ/mol)	-42.48	
	ΔS (J/mol·K)	48.44	
	Confidence Level (%)	95	

Table S9: Thermodynamic data the replicate 3 of of **3^{2+}□CB[8]**.

**Models**	**Variable**	**Value**	**Error**
Independent	Kd (M)	4.36E-08	3.47E-08
	n	1.216	0.006
	ΔH (kJ/mol)	-28.29	0.490
	Ka (M^{-1})	2.29E+07	
	-TΔS (kJ/mol)	-13.72	
	ΔG (kJ/mol)	-42.01	
	ΔS (J/mol·K)	46.03	
	Confidence Level (%)	95	

4.3. ITC data of complex 4^{3+}□CB[8]

Assessed concentration of host (**CB[8]**) in the cell was 170.5 µM. Assessed concentration of guest **4**$^{3+}$ in the cell was 3.59 mM (ε_{262nm} = 18132 $M^{-1}cm^{-1}$).

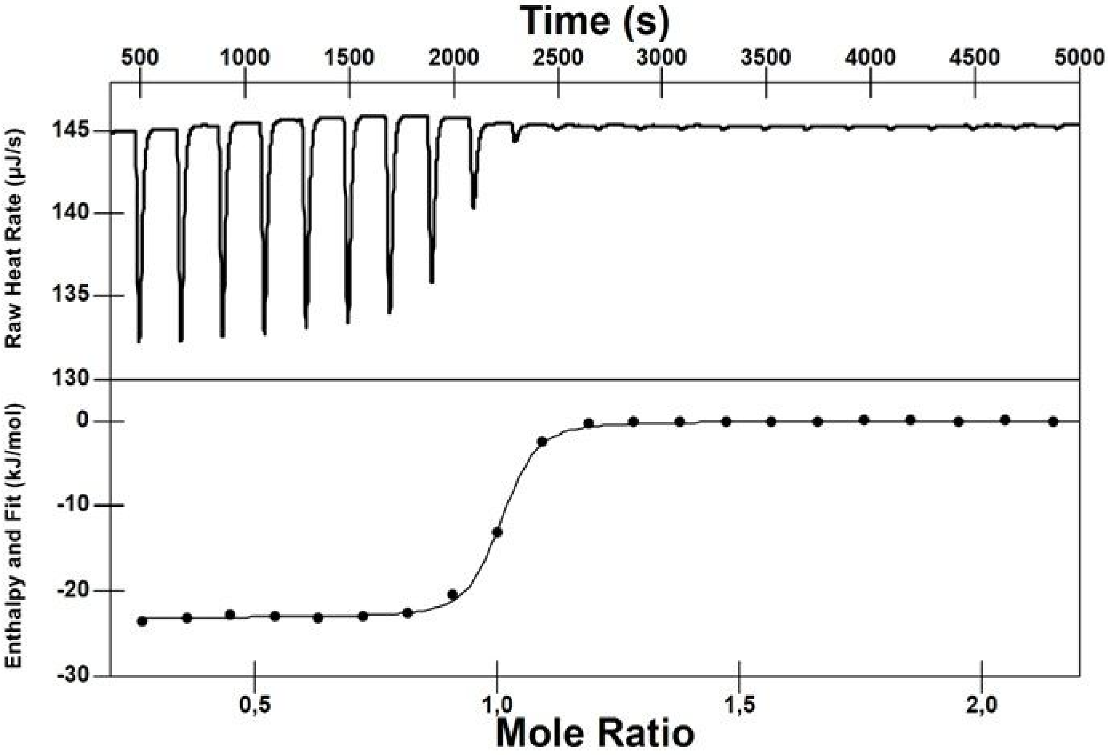

Figure S142: Titration and fitting graphics of **4**$^{3+}$□**CB[8].**

Table S10: Titration data of one of the replicates of **4**$^{3+}$□**CB[8]**.

Injection	Y: Area Data (μJ)	Injection Volume (μL)	Moles (Syringe)	Moles (Cell)	X: Mole Ratio	Cell Volume (μL)	Independent Model	Residual
1	6.14984	4	1.44E-08	1.61E-07	0.089031	950	-332.23809	-
2	-162.78361	4	2.87E-08	1.61E-07	0.178437	950	-332.14857	-
3	-339.05659	4	4.29E-08	1.60E-07	0.268223	950	-332.02418	-7.03241
4	-331.45840	4	5.71E-08	1.59E-07	0.358387	950	-331.84405	0.38565
5	-328.26207	4	7.12E-08	1.59E-07	0.448933	950	-331.56899	3.30692
6	-330.48944	4	8.53E-08	1.58E-07	0.539862	950	-331.11820	0.62876
7	-333.60951	4	9.93E-08	1.57E-07	0.631175	950	-330.30235	-3.30715
8	-329.56163	4	1.13E-07	1.57E-07	0.722874	950	-328.58841	-0.97321
9	-325.06062	4	1.27E-07	1.56E-07	0.814961	950	-323.97090	-1.08972
10	-293.72832	4	1.41E-07	1.55E-07	0.907438	950	-304.05469	10.32637
11	-187.74592	4	1.55E-07	1.55E-07	1.000305	950	-183.99536	-3.75057
12	-35.10387	4	1.68E-07	1.54E-07	1.093566	950	-38.04713	2.94326
13	-2.11417	4	1.82E-07	1.53E-07	1.187220	950	-10.16127	8.04710
14	0.49124	4	1.96E-07	1.53E-07	1.281270	950	-4.44770	4.93893
15	-1.99578	4	2.09E-07	1.52E-07	1.375719	950	-2.46751	0.47173
16	0.10074	4	2.23E-07	1.51E-07	1.470566	950	-1.56197	1.66271
17	-0.55396	4	2.36E-07	1.51E-07	1.565815	950	-1.07489	0.52093
18	-1.38428	4	2.49E-07	1.50E-07	1.661466	950	-0.78353	-0.60075
19	1.25851	4	2.63E-07	1.49E-07	1.757522	950	-0.59568	1.85419
20	1.18184	4	2.76E-07	1.49E-07	1.853984	950	-0.46763	1.64946
21	-0.26015	4	2.89E-07	1.48E-07	1.950853	950	-0.37649	0.11634
22	1.13751	4	3.02E-07	1.48E-07	2.048133	950	-0.30936	1.44688
23	-0.70718	4	3.15E-07	1.47E-07	2.145824	950	-0.25852	-0.44866
24	0.08678	4	3.28E-07	1.46E-07	2.243927	950	-0.21911	0.30589
25	1.14085	4	3.41E-07	1.46E-07	2.342446	950	-0.18795	1.32879

Table S11: Thermodynamic data of the replicate 1 of **4**$^{3+}$□**CB[8]**.

Models	Variable	Value	Error
Independent	Kd (M)	1.30E-07	4.34E-08
	n	1.036	0.004
	ΔH (kJ/mol)	-23.42	0.211
	Ka (M^{-1})	7.70E+06	
	-TΔS (kJ/mol)	-15.88	
	ΔG (kJ/mol)	-39.31	
	ΔS (J/mol·K)	53.28	
	Confidence Level (%)	95	

Table S12: Thermodynamic data of the replicate 2 of **4**$^{3+}$□**CB[8]**.

Models	Variable	Value	Error
Independent	Kd (M)	1.70E-07	3.93E-08
	n	0.963	0.003
	ΔH (kJ/mol)	-23.16	0.21
	Ka (M^{-1})	5.90E+06	
	-TΔS (kJ/mol)	-15.49	
	ΔG (kJ/mol)	-38.65	
	ΔS (J/mol·K)	51.94	
	Confidence Level (%)	95	

Table S13: Thermodynamic data of the replicate 3 of **4^{3+}□CB[8]**.

**Models**	**Variable**	**Value**	**Error**
Independent	Kd (M)	1.70E-07	4.79E-08
	n	1.126	0.005
	ΔH (kJ/mol)	-23.8	0.245
	Ka (M^{-1})	5.89E+06	
	-TΔS (kJ/mol)	-14.85	
	ΔG (kJ/mol)	-38.64	
	ΔS (J/mol·K)	49.79	
	Confidence Level (%)	95	

4.4. ITC data of complex 6^+□CB[8]

Assessed concentration of host (**CB[8]**) in the cell was 127.8 µM. Assessed concentration of guest **6**$^+$ in the cell was 5.33 mM (ε_{268nm} = 3201 $M^{-1}cm^{-1}$).

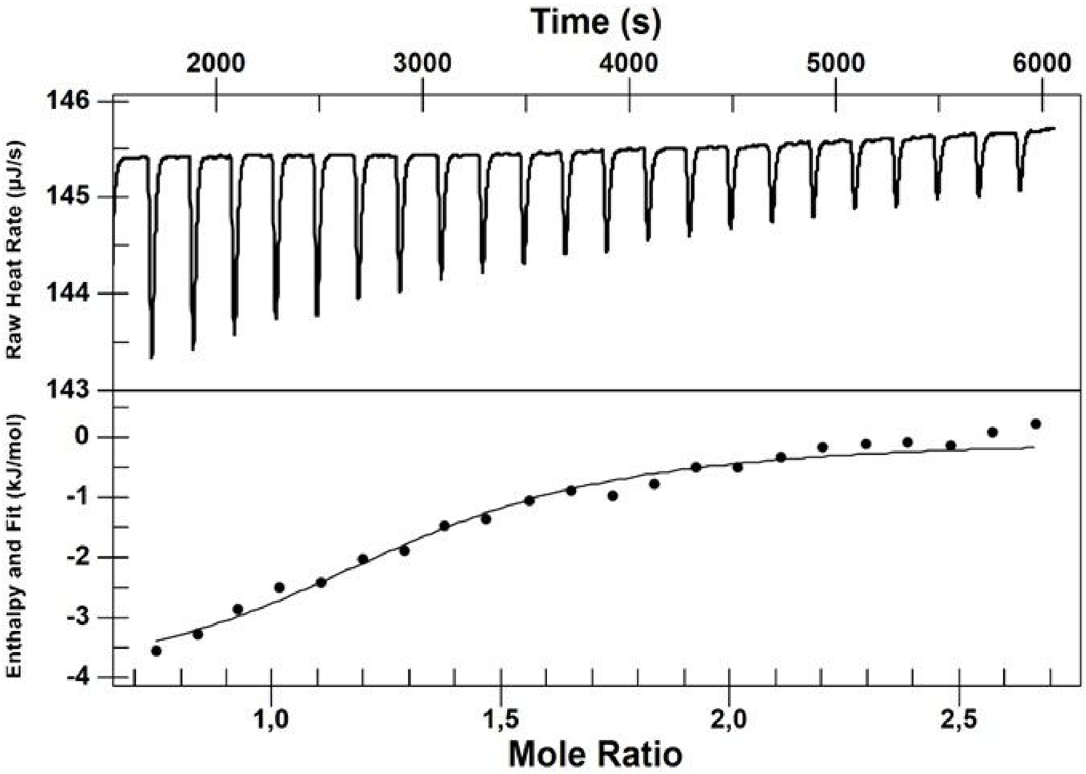

Figure S143: Titration and fitting graphics of **6**$^+$**□CB[8].**

Table S14: Titration data of one of the replicates of **6**$^+$□**CB[8]**.

Injection	Y: Area Data (µJ)	Injection Volume (µL)	Moles (Syringe)	Moles (Cell)	X: Mole Ratio	Cell Volume (µL)	Independent Model	Residual
1	14.161261	0.86	4.58E-09	1.21E-07	0.037789	950	-21.49708	-
2	18.5976116	2	1.52E-08	1.21E-07	0.125856	950	-49.476841	-
3	15.215738	2	2.59E-08	1.21E-07	0.214108	950	-48.643125	-
4	17.7737946	2	3.65E-08	1.21E-07	0.302547	950	-47.646048	-
5	16.5245571	2	4.71E-08	1.20E-07	0.391172	950	-46.451024	-
6	15.1472216	2	5.76E-08	1.20E-07	0.479984	950	-45.018942	-
7	-7.3263851	2	6.82E-08	1.20E-07	0.568983	950	-43.308591	-
8	-37.367421	2	7.87E-08	1.20E-07	0.658171	950	-41.281874	-
9	-37.982746	2	8.92E-08	1.19E-07	0.747546	950	-38.912698	-
10	-37.892427	2	9.96E-08	1.19E-07	0.837110	950	-36.199381	-1.6930457
11	-34.223255	2	1.10E-07	1.19E-07	0.926863	950	-33.177845	-1.0454109
12	-26.768131	2	1.21E-07	1.19E-07	1.016805	950	-29.92958	3.1614493
13	-25.899024	2	1.31E-07	1.18E-07	1.106937	950	-26.577161	0.67813719
14	-21.392109	2	1.41E-07	1.18E-07	1.197260	950	-23.264379	1.87227047
15	-20.927515	2	1.52E-07	1.18E-07	1.287772	950	-20.127159	-0.8003558
16	-17.649845	2	1.62E-07	1.18E-07	1.378476	950	-17.267914	-0.381931
17	-16.922508	2	1.72E-07	1.17E-07	1.469371	950	-14.743538	-2.1789697
18	-12.918549	2	1.83E-07	1.17E-07	1.560458	950	-12.56824	-0.3503091
19	-11.204696	2	1.93E-07	1.17E-07	1.651737	950	-10.725249	-0.479447
20	-10.040953	2	2.03E-07	1.17E-07	1.743209	950	-9.1802381	-0.860715
21	-8.7536512	2	2.13E-07	1.16E-07	1.834873	950	-7.8921089	-0.8615424
22	-7.5822147	2	2.24E-07	1.16E-07	1.926731	950	-6.8199075	-0.7623072
23	-7.4627458	2	2.34E-07	1.16E-07	2.018783	950	-5.9264433	-1.5363024
24	-5.5633949	2	2.44E-07	1.16E-07	2.111029	950	-5.1796881	-0.3837068
25	-4.4093002	2	2.54E-07	1.15E-07	2.203469	950	-4.5529153	0.14361508
26	-2.509514	2	2.64E-07	1.15E-07	2.296105	950	-4.0242329	1.51471886
27	-2.93123	2	2.74E-07	1.15E-07	2.388936	950	-3.575893	0.64466302
28	0.40135829	2	2.84E-07	1.15E-07	2.481963	950	-3.1935784	3.59493667
29	-0.7700746	2	2.94E-07	1.14E-07	2.575186	950	-2.865755	2.09568039
30	0.85228666	2	3.05E-07	1.14E-07	2.668606	950	-2.5831214	3.4354081

Table S15: Thermodynamic data of the replicate 1 of **6**$^{+}$□**CB[8]**.

**Models**	**Variable**	**Value**	**Error**
Independent	Kd (M)	1.48E-05	7.26E-06
	n	1.158	0.197
	ΔH (kJ/mol)	-5.173	2.001
	Ka (M^{-1})	6.76E+04	
	-TΔS (kJ/mol)	-22.4	
	ΔG (kJ/mol)	-27.57	
	ΔS (J/mol·K)	75.11	
	Confidence Level (%)	95	

Table S16: Thermodynamic data of the replicate 2 of **6**$^{+}$□**CB[8]**.

**Models**	**Variable**	**Value**	**Error**
Independent	Kd (M)	1.01E-05	4.49E-06
	n	1.215	0.107
	ΔH (kJ/mol)	-4.331	0.763
	Ka (M^{-1})	9.87E+04	
	-TΔS (kJ/mol)	-24.18	
	ΔG (kJ/mol)	-28.51	
	ΔS (J/mol·K)	81.09	
	Confidence Level (%)	95	

Table S 17: Thermodynamic data of the replicate 3 of **6**$^{+}$□**CB[8]**.

Models	Variable	Value	Error
Independent	Kd (M)	1.85E-05	7.85E-06
	n	1.124	0.11
	ΔH (kJ/mol)	-4.997	0.85
	Ka (M^{-1})	5.41E+04	
	-TΔS (kJ/mol)	-22.02	
	ΔG (kJ/mol)	-27.01	
	ΔS (J/mol·K)	73.85	
	Confidence Level (%)	95	

4.5. ITC data of complex 7^+□CB[8]

Assessed concentration of host (**CB[8]**) in the cell was 128.2 µM. Assessed concentration of guest **7**$^+$ in the cell was 4.84 mM (ε_{268nm} = 2058 $M^{-1}cm^{-1}$).

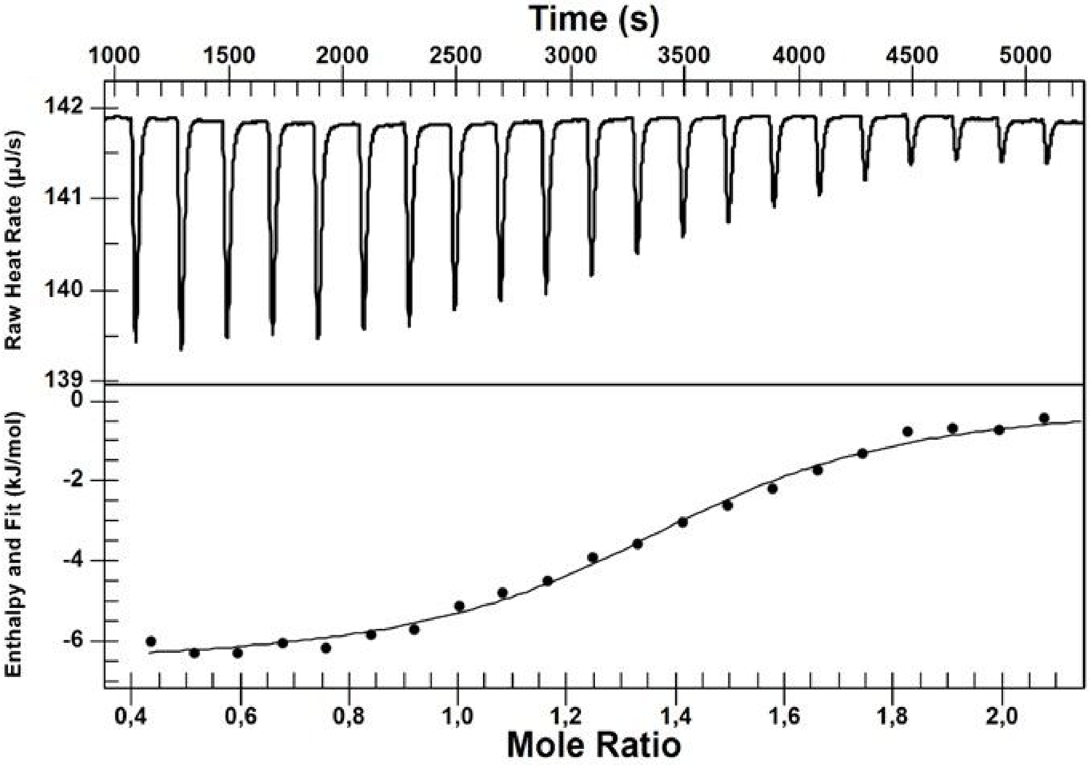

Figure S144: Titration and fitting graphics of **7**$^+$□**CB[8].**

Table S 18: Titration data of one of the replicates of **7**$^{+}$□**CB[8]**.

Injection	Y: Area Data (μJ)	Injection Volume (μL)	Moles (Syringe)	Moles (Cell)	X: Mole Ratio	Cell Volume (μL)	Independent Model	Residual
1	9.30287309	0.86	4.16E-09	1.22E-07	0.03420783	950	-26.892886	-
2	7.16345145	2	1.38E-08	1.21E-07	0.11392875	950	-62.362756	-
3	6.3458628	2	2.35E-08	1.21E-07	0.19381786	950	-62.075569	-
4	8.09667201	2	3.31E-08	1.21E-07	0.27387552	950	-61.73292	-
5	0.03438706	2	4.27E-08	1.21E-07	0.35410207	950	-61.320416	-
6	-57.985799	2	5.23E-08	1.20E-07	0.43449788	950	-60.819062	2.83326342
7	-60.792624	2	6.19E-08	1.20E-07	0.51506329	950	-60.203603	-0.5890209
8	-60.790102	2	7.14E-08	1.20E-07	0.59579868	950	-59.440268	-1.3498336
9	-58.486805	2	8.10E-08	1.20E-07	0.6767044	950	-58.483826	-0.0029787
10	-59.599264	2	9.05E-08	1.19E-07	0.7577808	950	-57.273998	-2.3252662
11	-56.415855	2	1.00E-07	1.19E-07	0.83902825	950	-55.731771	-0.6840839
12	-55.167945	2	1.09E-07	1.19E-07	0.92044711	950	-53.757353	-1.4105918
13	-49.556713	2	1.19E-07	1.19E-07	1.00203773	950	-51.233886	1.67717282
14	-46.265825	2	1.28E-07	1.18E-07	1.08380049	950	-48.044601	1.77877633
15	-43.529397	2	1.38E-07	1.18E-07	1.16573575	950	-44.113005	0.5836083
16	-37.861456	2	1.47E-07	1.18E-07	1.24784386	950	-39.467163	1.60570715
17	-34.705061	2	1.56E-07	1.18E-07	1.3301252	950	-34.300403	-0.4046579
18	-29.548293	2	1.66E-07	1.17E-07	1.41258012	950	-28.968516	-0.5797769
19	-25.170549	2	1.75E-07	1.17E-07	1.49520901	950	-23.886822	-1.283727

20	-21.368611	2	1.84E-07	1.17E-07	1.57801221	950	-19.383551	-1.9850599
21	-16.921788	2	1.94E-07	1.17E-07	1.66099011	950	-15.615525	-1.3062632
22	-12.546001	2	2.03E-07	1.16E-07	1.74414306	950	-12.583058	0.03705715
23	-7.5062964	2	2.12E-07	1.16E-07	1.82747144	950	-10.195821	2.68952437
24	-6.4584427	2	2.22E-07	1.16E-07	1.91097562	950	-8.3336187	1.87517592
25	-7.0315045	2	2.31E-07	1.16E-07	1.99465597	950	-6.8814799	-0.1500245
26	-4.3801379	2	2.40E-07	1.15E-07	2.07851286	950	-5.7433231	1.36318523

Table S19: Thermodynamic data of the replicate 1 of **7**$^{+}$□**CB[8]**.

Models	Variable	Value	Error
Independent	Kd (M)	5.58E-06	1.25E-06
	n	1.368	0.026
	ΔH (kJ/mol)	-6.672	0.231
	Ka (M^{-1})	1.79E+05	
	-TΔS (kJ/mol)	-23.32	
	ΔG (kJ/mol)	-29.99	
	ΔS (J/mol·K)	78.2	
	Confidence Level (%)	95	

Table S20: Thermodynamic data of the replicate 2 of **7**$^{+}$□**CB[8]**.

Models	Variable	Value	Error
Independent	Kd (M)	4.98E-06	1.48E-06
	n	1.456	0.036
	ΔH (kJ/mol)	-6.552	0.277
	Ka (M^{-1})	2.01E+05	
	-TΔS (kJ/mol)	-23.72	
	ΔG (kJ/mol)	-30.27	
	ΔS (J/mol·K)	79.54	
	Confidence Level (%)	95	

Table S21: Thermodynamic data of the replicate 3 of **7**$^{+}$□**CB[8]**.

Models	Variable	Value	Error
Independent	Kd (M)	3.79E-06	1.28E-06
	n	1.663	0.038
	ΔH (kJ/mol)	-6.147	0.281
	Ka (M^{-1})	2.64E+05	
	-TΔS (kJ/mol)	-24.8	
	ΔG (kJ/mol)	-30.94	
	ΔS (J/mol·K)	83.17	
	Confidence Level (%)	95	

4.6. ITC data of negative controls

4.6.1. 5^{2+} and CB[8]

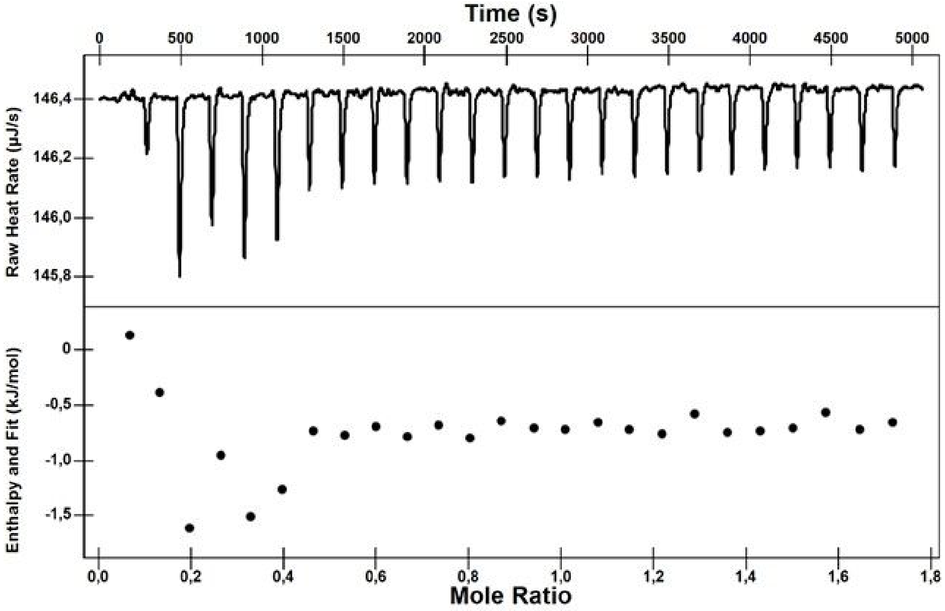

Figure S145: Raw data of the titration of $\mathbf{5^{2+}}$ and **CB[8]**.

4.6.2. 8^{+} and CB[8]

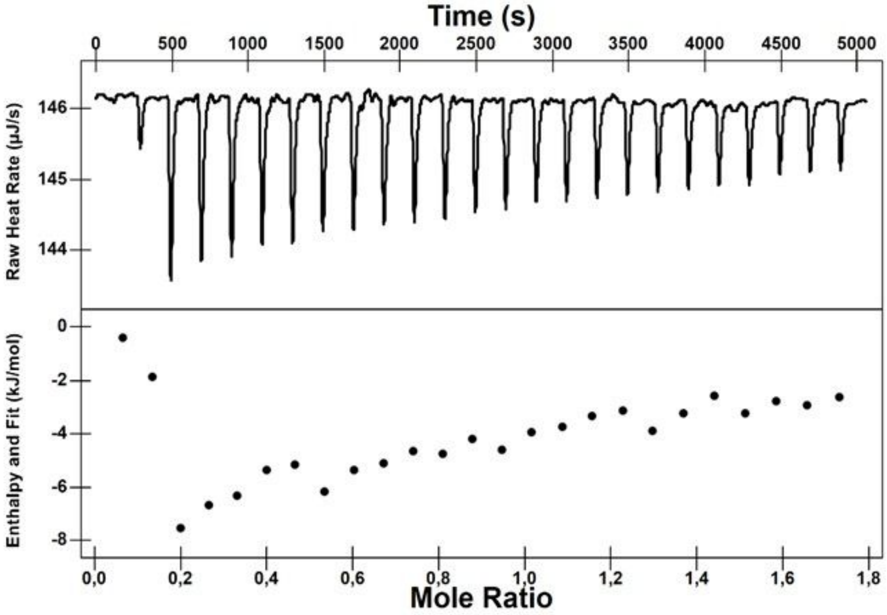

Figure S146: Raw data of the titration of $\mathbf{8^{+}}$ and **CB[8]**.

4.7. ITC data of complex $\mathbf{2}^{2+}$□CB[7]

Assessed concentration of host (CB[7]) in the cell was 104.9 µM. Assessed concentration of guest $\mathbf{2}^{2+}$ in the cell was 2.89 mM (ε_{262nm} = 5297$M^{-1}cm^{-1}$).

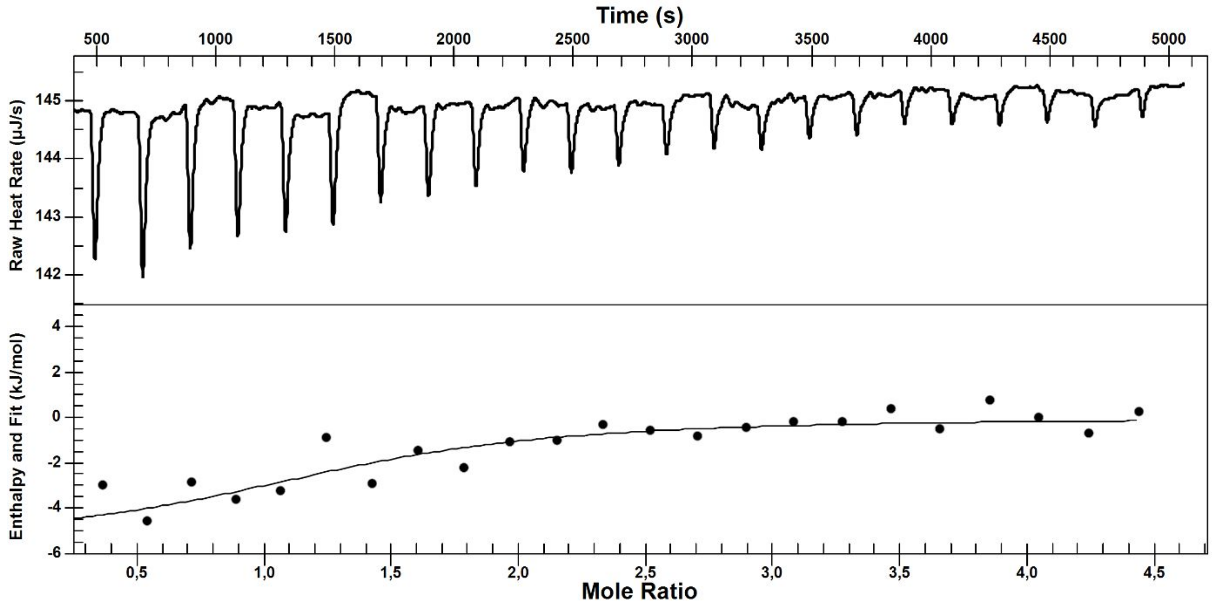

Figure S147: Titration and fitting graphics of **$\mathbf{2}^{2+}$□CB[7].**

Table S22: Titration data of one of the replicates of **2**$^{2+}$□**CB[7]**.

Injection	Y: Area Data (μJ)	Injection Volume (μL)	Moles (Syringe)	Moles (Cell)	X: Mole Ratio	Cell Volume (μL)	Independent Model	Residual
1	-4.5242333	0.86	2.31E-09	9.96E-08	0.0232351 2	950	-19.396049	-
2	-14.391264	6	1.84E-08	9.89E-08	0.1863709 6	950	-135.29833	-
3	-127.92902	6	3.45E-08	9.83E-08	0.3505436 8	950	-135.24583	7.3168021
4	-128.72306	6	5.04E-08	9.77E-08	0.5157598 7	950	-135.16672	6.44365701
5	-154.34918	6	6.62E-08	9.71E-08	0.6820261 6	950	-135.03933	-19.309858
6	-137.33266	6	8.19E-08	9.65E-08	0.8493492 3	950	-134.81433	-2.518329
7	-142.39555	6	9.76E-08	9.59E-08	1.0177357 9	950	-134.3585	-8.0370555
8	-125.55286	6	1.13E-07	9.52E-08	1.1871926 1	950	-133.20121	7.64835584
9	-117.38969	6	1.29E-07	9.46E-08	1.3577264 8	950	-128.66108	11.2713954
10	-98.066837	6	1.44E-07	9.40E-08	1.5293442 5	950	-93.791054	-4.2757826
11	-15.339958	6	1.59E-07	9.35E-08	1.7020528 2	950	-20.230132	4.89017473
12	0.30665601	6	1.74E-07	9.29E-08	1.8758591 1	950	-4.1726529	4.47930896
13	-15.686289	6	1.89E-07	9.23E-08	2.0507701	950	-1.6514662	-14.034823
14	0.92199794	6	2.04E-07	9.17E-08	2.2267928 1	950	-0.872951	1.79494898
15	-10.593408	6	2.19E-07	9.11E-08	2.4039343 1	950	-0.5369654	-10.056443
16	-19.607721	6	2.34E-07	9.05E-08	2.5822017 1	950	-0.3624458	-19.245275
17	-6.6107481	6	2.48E-07	9.00E-08	2.7616021 6	950	-0.2604833	-6.3502647
18	15.6549527	6	2.63E-07	8.94E-08	2.9421428 8	950	-0.1958604	15.8508131
19	-0.005081	6	2.78E-07	8.88E-08	3.1238310 9	950	-0.1523813	0.14730033

20	7.37554802	6	2.92E-07	8.83E-08	3.30667411	950	-0.1217587	7.49730675
21	-10.963578	6	3.06E-07	8.77E-08	3.49067926	950	-0.0993967	-10.864181
22	-2.9677103	6	3.20E-07	8.72E-08	3.67585394	950	-0.0825811	-2.8851293
23	-14.587513	6	3.35E-07	8.66E-08	3.86220557	950	-0.0696257	-14.517887
24	2.53627651	6	3.49E-07	8.61E-08	4.04974165	950	-0.0594389	2.59571536
25	2.95707832	6	3.62E-07	8.55E-08	4.23846969	950	-0.0512883	3.00836659

Table S23: Thermodynamic data of the replicate 1 of **2**$^{2+}$□**CB[7]**.

Models	Variable	Value	Error
Independent	Kd (M)	2.32E-05	7.88E-05
	n	1.459	0.822
	ΔH (kJ/mol)	-3.569	33.26
	Ka (M^{-1})	4.30E+04	
	-TΔS (kJ/mol)	-22.88	
	ΔG (kJ/mol)	-26.45	
	ΔS (J/mol·K)	76.74	
	Confidence Level (%)	95	

Table S24: Thermodynamic data of the replicate 2 of **2**$^{2+}$□**CB[7]**.

Models	Variable	Value	Error
Independent	Kd (M)	5.80E-05	4.00E-05
	n	0.1	0.332
	ΔH (kJ/mol)	-59.05	33.22
	Ka (M^{-1})	1.73E+04	
	-TΔS (kJ/mol)	34.87	
	ΔG (kJ/mol)	-24.18	
	ΔS (J/mol·K)	-117	
	Confidence Level (%)	95	

Table S25: Thermodynamic data of the replicate 3 of **2**$^{2+}$□**CB[7]**.

**Models**	**Variable**	**Value**	**Error**
Independent	Kd (M)	2.55E-05	7.57E-05
	n	1.277	1.054
	ΔH (kJ/mol)	-5.539	67.25
	Ka (M^{-1})	3.92E+04	
	-TΔS (kJ/mol)	-20.68	
	ΔG (kJ/mol)	-26.22	
	ΔS (J/mol·K)	69.36	
	Confidence Level (%)	95	

5.1. ITC data of complex 4^{3+}□CB[7]

Assessed concentration of host (CB[7]) in the cell was 104.9 μM. Assessed concentration of guest $\mathbf{4}^{3+}$ in the cell was 2.69 mM (ε_{262nm} = 18132 $M^{-1}cm^{-1}$).

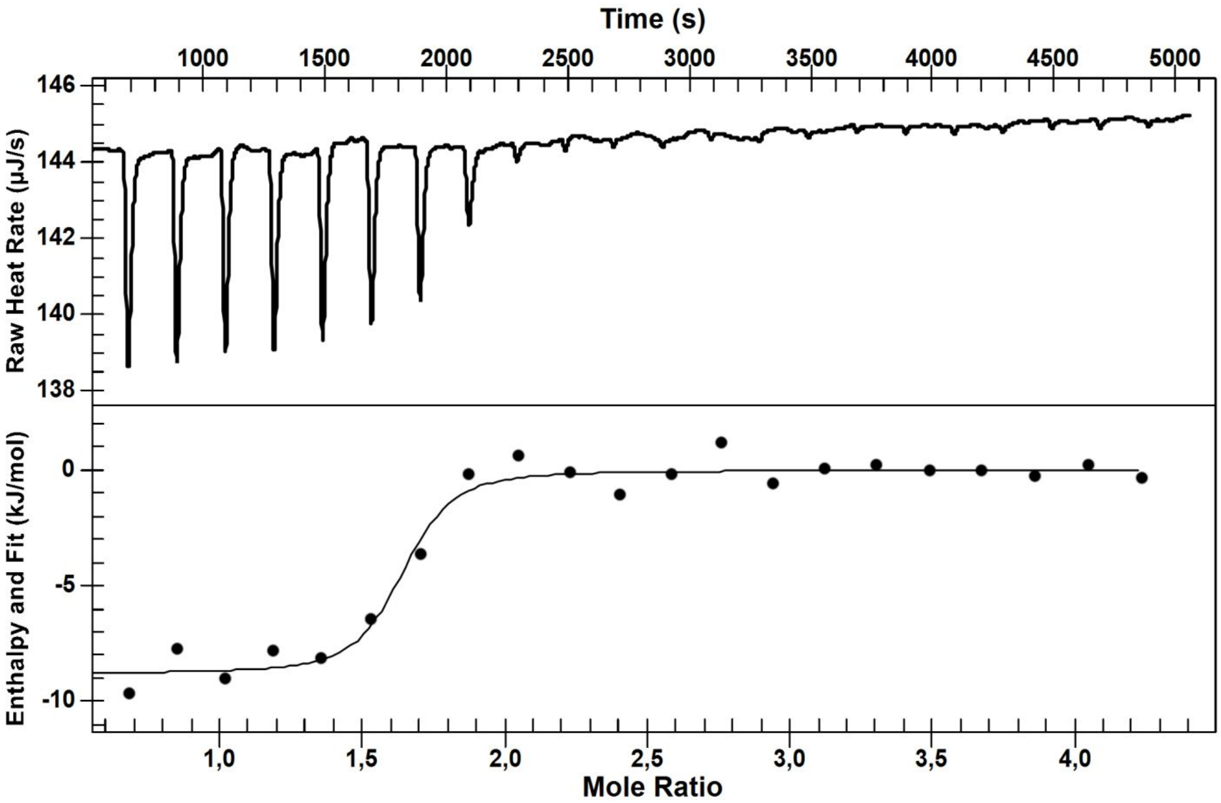

Figure S148: Titration and fitting graphics of $\mathbf{4}^{3+}$**□CB[7].**

Table S26: Titration data of one of the replicates of **4**$^{3+}$□**CB[8]**.

Injection	Y: Area Data (μJ)	Injection Volume (μL)	Moles (Syringe)	Moles (Cell)	X: Mole Ratio	Cell Volume (μL)	Independent Model	Residual
1	-4.5242333	0.86	2.31E-09	9.96E-08	0.02323512	950	-19.396049	-
2	-14.391264	6	1.84E-08	9.89E-08	0.18637096	950	-135.29833	-
3	-127.92902	6	3.45E-08	9.83E-08	0.35054368	950	-135.24583	7.3168021
4	-128.72306	6	5.04E-08	9.77E-08	0.51575987	950	-135.16672	6.44365701
5	-154.34918	6	6.62E-08	9.71E-08	0.68202616	950	-135.03933	-19.309858
6	-137.33266	6	8.19E-08	9.65E-08	0.84934923	950	-134.81433	-2.518329
7	-142.39555	6	9.76E-08	9.59E-08	1.01773579	950	-134.3585	-8.0370555
8	-125.55286	6	1.13E-07	9.52E-08	1.18719261	950	-133.20121	7.64835584
9	-117.38969	6	1.29E-07	9.46E-08	1.35772648	950	-128.66108	11.2713954
10	-98.066837	6	1.44E-07	9.40E-08	1.52934425	950	-93.791054	-4.2757826
11	-15.339958	6	1.59E-07	9.35E-08	1.70205282	950	-20.230132	4.89017473
12	0.30665601	6	1.74E-07	9.29E-08	1.87585911	950	-4.1726529	4.47930896
13	-15.686289	6	1.89E-07	9.23E-08	2.0507701	950	-1.6514662	-14.034823
14	0.92199794	6	2.04E-07	9.17E-08	2.22679281	950	-0.872951	1.79494898
15	-10.593408	6	2.19E-07	9.11E-08	2.40393431	950	-0.5369654	-10.056443
16	-19.607721	6	2.34E-07	9.05E-08	2.58220171	950	-0.3624458	-19.245275
17	-6.6107481	6	2.48E-07	9.00E-08	2.76160216	950	-0.2604833	-6.3502647
18	15.6549527	6	2.63E-07	8.94E-08	2.94214288	950	-0.1958604	15.8508131
19	-0.005081	6	2.78E-07	8.88E-08	3.12383109	950	-0.1523813	0.14730033

20	7.37554802	6	2.92E-07	8.83E-08	3.3066741<br>1	950	-0.1217587	7.49730675
21	-10.963578	6	3.06E-07	8.77E-08	3.4906792<br>6	950	-0.0993967	-10.864181
22	-2.9677103	6	3.20E-07	8.72E-08	3.6758539<br>4	950	-0.0825811	-2.8851293
23	-14.587513	6	3.35E-07	8.66E-08	3.8622055<br>7	950	-0.0696257	-14.517887
24	2.53627651	6	3.49E-07	8.61E-08	4.0497416<br>5	950	-0.0594389	2.59571536
25	2.95707832	6	3.62E-07	8.55E-08	4.2384696<br>9	950	-0.0512883	3.00836659

Table S27: Thermodynamic data of the replicate 1 of $\mathbf{4}^{3+}$□**CB[8]**.

Models	Variable	Value	Error
Independent	Kd (M)	4.32E-07	5.37E-07
	n	1.562	0.052
	ΔH (kJ/mol)	-8.82	0.604
	Ka (M^{-1})	2.32E+06	
	-TΔS (kJ/mol)	-27.51	
	ΔG (kJ/mol)	-36.33	
	ΔS (J/mol·K)	92.27	
	Confidence Level (%)	95	

Table S28: Thermodynamic data of the replicate 2 of $\mathbf{7}^{+}$□**CB[8]**.

Models	Variable	Value	Error
Independent	Kd (M)	1.75E-07	3.11E-07
	n	1.5	0.044
	ΔH (kJ/mol)	-8.394	0.515
	Ka (M^{-1})	5.71E+06	
	-TΔS (kJ/mol)	-30.17	
	ΔG (kJ/mol)	-38.57	
	ΔS (J/mol·K)	101.2	
	Confidence Level (%)	95	

6. SELECTIVE INVERSION RECOVERY KINETICS EXPERIMENTS

The sample for the Selective Inversion Recovery experiment was prepared according to the procedure described in **Section 3.1**, to obtain a mixture of the model **2**$^{2+}$ and the complex **2**$^{2+}$**□CB[8]** in an approximate 1:1 molar ratio. The magnetization transfer spectra were obtained using an inversion recovery sequence with a calibrated 180º pulse. The relaxation times (T1) for the resonances of interest were measured before the magnetization transfer experiment by using the inversion recovery method. In the experiments 32 spectra were collected with a delay between the selective inversion pulse and the spectrum acquisition (d2) ranging from 0.0001 s to 30 s (see **Table S22** and **Table S24**). To assess the rate constant of the guest exchange, the selected signal to invert was the doublet at 6.94 ppm corresponding to one of the signals in the xylene motif of the free guest ($\mathbf{f_{out}}$ in **Figure S147**). The magnetization transfer data was obtained from the relative integrals of the inverted signal and the corresponding equivalent signal in the complex ($\mathbf{f_{in}}$ in **Figure S147**). Then, to determine the rate constant of the phenyl exchange within the complex, the selected signal was the multiplet at 7.85 ppm corresponding to one of the signals of the phenyl groups outside the macrocycle's cavity (**x**´ in **Figure S149**). The magnetization transfer data was obtained from the relative integrals of the inverted signal and the equivalent signal in the phenyl group inside the CB[8]´s cavity (**x** in **Figure S149**). The experimental data was normalized and then fitted by least-squared minimization of the predicted values according to Marquardt´s algorithm and McClung´s formulation for the parameters, using the software CIFIT, to obtain the corresponding exchange rate constants.[4, 5]

[4] Williams, T. J.; Kershaw, A. D.; Li, V.; Wu, X. An Inversion Recovery NMR Kinetics Experiment. *J. Chem. Educ.* **2011**, *88* (5), 665-669.
[5] Bain, A. D.; Cramer, J. A. Slow Chemical Exchange in an Eight-Coordinated Bicentered Ruthenium Complex Studied by One-Dimensional Methods. Data Fitting and Error Analysis. *J. Magn. Reson. A* **1996**, *118* (1), 21-27.

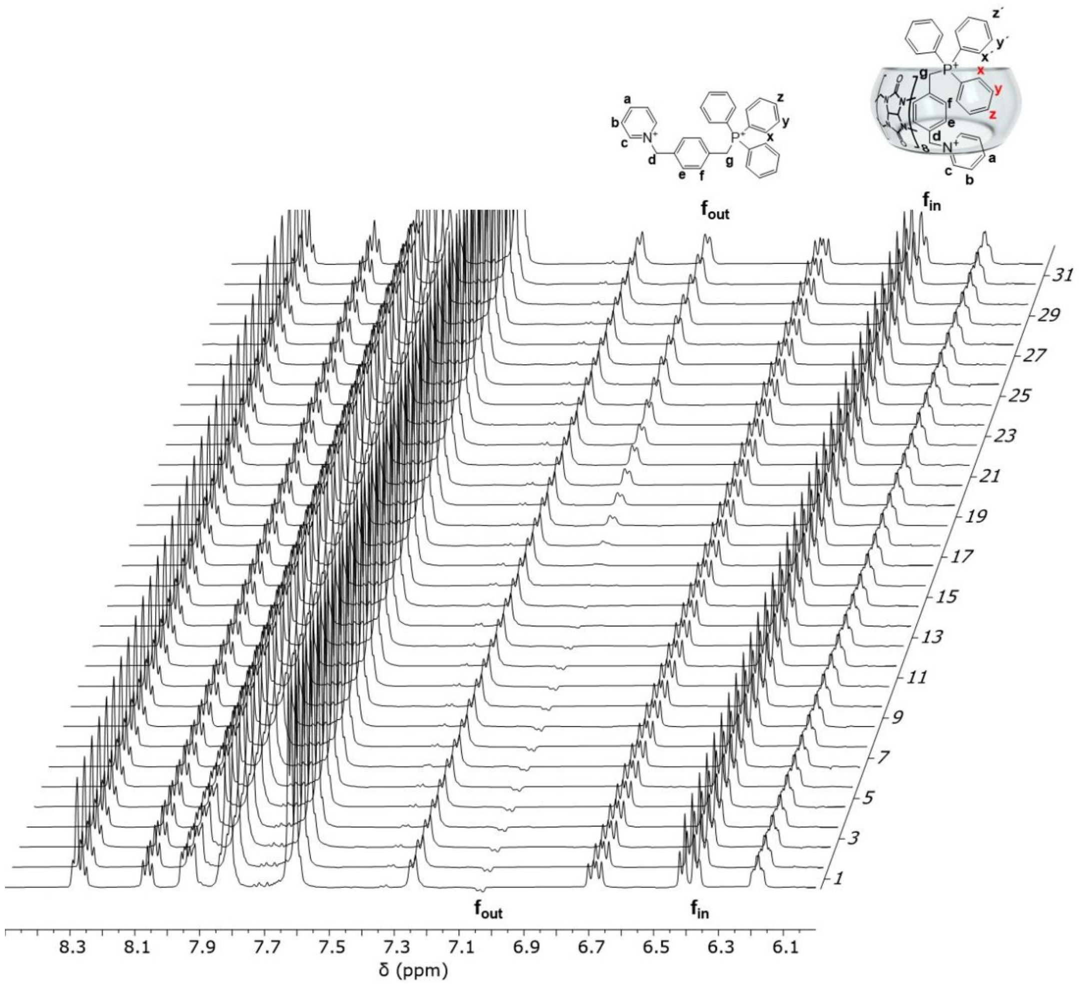

Figure S149: Spectra recollected in ^{1}H NMR (D_2O 500 MHz**)** selective inversion recovery experiment of a mixture of **2**$^{2+}$ and the complex **2**$^{2+}$□**CB[8]** in a 1:1 molar ratio. Inverted signal of the free guest and its corresponding equivalent in the complex are marked as $\mathbf{f_{out}}$ and $\mathbf{f_{in}}$ respectively. From the bottom spectrum to the top spectrum the delay between the selective inversion pulse and spectrum acquisition (d2) increases.

Table S29: Experimental data of the SIR experiment for the **f** signal of $\mathbf{2^{2+}}$□**CB[8].**

	f_{out}		f_{in}	
d2 (s)	**Integral (7.000 - 6.894 ppm)**	**Normalization**	**Integral (6.300-6.229 ppm)**	**Normalization**
0.0001	-12.9101	-0.128631495	120.645	0.973430264
0.0002	-12.9752	-0.129280128	122.297	0.986759509
0.0004	-12.4011	-0.123560006	123.03	0.992673756
0.0008	-12.5226	-0.124770587	120.507	0.972316804
0.001	-13.0884	-0.130408011	123.938	1
0.002	-13.109	-0.130613262	119.617	0.965135794
0.004	-12.3746	-0.12329597	120.543	0.972607271
0.008	-12.4948	-0.124493598	121.631	0.981385854
0.012	-11.4762	-0.114344642	119.891	0.967346577
0.016	-11.5728	-0.115307129	121.297	0.978690958
0.02	-10.6737	-0.106348827	122.448	0.98797786
0.04	-9.24939	-0.092157525	121.974	0.984153367
0.06	-7.98504	-0.079560006	121.509	0.980401491
0.08	-3.56829	-0.035553131	121.264	0.978424696
0.1	-5.78052	-0.057594978	121.026	0.976504381
0.2	1.20605	0.012016639	119.969	0.967975923
0.3	8.47148	0.084406715	120.887	0.975382853
0.4	13.3132	0.132647835	120.677	0.973688457
0.8	33.5236	0.334016839	118.687	0.957632042
1	42.6744	0.425192049	120.556	0.972712163
1.4	54.3818	0.541840283	119.031	0.960407623
1.8	65.1792	0.649421611	121.969	0.984113024
2	70.5943	0.703375679	120.921	0.975657183
3	82.6996	0.823988442	120.487	0.972155433
4	91.5149	0.911820854	121.534	0.980603205
5	95.2218	0.948755044	121.308	0.978779712
6	98.931	0.985712151	122.483	0.988260259
7	98.4151	0.980571913	120.803	0.974705094
8	98.5552	0.981967817	118.775	0.958342074
10	100.271	0.999063419	122.75	0.990414562
20	98.8104	0.984510537	118.891	0.959278026
30	100.365	1	118.684	0.957607836

Table S30: Plot data of the SIR experiment for the **f** signal of **2^{2+}□CB[8].**

	**Calculated**		**Observed**		**Calculated-Observed**	
d2 (s)	f_{in}	f_{out}	f_{in}	f_{out}	f_{in}	f_{out}
0.0001	0.9809	-0.1238	0.9734	-0.1286	0.0075	0.0049
0.0002	0.9809	-0.1237	0.9868	-0.1293	-0.0059	0.0056
0.0004	0.9809	-0.1235	0.9927	-0.1236	-0.0118	0
0.0008	0.9809	-0.1232	0.9723	-0.1248	0.0086	0.0015
0.001	0.9809	-0.1231	1	-0.1304	-0.0191	0.0073
0.002	0.9808	-0.1224	0.9651	-0.1306	0.0157	0.0083
0.004	0.9808	-0.1209	0.9726	-0.1233	0.0081	0.0024
0.008	0.9806	-0.1179	0.9814	-0.1245	-0.0008	0.0066
0.012	0.9804	-0.115	0.9673	-0.1143	0.0131	-0.0007
0.016	0.9803	-0.1121	0.9787	-0.1153	0.0016	0.0032
0.02	0.9801	-0.1092	0.988	-0.1063	-0.0079	-0.0028
0.04	0.9793	-0.0947	0.9842	-0.0922	-0.0048	-0.0025
0.06	0.9786	-0.0804	0.9804	-0.0796	-0.0018	-0.0009
0.08	0.9779	-0.0664	0.9784	-0.0356	-0.0005	-0.0308
0.1	0.9772	-0.0525	0.9765	-0.0576	0.0007	0.0051
0.2	0.9742	0.0142	0.968	0.012	0.0062	0.0022
0.3	0.9717	0.0766	0.9754	0.0844	-0.0036	-0.0078
0.4	0.9698	0.135	0.9737	0.1326	-0.0039	0.0023
0.8	0.9655	0.333	0.9576	0.334	0.0079	-0.001
1	0.9648	0.414	0.9727	0.4252	-0.0079	-0.0111
1.4	0.965	0.5473	0.9604	0.5418	0.0046	0.0054
1.8	0.9662	0.6497	0.9841	0.6494	-0.0179	0.0003
2	0.967	0.6918	0.9757	0.7034	-0.0087	-0.0116
3	0.9709	0.8364	0.9722	0.824	-0.0013	0.0124
4	0.9736	0.9121	0.9806	0.9118	-0.007	0.0003
5	0.9752	0.9517	0.9788	0.9488	-0.0035	0.003
6	0.9761	0.9726	0.9883	0.9857	-0.0121	-0.0132
7	0.9766	0.9835	0.9747	0.9806	0.0019	0.0029
8	0.9769	0.9892	0.9583	0.982	0.0185	0.0073
10	0.9771	0.9938	0.9904	0.9991	-0.0133	-0.0052
20	0.9772	0.9956	0.9593	0.9845	0.0179	0.0111
30	0.9772	0.9956	0.9576	1	0.0196	-0.0044

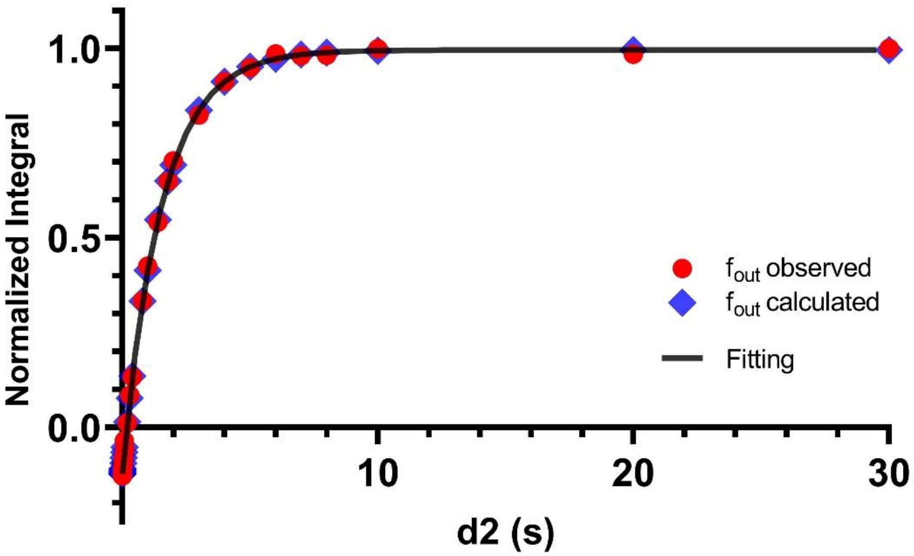

Figure S150: Observed (red) and calculated (blue) data and fitting (black) graphic of the SIR experiment for $\mathbf{f_{out}}$ signal.

In the case of the guest exchange, since the concentration of free host is constant during the magnetization transfer process, the chemical equilibrium (Eq. 1) can be treated as consisting of two unimolecular elementary reactions (Eq. 2): the forward reaction in which the species $\mathbf{2}^{2+}$ is converted into $\mathbf{2}^{2+}$⊂CB[8]; and the backward reaction in which $\mathbf{2}^{2+}$⊂CB[8] is converted into $\mathbf{2}^{2+}$.

$$\mathbf{2}^{2+} + CB[8] \underset{k_{out}}{\overset{k_{in}}{\leftrightarrow}} \mathbf{2}^{2+}\text{?CB[8] (Eq 1)}$$

$$\mathbf{2}^{2+} \underset{k_{-1}}{\overset{k_{1}}{\leftrightarrow}} \mathbf{2}^{2+}\text{?CB[8] (Eq 2)}$$

According to this equilibrium, the exchange rate constant observed during the NMR experiments is $k_{ex} = k_1 + k_{-1}$, where the rate constant k_{-1} corresponds to the k_{out} (Eq. 3) of the guest exchange and the rate constant k_1 is directly proportional to k_{in} and the concentration of free CB[8] (Eq 4). The latter can be determined from the relative populations of each species in solution p, as determined by NMR integration, along with the thermodynamic constant K_a, which is the ratio between $k_{in}$and k_{out} (Eq. 5).

$$k_{-1} = k_{out} \text{ (Eq. 3)}$$

$$k_1 = k_{in}\cdot[CB8]_{free} \text{ (Eq. 4)}$$

$$[CB8]_{free} = \frac{p_{\mathbf{2}^{2+}\subset CB[8]}}{p_{\mathbf{2}^{2+}} \cdot K_a} \text{ (Eq. 5)}$$

Hence, for the guest exchange by selectively inverting the signal **f$_{out}$** corresponding to one of the two doublets of the xylyl moiety on the uncomplexed cation, the fitting of the obtained data resulted in $k_{ex\ (guest\ exchange)} = k_1 + k_{-1} = (0.32 \pm 0.09)s^{-1}$. Using the above-mentioned equations (Eq. 1-5) and considering the relative populations of free and bound guest in solution ($p_{\mathbf{2}^{2+}} = 0.44$ and $p_{\mathbf{2}^{2+}\subset CB[8]} = 0.56$) and the thermodynamic constant obtained by ITC ($K_a = 1.24 \cdot 10^7\ M^{-1}$), the kinetics for the guest exchange are $k_{in} = 1.69 \cdot 10^7 s^{-1} \cdot M^{-1}$ and $k_{out} = 0.14\ s^{-1}$.

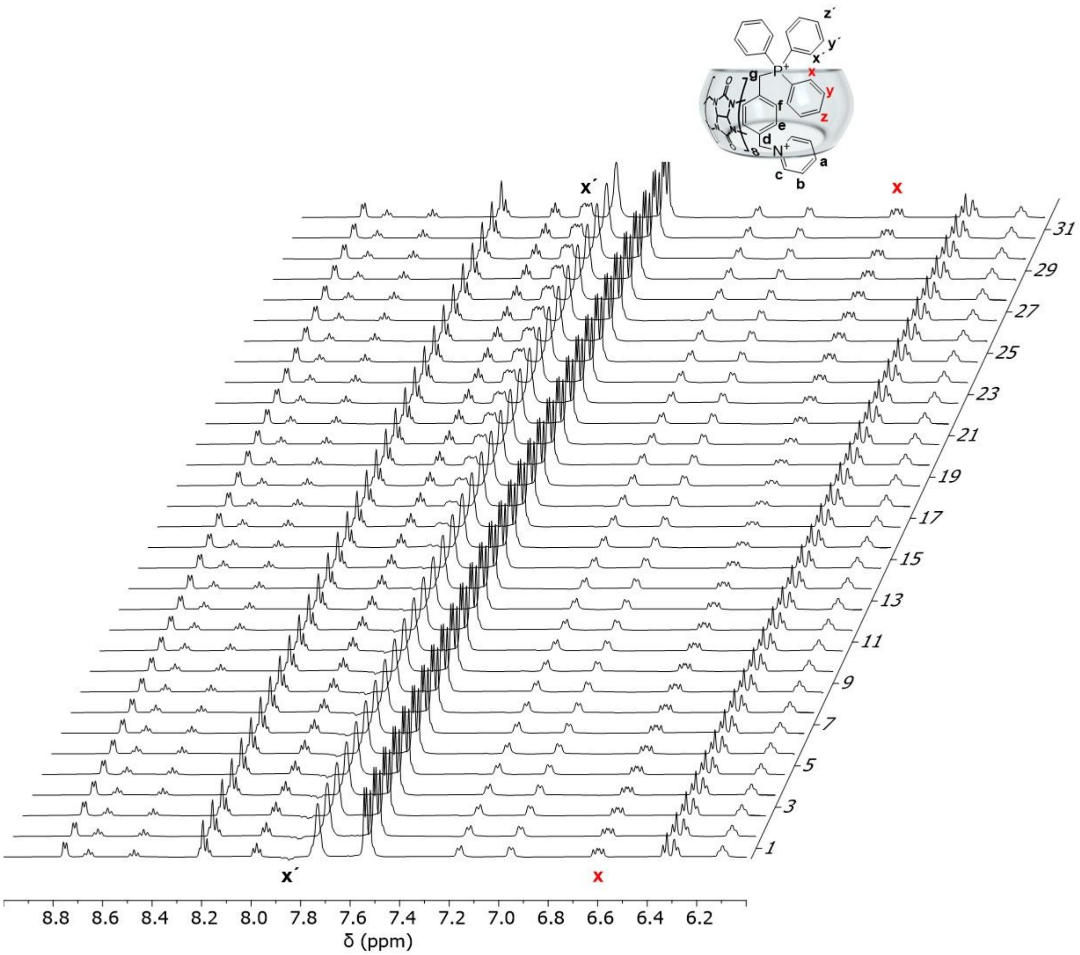

Figure S151: Spectra recollected in ^{1}H NMR (D_2O 500 MHz**)** selective inversion recovery experiment of a mixture of $\mathbf{2}^{2+}$ and the complex $\mathbf{2}^{2+}$□**CB[8]** in a 1:1 molar ratio. Inverted signal of the phenyl groups outside the cavity of CB[8] and its corresponding equivalent inside the cavity are marked as **x´** and **x,** respectively. From the bottom spectrum to the top spectrum the delay between the selective inversion pulse and spectrum acquisition (d2) increases.

Table S 31 Experimental data of the SIR experiment for the **x** and **x´** signal of **2^{2+}□CB[8].**

	x´		***x***	
d2 (s)	**Integral (7.901 - 7.800 ppm)**	**Normalization**	**Integral (6.650 - 6.550 ppm)**	**Normalization**
0.0001	-28.1246	-0.112683652	117.622	0.951195646
0.0002	-26.3687	-0.105648486	112.754	0.911828687
0.0004	-27.1648	-0.10883813	113.689	0.919389925
0.0008	-27.0604	-0.108419842	113.907	0.921152866
0.001	-29.9695	-0.120075404	113.813	0.920392699
0.002	-26.3537	-0.105588387	112.562	0.910276005
0.004	-29.0646	-0.116449844	112.694	0.911343474
0.008	-23.7027	-0.094966926	112.578	0.910405396
0.012	-22.643	-0.090721146	113.183	0.915297961
0.016	-21.6491	-0.086738999	110.807	0.896083521
0.02	-18.363	-0.073572954	109.33	0.884139191
0.04	-10.146	-0.04065083	103.399	0.836175874
0.06	-3.85137	-0.015430848	101.673	0.822217909
0.08	5.56181	0.022283875	94.4311	0.763653493
0.1	12.8717	0.051571584	97.3337	0.787126487
0.2	41.8894	0.167833518	80.4723	0.650770276
0.3	68.3999	0.274050138	77.2628	0.624815417
0.4	86.8709	0.348055804	73.2968	0.592742829
0.8	133.919	0.536558102	73.5563	0.594841376
1	150.079	0.601304545	75.7543	0.61261635
1.4	173.831	0.696468995	86.1463	0.696655264
1.8	191.9	0.768864012	91.357	0.738793598
2	199.745	0.800295686	97.4435	0.788014427
3	222.922	0.893156349	107.479	0.869170366
4	235.536	0.943695435	113.909	0.92116904
5	242.958	0.973432323	121.155	0.979766612
6	249.413	0.999294841	121.201	0.980138609
7	248.401	0.995240175	121.883	0.985653865
8	245.076	0.981918274	120.492	0.974405007
10	249.589	1	123.657	1
20	245.255	0.982635453	119.51	0.966463686
30	249.314	0.998898189	121.147	0.979701917

Table S32: Plot data of the SIR experiment for x and x´ signal of 2^{2+}□CB[8].

	Calculated		Observed		Calculated-Observed	
d2 (s)	***x***	***x´***	***x***	***x´***	***x***	***x´***
0.0001	0.868	-0.1351	0.9512	-0.1127	-0.0832	-0.0224
0.0002	0.8679	-0.1349	0.9118	-0.1056	-0.0439	-0.0292
0.0004	0.8678	-0.1343	0.9194	-0.1088	-0.0516	-0.0255
0.0008	0.8675	-0.1333	0.9212	-0.1084	-0.0537	-0.0249
0.001	0.8674	-0.1328	0.9204	-0.1201	-0.053	-0.0127
0.002	0.8667	-0.1302	0.9103	-0.1056	-0.0436	-0.0246
0.004	0.8654	-0.125	0.9113	-0.1164	-0.046	-0.0085
0.008	0.8627	-0.1148	0.9104	-0.095	-0.0477	-0.0198
0.012	0.8602	-0.1047	0.9153	-0.0907	-0.0551	-0.014
0.016	0.8577	-0.0948	0.8961	-0.0867	-0.0384	-0.0081
0.02	0.8552	-0.0851	0.8841	-0.0736	-0.0289	-0.0115
0.04	0.8437	-0.0389	0.8362	-0.0407	0.0075	0.0017
0.06	0.8333	0.0036	0.8222	-0.0154	0.0111	0.019
0.08	0.824	0.0427	0.7637	0.0223	0.0604	0.0204
0.1	0.8157	0.0788	0.7871	0.0516	0.0286	0.0272
0.2	0.7861	0.2219	0.6508	0.1678	0.1353	0.0541
0.3	0.7705	0.3203	0.6248	0.2741	0.1456	0.0463
0.4	0.7634	0.3905	0.5927	0.3481	0.1707	0.0424
0.8	0.7704	0.5448	0.5948	0.5366	0.1756	0.0082
1	0.7804	0.5917	0.6126	0.6013	0.1678	-0.0096
1.4	0.8006	0.6661	0.6967	0.6965	0.104	-0.0304
1.8	0.8185	0.7261	0.7388	0.7689	0.0797	-0.0427
2	0.8264	0.7523	0.788	0.8003	0.0384	-0.048
3	0.8572	0.8541	0.8692	0.8932	-0.012	-0.0391
4	0.8773	0.9202	0.9212	0.9437	-0.0439	-0.0235
5	0.8903	0.9633	0.9798	0.9734	-0.0894	-0.0101
6	0.8988	0.9913	0.9801	0.9993	-0.0813	-0.008
7	0.9044	1.0095	0.9857	0.9952	-0.0813	0.0143
8	0.908	1.0214	0.9744	0.9819	-0.0664	0.0395
10	0.9118	1.0341	1	1	-0.0882	0.0341
20	0.9146	1.0433	0.9665	0.9826	-0.0518	0.0607
30	0.9147	1.0435	0.9797	0.9989	-0.065	0.0446

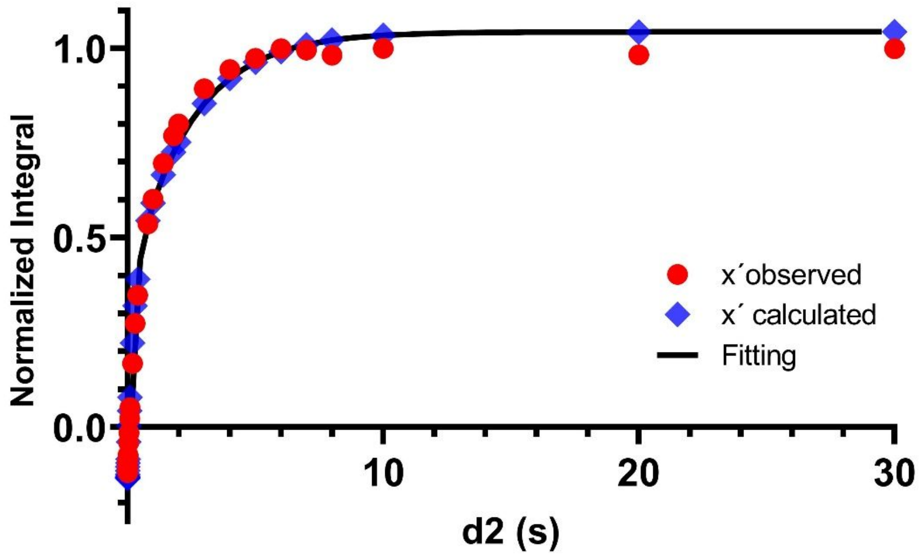

Figure S152: Observed (red) and calculated (blue) data and fitting (black) graphic of the SIR experiment for **x´** signal.

The same protocol applied to the signal (**x´**) corresponding to the outside nuclei of the phenyl units in the complex $\mathbf{2}^{2+}\subset$CB[8] allowed us to derive $k_{ex\,(phenyl\ exchange)} = k_1 + k_{-1} = (6.80 \pm 0.44)s^{-1}$. As in the previous exchange, the rate constant k_{-1} corresponds to the k_{out} of the phenyl exchange, and the rate constant k_1 is directly proportional to k_{in} and the concentration of outside phenyl moieties. Since all conformers are equal in terms of energy, the free energy associated with the phenyl exchange ΔG^0 is 0, and consequently, K_a corresponds to 1. Using the previously described equations (Eq. 1-5) and considering that the populations of phenyl rings outside and inside observed by NMR are in a 2:1 ratio respectively, the kinetics for the phenyl exchange are $k_{in} = 4.53\ s^{-1}$ and $k_{out} = 2.26\ s^{-1}$.

Table S33: Rate constants obtained from the SIR experiments for both processes studied (Guest exchange and Phenyl exchange).

Process	k_{in}	k_{out}
Guest exchange (f_{out} and f_{in} SIR))	$1.69 \cdot 10^7\ M^{-1} \cdot s^{-1}$	$0.14\ s^{-1}$
Phenyl exchange (x´ and x SIR)	$4.53\ s^{-1}$	$2.26\ s^{-1}$

7. COMPUTATIONAL DETAILS:

All quantum mechanical calculations reported in this work were performed using the free-available program packages ORCA 5.0.3 (dispersion-corrected density functional theoretical methods, DFT-D), [6] and xTB 6.6.0 (tight binding semiempirical quantum-mechanical methods, tb-SQM).[7] Unless otherwise indicated, computations were carried out with default settings.

Cartesian coordinates in .xyz format for all the structures at the different levels of theory discussed in this work are available as supplementary information on the **S1.zip** file.

7.1. Representative minimum structures.

Initial geometries for the different guests (**G**), CB[7,8] hosts (**H**), and the corresponding complexes (**G·H**), were generated by hand using the AVOGADRO software,[8] and further optimized by employing the GFN2-xTB tb-SQM,[9] including the analytical linearized Poisson-Boltzmann (ALPB) solvation model (water). [10]

Due to their rigidity, no searches for the lowest-lying structures were conducted in the case of CB[7/8], with the initial geometries of both at the GFN2-xTB/ALPB(water) level taken as representative, and being further optimized with the composite DFT-D method r^2SCAN-3c,[11] including solvation effects in water by means of the CPCM model,[12] and checking the true nature of each geometry as local minimum on the potential free energy surfaces by frequency calculations. In the case of the triphenylphosphonium guests **G**, starting from the hand-generated initial structures, representative conformers were obtained at the GFN2-xTB/ALPB(water) level of theory, by conformational searches with the utility/driver for the

---

[6] F. Neese, *WIREs Comput Mol Sci*. **2022**, *12*, e1606.
[7] Bannwarth, C.; Caldeweyher, E.; Ehlert, S.; Hansen, A.; Pracht, P.; Seibert, J.; Spicher, S.; Grimme, S. Extended tight-binding quantum chemistry methods. *WIREs Comput. Mol. Sci.* **2021**, 11, e1493.
[8] Hanwell, M. D.; Curtis, D. E.; Lonie, D. C.; Vandermeersch, T.; Zurek, E.; Hutchison, G. R. Avogadro: An advanced semantic chemical editor, visualization, and analysis platform. *J. Cheminform.* **2012**, *4*, 17.
[9] Bannwarth, C.; Ehlert, S.; Grimme, S. GFN2-xTB—An Accurate and Broadly Parametrized Self-Consistent Tight-Binding Quantum Chemical Method with Multipole Electrostatics and Density-Dependent Dispersion Contributions. *J. Chem. Theory Comput.* **2019**, *15*, 1652-1671.
[10] Ehlert, S.; Stahn, M.; Spicher, S.; Grimme, S. Robust and Efficient Implicit Solvation Model for Fast Semiempirical Methods. *J. Chem. Theory Comput.* **2021**, 17, 4250-4261.
[11] Grimme, S.; Hansen, A.; Ehlert, S.; Mewes, J.-M. r2SCAN-3c: A "Swiss army knife" composite electronic-structure method" *J. Chem. Phys.* **2021**, *154*, 064103.
[12] Barone, V.; Cossi, M. Quantum Calculation of Molecular Energies and Energy Gradients in Solution by a Conductor Solvent Model. *J. Phys. Chem. A* **1998,** *102*, 1995-2001.

xTB software CREST (conformer-rotamer sampling tool),[13] employing standard settings. The lowest lying structures obtained in each case were subsequently re-optimized using the r^2SCAN-3c/CPCM(water) scheme, checking the true nature of each geometry as local minimum on the potential free energy surfaces by frequency calculations. Geometries of representative structures for the guests are depicted in **Figure S151**.

In the case of the inclusion/exclusion complexes, representative binding modes were obtained by applying the recently reported AISS docking protocol,[14] developed by Grimme *et al* at the GFN2-xTB/ALPB(water) level of theory and as implemented in the software xTB 6.6.0, starting in each case from the representative structures discussed above for host and guest. The best pose obtained in each case was subsequently re-optimized at the r^2SCAN-3c/CPCM(water), checking by frequency calculations the true nature of each geometry as local minimum on the potential free energy surfaces. Geometries of the representative binding modes obtained through this method are depicted in **Figure S152** (CB[8]) and **S153** (CB[7]).

---

[13] Pracht, P.; Bohle, F.; Grimme, S. Automated exploration of the low-energy chemical space with fast quantum chemical methods. *Phys. Chem. Chem. Phys.* **2020**, *22*, 7169-7192.

[14] Plett, C.; Grimme, S. Automated and Efficient Generation of General Molecular Aggregate Structures, *Angew. Chem. Int. Ed.* **2023**, *62*, e202214477.

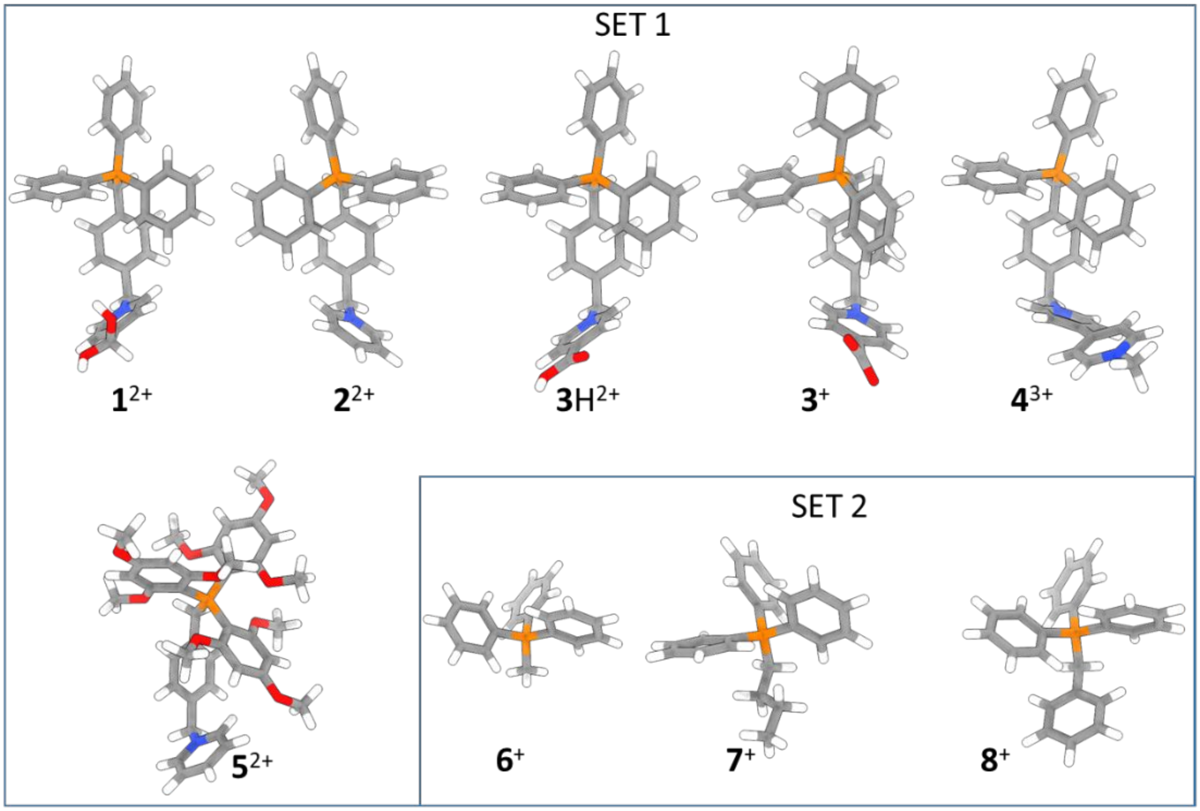

Figure S153: Stick depiction of the representative structures of alkyltriphenylphosphonioum cations discussed in this work, obtained after standard CREST runs at the GFN2-xTB/ALPB(H_2O) level, and re-optimized as local minima (zero imaginary frequencies) located on the corresponding potential energy surface at the r^2SCAN-3c/CPCM(water) level of theory. Color scheme: carbon, grey; nitrogen, blue; oxygen, red; phosphorous, orange; hydrogen, white.

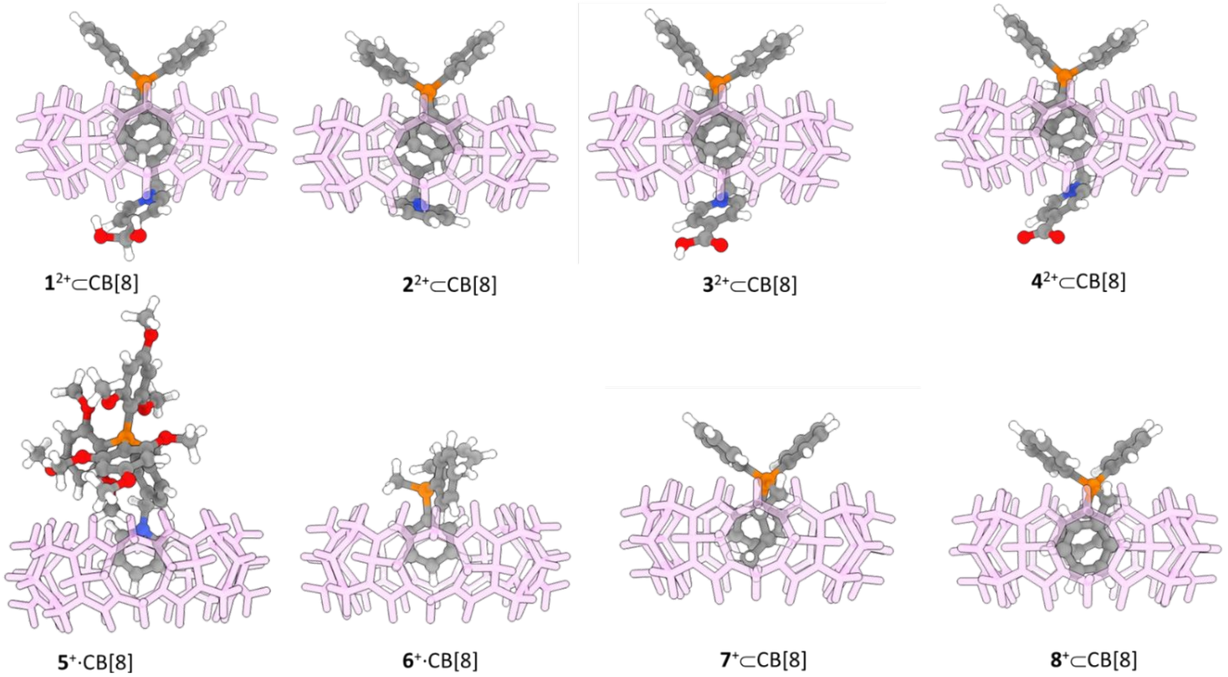

Figure S154: Mixed stick (CB[8])/ball-and-stick(**G**) depiction of the representative structures of **G**·CB[8] aggregates discussed in this work, obtained after aISS runs with standard settings at the GFN2-xTB/ALPB(H_2O) level, and re-optimized as local minima (zero imaginary frequencies) located on the corresponding potential energy surface at the r^{2}SCAN-3c/CPCM(water) level of theory. Color scheme: CB[8], semi-transparent pink sticks; Guests: carbon, grey; nitrogen, blue; oxygen, red; phosphorous, orange; hydrogen, white.

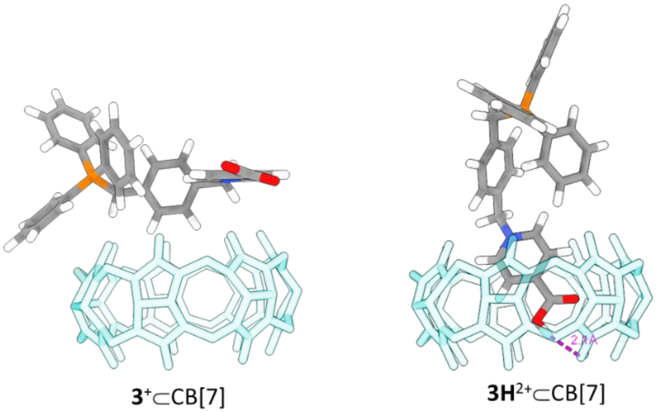

Figure S155: Mixed stick (CB[8])/ball-and-stick(**G**) depiction of the representative structures of **G**·CB[7] aggregates discussed in this work, obtained after aISS runs with standard settings at the GFN2-xTB/ALPB(H_2O) level, and re-optimized as local minima (zero imaginary frequencies) located on the corresponding potential energy surface at the r^{2}SCAN-3c/CPCM(water) level of theory. Color scheme: CB[7], semi-transparent green sticks; Guests: carbon, grey; nitrogen, blue; oxygen, red; phosphorous, orange; hydrogen, white.

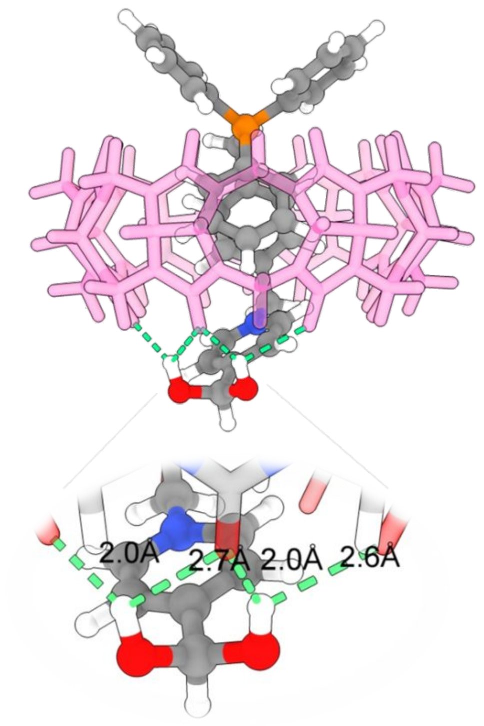

Figure S156: Mixed stick (CB[8])/ball-and-stick(1^{2+}) depiction of the representative structures of 1^{2+}⊂CB[8] at the r^2SCAN-3c/CPCM(water), showing hydrogen bonds as dashed green lines with distance labels.

7.2. Free energy calculations at r^{2}SCAN3c/CPCM(H_2O) level of theory.

Free energies for the bimolecular association processes **H** + **G** ⇌ **G·H** were calculated, using the representative structures for each species as discussed above, and following the supramolecular approach:

$$\Delta G^{o}_{aq} = G^{o}_{aq}\mathbf{G{\cdot}H} - G^{o}_{aq}\mathbf{G} - G^{o}_{aq}\mathbf{H} + 1.89 \text{ (Eq. 6)}$$

where for each species X (= **H**, **G** or **H·G**) in eq.1, the free energy in aqueous solution was computed as:

$$G^{o}_{aq}(\mathbf{X}) = \left[E^{DFT}_{gas}(\mathbf{X}) + \delta_{\text{solv}}(\mathbf{X})\right] + G^{o}_{aq,mrrho}(\mathbf{X}) \text{ (Eq. 7)}$$

Consequently, each compound **X** was minimized using the composite electronic structure method r^{2}SCAN-3c$^{/CPCM(water)}$ and the different contributions obtained at this level of theory after frequency calculations, including the thermostatical corrections on energy to free energy $G^{o}_{aq,mrrho}(\mathbf{X})$, following the modified rigid rotor harmonic oscillator approach. Finally, the concentration-induced free-energy shift of +1.89 kcal mol^{-1} was included in the reported values of ΔG^{o}_{aq} computed according to Eq.6), as required from the change of the estimated values of free energies for each species in solvated gas phase at 1 atm to 1 mol L^{-1} in solution. The obtained results are compiled in **Table S27** and **S28**.

Table S34: Targeted quantities $G^{o}_{aq}(X)$ at the r^{2}SCAN-3c/CPCM(water) level of theory for each of the local minima of GUEST and HOST and discussed in this work, and used for the estimation of their free energies of binding in water (see **Table S28**).

SPECIES[a]	$G^{o}_{aq}(X)$ (ha)
**CB7**	-4210.883403
**CB8**	-4812.435428
**1**$^{2+}$	-1782.933724
**2**$^{2+}$	-1593.215089
**3**H^{2+}	-1781.770525
**3**$^{+}$	-1781.335419
**4**$^{3+}$	-1879.893899
**5**$^{2+}$	-2623.529833
**6**$^{+}$	-1075.615416
**7**$^{+}$	-1193.432290

**8^{+}**	-1306.537410

Table S35: Targeted quantities $\boldsymbol{G^{\circ}_{gas,mrrho}(X)}$ r²SCAN-3c/CPCM(water) level of theory for each of the local minima of inclusion and exclusion complexes discussed in this work, and used for the estimation of their free energies of binding $\Delta\boldsymbol{G^{\circ}_{aq}}$ following Eq. 6.

**Complex**[a]	$G^{\circ}_{aq}\,(X)$ **(ha)**	ΔG°_{aq} **(kcal/mol, eq. 1)**
**1**$^{2+}$⊂CB[8]	-6595.395786	-18.6
**2**$^{2+}$⊂CB[8]	-6405.673312	-16.2
**3**H^{2+}⊂CB[8]	-6594.226631	-14.9
**3**$^{+}$⊂CB[8]	-6593.789800	-13.9
**3**H^{2+}⊂CB[7]	-5992.655440	-2.84
**3**$^{+}$⊂CB[7]	-5992.210121	3.6
**4**$^{3+}$⊂CB[8]	-6692.355179	-18.1
**5**$^{2+}$⊂CB[8]	-7435.960489	1.1
**6**$^{+}$⊂CB[8]	-5888.046500	0.8
**7**$^{+}$⊂CB[8]	-6005.874919	-6.4
**8**$^{+}$⊂CB[8]	-6118.984785	-9.4

7.3. Kinetic profiles for the complexation of guests 2^{2+} and 7^{+} at the GFN-xTB/alpb(H_2O) level of theory.

7.3.1. Guest 2^{2+}

In order to tackle a potential binary dissociative mechanism for the ingression/egression of the guest into/from the cavity of the host, we considered as starting points the representative minima ($\mathbf{I_m}$) for **2**$^{2+}$□CB[8], obtained at the GFN2-xTB/alpb(H_2O) level as discussed in **Section 6.1**. Then two possible options for the complete attachment/detachment of the guest were considered: displacing the guest arbitrarily to the right or left, regarding the centre of mass of the complex and along the C8 rotational axis of the host (**Figure S155**). Subsequently, the obtained geometries were optimized at the GFN2-xTB/alpb(H_2O) level of theory, with the resulting structures used as tentative inputs ($\mathbf{tI_0^{\pm}}$) for the calculation of the minimum energy paths (MEPs) connecting $\mathbf{tI_0^{\pm}}$ and $\mathbf{I_m}$, using the Nudged Elastic Band (NEB) method, [15] with standard settings and as implemented in ORCA.

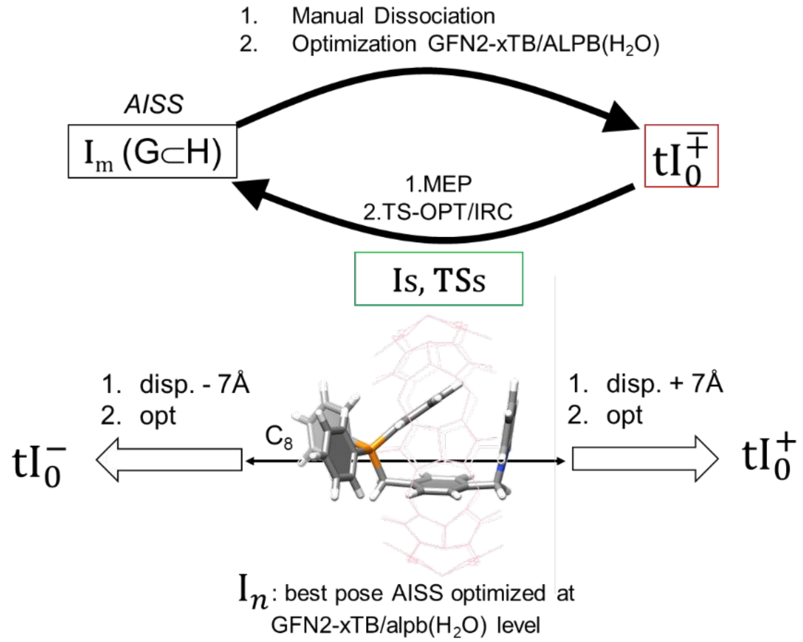

Figure S157: Strategy used for the generation of tentative initial structures for the ingression-egression of a Guest from or into the cavity of CB[8].

[15] Ásgeirsson, V.; Birgisson, B. O.; Bjornsson, R.; Becker, U.; Neese, F.; Riplinger, C.; Jónsson, H.; Nudged Elastic Band Method for Molecular Reactions Using Energy-Weighted Springs Combined with Eigenvector Following, *J. Chem. Theory Comput.*, **2017**, *17*, 4929-4945.

From this point, the following protocol was applied, with the required calculations performed at the GFN2-xTB/alpb(H_2O) level of theory as implemented in ORCA:

A) if the NEB algorithm was found to converge to the MEP with **NO** intermediate minima found, the resulting transition state proposed by the software was optimized, verified as having one and only one imaginary vibration mode by frequency calculations, and the connection of the obtained transition states with I_m and $\mathbf{tI_0^-}$/ $\mathbf{tI_0^+}$ confirmed by intrinsic reaction path calculations (IRC).

B) if the algorithm suggested intermediate minima on the proposed MEP, the initially-proposed path was conveniently divided, and the resulting paths re-evaluated as in A).

C) Steps A) and B) were repeated as required, until the full multistep path connecting the dissociated and associated complexes was found, including their corresponding minima ($\mathbf{I}_n$, n = 1,2,3, ...) and transition states (TS_n, n = 1, 2,3, …). The obtained **paths A** and **B** for guest $\mathbf{2}^{2+}$are depicted in **Figure S156**, with targeted energy quantities and frequencies being summarized in **Table S29**.

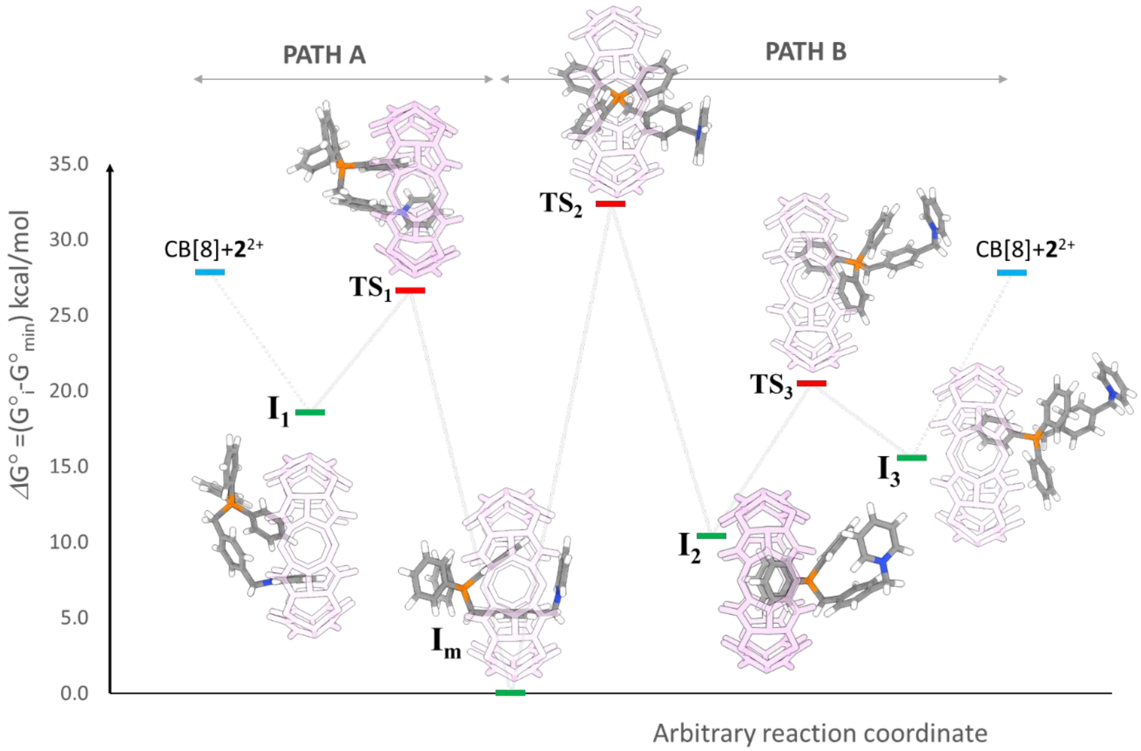

Figure S158: Comparison of reaction paths A and B found for the ingression-egression of guest $\mathbf{2}^{2+}$ at the GFN2-xTB/ALPB(H_2O) level. $\Delta G°$ barriers calculated from $\boldsymbol{G^{\circ}_{aq}(X)}$ (obtained from frequency calculations and summarised in Table S2.), and normalized with I_m as reference. Colour scheme as in **Figure S154**.

Table S36: Targeted quantities $\boldsymbol{G^{o}_{aq}(X)}$ at the GFN2-xTB/ALPB(H_2O) level for each of the local minima and transition states found for the ingression-egression of guest 2^{2+} from and into the cavity of CB[8] trough paths A and B (Arbitrary labels in **Figure S158**).

**Structure**[a] (as labelled in **Figure S158**)	**Number of Imaginary modes and** $\boldsymbol{\upsilon}$ **(cm^{-1})**	$G^{o}_{aq}(X)$ **(ha)**
$\mathbf{2^{2+}}$	none	-84.97626853
CB[8]	none	-286.09002687
**PATH A**		
$\mathbf{I_1}$	none	-371.0792011
$\mathbf{TS_1}$	1 (-19.77)	-371.0663415
$\mathbf{I_m}$	none	-371.1087562
**PATH B**		
$\mathbf{I_m}$	none	-371.1087562
$\mathbf{TS_2}$	1 (-15.35)	-371.057233
$\mathbf{I_2}$	none	-371.0922015
$\mathbf{TS_3}$	1 (-21.90)	-371.0761417
$\mathbf{I_3}$	none	-371.0839689

The same approach described above was used for modelling the exchange mechanism for the phenyl rings on the $\mathbf{2^{2+}}$□CB[8] complex, considering in this case as initial guesses for the endpoints: a) the representative minimum for the complex ($\mathbf{I_m}$), and the manually-generated structure obtained by exchanging the arbitrary labels of the phenyl ring inserted on the cavity of CB[8] and one of the phenyl groups located outside the receptor. The obtained **path C** is depicted in **Figure 5** of the manuscript, with targeted energy quantities and frequencies being summarized in **Table S30.**

Table S37: Targeted quantities $\boldsymbol{G^{o}_{aq,mrrho}(X)}$ for each of the local minima and transition state found for path C concerning the Ph exchange in $\mathbf{2^{+}\subset CB[8]}$, as depicted and arbitrary labelled in **Figure 5** of the manuscript

**Structure**[a]	**Number of Imaginary modes and** $\boldsymbol{\upsilon}$ **(cm^{-1}))**	$G^{o}_{aq}(X)$ **(ha)**
$\mathbf{I_m}$	none	-371.1087562
$\mathbf{TS_2}$	1 (-6.85)	-371.08460850
$\mathbf{I_2}$	none	-371.08563853
$\mathbf{TS_3}$	1 (-47.58)	-371.07927217
$\mathbf{I_3}$	none	-371.08330219
$\mathbf{TS_4}$	1 (-11.52)	-371.07886968
$\mathbf{I_4}$	none	-371.08315639

**TS_5**	1 (-7.50)	-371.08189765

7.3.2. Guest 7^{2+}

The very same computational workflow discussed inSection 6.3.1. to study the ingression-eggresion and phenyl exchange of guest $\mathbf{2}^{2+}$ was applied to guest $\mathbf{7}^{+}$, producing the kinetic profile shown in **Figure S159**.

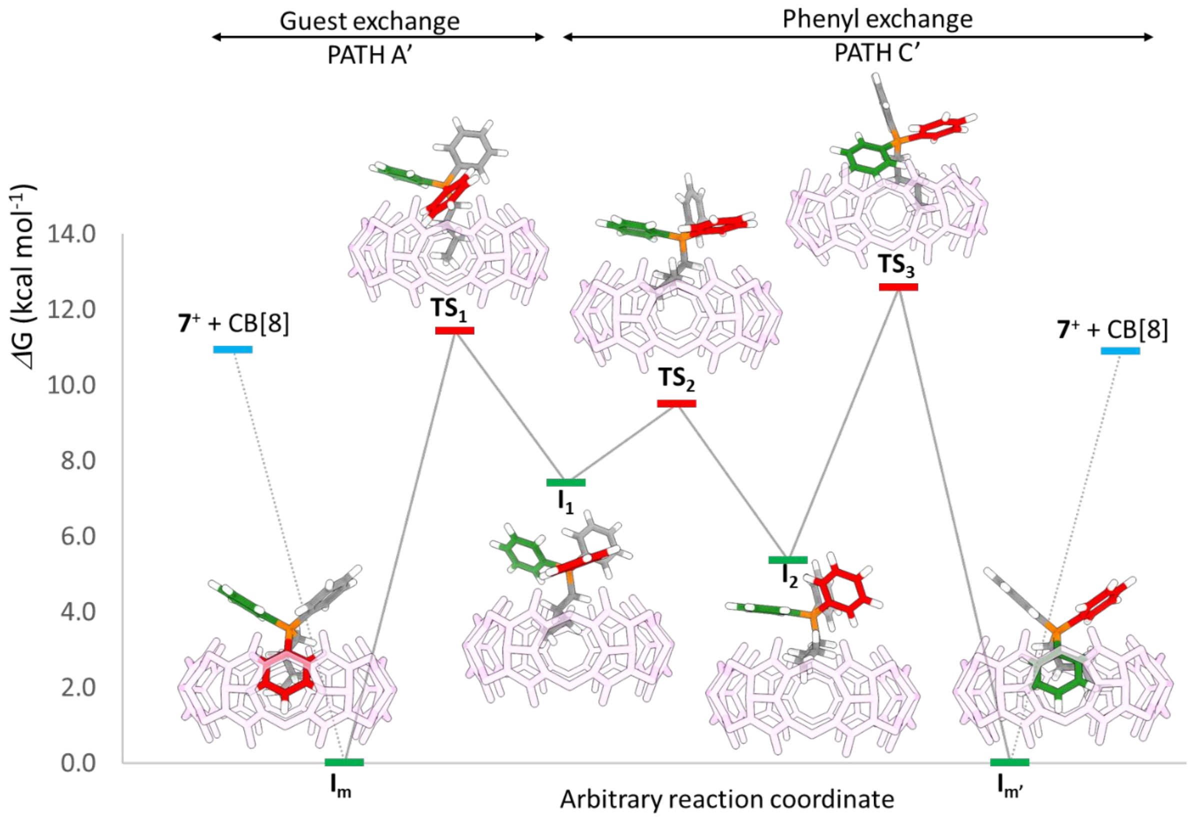

Figure S159: Depiction of the reaction path found for ingression-egression of guest $\mathbf{7}^{+}$ into the cavity of CB[8] (path A'), and phenyl exchange (path C'), both at the GFN2-xTB/ALPB(H_2O) level. Free energy barriers calculated from $\boldsymbol{G}^{\circ}_{\boldsymbol{aq,mrrho}}(\boldsymbol{X})$ values obtained from the corresponding frequency calculations (summarised in **Table S31**). Colour scheme as in Figure S2, exchanging phenyl rings marked in red and green.

Table S38: Targeted quantities $\boldsymbol{G^{\circ}_{aq,mrrho}(X)}$ for each of the local minima and transition states found for the ingression-egression of guest **7**$^+$ into the cavity of **CB[8]** (path A'), and phenyl exchange (path C'), both at the GFN2-xTB/ALPB(H_2O) level.

**Structure**[a] (as labelled in **Figure S159**)	**Number of Imaginary modes and υ (cm^{-1})**	$\boldsymbol{G^{\circ}_{aq}(X)}$ **(ha)**
**7**$^+$	none	-61.1718038
CB[8]	none	-286.09002687
**PATH A' (guest exchange)**		
**Im**	none	-347.2762441
**TS_1**	-21.87	-347.258042
**I_1**	none	-347.2644213
**PATH C' (phenyl exchange)**		
**Im**	none	-347.2762441
**TS_1**	-21.87	-347.258042
**I_1**	none	-347.2644213
**TS_2**	-38.38	-347.2610987
**I_2**	none	-347.2676858
**TS_3**	-28.18	-347.256176
**$I_{m'}$**	none	-347.2766665
